# Independent Evolution of Strychnine Recognition by Bitter Taste Receptor Subtypes

**DOI:** 10.3389/fmolb.2018.00009

**Published:** 2018-03-02

**Authors:** Ava Yuan Xue, Antonella Di Pizio, Anat Levit, Tali Yarnitzky, Osnat Penn, Tal Pupko, Masha Y. Niv

**Affiliations:** ^1^Robert H. Smith Faculty of Agriculture, Food and Environment, Institute of Biochemistry, Food Science and Nutrition, The Hebrew University of Jerusalem, Rehovot, Israel; ^2^The Fritz Haber Center for Molecular Dynamics, The Hebrew University of Jerusalem, Jerusalem, Israel; ^3^Department of Pharmaceutical Chemistry, University of California, San Francisco, San Francisco, CA, United States; ^4^Tali Yarnitzky Scientific Consulting, Maccabim-Reut, Israel; ^5^Modeling, Analysis and Theory Group, Allen Institute for Brain Science, Seattle, WA, United States; ^6^The Department of Cell Research and Immunology, George S. Wise Faculty of Life Sciences, Tel Aviv University, Tel Aviv, Israel

**Keywords:** bitter taste receptor, homology modeling, ligand recognition, functional residues, evolution, ancestor functionality, ancestral reconstruction, phylogenetics

## Abstract

The 25 human bitter taste receptors (hT2Rs) recognize thousands of structurally and chemically diverse bitter substances. The binding modes of human bitter taste receptors hT2R10 and hT2R46, which are responsible for strychnine recognition, were previously established using site-directed mutagenesis, functional assays, and molecular modeling. Here we construct a phylogenetic tree and reconstruct ancestral sequences of the T2R10 and T2R46 clades. We next analyze the binding sites in view of experimental data to predict their ability to recognize strychnine. This analysis suggests that the common ancestor of hT2R10 and hT2R46 is unlikely to bind strychnine in the same mode as either of its two descendants. Estimation of relative divergence times shows that hT2R10 evolved earlier than hT2R46. Strychnine recognition was likely acquired first by the earliest common ancestor of the T2R10 clade before the separation of primates from other mammals, and was highly conserved within the clade. It was probably independently acquired by the common ancestor of T2R43-47 before the homo-ape speciation, lost in most T2Rs within this clade, but enhanced in the hT2R46 after humans diverged from the rest of primates. Our findings suggest hypothetical strychnine T2R receptors in several species, and serve as an experimental guide for further study. Improved understanding of how bitter taste receptors acquire the ability to be activated by particular ligands is valuable for the development of sensors for bitterness and for potential toxicity.

## Introduction

Bitterness perception is considered a key defense mechanism against the ingestion of potentially toxic substances (Chandrashekar et al., 2000). The recognition of bitter molecules is mediated by a set of chemosensory receptors denoted as taste receptors type 2 (T2Rs or TAS2Rs), which belong to the superfamily of G protein-coupled receptors (GPCRs). T2Rs are classified as a subfamily of Class A (rhodopsin-like) GPCRs. This class comprises over 80% of all GPCRs in human, and includes receptors that are involved in a wide range of physiological functions in vision, olfaction, taste, immune, cardiovascular and neurological systems, and more (Venkatakrishnan et al., 2014; Di Pizio et al., 2016; Wacker et al., 2017).

Human T2Rs (hT2Rs) are expressed in the type II taste bud cells in the oral cavity (Adler et al., 2000), and in several tissues throughout the body, where they are suggested to regulate food intake in the intestine (Avau et al., 2015), serve as immunity sentinels in the airway muscles (Liggett, 2013), and affect the maturation process of sperm (Trivedi, 2012). The 25 hT2Rs recognize bitter compounds of wide chemical diversity (Wiener et al., 2012; Di Pizio and Niv, 2015; Behrens and Meyerhof, 2017). Furthermore, taste prediction using BitterPredict computational tool indicates that over 77% of the natural products may have some bitterness (Dagan-Wiener et al., 2017). Many T2Rs, such as human T2R14, T2R10, and T2R46 are activated by many chemically diverse bitter compounds, while other subtypes are more selective (Di Pizio and Niv, 2015; Behrens and Meyerhof, 2017).

Despite the sequence conservation of T2Rs among vertebrates (Bachmanov et al., 2014), the number of *T2R* genes and the proportion of pseudogenes differ widely across species, indicating gene expansions and contractions during evolution (Sugawara et al., 2011). As an example, no *T2R* gene was detected in the bottlenose dolphin (Jiang et al., 2012), turkey and chicken have only 2 and 3 functional T2Rs, respectively, while ~50 T2Rs have been identified in the Western clawed frog (*Xenopus tropicalis*) (Behrens et al., 2014); 44% of *T2R* genes in cows are pseudogenized vs. 12% in rat (Sugawara et al., 2011). Such variation could reflect adaptation to specific environmental challenges, and diets in particular. For instance, extensive loss of bitter taste perception has been found in aquatic mammals such as cetaceans and pinnipedia, which swallow food whole (Kishida et al., 2015; Liu et al., 2016), and the percentage of *T2R* pseudogenes is 10 times higher in vampire bats, which exclusively feed on blood and do not tend to accidentally consume other (potentially toxic) items, compared to non-vampire bats (Hong and Zhao, 2014).

Interestingly, protein sequence similarities among T2Rs do not correspond to overlapping ligand spectra. The highly similar hT2R43-50 cluster (over 70% sequence identity) displays remarkably varied tuning breadth, while hT2R10 and hT2R46 share many common agonists, despite their sequence identity being only 34% (Born et al., 2013).

Strychnine is a potent neurotoxin that elicits a distinct bitter taste. It is most commonly derived from the seeds of Strychnos nux-vomica, a deciduous tree native to India and Southeast Asia (USDA PLANTS db: https://plants.usda.gov/core/profile?symbol=STNU4)^1^. The only two human bitter taste receptors activated by strychnine in cell-based assays are hT2R10 and hT2R46 subtypes (Meyerhof et al., 2010). Human T2R46 is more sensitive to strychnine, with an effective concentration as low as 0.1 μM, whereas hT2R10 activation threshold for strychnine is 30 times higher (Brockhoff et al., 2010; Meyerhof et al., 2010; Born et al., 2013).

Using homology modeling, molecular docking, site-directed mutagenesis, and functional assays. Meyerhof et al. have shown that hT2R10 and hT2R46 display different binding modes for strychnine within the TM binding cavity (Reichling et al., 2008; Brockhoff et al., 2010; Meyerhof et al., 2010; Born et al., 2013; Sandal et al., 2015). This is indicative of an independent acquirement of strychnine responsiveness during the evolution of the two receptors rather than descending from a common ancestral binding mode (Born et al., 2013). Independent evolution by specific amino acid replacements within T2Rs was suggested as a general mechanism for adaptation to specific dietary sources by different primate species (Imai et al., 2012). In order to understand how bitter taste receptors acquired the ability to be activated by their ligands, we investigated the possible evolutionary history of molecular recognition of a single bitter compound, strychnine, that activates two human T2R (hT2R) subtypes. By examining the similarities and disparities, as well as mutation rates, within previously elucidated strychnine binding sites in human T2R10 and T2R46, and by projecting binding site information to related T2R sequences from other species, we retrace the history of strychnine recognition acquired by bitter taste receptors.

## Results and discussion

Sequences of hT2R46- and hT2R10-like proteins were obtained from NCBI, and their multiple sequence alignment was constructed as detailed in Methods. The analysis of phylogeny and reconstructed ancestors was based on the full sequences. Next, key ligand-binding positions that were elucidated in previous studies (Reichling et al., 2008; Brockhoff et al., 2010; Born et al., 2013; Sandal et al., 2015) were analyzed, and, using known mutagenesis data, the potential effects on strychnine interactions due to evolutionary variation at these positions were predicted. hT2R46 and hT2R10 models in complex with strychnine were used to discuss the roles of key positions in the 3D structural context. Finally, the predicted site-specific selective pressure was analyzed.

### Phylogeny with predicted common ancestors

**Figure 1:**
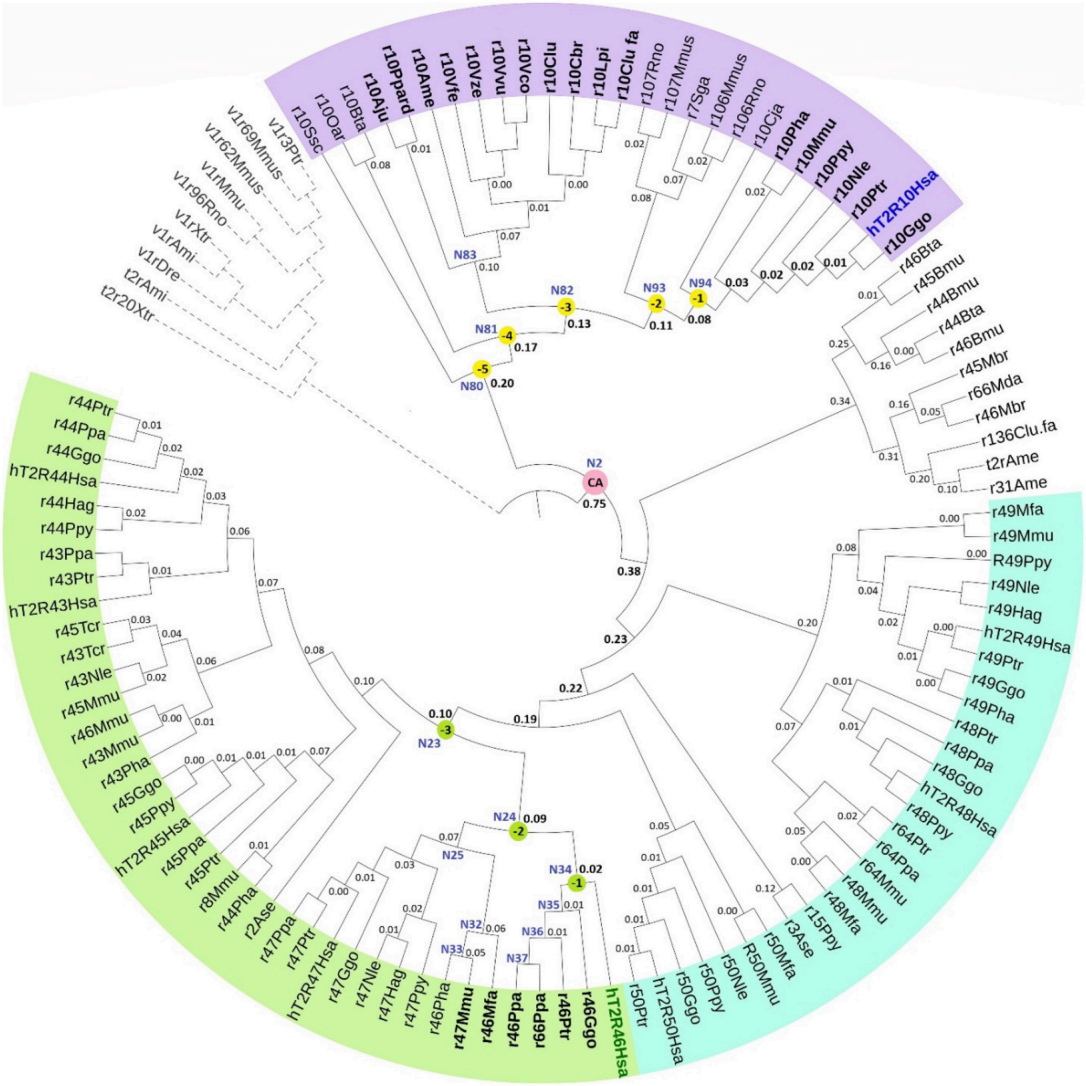
Phylogeny with reconstructed common ancestors and estimated relative divergence times. Divergence times are displayed near the corresponding nodes. Color ranges: purple, sequences having >60% identity with hT2R10; cyan, sequences having >65% identity with hT2R46; green, sequences having >80% identity with hT2R46. Yellow dots: N94 (−1) through N80 (−5), the five most recent common ancestor of hT2R10 clade, with N94 (−1) being the most recent and N80 (−5) the earliest. Green dots: common ancestors N34 (−1), N24 (−2), and N23 (−3) in the hT2R46 clade. Pink dot: N2, the common ancestor of hT2R10 and hT2R46. N83: the common ancestor of canidae (Vfe, Vze, Vvu, Vco, Clu, Cbr, Lpi, Clu.fa) and felidae (Ppard, Aju). N35-N37: the common ancestors of gorilla (Ggo) T2R46, chimp (Ptr) T2R46, and bonobo (Ppa) T2R46 and T2R66, respectively. N32-N33: common ancestor of Rhesus macaque (Mmu) T2R47, Hamadryas baboon (Pha) T2R46 and crab-eating macaque (Mfa) T2R46, and common ancestor of the first two, respectively. N25: common ancestor of primate T2R47, the closest sister clade of T2R46. Outgroup clade in gray dashed lines. Tree was constructed using iTOL tool (Letunic and Bork, 2016). The original RelTime tree constructed in MEGA7 (Kumar et al., 2016) is shown in Supplementary 3, Figure S1.

The phylogenetic tree with reconstructed common ancestors and estimated relative divergence times is shown in Figure 1. The predicted common ancestors are located at each branching point (node) labeled as “N#,” with smaller numbers indicating earlier events along the timeline within the same clade. The following common ancestors were selected for analysis: the ancestor common to all primates T2R10 (N94) and the direct ancestor of hT2R46 (N34) are both marked “(−1)”; the next most recent common ancestor of primates T2R10 and their homologs from rodents (*Rattus norvegicus*, Rno and *Mus musculus*, Mmus) (N93) and the one common to the T2R46 and T2R47 clades (N24) are labeled as “(−2)”; the ancestor common to all the predicted strychnine binders in T2R10 clade, N82, and N23 encompassing all the T2R43-47 subtypes in primates are labeled as “(−3),” “(−4),” and “(−5)” denote the next two most recent common ancestors in T2R10 clade, corresponding to N81which includes hT2R10 homologs from cow (*Bos taurus*, Bta) and sheep (*Ovis aries*, Oar) and N80 with the addition of pig (*Sus scrofa*, Ssc) T2R10. Finally, N2 denotes the earliest common ancestor of the entire group of interest (ingroup, IG), including both hT2R10 and hT2R46 clades.

Robustness of the tree topology and of prediction of ancestral sequences were tested by varied outgroup (OG) samplings and ingroup selections (Supplementary 1). OG sequences are outside of the group of interest but still close relatives (Wilberg, 2015). As an essential component of phylogenetic analysis, OG selection can influence branch order and length, clade monophyly, as well as divergence rates (Puslednik and Serb, 2008). Increased OG sampling has been shown to improve the stability of tree topology (Nixon and Carpenter, 1993). The resulting tree structure was highly consistent across different OG samples. However, increased variation of the predicted amino acids was found in earlier reconstructed common ancestors with different OG and IG samplings. This is in accord with declining posterior probability (an estimate of confidence in each inferred ancestral character at a specific position) of the ancestral sequence reconstruction, even though the overall tree structure remained largely unaffected. The common ancestor of hT2R10 and hT2R46 is especially prone to the influence of OG and IG selections, exhibiting high variability in the predicted residues in key positions for strychnine detection (Supplementary 2, tab “Summary”).

### Estimated divergence times

Relative evolutionary timescales were estimated using RelTime (Tamura et al., 2012; Mello et al., 2017) to determine the relative ordering and spacing of the evolutionary process of T2R10 and T2R46. The number on the branch (Figure 1) denotes the relative time lapsed from the previous node. Hence, a smaller number indicates faster divergence rate or shorter interval from the most recent common ancestor.

According to the constructed phylogeny with estimated relative times of divergence, T2R43-47 (Figure 1, green range) evolved more recently compared to T2R10s (purple range) through a series of diversification events from their common ancestor N23. Acceleration of gene duplication was accompanied with increased functional divergence in the T2R43-47 group, as indicated by the relative times of divergence becoming smaller further down the branches (Figure 1). The hypothesized relative divergence times shown on the branches also reveal that among the five human T2R receptors in this clade, hT2R46 diverged first, followed by hT2R47, hT2R45, hT2R43, and lastly hT2R44.

In contrast to hT2R46, the hT2R10 sequence is inferred to have evolved much earlier on the time-tree with a lower relative divergence rate of 1.52 (vs. 1.98 for hT2R46) (Figure 1). Human T2R10 appears to have diverged last among all the sampled T2R10s in primates, canids, felidae, and panda. This is different from human T2R46, which seems to have diverged later on, but prior to T2R46s in primates, such as gorilla, chimp, and bonobo. The next section explores which of the homologs and ancestral receptors are likely to have the ability to bind strychnine.

### Analysis of the key functional residues

Throughout the text, residue positions are indicated using the Ballesteros–Weinstein (BW) numbering scheme, in which the most conserved residue in a given TM domain X is assigned the index X.50, and the remaining TM residues are numbered relative to this position (Ballesteros and Weinstein, 1995). Particularly, BW numbering is shown as superscripts.

Site-directed mutagenesis, functional assays, and modeling studies were previously carried out to elucidate strychnine binding site in hT2R10 (Born et al., 2013) and in hT2R46 (Reichling et al., 2008; Brockhoff et al., 2010; Sandal et al., 2015). Residues that, according to these studies, were shown to be involved in strychnine binding either in the hT2R10-like or in hT2R46-like binding mode are designated as key positions (Table 1).

**Table 1:** Predicted residues in known key positions for strychnine-binding with posterior probability in selected ancestral sequences.

**Key positions**	**hT2R10**	**N94 (−1)**	**N93 (−2)**	**N83**	**N82 (−3)**	**N81 (−4)**	**N80 (−5)**	**hT2R46**	**N35-37**	**N34 (−1)**	**N33**	**N32**	**N25**	**N24 (−2)**	**N23 (−3)**	**CA N2**
2.61	G65	G	G	G	G	G	G	**W66**	**W**	**W**	**W**	**W**	**W**	**W**	**W**	***G***
2.65	I69	I	I	I	I	I	I	**E70**	**E**	**E**	*V*	*V*	*V*	*V*	*V*	***V***
2.66	F70	F	F	F	F	F	F	**L71**	**L**	**L**	**L**	**L**	**L**	**L**	**L**	***F***
3.25	I81	I	I	I	I	I	I	**I82**	**I**	**I**	*T*	*T*	**I**	**I**	**I**	**I**
3.29	**S85**	**S**	**S**	**S**	**S**	**S**	**S**	Y85	Y	Y	Y	Y	Y	Y	Y	**S**
3.32	**W88**	**W**	**W**	**W**	**W**	**W**	**W**	**W88**	**W**	**W**	**W**	**W**	**W**	**W**	**W**	**W**
3.33	**V89**	**V**	**V**	**V**	**V**	**V**	**V**	A89	A	A	*V*	A	A	A	A	***T***
3.36	**N92**	**N**	**N**	**N**	**N**	**N**	**N**	**N92**	**N**	**N**	**N**	**N**	**N**	**N**	**N**	**N**
3.37	**Q93**	**Q**	**Q**	**Q**	**Q**	**Q**	*H*	H93	H	H	H	H	H	H	H	***H***
4.65	I150	I	I	I	I	I	I	**N150**	**N**	**N**	**N**	**N**	**N**	**N**	**N**	***D***
ECL2	D159	N	N	N	N	S	S	**N161**	**N**	**N**	**N**	**N**	**N**	**N**	**N**	**N**
5.39	**K174**	**K**	**K**	**K**	**K**	*N*	*N*	**N176**	**N**	**N**	**N**	**N**	**N**	**N**	**N**	**N**
5.40	**Q175**	**Q**	**Q**	**Q**	**Q**	**Q**	**Q**	T177	T	T	M	M	M	M	M	***M***
5.42	**L177**	**L**	**L**	**L**	**L**	**L**	**L**	**V179**	**V**	**V**	**V**	**V**	**V**	**V**	**V**	**L**
5.43	**L178**	**L**	**L**	**L**	**L**	**L**	**L**	T180	T	T	T	T	T	T	T	**L**
6.51	**Y239**	**Y**	**Y**	**Y**	**Y**	**Y**	**Y**	**Y241**	**Y**	**Y**	**Y**	**Y**	**Y**	**Y**	**Y**	**Y**
6.63	T251	T	T	T	T	T	T	**E253**	**E**	**E**	**E**	**E**	**E**	**E**	**E**	***N***
7.39	**M263**	**M**	**M**	**M**	**M**	**M**	**M**	**E265**	**E**	**E**	*Q*	*Q*	*Q*	*Q*	*Q*	**M**
7.42	**T266**	**T**	**T**	**T**	**T**	**T**	**T**	**A268**	*T*	*T*/A^*^	*I*	*I*	*I*	**A**	*R*	**A**
7.43	A267	V	V	V	V	V	V	**F269**	**F**	**F**	**F**	**F**	**F**	**F**	*I*	***I***

Posterior probability legend:

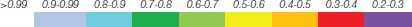

Font colors of the key positions, designated by BW numbering:

*Blue bold—residues important for strychnine recognition in hT2R10*.

*Green bold —residues important for strychnine recognition in hT2R46*.

*Gray italic—residues that differ in the predicted ancestral sequences compared to those in hT2R10 or hT2R46*.

* *Due to high variability at A268^7.42^ in the hT2R46 clade, the two predictions from different IG/OG samplings exhibited similar confidence level and were both included*.

**Figure 2:**
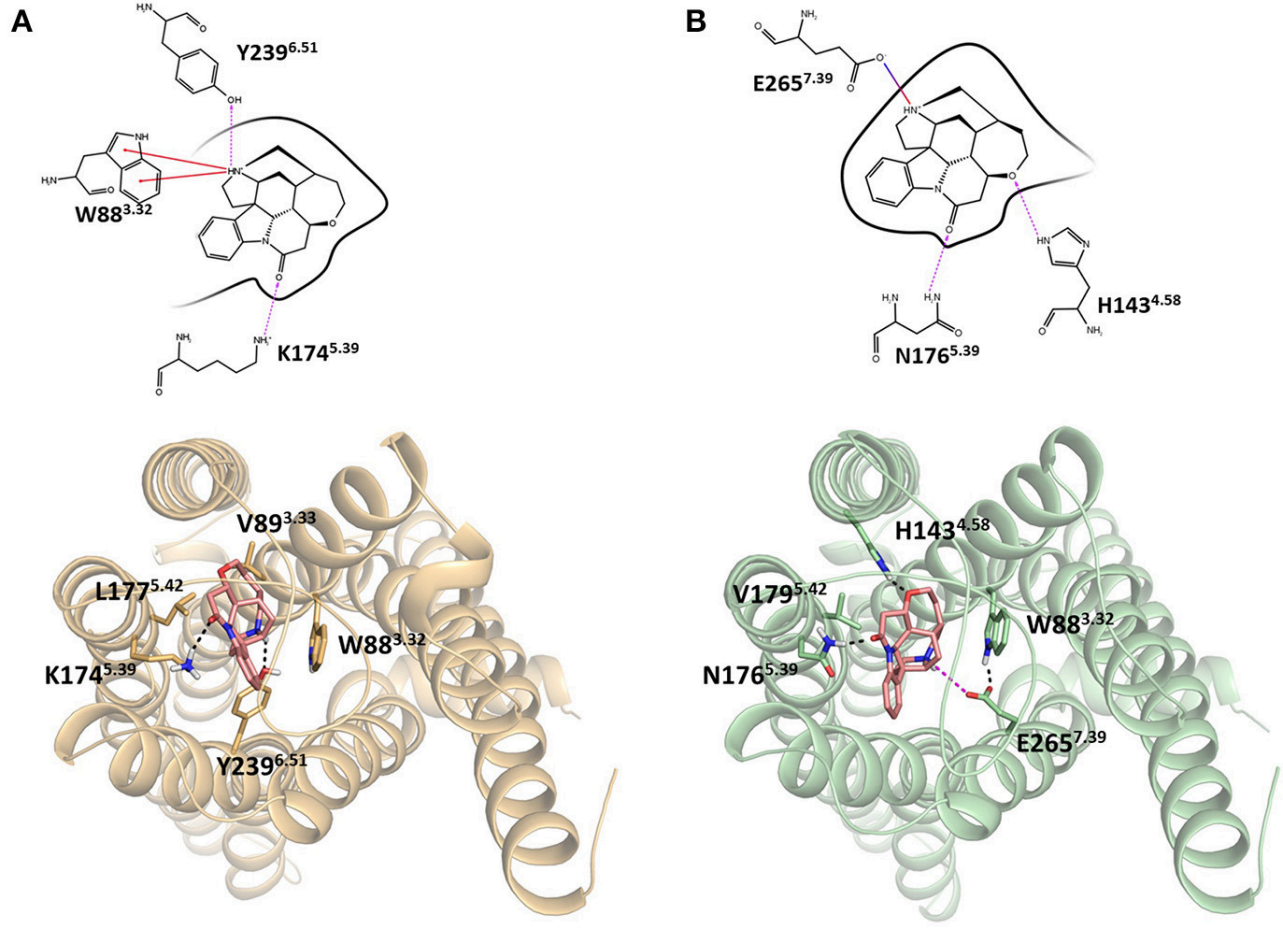
Homology models of **(A)** hT2R10 and **(B)** hT2R46 with strychnine docked into the putative orthosteric binding sites. Docking scores, used as an approximation of binding affinity, are of −9.619 kcal/mol for hT2R46/strychnine complex and of −7.949 kcal/mol for hT2R10/strychnine complex. Indeed, hT2R46 is more sensitive to strychnine respect to hT2R10. 2D diagrams depicting the predicted ligand interactions with binding site residues are shown in top panels. Hydrogen bonds are shown as magenta dashed lines, cation-π interactions as red lines, salt-bridge as blue-red lines. Bottom panels present extracellular views of the orthosteric binding sites, with strychnine shown as sticks with pink carbons, and residues interacting with strychnine shown as tan (hT2R10) or light green (hT2R46) sticks. Hydrogen bonds are shown as black dashed lines, salt-bridge as magenta dashed lines. Figure was created using the Ligand Interaction Diagram available in Maestro (2D) and Pymol (3D).

Integrating receptor modeling and induced fit docking calculations, new homology models were created in the current study and provide a working model of binding, that is consistent with experimental data (Figure 2). Strychnine in complex with hT2R10 establishes H-bonds with Y239^6.51^ and K174^5.39^, hydrophobic interactions with W88^3.32^, V89^3.33^, and L177^5.42^; while it forms H-bonds with H143^4.58^ and N176^5.39^, a salt-bridge with E265^7.39^, and hydrophobic contacts with W88^3.32^ and V179^5.42^ in hT2R46. Most of the residues found to be relevant for strychnine-binding experimentally (Table 1) indeed establish direct contacts with strychnine in the models (Figure 2). Some (e.g., positions 3.36 and 3.37) do not, and this may be either a shortcoming of the model, the lack of representation of water molecules that might be mediating ligand-protein interactions, or due to other indirect effect of mutation on activation.

In order to track the potential ability to recognize strychnine during evolution, ancestral sequences were reconstructed (see Methods for details) and residues in key positions were analyzed. Increased variability in key positions was observed in the common ancestors that evolved earlier and farther away from the terminal branches. Consequently, those ancestors that are more closely related to hT2R10 or hT2R46 are predicted to be more likely to recognize strychnine in the same binding mode than the rest of the ancestors. Table 1 lists the key positions of the ancestral sequences; the sequences are listed from left to right in the order of descending evolutionary relationship.

Common ancestor (−1) in both hT2R10 and hT2R46 clades displays the highest conservation of key residues, whereas (−5) in hT2R10 clade and (−3) in hT2R46 clade are inferably least likely to bind strychnine due to the alterations in key positions. Common ancestors of the adjacent clades (i.e., N83, N35-37, N32-33, and N25) and the common ancestor of hT2R10 and hT2R46 (N2) are also presented for comparison. Full sequence multiple sequence alignment (MSA) of hT2R10 and hT2R46 clades including the ancestral sequences listed in Table 1 are available in FASTA format in Supplementary 1.

#### The hT2R10-like sequences

**Figure 3:**
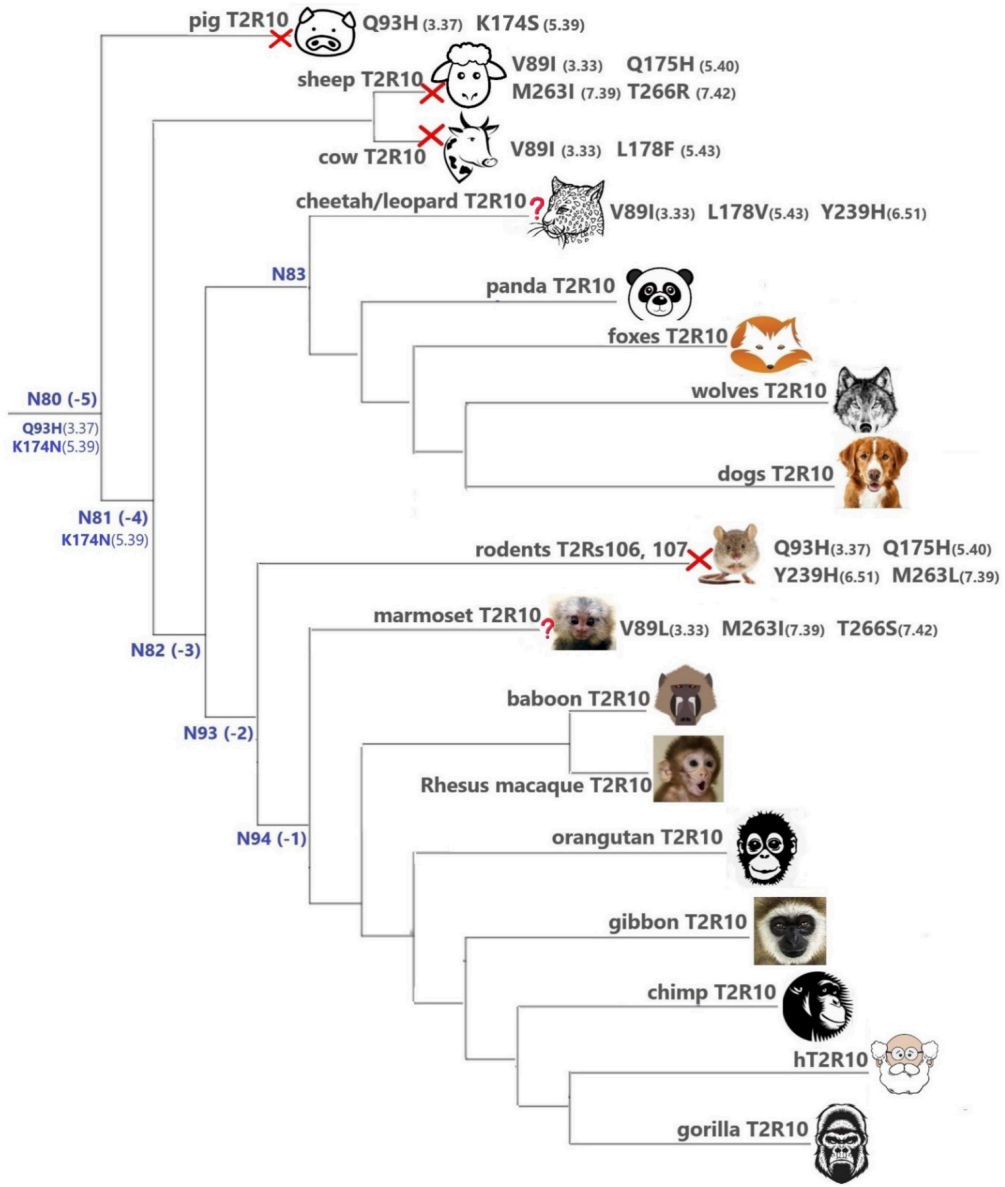
T2Rs that are the most hT2R10-similar; the majority have 100% identical key residues for strychnine recognition and therefore are predicted as strychnine binders; a predicted non-binder is marked with a red cross next to it and an uncertain prediction with a question mark. Variations of key residues are listed next to the species. Common ancestors within the T2R10 clade in Figure 1 are also presented here in blue with predicted key residue variations. Baboon image created by © Kenneth Chiou, in “Population genomics of a baboon hybrid zone in Zambia”, http://kennychiou.com/dissertation/#species.

Overall, T2R10 is highly conserved within several species of primates (posterior probability > 0.99), canidae, felidae, and even panda (*Ailuropoda melanoleuca*, Ame), which is the only extant genus in the bear subfamily *Ailuropodinae* since over 2 million years ago (Jin et al., 2007). We find that the key positions (see Figure 2 and Table 1) are identical to those in hT2R10, suggesting their intact ability to bind strychnine, in T2R10s from the following 14 organisms: chimpanzee (*Pan troglodytes*, Ptr), Western gorilla (*G. gorilla*, Ggo), Nomascus gibbon (*Nomascus leucogenys*, Nle), Rhesus macaque (*Macaca Mulatta*, Mmu), Bornean orangutan (*Pongo pygmaeus*, Ppy), Hamadryas baboon (*Papio hamadryas*, Pha), wolf (*Canis lupus*, Clu), African hunting dog (*Lycaon pictus*, Lpi), Tibetan fox (*Vulpes ferrilata*, Vfe), Corsac fox (*Vulpes corsac*, Vco), red fox (*Vulpes vulpes*, Vvu), maned wolf (*Chrysocyon brachyurus*, Cbr), fennec fox (*Vulpes zerda*, Vze), and panda (Ame). Leopard (*Panthera pardus*, Ppard) and cheetah (*Acinonyx jubatus*, Aju) T2R10s may also bind strychnine—in spite of the three altered key residues (Figure 3).

In contrast, T2R10s in pig (Ssc) with Q93^3.37^H and K174^5.39^S variations, cow (Bta) with V89^3.33^I and L178^5.43^F, and sheep (Oar) with V89^3.33^I, Q175^5.40^H, M263^7.39^I, and T266^7.42^R, are not likely to recognize strychnine due to several altered key functional residues (Figure 3; Supplementary 2, tab “R10-likeKeyResidues”). Specifically, positions 5.40, 5.43, and 7.42 were found to be relevant for hT2R10 activation by strychnine in mutagenesis studies (Born et al., 2013). There is no available mutagenesis data for K174^5.39^, but our modeling suggests that it may be involved in H-bond interaction with strychnine (Figure 2).

Experimental data showed that single mutations in positions 3.37 and 7.39 affect activation by strychnine, and Q93A and M263A/E led to complete loss of responsiveness for all agonists tested (Born et al., 2013). In rodents, 3.37 and 7.39 correspond to Q39H and M263L, therefore rodent T2R106 and T2R107 are not predicted to be activated by strychnine. Indeed, mouse T2R106 and T2R107 were not activated by any ligand tested in the study, including strychnine (Lossow et al., 2016). Thus, these subtypes may be either non-functional or still orphan. According to Lossow et al. (2016), the only two mouse T2R subtypes that can be activated by strychnine are T2R117 and T2R140, which were not included in our study because due to low protein sequence identity (<40%) with hT2R10 and hT2R46, suggesting the presence of other ways of T2R-strychnine interaction.

In marmoset (*Callithrix jacchus*, Cja) T2R10, the variants V89^3.33^I, M263^7.39^I, and T266^7.42^S involve chemically similar residues, therefore it is difficult to predict their effect on the strychnine-binding ability (Figure 3).

It is puzzling that certain hypercarnivores, such as wolves, are predicted to be more strychnine-sensitive than exclusively herbivorous animals, such as sheep and cow. However, hypercarnivores are exposed to plant material indirectly from the viscera of their prey (Shang et al., 2017). Ruminants, on the other hand have been found to be less sensitive to bitter taste than other mammals, which could be due to their dependence on plant-based diets which usually accompany rather high occurrence of bitterness (Ginane et al., 2011). In fact, horses were found to be able to tolerate relatively large amounts of strychnine and cows were even more resistant to strychnine ingestion, presumably because of its partial breakdown in the rumen (Humphreys and Myra, 1988). Given the wide range of ligands for T2Rs (Wiener et al., 2012; Di Pizio and Niv, 2015), conservation or variation of T2Rs can be driven by various chemicals unrelated to strychnine. It is also possible that the strychnine recognition function in sheep and cow is compensated by another T2R subtype, or that the T2R10 of sheep and cow has a different strychnine binding mode. Moreover, the fact that all key residues were preserved in the T2R10 of panda, which only feeds on bamboo, may suggest a lower tolerance on other plants and hence stronger ability to recognize strychnine. It is also possible that the variations in other residues of panda T2R10 could dramatically hinder strychnine binding and that the prediction of panda T2R10 to be a strychnine-binder is false positive. Intriguingly, the known orthologs of hT2R10 came mostly from omnivores (primates) and carnivores (canidae and felidae). Figure 3 summarizes the most hT2R10-similar T2Rs with their key residue variations, and the inferred ability or inability to recognize strychnine.

#### T2R10 clade predicted common ancestors

The common ancestors of primates, canidae, panda and felidae [N94 (−1), N93 (−2), N83, and N82 (−3); Figures 1, 3] were all predicted to have 100% conservation in the key positions and hence were likely to bind strychnine (Table 1). The common ancestor of sheep and cow T2R10s, N81, harbors all the key residues for strychnine recognition except K174^5.39^N, which may affect H-bond formation with the ligand. Moreover, N80, the earliest ancestor of the T2R10 clade, may not be activated by strychnine either because of an additional variation Q93^3.37^H (Table 1). As reported by Born et al. (2013), Q93^3.37^A in hT2R10 resulted in a complete loss of responsiveness to all agonists, but the effect of Q93^3.37^H mutation remains to be tested.

#### The hT2R46-like sequences

Within the hT2R46-like sequences (Figure 1), predicted ancestral sequences amino acids in the determining positions for strychnine recognition exhibit much higher variability than those in the T2R10 clade. E265^7.39^K, A268^7.42^R, and F269^7.43^N, causing almost a complete loss of responsiveness, were identified as key residues for T2R46 activation by strychnine (Brockhoff et al., 2010). Particularly, residues E265^7.39^ and A268^7.42^ were found to be especially important for strychnine recognition, showing that transferring E265^7.39^ and A268^7.42^ into the corresponding positions into the strychnine insensitive hT2R31 and hT2R43 is enough to confer strychnine sensitivity. In addition, the crucial role of a negatively charged group in position 7.39 was underlined by the same study, as substituting Glu with Gln reduced hT2R46 responsiveness to strychnine by almost 100-fold, while substituting with Lys nearly abolished receptor activation. As previously found (Brockhoff et al., 2010), our docking studies confirm that the negatively charged carboxylate anion of E265^7.39^ forms a salt-bridge with the positively charged nitrogen of strychnine. In addition to direct interaction with the agonist, E265^7.39^ establishes intramolecular H-bonds with W88^3.32^ and Y241^6.51^ and helps shape the binding cavity, as suggested also by Sandal et al. (2015).

A268^7.42^ does not allow substitution with long side-chain residues: it has been found experimentally that A268^7.42^G only leads to slight decrease of sensitivity, but the A268^7.42^R mutant abolishes hT2R46 sensitivity to strychnine almost completely (Brockhoff et al., 2010). Hydrophobicity in position 7.43 was found to be fundamental for strychnine-binding (Brockhoff et al., 2010; Sandal et al., 2015, T2R46.pdb in Supporting information).

Accordingly, we suspect that hT2R46-like sequences which contain substitutions that lead to both altered negative charge in position 7.39 and altered hydrophobicity/steric effect in 7.42 or 7.43, i.e., would be unlikely to recognize strychnine.

**Figure 4:**
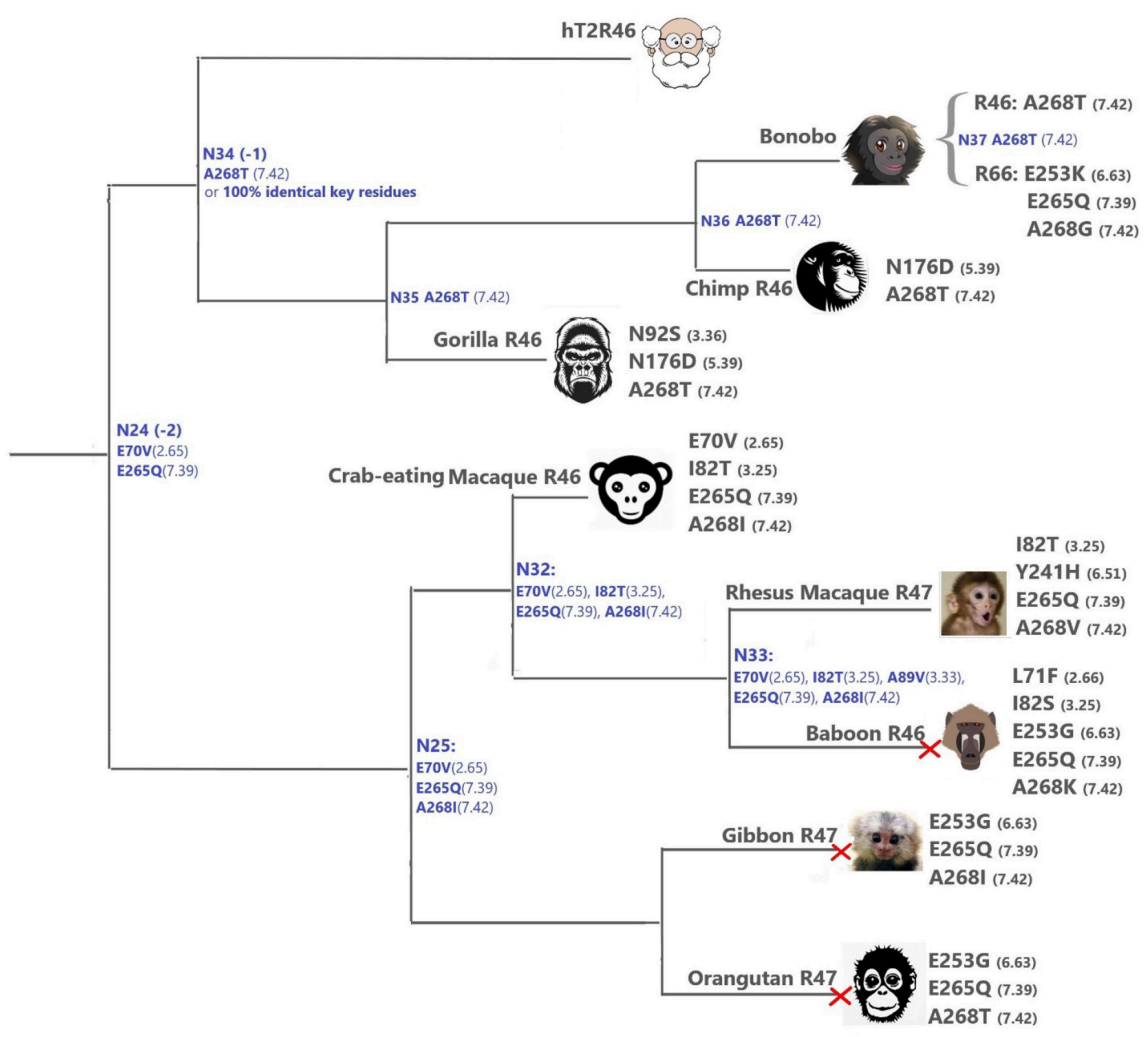
T2Rs that are the most hT2R46-similar, the majority of which are predicted to recognize strychnine, probably with largely reduced sensitivity compared to human; a predicted non-binder is marked with red cross next to it. Variations of key residues are listed next to the species. Most of the common ancestors within the hT2R46 and nearby clades in Figure 1 are also presented here in blue with predicted key residue variations (see “T2R46 Clade Predicted Common Ancestors”). Baboon image created by © Kenneth Chiou, in “Population genomics of a baboon hybrid zone in Zambia”, http://kennychiou.com/dissertation/#species.

The primates (Figure 4) T2Rs that are most closely related to hT2R46 vary in positions 2.65, 3.25, 3.36, 5.39, and 6.63, which are relatively tolerant to mutations (Figure 4). For instance, the point mutation E70^2.65^V resulted in largely unaltered activation by strychnine, and I82^3.25^T, N92^3.36^G, N176^5.39^D, and E253^6.63^G mutations led to decreased (rather than abolished) response to strychnine (Brockhoff et al., 2010; Sandal et al., 2015). Therefore, the hT2R46-related T2Rs from primates are likely to be strychnine binders, possibly with lower sensitivity than hT2R46.

Y241^6.51^F was found to reduce the maximum activation level (Brockhoff et al., 2010), while Y241^6.51^S showed a higher activation level (Sandal et al., 2015), demonstrating the importance of the H-bond between Y241^6.51^ and strychnine for hT2R46 activation (see Figure 2). Rhesus Macaque T2R10 has Histidine in this position, which may form hydrogen bonds with strychnine.

N92^3.36^S and E253^6.63^K in gorilla and bonobo T2R46, respectively, could possibly lead to reduced strychnine responsiveness. E253 is predicted to make an H-bond with K258^ECL3^. Despite the uncertainty of loop modeling, the change from a negative to a positive amino acid is expected to have dramatic effects on the receptor's functionality. Additionally, A268^7.42^T and A268^7.42^I may also reduce strychnine binding due to increased sterical hindrance.

When all the key residues are considered in light of the experimental results from the same study, the most plausible strychnine binders in the T2R46 clade are listed as follows: T2R46s from the chimp (Ptr), bonobo (*Pan paniscus*, Ppa), western gorilla (Ggo) and crab-eating macaque (*M. fascicularis*, Mfa), bonobo T2R66, and Rhesus macaque (Mmu) T2R47, although none of them contains all the 11 key residues as seen in hT2R46 (Figure 4; Supplementary 2, tab “R46-likeKeyResidues”). Notably, the 100% conservation of key functional residues and 99.99% identity of overall DNA sequence of Neanderthal T2R46 compared to hT2R46 indicates that the ability of strychnine recognition by T2R46 subtype was acquired before the divergence of Neanderthals and modern humans over 400,000 years ago (Hublin, 2009); Neanderthal T2R10 has 100% protein sequence identity with human T2R10. (DNA sequence alignments of Neanderthal T2R10 and T2R46 with hT2R10 and hT2R46, respectively, are available in Supplementary 1).

#### T2R46 clade predicted common ancestors

**Figure 5:**
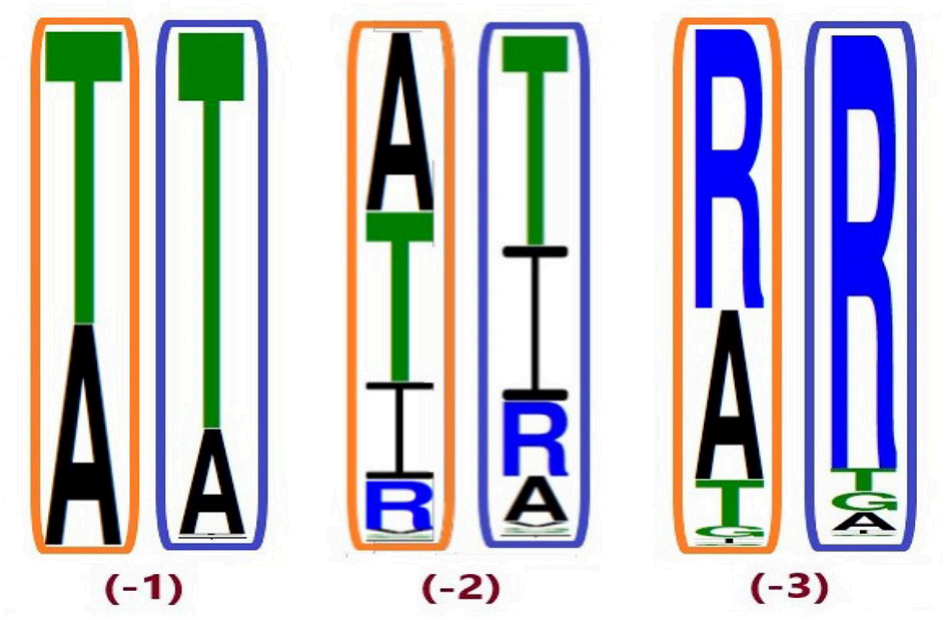
Conservation of position A268^7.42^ from different ingroup (IG) samplings, generated based on the 100 most likely ancestral sequences of hT2R46 by using FastML (Ashkenazy et al., 2012). Orange rectangles: IG with 55% identity cutoff; blue rectangles: enlarged IG with 45% identity cutoff. Height of amino acid symbols is proportional to their relative frequency at that position.

Despite higher variability of key functional residues in the T2R sequences within the T2R46 clade, the likelihood of the two most recent common ancestors [N34 (−1) and N24 (−2) in Figures 1, **4**] being strychnine binders (possibly with less sensitivity) is substantial due to high conservation of the residues important for strychnine recognition. Among the 15 identified key residues in hT2R46, at least 14 were predicted to be unchanged in N34 (A268^7.42^ could be an exception, albeit the variation was predicted with lower confidence), and only two differ in chemical nature in the predicted N24, namely E70^2.65^V and E265^7.39^Q (Table 1; Figure 4). As discussed above, E70^2.65^V only decreased hT2R46's strychnine responsiveness by roughly 20% compared to the wild type. In contrast, mutagenesis in position 7.39 is more likely to alter responsiveness: E265^7.39^D maintained the same maximum activation with modest decrease of sensitivity, E265^7.39^Q led to further reduction in sensitivity and lower maximum activation level, and E265^7.39^K almost annihilated the response (Brockhoff et al., 2010). Therefore, the predicted ancestral sequences N34 and N24 are likely to have a modest sensitivity to strychnine. Notably, residue in position 7.42 was highly variable depending on the selections of IG and OG, as exemplified by Figure 5 (detailed illustration in Supplementary 2, tab “Summary”). In N34 (−1), the direct ancestor of hT2R46, it is predicted as Thr with a slightly higher confidence than Ala when using protein sequences with an identity above 55% compared to hT2R10 or hT2R46; the confidence of predicted Thr remarkably increased when the cutoff identity was lowered to 45% with 27 more reference sequences included as hT2R46 homologs. Variations were also predicted in this position in the next two most recent common ancestors N24 (−2) and N23 (−3) (Figure 5). This high variability in position 7.42 denotes the possibility of increased positive selection, as commonly found in T2Rs (Shi et al., 2003; Risso et al., 2017). In fact, position 7.42 was previously inferred as a positively selected site within the hT2R43-50 subtypes (Shi et al., 2003). The rest of the key residues were found to be highly resilient to the varied IG and OG samplings. Both A268^7.42^T and A268^7.42^R may cause steric hindrance and affect strychnine sensitivity, leaving the strychnine binding ability of these common ancestors an open question.

#### T2R10 vs. T2R46 clades

T2R10 is more conserved and species-general but has less homologous genes within the same clade, while T2R46 is highly species-specific with more homologs in different primates (a similar pattern was found also among the corresponding pseudogenes, not shown). Our results indicate that the strychnine-binding ability could have been developed as early as the common ancestors (N80-N82) of the T2R10 clade, prior to the speciation of primates and other mammals (i.e., felidae, canidae, and bears). In contrast, the earliest predicted strychnine binders in the T2R46 clade (N23 and N24) came into existence much more recently, possibly right before humans were separated from other primates. All the inferred common ancestors throughout the time elapsed from N80 (0.95) to N23 (1.87) (Figure 1) were not likely to bind strychnine, confirming the independent acquisition of binding ability, as suggested earlier (Born et al., 2013). Following N23 and N24, this function was lost during the rapid gene expansion but was preserved in a few *T2R* genes and with improved sensitivity in hT2R46 after the hominid divergence. Furthermore, despite the fact that common ancestors (−1) through (−4) in T2R10 clade and (−1), (−2) in T2R46 clade (with less confidence) were predicted to be strychnine binders, the common ancestor of the T2R10 and T2R46 clades, N2, is not predicted to bind strychnine due to the largely altered key functional residues predicted in the binding site (Table 1). Although 11 out of the 20 identified key residues (Table 1) were predicted to be conserved, the combination of the varying residues is not expected to confer strychnine-binding ability: considering the different modes of strychnine recognition (Born et al., 2013), partial conservation of the key residues from both hT2R subtypes is not conducive to function retention. Specifically, positions 3.32, 3.36, and 6.51 are the same in N2 as in T2R10 and T2R46 clades, but these positions are conserved in most of hT2Rs, suggesting a structural of functional role rather than ligand recognition and selectivity (Sandal et al., 2015; Di Pizio et al., 2016). Positions 7.39 and 7.42 are essential for strychnine recognition in hT2R46 (Brockhoff et al., 2010), but are hT2R10-like in N2; likewise, positions that were experimentally shown to be important for strychnine-hT2R10 binding, are different in N2.

### Position-specific positive selection

Position-specific analysis of the ratio between non-synonymous and synonymous mutation rates (dN and dS, respectively) in the binding sites was carried out next.

In a previous study using full sequences of T2R10 and T2R46 from six primates, the overall dN/dS ratio in T2R10 appeared to be lower than that in T2R46, although both were less than 1 (Fischer et al., 2005), indicating that T2R46 could have been under either lower evolutionary constraints or higher positive selection than T2R10. The dN/dS ratios for the key functional positions from hT2R10 and hT2R46 for strychnine recognition were carried out here and are summarized in Table 2; further details can be found in Supplementary 2, tabs “dN_dS-R10” and “dN_dS-R46.” Among the 12 key positions in hT2R10, one was highly conserved under purifying selection (dN/dS < 1) while none under positive selection, whereas four (including A268^7.42^) out of the 15 key positions in hT2R46 are under significant positive selection (dN/dS > 1) and one under purifying selection (Table 2). The total number of positive selected positions was 2 in hT2R10 vs. 15 in hT2R46, while the numbers of negatively selected positions within the two sequences are more comparable (Table 2).

**Table 2:** Predicted key residues under position-specific positive or negative selection.

	**Key positions under selection pressure**	**dN/dS estimated**	**CI**	**Total # of pos. selected positions**	**Total # of neg. selected positions**
hT2R10	N92^3.36^	0.26	[0.0058,0.55]	2	58
hT2R46	N161^ECL2^	0.44	[0.15,0.91]	15	86
	N92^3.36^, N176^5.39^, E253^6.63^, A268^7.42^	2.7	[2.7,2.7]		

Based on our modeling, N176 directly interact with strychnine via H-bond, whereas E253 forms an intramolecular H-bond with K258^ECL3^ in the absence of direct interaction with strychnine (Figure 2). However, experimental data reported higher tolerance to mutations in both positions (Brockhoff et al., 2010), which is consistent with the predicted positive selective pressure and consequently increased diversity (Table 2). The only negatively selected key site in hT2R46—N161—is involved in Asn-linked glycosylation and, is highly conserved across the 25 hT2Rs (Reichling et al., 2008) and is probably critical for a general function rather than specific agonist interaction. The role of N92, the key site in hT2R10 under negative selection, was yet to be elucidated. Interestingly, the same position N92^3.36^ in hT2R46 is predicted to be under positive selection.

Additionally, research has demonstrated the critical functions of extracellular loop (ECL) 2 in GPCRs (Wheatley et al., 2012; Woolley and Conner, 2016). Indeed, almost half of the positively selected sites in hT2R46 were located in ECL2, and one of the two positively selected positions in hT2R10 was also found in ECL2 (Supplementary 2, tabs “dN_dS-R10” and “dN_dS-R46”). This points to the significant role of ECL2 in ligand recognition, selectivity, and binding.

## Conclusion

In humans, the bitter taste receptors hT2R10 and hT2R46 are activated by strychnine. Here, phylogenetic analysis and estimation of relative divergence times were combined with previously obtained information about key binding site positions, to analyze when strychnine recognition was acquired by bitter taste receptors. Combining sequence-based and experimentally validated structure-based studies we suggest strychnine binding receptors for other species and for ancestral sequences. The findings suggest that hT2R10 and hT2R46 independently acquired the ability for strychnine recognition. Over the course of evolution, the sensitivity to strychnine was first developed in the earliest common ancestor of the T2R10 clade, and was preserved in most of the sequences within this clade but lost in a few species such as common marmoset, sheep, and rodents. As the descendants of N2 diverged into more T2R subtypes in different mammals, strychnine recognition was re-acquired possibly by the common ancestor of T2R43-47, which then underwent positive selection with accelerated gene duplication and increased gene diversity, resulting in highly similar clusters of T2Rs with diverse tuning spectra. The majority of homologs within the T2R46 clade lost the ability to detect strychnine, but this function was preserved in the T2Rs of a few primates and enhanced in the T2R46 subtype in certain primates, such as Neanderthals and modern humans. The low ratio of non-synonymous to synonymous mutation rates is indicative of high conservation of key amino acid sites in hT2R10-related T2Rs, while certain strychnine-binding key positions, especially 7.42, in hT2R46-related T2R subtypes, are susceptible to variation, suggesting a potential focal point for receptor adaptation to environmental fluctuations.

In this paper we provided an in-depth analysis of known and predicted T2Rs activated by strychnine. Owing to the low sequence identity between Class A GPCR templates and hT2R subtypes, it was suggested that advanced docking methods and molecular dynamics simulations, iteratively integrated with experimental tests, should be applied to overcome limitations in the structural predictions of ligand binding, as exemplified by Di Pizio et al. (2017) and Fierro et al. (2017). Therefore, undertaking further studies to investigate the effect of the variants discussed in this study would complement our understanding of bitter taste evolution and furnish a validation of our approach that may be applied to other biological systems.

## Materials and methods

### Homologs identification

The human T2R10 and T2R46 sequences (UniProtKBs: Q9NYW0 and P59540, respectively) were used as queries to retrieve similar T2R sequences from different species via NCBI PSI-BLAST. Two iterations were run for each query against the non-redundant protein sequences database (retrieved during January–June, 2017). Given the questionable consistency of protein nomenclature across orthologs and variation in the total number of bitter taste receptors between species, all sequences with 55% and above amino acid sequence identity (E-values after the 1st iteration: 6e-88 for hT2R10 and 5e-84 for hT2R46) to the query sequences were initially retrieved; fragmental sequences with 265 residues or less were removed to avoid excessive gap-opening in ancestral sequence reconstruction. This resulted in a total of 87 reference sequences (RS) (17 sequences for hT2R10 and 70 sequences for hT2R46, available in FASTA format in Supplementary 1).

The T2R10 clade was then supplemented with the following nine T2R10s obtained via PCR from a recently published study: wolf (Clu), maned wolf (Cbr), African hunting dog (Lpi), cheetah (Aju), leopard, Tibetan fox (Vfe), Corsac fox (Vco), red fox (Vvu), and Fennec fox (Vze) (Shang et al., 2017; Supplementary 1). All of these nine T2Rs have over 74% protein sequence identity with the hT2R10. From the same study, five T2R43 sequences showed 60% full protein sequence identity with hT2R46. However, due to their distant relationship with hT2R46 in the phylogenetic tree (Supplementary 1) with the majority of the key residues for strychnine recognition altered, these sequences were not added to the final hT2R46 clade.

For the T2R46 clade, the initial phylogeny (details in the next section) constructed with the sequences obtained through PSI-BLAST showed only four sequences within the subgroup of hT2R46 among the 70 sequences from more than 10 primates that were sampled. Although diversification could result in lower conservation, this could also be attributed to the deactivation of T2R46 in certain primates under lineage-specific evolutionary constraints (Go et al., 2005; Risso et al., 2017). We searched via BLASTn for pseudogene nucleotide sequences most similar (over 80% identity) to the hT2R46 gene. We also looked for human T2R pseudogenes (hT2R12p, 15p, 18p, 63p, 64p, and 67p) that are located on the same chromosome as hT2R10 and hT2R46 (*12p13.2*) in the HGNC database (retrieved in April, 2017: http://www.genenames.org/cgi-bin/genefamilies/set/1162), and added them to the T2R46 clade. Eleven pseudogenes were added after functional restoration and translation into protein sequences (Supplementary 1, “Restored Pseudogenes” & Supplementary 3 “Pseudogene restoration”) (Martin et al., 2009). The resulting tree (Supplementary 3; see details concerning tree construction below) showed that these pseudogenes were not close homologs of hT2R46 *per se* but of other hT2Rs which have high sequence identity with hT2R46, such as hT2R48 and hT2R49. The results presented in the main text are highly similar to those obtained after addition of pseudogenes except for a few variations in the predicted common ancestors with varied confidence level (e.g., A268^7.42^ as discussed before) (phylogenetic tree with predicted ancestral sequences using the 11 pseudogenes are listed in Supplementary 1).

Lastly, a set of pre-aligned human T2Rs were supplemented to the data set described above. The alignment was manually adjusted based on existing data from mutagenesis (Brockhoff et al., 2010; Meyerhof et al., 2010; Born et al., 2013), and the Ballesteros–Weinstein numbers were assigned accordingly (detailed in “Key positions analysis”). We began by using all 25 hT2Rs, then reduced to 9 hT2Rs including the reference sequences (hT2R43-50 and hT2R10), leaving only those that are at least 70% identical with either hT2R10 or hT2R46, as the sequence identities of the remaining hT2Rs are lower than 45% (Supplementary 1, “Aligned hT2Rs”).

In total, 105 hT2R10- or hT2R46-related reference sequences were retrieved, of which 27 were hT2R10-related and 78 hT2R46-related. The T2R10 and T2R46 nucleotide sequences from the Neanderthal genome database (http://neandertal.ensemblgenomes.org/index.html) were also retrieved but not used for phylogenetic tree construction due to over 99.99% identity with hT2R10 and hT2R46, respectively.

Different outgroups (OGs) were sampled to assess their effects on the tree topology and predicted ancestral sequences. As a significant component of phylogenetic analysis, OG selection can influence branch order and length, clade monophyly, as well as divergence rates (Puslednik and Serb, 2008). Increased OG sampling has been shown to improve the stability of tree topology (Nixon and Carpenter, 1993). The V1R proteins (member of the type 1 vomeronasal pheromone receptor gene family) commonly serve as OGs in previous T2Rs related studies (Meyerhof and Korsching, 2009; Li and Zhang, 2014). We selected the V1Rs from apes (Ptr, Mmu), mouse (*Mus musculus*, Mmus), rat (*Rattus norvegicus*, Rno), frog (Xtr), alligator (*Alligator mississipiensis*, Ami), and fish (*Danio rerio*, Dre) as well as the T2Rs from frog, alligator, and fish, which are no more similar to the query sequences than the V1Rs (see Supplementary 3, “Outgroup identity with query sequences”). The resulting ingroup monophyly is fairly consistent across different outgroup samples. However, variations abound on the protein sequence level among the inferred ancestors with different IG and OG samplings (Supplementary 2, tab “Summary”).

### Phylogeny and ancestral sequence reconstruction

The 116 protein sequences obtained from the previous steps were first aligned by MAFFT server (Katoh et al., 2002), followed by a quality assessment of the resulting MSA using the GUIDANCE2 server (Sela et al., 2015). The final alignment was calibrated to preserve the consistency of pre-assigned BW numbers from previous studies (Brockhoff et al., 2010; Meyerhof et al., 2010; Born et al., 2013). The evolutionary tree was constructed using Cipres' RaxML (Randomized Axelerated Maximum Likelihood) version 8, HPC BlackBox (Stamatakis, 2014) (see Supplementary 1 for MSA and phylogeny). We then inferred what ancestral sequences these receptors might have had using the FastML server (Ashkenazy et al., 2012). All the parameters used in these steps were set to default.

### Divergence times estimation

To add a temporal dimension to the reconstructed phylogenetic tree and thus infer the precedence of certain T2Rs to others, the relative times of divergence for the branching points were estimated using a molecular dating method named RelTime (Tamura et al., 2012) with the JTT matrix-based model (Jones et al., 1992) as implemented in MEGA 7 (Kumar et al., 2016). All the parameters were kept as default.

### Key positions analysis

The key positions are indicated using the BW. Amino acids in those key positions within the hT2R10- or hT2R46-like sequences and their reconstructed ancestral sequences were compared to the corresponding key residues from hT2R10 and hT2R46 (Table 1).

### Structural analysis

Homology models of hT2R10 and hT2R46 were constructed using Prime (version 4.8, Schrödinger, LLC, New York, NY, 2017), the sequence alignment previously generated (Di Pizio et al., 2016; Karaman et al., 2016) and the structures of β2 adrenergic receptor in its active state (PDB ID: 3SN6) (Rasmussen et al., 2011) and human kappa opioid receptor (PDB ID: 4DJH) (Wu et al., 2012) as templates, as described in Di Pizio et al. (2017). Hydrogen atoms and side chain orientations of the receptor were optimized at physiological pH with the Protein Preparation Wizard tool in Maestro (version 11.2, Schrödinger, LLC, New York, NY, 2017).

The 3D conformation and protonation state at pH 7 ± 0.5 of strychnine was generated with LigPrep (version 4.2, Schrödinger, LLC, New York, NY, 2017). Strychnine is predicted to be protonated at this pH. The Schrödinger Induced-Fit docking protocol (Glide version 7.5; Prime version 4.8, Schrödinger, LLC, New York, NY, 2017) was used to predict the binding modes of the strychnine to the hT2R10 and hT2R46 receptor models. Glide Standard Precision (SP) was used as scoring function.

### Site-specific selective pressure

Functional DNA sequences were retrieved first via BLASTn from NCBI nucleotide collection database under default settings (Supplementary 1). The selected 56 sequences (24 hT2R10-like and 28 hT2R46-like) had at least 80% identity with hT2R10 or hT2R46 and length of at least 750 nucleotides. Underlying selective forces in the key positions responsible for strychnine recognition were identified by the site-specific ratios of dN vs. dS via Selecton Server (Stern et al., 2007). Default settings were applied except for changing the precision level from “medium” to “high.”

## Author contributions

AYX, AL, and MYN Designed research; AYX, ADP, and AL Performed research; AYX, MYN, ADP, OP, and TP Analyzed the data; AYX, ADP, and AL Wrote the paper. All the authors read and edited the paper.

### Conflict of interest statement

The authors declare that the research was conducted in the absence of any commercial or financial relationships that could be construed as a potential conflict of interest.

## Footnotes

^1^USDA PLANTS database. “*Strychnos nux-vomica”. Natural Resources Conservation Service PLANTS Database*. USDA. (Accessed June 1, 2017).
