# Corrigendum: Independent Evolution of Strychnine Recognition by Bitter Taste Receptor Subtypes

**DOI:** 10.3389/fmolb.2018.00084

**Published:** 2018-09-11

**Authors:** Ava Yuan Xue, Antonella Di Pizio, Anat Levit, Tali Yarnitzky, Osnat Penn, Tal Pupko, Masha Y. Niv

**Affiliations:** ^1^Robert H. Smith Faculty of Agriculture, Food and Environment, Institute of Biochemistry, Food Science and Nutrition, The Hebrew University of Jerusalem, Rehovot, Israel; ^2^The Fritz Haber Center for Molecular Dynamics, The Hebrew University of Jerusalem, Jerusalem, Israel; ^3^Department of Pharmaceutical Chemistry, University of California, San Francisco, San Francisco, CA, United States; ^4^Tali Yarnitzky Scientific Consulting, Maccabim-Reut, Israel; ^5^Modeling, Analysis and Theory Group, Allen Institute for Brain Science, Seattle, WA, United States; ^6^The Department of Cell Research and Immunology, George S. Wise Faculty of Life Sciences, Tel Aviv University, Tel Aviv, Israel

**Keywords:** bitter taste receptor, homology modeling, ligand recognition, functional residues, evolution, ancestor functionality, ancestral reconstruction, phylogenetics

In the original article, **the baboon image** was inserted without the attribution to the author, Dr. Kenneth Chiou. The attribution of the image has now been inserted in **Figure 3** and **Figure 4**, and reads:

Baboon image created by © Kenneth Chiou, in “Population genomics of a baboon hybrid zone in Zambia”, http://kennychiou.com/dissertation/#species.

The authors apologize for the omission and state that the current version does not change the scientific conclusions of the article in any way.

The original article has been updated.

## Conflict of interest statement

The authors declare that the research was conducted in the absence of any commercial or financial relationships that could be construed as a potential conflict of interest.

