# Supplementary material for: Independent Evolution of Strychnine Recognition by Bitter Taste Receptor Subtypes
Source: Front Mol Biosci. 2018 Mar 2;5:9. doi: 10.3389/fmolb.2018.00009 (PMC5840161; doi:10.3389/fmolb.2018.00009)
Supplement: Supplementary file 1 [file DataSheet1.pdf]

Supplementary 1

Independent Evolution of Strychnine Recognition by Bitter Taste Receptor Subtypes

Ava Yuan Xue, Antonella Di Pizio, Anat Levit, Tali Yarnitzky, Osnat Penn, Tal Pupko and Masha Y. Niv

Binomial nomenclature & common names used in the supplementaries

Aju AcinonyxJubatus
Ame Panda
Ami alligator
Ase Rhowler
Bmu Dyak
Bta Cattle
Cbr ManedWolf
Cja CMarmos
Clu Wolf
Clu.fa Canis
Dre Zebrafish
Ggo Gorilla
Hag Agibbon
Lpi HungtingDOg
Mbr Bbat
Mda Dbat
Mfa CEmacaque
Mmu Rmacaque
Mmus Mouse/mus
Nle Ngibbon
Oar sheep
Pha Hbaboon
Ppa Bonobo
Ppard PantheraPardus
Ppy BOrangutan
Ptr Chimp
Rno Rat
Sga Gmolerat
Ssc pig
Tcr Slutung
Vco CorsacFox
Vfe TibetanFox
Vvu RedFox
Vze FennecFox
Xtr frog

MAFFT MSA Alignments of the 105 IG and 10 OG sequences

```
>hT2R10
-----------------------M-------------LRVVEGIFIFVVVSESVFGVLGN
GFIGLVNCIDCAKN--KLSTIGFILTGLAISRIFLIWIIITDGFIQIFSPNIYASGNLIE
YISYFWVIGNQSSMWFATSLSIFYFLKIANFSNYIFLWLKSRTNMV-LPFMIVFLLISSL
LNFAYIAKILNDYKTK-------N-DTVWDLNMYKS------EYFIKQILLNLGVIFFFT
LSLITCIFLIISLWRHNRQMQSNVTGLRDSNTEAHVKAMKVLISFIILFILYF-IGMAIE
ISCF-----TVREN--KLLLMFGMTTTAIYPWGHSFILILGNSKLKQASLRVLQQLKCCE
KRKNL--------------------RVT------------------------
>R10chimp
-----------------------M-------------LRVVEGIFIFVVISESVFGVLGN
GFIGLVNCIDCAKN--KLSTIGFILTGLAISRIFLIWIIITDGFIQIFSPNIYASSNLIE
YISYFWVIGNQSSMWFATSLSIFYFLKIANFSNYIFLWLKSRTNMV-LPFMIVFLLISSL
LNFAYIAKILNDYKMK-------N-DTVWDLNMYKS------EYFIKQILLNLGVIFFFT
LSLITCVLLIISLWRHNRQMQSNVTGLRDSNTEAHVKAMKVLISFIILFILYF-IGMAIE
ISYF-----TVREN--KLLLMFGMTTTAIYPWGHSFILILGNSKLKQASLRVLQQLKCCE
KRKNL--------------------RVT------------------------
>R10gorilla
-----------------------M-------------LRVVEGIFIFVVISEXVFGVLGN
GFIGLVNCIDCAKN--KLSTIGFILTGLAISRIFLIWIIITDGFIQIFSPDIYASGNLIE
YISYFWVIGNQSSMWFATSLSIFYFLKIANFSNYIFLWLKSRTNMV-LPFMIVFLLISSL
```

```
LNFAHIAKILNDYKMK-------N-DTVWDLNMYKS------EYFIKQILLNLGVIFFFT
LSLITCVFLIISLWRHNRQMQSNVTGLRDSNTEAHVKAMKVLISFXILFILYF-IGMAIE
ISCF-----TVREN--KLLLMFGMTTTAIYPWGHSFILILGNSKLKQASLRVLQQLKCCE
KRKNL--------------------RVT-----------------------
>R10NGibbon
----------------------M-------------LSVVEGIFIFVVITESVFGVLGN
GFIGLVNCIDCAKN--KLSTIGFILTGLAISRIFLIWIIITDGFIKIFSPDIYASGNLIE
YISYFWVISNQSSMWFATSLGIFYFLKIANFSNYIFLWLKSRTNMV-LPFMIVFLLISLL
LNFAHIAKILNDHKMK-------N-DTVWHLNLYKS------EYFIKQVLLNLGVIFFFT
LSLITCVLLIISLWRHNRQMQSNVTGLRDSNTEAHVKAMKVLVAFIILFILYF-IGMAIE
ISYF-----TVPEN--KLLLMFGMTTTAIYPWGHSFILILGNSKLKQASLRVLQQLKCCE
KRKNL--------------------RVT-----------------------
>R10BOrang
----------------------M-------------LSVVEGIFIFVVISESVFGVLGN
GFIGLVNCIDCAKN--KLSTIGFILTGLAISRIFLIWVIITDGFIQIFSPDIYASGNLIE
YISYIWVIGNQSSMWFATSLSIFYFLKIANFSNYIFLWLKSRTNMV-LPFMMAFLLISSL
LNFAHIVKILNDHKMK-------N-DTVWHLNMYKS------EYFIKQILLNLGVIFFFT
LSLITCVLLIISLWRHNRQMQSNVTGLRDSNTEAHVKAMKVLISFIILFILYF-IGMALE
ISRF-----TVPEN--KLLLMFGMTTTAIYPWGHSFILILGNSKLKQASLRVLQQLKCCE
KRKKS--------------------QSH----------------------I
>R10Rmacaq
----------------------M-------------LSVVEGIFIFVVISESVFAVLGN
GFIGLVNCIDCAKN--KLSTIGFILTGLAISRIFLIWLIITDGFIRIFSPDIYASGNLIE
YISYFWVISNQSSMWFATSLSIFYFLKIANFSNYIFLWLKSRTNRV-LPLLMAFLLISCL
LNFAYIAKILNDFKMK-------N-DTVWRLNIFKN------EYFVKQILLNLGVIFFFT
LSLITSVLLIISLWRHNRQMQSNVTGLRDSNTEAHVKAMKVLISFIILFILYF-IGIAIE
ISYF-----TVPEN--KLLLMFGMTTTAIYPWGHSFILILGNSKLKQASLRVLQQLKCCE
KRKNL--------------------RAT-----------------------
>R10HBaboon
----------------------M-------------LSVVEGILILVVISESVFGVLGN
GFIGLVNCIDCAKN--KLSTIGFILTGLAISRIFLIWIIITDGFIQIFSPDVYASGNLIE
YISYFWVITNQSSIWFATSLSIFYFLKIANFSNYIFLWLKSRINRV-LPLLMGFLLISCL
LNFAYIVKILNDLKMK-------N-DTVWRLNMYKS------EYFIKQLLLNLGVIFFFT
LSLITSVLLIISLWRHNRQMQSNVTGLRDSITEAHVKAMKVLISFIILFILYF-IGIAIE
ISYF-----TVPEN--KLLLIFGMTTTAIYPWGHSFILILGNSKLKQASLRVLQQLKCCE
ERKNL--------------------RAT-----------------------
>R10CMarmos
----------------------M-------------LSVVDGIFIFVVISESVLGVLGN
GFIGLVNCINCVKH--KFSLIGFILTGLAISRICLIWLLITDGFMHIFSPDIYFSSNLIE
CISYFWLIINQSSIWFATCLSIFYFLKIANFSNTIFLWLKSRINRI-PAFLLGCLLISWL
FSFPHTVKMFKDHKLK-------N-ATDWHFNVSKS------EYFIKQVLLNLGVIFSFT
LSLTTCVLLIISLWRHNRQMQLNLTGFRDVNTEAHVKAMKVLISFIILSILYF-VGIIIE
ITFF-----SVPEH--KLLLMFGITTSVLYPWSHSFILILGNSKLKQASLRALQQLKCCK
KQKRL--------------------RAT-----------------------
>pigT2R10
----------------------M-------------LSIVESLLIFISVSQSILGFLGN
GFIGLVTCIDCVKN-KNISMISFILTGLATSRICLIWLIIIDGFIKIFFPDLYISGKLTE
YISYSWVIVNHSSIWFATSLSIFYFLKIANFSHHIFLWLKGKINRV-LLILMGYLFISWL
FTFPQVVKIISDSKKK-------NGSSFWPLNMHKL------EYFMSQFLLNLGVILLFI
LCMITCFLLIISLWRHNRQMQSNATGFRDPSTEAHIKAMKIVISFIILFILYF-IGVAIE
ISCG-----TQPEN--KLLFIFGMITTAIFPWGHSFILILGNKKLKQASLKVLKQLKCWG
KEKLL--------------------RTP-----------------------
>PantheraPardusR10
----------------------M-------------LSIVEGLLIFIAVSESVLGVLGN
GFIGLVNCMDCVKN-KKFSMIGFILTGLATSRICLILIVIVDGFIKIFSPDMYSSGHLID
YISYLWIIINQSNIWFATSLSTFYFLKIANFSHHMFLWLKGRINWV-LPLLMGSLFISWL
FTFPQIVKILRDSKVV-------NGNSTWQLNMLKS------EFFTKQILVNVGVLLLFT
LFLITCFLLIISLWRHSRRMQLNVTGFQDPSTEAHMKAMKVLISFLILFILHF-IGLAIE
IACF-----TMPEK--KLLFIFGMMTTVLYPWGHSFILILGNSKLKQASLRALQQVKCY-
---------------------------------------------------
>AcinonyxJubatusR10
----------------------M-------------LSIVEGLLIFIAVSESVLGVLGN
GFIGLVNCMDCVKN-KKFSMFGFILTGLATSRICLILIVIADGFIKIFSPDMYSSGHLID
YISYLWIIINQSNIWFATSLSTFYFLKIANFSHHMFLWLKGRINWV-LPLLMGSLFISWL
FTFPQIVKILSDSKVG-------NGNTTWQLNMPKS------EFFTKQILVNVGVLLLFT
LFLITCFLLIISLWRHSRRMQLNVTGFQDPSTEAHMKAMKVLISFIILFILHF-IGLAIE
```

```
IACF-----TMREK--KLLFIFGMMTTVLYPWGHSFILILGNSKLKQASLRALQQVKCC-
----------------------------------------------------
>CorsacFoxR10
----------------------M-------------LSILEVLLIFIAVSESILGVLGN
GFIGLVNCIDCVKN-KKFSMVGFILTGLATSRICLILIIITDGFIKIFSPDMYSSGNLID
YISYLWVIINQSSIWFATSLSIFYFLKIANFSHHIFLWLKGRINSV-LPLLMGSLFISWL
FTFPQIVKIINDNRMK-------SRNTTWQLNMQKS------EFFTKQILLNLGVILLFT
LCLITCFLLIISLWRHNRHMQLNVTGLRDPSTEAHVKAMKILVSFIILFILYF-IGIAIE
ISCF-----ILPEN--KLLFIFGMMTTAIYPWGHSFILILGNSKLKQASLKTLQQLKCCE
ARRLL--------------------TAAQIHVGGNG----------CSRRII
>ManedWolfR10
----------------------M-------------LSILEGLLIFIAVSESILGVLGN
GFIGLVNCIDCVKN-KKFSMVGFILTGLATSRICLILIIITDGFIKIFSPDMYSSGNLID
YISYLWVIINQSSIWFATSLSIFYFLKIANFSHHIFLWLKGRINSV-LPLLMGSLFISWL
FTFPQIVKIINDNRMK-------SRNTTWQLNMQKS------EFFTKQILLNLGVILLFT
LCLITCFLLIVSLWRHNRHMQLNVTGLRDPSTEAHVKAMKILVSFIILFILYF-IGIAIE
ISCF-----ILPEN--KLLFIFGMMTTAIYPWGHSFILILGNSKLKQASLKTLQQLKCCE
ARRLL--------------------TAAQIHVGGNG----------CSRRII
>TibetanFoxR10
----------------------M-------------LSILEGLLIFIAVSESILGVLGN
GFIGLVNCIDCVKN-KKFSMVGFILTGLATSRICLILIIITDGFIKIFSPDMYSSGNLID
YISYLWVIINQSSIWFATSLSIFYFLKIANFSHHIFLWLKGRINSV-LPLLMGSLFISWL
FTFPQIVKIINDNRMK-------SRNTTWQLNMQKS------EFFTKQILLNLGVILLFT
LCLITCFLLIISLWRHNRHMQLNVTGLRDPSTEAHVKAMKILVSFIILFILYF-IGIAIE
ISCF-----ILPEN--KLLFIFGMMTTAIYPWGHSFILILGNSKLKQASLKTLQQLKCCE
ARRLL--------------------TAAQIHVGGNG----------CSRRII
>WolfR10
----------------------M-------------LSILEGLLIFIAVSESILGSLGN
GFIGLVNCIDCVKN-KKFSMVGFILTGLATSRICLILIIITDGFIKIFSPDMYSSGNLID
YISYLWVIINQSSIWFATSLSIFYFLKIANFSHHIFLWLKGRINSV-LPLLMGSLFISWL
FTFPQIVKIINDNRMK-------SRNTTWQLNMQKS------EFFTKQILLNLGVILLFT
LCLITCFLLIVSLWRHNRHMQLNVTGLRDPSTEAHVKAMKILVSFIILFILYF-IGIAIE
ISCF-----ILPEN--KLLFIFGMMTTAIYPWGHSFILILGNSKLKQASLKTLQQLKCCE
ARRLL--------------------TAAQIHVGGNG----------CSRRII
>HuntingDogR10
----------------------M-------------LSILEGLLIFIAVSESILGVLGN
GFIGLVNCIDCVKN-KKFSMVGFILTGLATSRICLILIIITDGFIKIFSPDMYSSGNLID
YISYLWVIINQSSIWFATSLSIFYFLKIANFSHHIFLWLKGRINSV-LPLLMGSLFISWL
FTFPQIVKIINDNRMK-------SRNTTWQLNMQKS------EFFTKQILLNLGVILLFT
LCLITCFLLIVSLWRHNRHMQLNVTGLRDPSTEAHVKAMKILVSFIILFILYF-IGIAIE
ISCF-----ILPEN--KLLFIFGMMTTAIYPWGHSFILILGNSKLKQASLKTLQQLK-CE
ARRLL--------------------TAAQIHVGGNG----------CSRRII
>R10Canis
----------------------M-------------LSILEGLLIFIAVSESILGVLGN
GFIGLVNCIDCVKN-KKFSMVGFILTGLATSRICLILIIITDGFIKIFSPDMYSSGNLID
YISYLWVIINQSSIWFATSLSIFYFLKIANFSHHIFLWLKGRINSV-LPLLMGSLFISWL
FTFPQIVKIINDNRMK-------SRNTTWQLNMQKS------EFFTKQILLNLGVILLFT
LCLITCFLLIVSLWRHNRHMQLNVTGLRDPSTEAHVKAMKILVSFIILFILYF-IGIAIE
ISCF-----ILPEN--KLLFIFGMMTTAIYPWGHSFILILGNSKLKQASLKTLQQLK-CE
ARRLL--------------------TAAQIHVGGNG----------CSRRII
>RedFoxR10
----------------------M-------------LSILKSPLIFIAVSESILGVLGN
GFIGLVNCIDCVKN-KKFSMVGFILTGLATSRICLILIIITDGFIKIFSPDMYSSGNLID
YISYLWVIINQSSIWFATSLSIFYFLKIANFSHHIFLWLKGRINSV-LPLLMGSLFISWL
FTFPQIVKIINDNRMK-------SRNTTWQLNMQKS------EFFTKQILLNLGVILLFT
LCLITCFLLIISLWRHNRHMQLNVTGLRDPSTEAHVKAMKILVSFIILFILYF-IGIAIE
ISCF-----ILPEN--KLLFIFGMMTTAIYPWGHSFILILGNSKLKQASLKTLQQLKCCE
ARRLL--------------------TAAQIHVGGNG----------CSRRII
>FennecFoxR10
----------------------M-------------LSILERLSFFIAVSESILGVLGN
GFIGLVNCIDCVKN-KKISMVGFILTGLATSRICLILIIITDGFIKIFSPDMYSSGNLID
YISYLWVIINQSSIWFATSLSIFYFLKIANFSHHIFLWLKGRINSV-LPLLMGSLFISWL
FTFPQIVKIINDNRMK-------SRNTTWQLNMQKS------EFFTKQILLNLGVILLFT
LCLITCFLLIISLWRHNRHMQLNVTGLRDPSTEAHVKAMKILVSFIILFILYF-TGIAIE
ISCF-----ILPEN--KLLFIFGMMTTAIYPWGHSFILILGNSKLKQASLKTLQQLKCCE
ARRLL--------------------TAAQIHVGGNG----------CSRRII
```

```
>R10panda
-----------------------M-------------LSILEGLFIFIAVSESILGVLGN
GFIGLVNCIDCVKN-KKFSMIGFILTGLATSRICLILIIITDGLIKIFFPDMYSSGNLID
YISYLWVIFNQSSIWFATSLSVFYFLKIANFSHHIFLWLKGRINRV-LHLLMGSLFISWL
FTFPQIVEIINESRMK-------SGNATWNLHMQRS------KFFTKQILLNLGVILLFT
LCLITCFLLIISLWRHNRHMQLNVTGPRDPSTEAHVKAMKVLISFIILFILYF-IGIAIE
ISCF-----TLPEN--KLLFIFGMVTTVIYPWGHSFILILGNSKLKQASLRALQRFKRCE
VGRLL--------------------TAAQTRVRSNG----------CSRRMI
>R10cattle
-----------------------M-------------LSIVEGLLLFVAVNESVLGVLGN
GFIGLVNCINCVKN-KKISTLSLILTGLASSRFCLIWIITTDAYVRVFSPDMYLSGNLSQ
YIAYLWIIMNQSSVWFTTSLSIFYFLKIANFSHCIFLWLKGHITEI-LLLLMGCLPISWL
FTFPNITMPFINNIMK-------NRSTTGLVTMQKS------EYFINQILFNLGTFLVFV
LCLITCFLIITSLWRHNRRMQLNATGFRDPSTEAHIKAMKILVSFIILFILYF-VGTAIQ
ILSV-----TVPEN--KLLFIFGMTTTILYPCGHSFILILGNSKLNQASLRVLKLLKC--
----------------------------------------------------
>R10sheep
-----------------------M-------------LSTVEGLLIYVAVSESVLGVLGN
GFIGVVSCIDCVKS-KKIPTVSLILTGLASSRFCLIWIIITDAYVRMFFPDTYLSGNLSQ
NIAHFWIIMNQSSIWFATSLNIFYFLKIANYSHCIFLWLKGHINRV-LLLFMGSLLISWL
FAFPSIAKPSINDIMK-------NRSSTWLIALHKR------EYLTNHILLNIGVILVFV
LCLITCFLLITSLWRHNRKMQLNATGFRDPSTEAHIKAMKTLVSFIILFILYF-VGTAIQ
ISGS-----TMPEN--NLLLIIGITTRLLYPCGHSLILILGNRKLKQDFLRVLKPLKCWG
KEKLL--------------------RIP-----------------------
>R107Mus
-----------------------M-------------LNSAEGILLCVVTSEAVLGVLGD
TYIALFNCMDYAKN-KKLSKIGFILIGLAISRIGVVWIIILQGYIQVFFPHMLTSGNITE
YITYIWVFLNHLSVWFVTNLNILYFLKIANFSNSVFLWLKRRVNAV-FIFLSGCLLTSWL
LCFPQMTKILQNSKMH-------QRNTSW-VHQRKN------YFLINQSVTNLGIFFFII
VSLITCFLLIVFLWRHVRQMHSDVSGFRDHSTKVHVKAMKFLISFMVFFILHF-VGLSIE
VLCF-----ILPQN--KLLFITGLTATCLYPCGHSIIVILGNKQLKQASLKALQQLKCCE
TKGNF--------------------RVK-----------------------
>R107Rat
-----------------------M-------------LSAAEGILLCVVTSEAVLGVLGD
TFIALANCMEYAKN-KKLSKIGFILIGLAISRIGVVWIIILQGYMQVFFPHILTFGNITE
YITYIWVFLNHLSVWFATNLNILYFLKIANFSNSVFLWLKSRVRVV-FIFLSGCLLTSWL
LCFPQFSKMLNNSKMY-------WGNTSW-LQQQKN------VFLINQSLTNLGIFFFII
VSLITCFLLIVFLWRHIRQMHSDGSGLRDLNTEAHVKAMRVLISFAVLFILHF-VGLSIQ
VLCF-----FLPQN--NLLFITGLIATCLYPCGHSIILILGNKQLKQASLKALQHLTCCE
TKRNL--------------------SVT-----------------------
>T7Gmolerat
-----------------------M-------------LSAAESILLCVVTTESVLGVLGN
GFIGLVNCIDWIRS-KKFSKINFILTGLVISRIFLIWILICKAYVKTLSSQPLDYGNLVE
FMNYIWVIVNHMNIWFATSLSIFYFFKIANFSNYIFLWLKRKIDVI-FILLIGCLFASWL
IAVSQIAKIINNAKAQ-------YRNTSWQVQLWKS------VFFINHASVNIGAILFFT
VALITCFLLIISLWRHKRRMESAFSGLRDLNSEAHVKAIKVLISFLILLVLYF-TGISLD
TFYA-----FMPAS--KLLFLSGLTTASLYPCCHSFVLILADRKLKQVSLRILQQLKCHE
EE-DL--------------------RAV-----------------------
>R106Mus
-----------------------M-------------LTVAEGILLCFVTSGSVLGVLGN
GFILHANYINCVR--KKFSTAGFILTGLAICRIFVICIIISDGYLKLFSPHMVASDAHII
VISYIWVIINHTSIWFATSLNLFYLLKIANFSHYIFFCLKRRINTV-FIFLLGCLFISWS
IAFPQTVKIFNV-KKQ-------HRNVSWQVYLYKN------EFIVSHILLNLGVIFFFM
VAIITCFLLIISLWKHNRKMQLYASRFKSLNTEVHVKVMKVLISFIILLILHF-IGILIE
TLSF-----LKYEN--KLLLILGLIISCMYPCCHSFILILANSQLKQASLKALKQLKCHK
KDKDV--------------------RVTW----------------------
>R106Rat
-----------------------M-------------LTIPEGILLCFITSGSVLGVLGN
GFILHVNCTDCVR--QKFSTTGFIFTGLAISRICVICIIISDGYLKLFSPHMVASDAHII
GISYLWIITNHTSTCFATILNLFYFLKIANFSHYIFFCLKRKLNTI-FIFLLGCLFISWS
VAFPQTVKIFND-KMK-------HRNTSWKFHLHKS------KFIINHILLNLGVIFFCM
VAIITSFLLIISLWKHNRKMQLYVSRFKSLNTEVHLKVMKVLISFIILLILHV-IGILIE
TLSF-----LRYEN--KLLLILGLNFSSMYPCCHSFILILANNQLKQASLKALKQFKCHK
KDKDV--------------------RET-----------------------
>hT2R43
-----------------------M-------------ITFLPIIFSSLVVVTFVIGNFAN
```

```
GFIALVNSIESFKR-QKISFADQILTALAVSRVGLLWVLLLNWYSTVLNPAFN-SVEVRT
TAYNIWAVINHFSNWLATTLSIFYLLKIANFSNFIFLHLKRRVKSVILVMLLGPL----L
FLACHLFVINMNEIVR---TKEFEGNMTWKIKLKSA------MYFSNMTVTMVANLVPFT
LTLLSFMLLICSLCKHLKKMQLRGKGSQDPSTKVHIKALQTVISFLLLCAIYF-LSIMIS
VWSF-----GSLEN--KPVFMFCKAIRFSYPSIHPFILIWGNKKLKQTFLSVFWQMRYWV
KGEKT--------------------SSP------------------------
>R43chimp
-----------------------M-------------ITFLPIIFSSLVVVTFVIGNFAN
GFIALVNSIEWFKR-QKISFADQILTALAVSRVGLLWVLLLNWYSTVLNPAFN-SVEVRT
TAYNIWAVINHFSNWLATSLSIFYLLKIANFSNFIFLHLKRRVKSVILVMLLGPL----L
FLACHLFVINMNEIVR---TKEFEGNMTWKIKLKSA------MYFSNMTVTMVANLVPFT
LTLLSFLLLICSLCKHLKKMQLHGKGSQDPSTKVHINALQTVISFLLLCAIYF-LSIMIS
VWSF-----GSLEN--KPVFMFCKAIRFSYPSIHPFILIWGNKKLKQTFLSVFWQMRYWV
KGEKT--------------------SSP------------------------
>R43bonobo
-----------------------M-------------ITFLPIIFSSLVVVTFVIGNFAN
GFIALVNSIEWFKR-QKISFADQILTALAVSRVGLLWVLLLNWYLTVLNPAFN-SVEVRT
TAYNIWAVINHFSNWLATSLSIFYLLKIANFSNFIFLHLKRRVKSVILVMLLGPL----L
FLACHLFMINMNEIVR---TKEFDGNMTWKIKLKSA------MYFSNMTVTMVANLVPFT
LTLLSFLLLICSLCKHLKKMQLHGKGSQDPSTKVHIKALQTVISFLLLCAIYF-LSIMIS
VWSF-----GSLEN--KPVFMFCKAIRFSYPSIHPFILIWGNKKLKQTFLSVFWQMRYWV
KGEKT--------------------SSP------------------------
>hT2R44
-----------------------M-------------TTFIPIIFSSVVVVLFVIGNFAN
GFIALVNSIERVKR-QKISFADQILTALAVSRVGLLWVLLLNWYSTVFNPAFY-SVEVRT
TAYNVWAVTGHFSNWLATSLSIFYLLKIANFSNLIFLHLKRRVKSVILVMLLGPL----L
FLACQLFVINMKEIVR---TKEYEGNMTWKIKLRSA------VYLSDATVTTLGNLVPFT
LTLLCFLLLICSLCKHLKKMQLHGKGSQDPSTKVHIKALQTVIFFLLLCAVYF-LSIMIS
VWSF-----GSLEN--KPVFMFCKAIRFSYPSIHPFILIWGNKKLKQTFLSVLRQVRYWV
KGEKP--------------------SSP------------------------
>R44chimp
-----------------------M-------------TTFIPIIFSSLVVVTFVIGNFAN
GFIALVNSIEWFKR-QKISFADQILTALAVSRVGLLWVLLLNWYSTVLNPAFY-SVEVRT
TAYNVWAVTGHFSNWLATSLSIFYLLKIANFSNLIFLHLKRRVKSVILVMLLGPL----L
FLACQLFMINMKEIVR---TKEYEGNMTWKIKLRSA------VYLSDATVTTLGNLVPFT
LTLLCFLLLICSLCKHLKKMQLHGKGSQDPSTKVHIKVLQTVISFLLLCAIYF-LSIMIS
VWSF-----GSLKN--KPVFMFCKAMRFSYPSIHPFILIWGNKKLKQTFLSVLRQVRYWV
KGEKP--------------------SSP------------------------
>44bonobo
-----------------------M-------------TTFIPIIFSSLVVVIFVIGNFAN
GFIALVNSIEWFKK-QKISFADQILTALAVSRVGLLWVLLLNWYSTVLNPAFY-SVEVRT
TAYNVWAVTGHFSNWLATSLSIFYLLKIANFSNLIFLHLKRRVKSVILVMLLGPL----L
FLACQLFMINMKEIVR---TKEYEGNMTWKIKLRSA------VYLSDATVTTLGNLVPFT
LTLLCFLLLICSLCKHLKKMQLHGKGSQDPSTKVHIKVLQTVISFLLLCAIYF-LSIMIS
VWSF-----GSLKN--KPVFMFCKAIRFSYPSIHPFILIWGNKKLKQTFLSVLRQVRYWV
KGEKP--------------------SSP------------------------
>R44BOrang
-----------------------M-------------ITFLPTIFSILVVVIFVIGNFGN
GFIALVNSIEWVKR-QKISFADQILTALAVSRVGLLWALLLNWYSTVFNPAFY-SVGVRT
TVYDVWTVTGHFSNWLATSLSIFYLLKIANFSNLIFLHLKRRVKSVILVMLLGPL----L
FLACQLFVINMKEILR---TKEYEGNMTWKIKLRSA------MYLSDATITTLANLVPFT
LTLLSFLLLICSLCKHLNKMQLHGKGSQDPSTKVHIKVLQTVISFLLLCAIYF-LSIMIS
VWSF-----GSLEN--KPVFMFCKAIRFSYPSIHPFILIWGNKKLKQTFLSVLRQVRYWV
KGEKP--------------------SSP------------------------
>R44Agibbon
-------------------------------------------------------GNFAN
GFIALVNSIEWVKR-QKISFADQIVTALAVSRVGLLWVLLLNWYSTVLNPAFY-SVELRT
TAYNVWAVTGHFSNWLATSLSIFYLLKIANFSNLIFLHLKRRVKSVILVMLLGPL----L
FLACQLFVINMKEILR---TKEYEGNMTWKIKLRSA------MYLSDATITTLANLVPFT
LTLLSFLLLICSLCKHLNKMQLHGKGSQDPSTKVHIKVLQTVISFLLLCAIYF-LSIMIS
VWSF-----GSLEN--KPVFMFCKAIRFSYPSIHPFILIWGNKKLKQIFL----------
----------------------------------------------------
>R44gorilla
-----------------------M-------------TTFIPIIFSSLVMVMFVTGNFAN
GFIALVNSIESVKR-QKISYADQILTALAVSRIGLLWVLLLNWYSTVLNPAFY-SVEVRT
TAYNVWAVTGHFSNWLATSLSIFYLLKIANFSNLIFLHLKRRVKSVILVMLLGPL----L
```

```
FLACQLFVINMKEIVQ---TKEYEGNXTWKIKLRSA------VYLSDATVTTLGNLVPFT
LTLLCFLLLICSLCKHLKKMQLHGKGSQDPSMKVHIKALQTVTSFLLLCAIYF-LSIMIS
VWSL-----GSLKN--KPVFMFCKAMRFSYPSIHPFILIWGNKKLKQTFLSVLQQVRYWV
KGEKP--------------------SSP------------------------
>hT2R46
-----------------------M-------------ITFLPIIFSILIVVTFVIGNFAN
GFIALVNSIEWFKR-QKISFADQILTALAVSRVGLLWVLVLNWYATELNPAFN-SIEVRI
TAYNVWAVINHFSNWLATSLSIFYLLKIANFSNLIFLHLKRRVKSVVLVILLGPL----L
FLVCHLFVINMNQIIW---TKEYEGNMTWKIKLRSA------MYLSNTTVTILANLVPFT
LTLISFLLLICSLCKHLKKMQLHGKGSQDPSMKVHIKALQTVTSFLLLCAIYF-LSIIMS
VWSF-----ESLEN--KPVFMFCEAIAFSYPSTHPFILIWGNKKLKQTFLSVLWHVRYWV
KGEKP--------------------SSS------------------------
>R46bonobo
-----------------------M-------------ITFLPIIFSILIVVTFVIGNFAN
GFIALANSIEWFKR-QKISFADQILTALAVSRVGLLWVLLLNWYATELNPAFY-SIEVRI
TAYNLWAVINHFSNWLATSLSIFYLLKIANFSNLIFLRLKRRVKSVVLVILLGPL----L
FLVCHLFVINMNQIIW---TKEYEGNMTWKIKLRSA------MYLSNTTVTILANLVPFT
LTLISFLLLICSLCKHLKKMQLHGKGSQDPSMKVHIKALQTVTSFLLLCAIYF-LSIIMS
VWSF-----ESLEN--KPVFMFCEAITFSYPSTHPFILIWGNKKLKQTFLSVLWHVRYWV
KGEKP--------------------SSS------------------------
>R46gorilla
-----------------------M-------------ITFLPIIFSILIVVTFVIGNFAN
GFIALANSIEWFKR-QKISFADQILTALAVSRVGLLWVLLLNWYATELNPAFY-SIEVRI
TAYNVWAVISHFSNWLATSLSIFYLLKIANFSNLIFLRLKRRVKSVVLVILLGPL----L
FLVCHLFVINMNQIIW---TKEYEGNMTWKIKLRSA------MYLSDTTVTILANLVPFT
LTLISFLLLICSLCKHLKKMQLHGKGSQDPSMKVHIKALQTVTSFLLLCAIYF-LSVIMS
VWSF-----ESLEN--KPVFMFCEAITFSYPSTHPFILIWGNKKLKQTFLSVLWHVRYWV
KGEKP--------------------SSS------------------------
>R46chimp
-----------------------M-------------ITFLPIIFSILIVVTFVIGNFAN
GFIALANSIEWFKR-QKISFADQILTALAVSRVGLLWVLLLNWYATELNPAFY-SIEVRI
TAYNLWAVINHFSNWLATSLSIFYLLKIANFSNLIFLRLKRRVKSVVLVILLGPL----L
FLVCHLFVINMNQIIW---TKEYEGNMTWKIKLRSA------VYLSDATVTTLGNLVPFT
LTLLCFLLLICSLCKHLKKMQLHGKGSQDPSMKVHIKALQTVTSFLLLCAIYF-LSIIMS
VWSF-----ESLEN--KPVFMFCEAITFSYPSTHPFILIWGNKKLKQTFLSVLWHVRYWV
KGEKP--------------------SSP------------------------
>R66bonobo
-----------------------M-------------ITFLPIIFSILIVVTFVIGNFAN
GFIALANSIEWFKR-QKISFADQILTALAVPRVGLLWVLLLNWYATELNPAFY-SIEVRI
TAYNLWAVINHFSNWLATSLSIFYLLKIANFSNLIFLRLKRRVKSVVLVILLGPL----L
FLVCHLFVINMNQIIW---TKEYEGNMTWKIKLRSA------MYLSNTTVTILANLVPFT
VTLISFLLLVCSLCKHLKKMQLHGKGSQDPSTKVHIKALQTVISFLLLCAIYF-VSVIIS
VWSF-----KNLEN--KPVFMFCQAIGFSCSSAHPFILIWGNKKLKQPFLSVLWQMRYWV
KGEKP--------------------SSS------------------------
>hT2R45
-----------------------M-------------ITFLPIIFSILVVVTFVIGNFAN
GFIALVNSTEWVKR-QKISFADQIVTALAVSRVGLLWVLLLNWYSTVLNPAFC-SVELRT
TAYNIWAVTGHFSNWPATSLSIFYLLKIANFSNLIFLRLKRRVKSVILVVLLGPL----L
FLACHLFVVNMNQIVW---TKEYEGNMTWKIKLRRA------MYLSDTTVTMLANLVPFT
VTLISFLLLVCSLCKHLKKMQLHGKGSQDPSTKVHIKVLQTVISFFLLRAIYF-VSVIIS
VWSF-----KNLEN--KPVFMFCQAIGFSCSSAHPFILIWGNKKLKQTYLSVLWQMRY--
----------------------------------------------------
>R45chimp
-----------------------M-------------ITFLPIIFSILVVVTFVIGNFAN
GFIALVNSTEWVKR-QKISFADQIVTALAVSRVGLLWVLLLNWYSTVLNPAFY-SVELRT
TAYNIWAVTGHFSNWLATSLSIFYLLKIANFSNLIFLHLKRRVKSVILVMLLGPL----L
FLACHLFVVNMNQIVW---TKEYEGNMTWKIKLRRA------MYLSDTTVTMLANLVPFA
LTLISFLLLVCSLCKHLKKMQLHGKGSQDPSTKVHIKALQTVISFLLLCAIYF-VSVIIS
VWSF-----KNLEN--KPVFMFCQAIGFSCSSAHPFILIWGNKKLKQTFLSVLWQMRYWV
KGEKP--------------------SSP------------------------
>R45bonobo
-----------------------M-------------ITFLPIIFSILVVVTFVIGNFAN
GFIALVNSTEWVKR-QKISFADQIVTALAVSRVGLLWVLLLNWYSTVLNPAFY-SVELRT
TAYNIWAVTGHFSNWLATSLSIFYLLKIANFSNLIFLHLKRRVKSVILVMLLGPL----L
FLACHLFVVNMNQIVW---TKEYEGNMTWKIKLRRA------MYLSDTTVTMLANLVPFT
VTLISFLLLVCSLCEHLKKMQLHGKGSQDPSTKVHIKALQTVISFLLLCAIYF-VSVIIS
```

```
VWSF-----KNLEN--KPVFMFCQAIGFSCSSAHPFILIWGNKKLKQPFLSVLWQMRYWV
KGEKP--------------------SSS------------------------
>R45gorilla
--------------------------------------------FSILVVVTFVIGNFAN
GFIALVNSTEWVKR-QKISFADQIVTALAVSRVGLLWVLLLNWYSTVLNPAFY-SVELRT
TAYNIWAVTGHFSNWLATSLSIFYLLKIANFSNLIFLRLKRRVKSVILVMLLGPL----L
FLACHLFVVNMNQIVW---TKEYEGNMTWKIKLRRA------MYLSDTTVTMLANLVPFT
VTLISFLLLVCSLCKHLKEMHLHGKGSQDPSTKVHIKALQTVISFLLLCAIYF-VSVIIS
VWSF-----KNLEN--KPVFMFCQAIGFSCSSAHPFIVIWGNKKLKQTFLSV--------
----------------------------------------------------
>R45BOrang
-------------------------------------------------------GNFAN
GFIALVNSIESVKR-QKISYADQILTALAVSRIGLLWVLLLNWYSTVLNPAFY-SVEVRT
TAYNVWAVTGHFSNWLATSLSIFYLLKIANFSNLIFLRLKRRVKSVILVMLLGPL----L
FLACHLFVVNMNQIVW---TKEYEGNMTWKIKLRRA------MYLSDTTVTMLANLVPFT
VTLISFLLLVCSLCKHLKEMHLHGKGSQDPSTKVHIKALQTVISFLLLCAIYF-VSVIIS
VWSF-----KNLEN--KPVFMFCQAIGFSCSSAHPFIVIWGNKKLKQIFL----------
----------------------------------------------------
>hT2R47
-----------------------M-------------ITFLPIIFSILIVVIFVIGNFAN
GFIALVNSIEWVKR-QKISFVDQILTALAVSRVGLLWVLLLHWYATQLNPAFY-SVEVRI
TAYNVWAVTNHFSSWLATSLSMFYLLRIANFSNLIFLRIKRRVKSVVLVILLGPL----L
FLVCHLFVINMDETVW---TKEYEGNVTWKIKLRSA------MYHSNMTLTMLANFVPLT
LTLISFLLLICSLCKHLKKMQLHGKGSQDPSTKVHIKALQTVTSFLLLCAIYF-LSMIIS
VCNL-----GRLEK--QPVFMFCQAIIFSYPSTHPFILILGNKKLKQIFLSVLRHVRYWV
KDRSL--------------------RLHR------------FTRAALCKG--
>R47gorilla
-----------------------M-------------ITFLPIIFSILIVVIFVIGNFAN
GFIALVNSIEWVKR-QKISFVDQILTALAVSRVGLLWVLLLHWYATQLNPAFY-SVEVRI
TAYNVWAVTNHFSSWLATSLSMFYLLKIANFSNLVFLRIKRRVKSVVLVILLGPL----L
FLVCHLFVINMDETIW---TKEYEGNMTWKIKLRSA------MYHSNMTLTMLANFVPLT
LTLISFLLLICSLCKHLKKMQLHGKGSQDPSTKVHIKALQTVTSFLLLCAIYF-LSMIIS
VCNF-----GRLEK--QPVFMFCQAIIFSYPSTHPFILILGNKKLKQIFLSVLWHVRYWV
KDRSL--------------------RLHR------------FTRAALCKG--
>R47chimp
-----------------------M-------------ITFLPIIFSILIVVIFVIGNFAN
GFIALVNSIEWVKR-QKISFVDQILTALAVSRVGLLWVLLLHWYATQLNPAFY-SVEVRI
TVYNVWAVTNHFSSWLATSLSMFYLLKIANFSNLIFLRIKRRVKSVVLVILLGPL----L
FLVCHLFVINMDETIW---TKEYEGNMTWKIKLRSA------MYHSNMTLTILANFVPLT
LTLISFLLLICSLCKHLKKMQLHGKGSQDPSTKVHIKALQTVTSFLLLCAIYF-LSMIIS
VCNL-----GRLEK--QPVFMFCQAIIFSYPSTHPFILILGNKKLKQIFLSVLWHVRYWV
KDRSL--------------------RLHR------------FTRGALCVF--
>R47bonobo
-----------------------M-------------ITFLPIIFSILIVVIFVIGNFAN
GFIALVNSIEWVKR-QKISFVDQILTALAVSRVGLLWVLLLHWYATQLNPAFY-SVEVRI
TVYNVWAVTNHFSSWLATSLSMFYLLKIANFSNLIFLRIKRRVKSVVLVILLGPL----L
FLVCHLFVINMDETIW---TKEYEGNMTWKIKLKSA------MYHSNMTLTILANFVPLT
LTLISFLLLICSLCKHLKKMQLHGKGSQDPSTKVHIKALQTVTSFLLLCAIYF-LSMIIS
VCNL-----GRLEK--QPVFMFCQAIIFSYPSTHPFILILGNKKLKQIFLSVLWHVRYWV
KDRSL--------------------RLH------------------------
>R47BOrang
-----------------------M-------------ITFLPIIFSILIVVIFVIGNFAN
GFIALVNSIEWVKR-QKISFADQILIALAVSRVGLLWALLLHWYATELNLAFY-SVEVRI
TAYNVWAVTNHFSNWLATSLSMFYLLKIANFSNLIFLRIKRRVKSVILVILLGPL----L
FLVCHLFVINMNEIVW---TKEYEGNLTWKIKLRNA------VFLSNMTLTMLANFVPLT
LTLISFLLLICSLCKHLKKMQLHGKGSQDPSTKVHIKALQTVTCFLLLCAIYF-LSMIIS
VYNF-----GRLEK--KPVFMFCQAITFSYPSTHAFILIWGNKKLKQIFLSVLWHVRYWV
KDRSL--------------------RLHR------------FTRAALCKG--
>R47Ngibbon
-----------------------M-------------ITFLPIIFSILIVVVFVIGNFAN
GFIALVNSIEWVKR-QKISFADQILTALAVSRVGLLWVLLLHWYATELNPAFY-SVEVRI
TAYNIWAVTNHFSNWLATSLSMFYLLKIANFSNLIFLRIKRRVKSVVLVILLGPL----L
FLACHLFVINVNEIVW---TKEYEGNLTWKTKLRNA------MYLSNTTLTMLANLVPLT
LTLISFLLLICSLCKHLKKMQLHGKGSQDPSTKVHLKVLQTVTSFLLLCAIYF-LSMIIS
VYNF-----GRLEK--KPVSMFCQAIIFSYPSTHAFILIWGNKKL---------------
----------------------------------------------------
```

```
>R47Agibbon
--------------------------------------------FSILIVVVFVIGNFAN
GFIALVNSIEWVKR-QKISFADQILTALAVSKVGLLWVLLLHWYATELNPAFY-SVEVRI
TAYNIWAVTNHFSNWLATSLSMFYLLKIANFSNLIFLRIKRRVKSVVLVILLGPL----L
FLACHLFVINVNEIVW---TKEYEGNLTWKTKLRNA------MYLSNITLTMLANLVPFT
LTLISFLLLVCSLCKHLKKMQLHGKGSQDPSTKVHLKALQTVTSFLLLCAIYF-LSMTIS
VYNF-----GRLEK--KPVFMFCQAIIFSYPSTHAFILIWGNKKLKQIFLSV--------
----------------------------------------------------
>R45Rmacaq
-------------------------------------ITFLPIIFFILVVVTFVIGNFAN
GFVALVNSTEWVKR-QKISFADQILTALAVSRIGLLWVLLLHWYSIVLNPAFY-RVEVRI
TTYNVWAVTSHLSNWLATSLGIFYLLKIANFSNLIFLHLKRRVKSVILVMLLGPL----L
FLACHLFVINVNEIVR---TKEYEGNMTWKIKLMSA------MHFSNTTVTMLANLVPFT
LTLLSFVLLIWSLCKHLKKMQLYGKGSQDPSTKVHIKALQTVISFLFLCAIYF-LSIMIS
VWNL-----ERLEN--KSFFLFCKAIRIMYPSAHTFVLIWGNKKLKQTFLSVLWQMRYW-
----------------------------------------------------
>r43Ngibbon
----------------------------------------------ILVVVTFVIGNFAN
GFIALVNSVELVKR-QKISFADHILTALAVSRVGLLWVLLLNWYSTVLNPAFY-SVDLRT
TAYNLWAVTSHFSNWLATCLSIFYVLKIANFSNLIFLHLKRRVKSVILVMLLGPL----L
FLACHLFVINVNEIVR---TKEYEGNMTWKIKLMSA------MHFSNTTVTMLANLVPFT
LTLLSFVLLIWSLCKHLKKMQLYGKGSQDPSTKVHIKALQTVISFLFLCAIYF-LSIMIS
VWNL-----ERLEN--KSFFLFCKAIRIMYPSAHTFVLIWGNKKL---------------
----------------------------------------------------
>R43Slutung
--------------------------------------------FSILVVVTFVIGNFAN
GFIALVKSVELVKR-QKISFADQILTALAVSRVGLLWVLLLNWYATVLNPAFY-SVDVRT
TTYNLWAVTSHLSNWLATSLSIFYLLKIANFSNLMFLHLKRRVKSVILVILLGPL----L
FLACHLFVINMNEIVR---TKDYEGNMTWKIKLMSA------MYLSNTTVTMLANLVPFT
LTLLSFVLLICSLCKHLKKMQLHGKGSQDPSTKVHIKALQTVISFLLLCAIYF-LSVMIS
VWSL-----ERLEN--KPFFLFCKAIRIMYPSAHAFFLIWGNKKLKQTFLSV--------
----------------------------------------------------
>R45Slutung
--------------------------------------------FCILVVVTFVIGNFAN
GFIALVNCTEWVKR-QKISFADQILTALAVSRIGLLWVLLLHWYSIVLNPAFY-SVEVRT
TTYNVWAVTSHLSNWLATSLSIFYLLKIANFSNLIFLHLKRRVKNVILVMLLGTL----L
FLPCHLFVINMNQSVQ---TKDYEGNMTWKIKLMRT------MFLSDATVTTLANLVPFT
LTLISFLLLICSLCKHLKKMQLHGKGSQDPSTKVHIKALQTVISFLLLCAIYF-LSVMIS
VWSL-----ERLEN--KPFFLFCKAIRIMYPSAHAFFLIWGNKKLKQTFLSV--------
----------------------------------------------------
>R46CEmacaq
-----------------------M-------------ITFLSITFSILVGVIFVIGNFAN
GFIALVNSIEWVKR-QKISFADQILTALAVSRVGLLWVLLLNWYATVLNPAFY-RVEVRT
TTYNVWAVTNHFSNWLATSLSIFYLLKIANFSNLMFLHLKRRVKSVILVTLLGPL----L
FLVCHLFVMNMNHIVW---RKEYEGNITWRIKLRSA------MYLSNVSVTMLANFVPLT
LTLISFLLLICSLCKHLKKMQLHGKGSQDASTKVHIKALQTVTSFLLLCAIYF-LSMILS
VWNF-----E-LEK--KPVFMFCQAVIFSYPSTHPLILIWGNKKLKQIFLSVLWNVRYWV
KGQKP--------------------SSP------------------------
>R47Rmacaq
-------------------------------------------------------GNFAN
GFIALVNSTEWVKR-QKISFADQILTALAVSRIGLLWVLLLNWYATVLNPAFY-TIEVRT
TTYNVWVVTNHFSNWLGTSLSMFYLLKIATFSNLIFLHLKRRVMNVILVMLLGSL----L
FLACHLFVINMNQIVQ---TKEYEGNMTWNVKLRSA------IYFSNMTVTMLANFVPIT
LTLISFLLLICSLCKHLKKMQVHGKGSQDPTTKVHIKALQTVTSFLLLCAIHF-LAIMLS
VWNF-----ERLEN--KPVIMFCQATVFSYPSTHPFILIWGNKKLKQIFL----------
----------------------------------------------------
>R44Hbaboon
-----------------------M-------------ITFLPIIFSILVVVTFVIGNFAN
GFIALVNSTEWVKR-QKISFADQILTALAVSRVGLLWVLLLNWYATVLNPAFY-SVEVRT
TTYNVWAVTNHFSNWLATSLSIFYLLKIANFSNLIFLHLKRRVKNVILVMLLGPL----L
ILACHLFMVNMNEIVR---TKEYEENMTWKYILRNA------IYHPGMTVTTLQNLVPFT
LTLISFLLLICSLCKHLKKMQLHGKGPQDPSTKVHIKALQIVISFLLLCVIYF-VSVIIS
IWSF-----ESLGN--KPVFMFCQAIRFSYPSAHPFIVIWGNKKLKQTFLSVLWNVRYWV
KGQKP--------------------SSL------------------------
>R8Rmacaq
-----------------------M-------------ITFLPIIFSILVVVTFVIGNFAN
```

```
GFIALVNSVELVKR-QKISFADQILTALAVSRVGLLWVLLLNWYATVLNPAFY-SVEVRT
TTYNVWAVTNHFSNWLATSLSIFYLLKIANFSNLIFLHLKRKVKNVILVMLLGPL----L
VLACHLFMVNMNEIVR---TKEYEENMTWKYILRNA------IYHPGMTVTTLQNLVPFT
LTLISFLLLICSLCKHLKKMQLHGRGSQDPSTKVHIKALQIVISFLLLCVIYF-VSVIIS
IWSF-----ESLGN--KPVLMFCQAIRFSYPSAHPFIMIWGNKKL---------------
----------------------------------------------------
>R46Rmacaq
-----------------------M-------------ITFLPIIFSILVVFTFVIGNFAN
GFIALVNSIEWVKR-QKISFADQILTALAVSRVGLLWILLLNWYSTVLNPAFY-SVEVRT
IVYNLWAVINHFSNWLATSLSIFYLLKIANFSNLIFLHLKRRVKSVVLVILLGPL----L
FLVCHLFVVNMNEIIQ---TKEYEGNMTWKSKLRSA------MYLSNTTVTILANLVPFI
LTLISFLLLICSLCKHLKKMQLRDKGSQDPSTKVHIKALQTVIS-LSLCAIYF-LSIMIS
SWSL-----GRVEN--KAIFMFCKAIRFSYPSAHAFILIWGNKKLKQTLLSVLWNVRYCV
KGQKL--------------------QSP------------------------
>R43Rmacaq
-----------------------M-------------ITFLPIIFSILVVFTFVIGNFAN
GFIALVNSIEWVKR-QKISFADQILTALAVSRVGLLWILLLNWYSTVLNPAFY-SVEVRT
IAYNLWAVINHFSNWLATSLSIFYLLKIANFSNLIFLHLRRRVKSVVLVILWGPL----L
FLVCHLFVVNMNEIIQ---TKEYEGNMTWKSKLRSA------MYLSNTTVTILANLVPFI
LTLISFLLLICSLCKHLKKMQLRDKGSQDPSTKVHIKALQTVIS-LSLCAIYF-LSIMIS
SWSL-----GRVEN--KAIFMFCKAIRFSYPSAHAFILIWGNKKLKQTLLSVLWNVRYCV
KGQKL--------------------QSP------------------------
>R43Hbaboon
-----------------------M-------------ITFLPIIFSILVVVTFVIGNCAN
GFIALVNSTEWVKR-QKISFADQILTALAVSRVGLLWVLLLNWYATVLNPAFY-SVEVRT
IVYNLWAVINHFSNWLATSLSIFYLLKIANFSNLIFLHLKRRVKSVVLVILLGPL----L
FLVCHLFVVNMNEIVR---TKEYEGNMTWKSKLRSA------MYLSNTTVTILANLVPFI
LTLISFLLLICSLCKHLKKMQLRDKGSQDPSTKVHIKALQTVIS-LLLCVIYF-LSIMIS
SWSL-----GRVEN--KAVFMFCKAIRFSYPSAHAFILIWGNKKLKQTLLSVLWNVRYCV
KGQKL--------------------PSP------------------------
>R46Hbaboon
-----------------------M-------------ITFLPITFSILIVVIFFIGNFAN
GFIALINSIEWVKR-QKISFAGQILTALAVSRVGLLWVLSLHWYATEFNLAFH-SVEVRS
TAYNVWVVTNHFSNWLSTSLSMFYLLRIATFSNLIFLHLNRRVKSVILVTLLGPL----L
FLVCQLFVMNMNQIVR---TKEYEGNMTWKIKLKSA------MYLSNTTVAMLANFVPLT
LTLISFLLLICSLCKHLKKMRVHGKGSQDPSTKVHTKALQIVTSFLLVCAIYF-LSIILS
VWNS-----GGLEN--KPFFMFCQAIKFSYPSTHPFILIWGNKTLKQTFLSVLRNVRYWV
KGQKP--------------------SSP------------------------
>r2Rhowler
----------------------------------------------ILVVVTFVLGNFAN
GFIALVNSIEWVKR-QKISFADQILTALAVSRVGLLWMLLLHWYSTVLNPAFYKSAEVRI
TTYNFWAVTSHFSNWLATSLSIFYLLKIVNFSNFIFVRLKRRVTSVIRVIMLGPL----L
LLICQLFVVNMNEIVR---TKEYEGNMTWKIKLRNA------MCLSNVTIALLANLVPFT
LTLVSFLLLLCSLCKHLKKMQLHGKRCQDPSTKVHIKALQTVISFLLLCATYF-LSIMIS
VWNF-----ENMEK--KAVFMFCQAIRFSYPSTHPFILIW--------------------
----------------------------------------------------
>r3Rhowler
----------------------------------------------VLVVVTFVLGNFAN
GFIALVNSIEWIKR-QKISFADQILTALAVSRVGLLWVILLHWYATVLNPDLY-SLEARI
TNNNAWAVTNHFSNWLATSLSIFYLLKIANFSNFIFLHLKRRLKSVIPVIQFGAL----L
FLVCHLVVVNMDESMW---TKEYEGNVSWKIKLNDT------THLSDMTVTTLANLIPFT
LSLTSSLLLICSLSKHLKKMQLHGKGSTDLNIKVHIKALRTVISFLLLFVIYV-LSLTTS
IWNL----RRTLQN--KPVFMLCQITIIIYPSFHSFILIW--------------------
----------------------------------------------------
>R15BOrang
-------------------------------------------------------GNFAN
GFIALVNSIEWVKR-QKISFADQILTALAVSRMGLLWVILLHWNATVLNPGLH-SLGVRI
TTINAWAVTNHFRIWIATSLSIFYLLKIANFSNFIFLHLKRRIKSVIPVILLGSL----L
FLVCHIVVVVNMDESMW---TKEYEGNVSWEIKLSDP------THLSEMTLTMLANLIPFT
LSLLSFLLLICSLSKHLKKMQLHGKGSPDSNTKVHIKVLQMVISFLLLFAVYF-LSPITS
IWKF----RRRLQN--EPVLMLCQTTAIIYPSFHSFILIWGSKKLKQTFLLILCQIKC--
----------------------------------------------------
>hT2R50
-----------------------M-------------ITFLYIFFSILIMVLFVLGNFAN
GFIALVNFIDWVKR-KKISSADQILTALAVSRIGLLWALLLNWYLTVLNPAFY-SVELRI
TSYNAWVVTNHFSMWLAANLSIFYLLKIANFSNLLFLHLKRRVRSVILVILLGTL----I
```

```
FLVCHLLVANMDESMW---AEEYEGNMTGKMKLRNT------VHLSYLTVTTLWSFIPFT
LSLISFLMLICSLYKHLKKMQLHGEGSQDLSTKVHIKALQTLISFLLLCAIFF-LFLIVS
VWSP-----RRLRN--DPVVMVSKAVGNIYLAFDSFILIWRTKKLKHTFLLILCQIRC--
----------------------------------------------------
>R50gorilla
-----------------------M-------------ITFLYIFFSILILVLFVLGNFAN
GFIALVNFIDWVKR-KKISSADQILTALAVSRIGLLWALLLNWYLTVLNPAFY-SVELRI
TSYNAWVVTNHFSMWLAASLSIFYLLKIANFSNLIFLHLKRRVRSVILVILLGTL----I
FLVCHLLVANMDESMW---AEEYEGNMTGKMKLRNT------VHLSYLTVTTLWSFIPFT
LSLISFLMLICSLCKHLKKMQLHGEGSQDLSTKVHIKALQTLISFLLLCAIFF-LFLIIS
VWSP-----RRLQN--DPVVMVSKAVGNIYLAFDSFILIWRTKKLKHTFLLILCQIRC--
----------------------------------------------------
>r50chimp
-----------------------M-------------ITFLYIFFSILIMVLFVLGNFAN
GFIALVNFIDWMKR-KKISSADQILTALAVSRIGLLWTLLLNWYLTVLNPAFY-SVELRI
TSYNAWVVTNHFSMWLAASLSIFYLLKIANFSNLIFLHLKRRVRSVILVILLGTL----I
FLVCHLLVANMDESMW---AEEYEGNITGKMKLRNT------VHLSYLTVTTLWSFIPFT
LSLISFLMLICSLCKHLKKMQLHGEGSQDLSTKVHIKALQTLISFLLLCAIFF-LFLIIS
VWSP-----RRLRN--DPVVMVSKAVGNIYLAFDSFILIWRTKKLKHTFLLILCQIRC--
----------------------------------------------------
>r50Ngibbon
--------------------------------------------------------NFAN
GFIALVNFIDWVKR-KKISSADQILTALAVSRIGLLWALLLNWYLTVLNPAFY-GVELRI
TSYNAWVVTNHFSMWLAASLSIFYLLKIANFSNLIFLHLKRRVRSVILVILLGTL----I
FLVCHLLVANMDENIW---AEEYEGNMTEKMKLRNT------VHLSYMTVTTLWSFIPFT
LSLISFLMLICSLCKHLKKMQLHGEGSQDLSTKVHIKALQTLISFLLLCAIFF-LFLIIS
VWSP-----RRLQN--DPVVMVSKAVGNIYLAFDSFILIWRTK-----------------
----------------------------------------------------
>R50BOrang
-----------------------M-------------VTFLHIFFSILILVLFVLGNFAN
GFIALVNFIDLVKR-KKISSADQILTALAVSRIGLLWALLLNWYLTVLNPAFY-SVELRI
TSYNAWVVTNHFSMWLAASLSIFYLLKIANFSNLIFLHLKRRVRSVILVILLGPL----T
FLVCHLFVANMDESMS---AEEYEGNMTGKLKLRNT------VHLSYLTVTTLWSFIPFT
LSLISFLMLICSLCKHVKKMQLHGEGSQDLSTKVHIKALQTLISFLLLCAIFF-LFLIIS
IWNP-----RRLQN--DPVVVVSKAVGNIYLALDSFILIWRTKKLKHTFLLILCQIRC--
----------------------------------------------------
>r50CEmacaq
-----------------------M-------------IPFLHIFFSVLILVLFVLGNFAN
GFIALVNFIDWVKR-KKISLADQILTALAVSRVGLLWALLLNWYLTELNPAFS-SVELRI
TSYNAWVVTNHFSMWLAASLSIFYLLKIANFSNLSFLNLKRRVRSIILVILLGSL----L
FLVCHLLAVNMDENMW---TEEYEGNMTGKMKLRNA------AHLSYMTVTTLWSFIPFM
LSLISFLMLIFSLCKHLKKMQLHGEGSRDPSTTVHIKALQTLISFLLLCAIFF-LFLIIS
VWSP-----RRLQN--EPVFMVCKAVGNIYLSFDSFVLIWRTKKLKHIFLLILCQIRC--
----------------------------------------------------
>R50Rmacaq
-----------------------M-------------IPFLHIFFSVLILVLFVLGNFAN
GFIALVNFIDWVKR-KKISLADQILTALAVSRVGLLWALLLNWYLTELNPAFY-SVELRI
TSYNAWVVTNHFSMWLAASLSIFYLLKIANFSNLSFLNLKRRVRSIILVILLGSL----L
FLVCHLLAVNMDENMW---TEEYEGNMTGKMKLRNA------AHLSYMTVTTLWSFIPFM
LSLISFLMLIFSLCKHLKKMQLHGEGSRDPSTTVHIKALQTLISFLLLCAIFF-LFLIIS
VWSP-----RRLQN--EPVFMVCKAVGNIYLSFDSFVLIWRTKKLKHIFLLILCQIRC--
----------------------------------------------------
>hT2R48
-----------------------M-------------MCFLLIISSILVVFAFVLGNVAN
GFIALVNVIDWVNT-RKISSAEQILTALVVSRIGLLWVMLFLWYATVFNSALY-GLEVRI
VASNAWAVTNHFSMWLAASLSIFCLLKIANFSNLISLHLKKRIKSVVLVILLGPL----V
FLICNLAVITMDERVW---TKEYEGNVTWKIKLRNA------IHLSSLTVTTLANLIPFT
LSLICFLLLICSLCKHLKKMRLHSKGSQDPSTKVHIKALQTVTSFLMLFAIYF-LCIITS
TWNL-----RTQQS--KLVLLLCQTVAIMYPSFHSFILIMGSRKLKQTFLSVLWQMTR--
----------------------------------------------------
>r48chimp
-----------------------M-------------VYFLLIILSILVVFAFVLGNFSN
GFIALVNVIDWVNT-RKISSADQILTALVVSRIGLLWVMLFLWYATVFNSALY-GLEVRI
VASNAWAVMNHFSIWLAASLSIFYLLKIANFSNLIFLHLKKRIKSVVLVILLGPL----V
FLICNLAVITMDERVW---TKEYEGNVTWKIKLRNA------IQLSNLTVTTLANLIPFT
LSLICFLLLICSLCKHLKKMRLHSKGSQDPSTKVHIKALQTVTSFLMLFAIYF-LCIITS
```

```
TWNL-----RTQQS--KLVLLLCQTVAIMYPSFHSFILIMGSRKLKQTFLSVLWQMTC--
----------------------------------------------------
>R48bonobo
-----------------------M-------------MCFLLIILSILVVFAFVLGNFSN
GFIALVNVIDWVNT-RKISSADQILTALVVSRIGLLWVMLFLWYATVFNSALY-GLEVRI
VASNAWAVMNHFSIWLAASLSIFCLLKIANFSNLIFLHLKKRIKSVVLVILLGPL----V
FLICNLAVITMDERVW---TKEYEGNVTWKIKLRNA------IQLSSLTVTTLANLIPFT
LSLICFLLLICSLCKHLKKMRLHSKGSQDPSTKVHIKALQTVTSFLMLFAIYF-LCIITS
TWNL-----RTQQS--KLVLLLCQTVAIMYPSFHSFILIMGSRKLKQTFLSVLWQMTR--
----------------------------------------------------
>R48gorilla
-----------------------M-------------MCFLLIISSILVVFAFVLGNVAN
GFIALVNVIDWVNT-RKISSADQILTALVVSRIGLLWVMLFLWYATVFNSALY-GLEVRI
VASNAWAVMNHFSMWLAAILSIFCLLKIANFSNLIFLHLKKRIKSVVLVILLGPL----V
FLICNLAVITMDERVW---TKEYEGNVTWKIKLRNA------IQLSNLTVTTLANLIPFT
LSLICFLLLICSLCKHLKKMRLHSKGSQDPSTKVHIKALQTVTSFLMLFAIYF-LCIITS
TWNL-----RTQQS--KLVLLLCQTVAIMYPSFHSFILIMGSRKLKQTFLSVLWQMTC--
----------------------------------------------------
>r64chimp
-----------------------M-------------VYFLLIILSILVVFAFVLGNFSS
GFIALVNVIDWVNT-RKISSADQILTALVVSRIGLLWVMLFLWYATVFNSALY-GLEVRI
VASNAWAVMNHFSIWLAASLSIFYLLKIANFSNLIFLHLKKRIRSVVLVILLGPL----V
FLICNLAVITMDERVW---TKEYEGNVTWKIKLRNA------IHLSDLTVSTLANLIPFI
LTLICFLLLICSLHKHLKKMQLHGKGSQDLSTKVHIKALQTVISFLMLYAIYF-LYLITL
TWNL-----WTQQN--KLVFLLCQTLGIMYPSFHSFFLIMGSRKLKQTFLSVLCQVTCLV
KGQQP--------------------STP------------------------
>R64bonobo
-----------------------M-------------VYFLLIILSILVVFAFVLGNFSN
GFVALVNVIDWVKT-RKISSADQILTALVVSRIGLLWVILFHWYANVFNSALY-SSEVGA
VASNISAIINHFSIWLAASLGIFYLLKIANFSNLIFLHLKKRIRSVVLVILLGPL----V
FLICNLAVITMDERVW---TKEYEGNVTWKIKLRNA------IHLSDLTVSTLANLIPFI
LTLICFLLLICSLHKHLKKMQLHGKGSQDLSTKVHIKALQTVISFLMLYAIYF-LYLITL
TWNL-----WTQQN--KLVFLLCQTLGIMYPSFHSFFLIMGSRKLKQTFLSVLCQVTCLV
KGQQP--------------------STP------------------------
>R48BOrang
----MLKELXPVLLSLHFFNCSNV-------------ICFLLIISSILVVFAFDFGNVAX
GFIALVNVIDWVKT-QKISSADQILTALVVSRIGLLWVMLSLWYATVFNSALY-GLEVKI
VASNAWAVTNHFSMWLAANLSIFYLLKIANFSNLIFLHLKKRIKSVVLVVVLGPL----V
FLICNLAVITMXDSVW---TKEYEGNVTWKIKLRNA------IHLSNLTVTTLANLIPFT
LSLICFLLLICSLCKHLKKMRLHSKGSQDPSTKFHIKALQTVTSFLVLFTIYF-LCIITS
TWNR-----RTQQS--KLVLLLCQIXAIMYPSFHSFILIMGSRKLKQTFLSVLWQMTC--
----------------------------------------------------
>r48Rmacaq
-----------------------M-------------MYFLFIILSILLVFAFVLGNFAN
GFIALVNVIDWVKT-RKISLVDQILTALVIYRIGLLWAILLYWYATMFNSALC-SSEVRI
FASNISAIINHFSIWLAASLSIFYLLKIANFSNLIFLHLQKRIKSVVRVMLLGPL----V
FLICNLAVVTTDEGVW---TKEYEGNVTWKIKLKNA------IHLSNLTISTLANLIPFT
LTLICFLLLIYSLCKHLKKIQLHGKGSQDLSTKVHIKSLQTVISFLMLFAIYS-LCLISL
TWSP-----WKQQN--KLVFLLCQTLAIMYPSFHSFILIMGNRKLKQTFLSVLWQVT---
----------------------------------------------------
>r48CEmacaq
-----------------------M-------------MYFLFIILSILLVFAFVLGNFAN
GFIALVNVIDWVKT-RKISLVDQILTALVIYRIGLLWAILLYWYATMFNSALC-SSEVRI
FASNISAIINHFSIWLAASLSIFYLLKIANFSNLIFLHLQKRIKSVVRVMLLGPL----V
FLICNLAVVTTDEGVW---TKEYEGNVTWKIKLKNA------IHLSNLTISTLANLIPFT
LTLICFLLLIYSLCKHLKKIQLHGKGSQDLSTKVHIKSLQTVISFLMLFAIYS-LCLISL
SWSP-----WKQQN--KLVFLLCQTLAIMYPSFHSFILIMGNRKLKQTFLSVLWQVT---
----------------------------------------------------
>R64Rmacaq
------------------------------------------------------IGNFAN
GFIALVNVIDWVKT-RKISLVDQILTALVIYRIGLLWAILLYWYATMFNSALC-SSEVRI
FASNISAIINHFSIWLAASLSIFYLLKIANFSNLIFLHLQKRIKSVVRVMLLGPL----V
FLICNLAVVTTDEGVW---TKEYEGNVTWKIKLKNA------IHLSNLTISTLANLIPFT
LTLICFLLLIYSLCKHLKKIQLHGKGSQDLSTKVHIKSLQTVISFLMLFAIYS-LCLISL
TWSP-----WKQQN--KLVFLLCQTLAIMYPSFHSFILIRGNRKLK--------------
----------------------------------------------------
```

```
>hT2R49
-----------------------M-------------MSFLHIVFSILVVVAFILGNFAN
GFIALINFIAWVKR-QKISSADQIIAALAVSRVGLLWVILLHWYSTVLNPTSS-NLKVII
FISNAWAVTNHFSIWLATSLSIFYLLKIVNFSRLIFHHLKRKAKSVVLVIVLGSL----F
FLVCHLVMKHTYINVW---TEECEGNVTWKIKLRNA------MHLSNLTVAMLANLIPFT
LTLISFLLLIYSLCKHLKKMQLHGKGSQDPSTKIHIKALQTVTSFLILLAIYF-LCLIIS
FWNF-----KMRPK--EIVLMLCQAFGIIYPSFHSFILIWGNKTLKQTFLSVLWQVTCWA
KGQNQ--------------------STP------------------------
>r49chimp
-----------------------M-------------MSFLHIVFSILVVVAFILGNFAN
GFIALINFIAWVKR-QKISSADQIIAALAVSRVGLLWVILLHWYSTVLNPTSS-NLKVII
FISNAWAVTNHFSIWLATSLSIFYLLKIVNFSRLIFHHLKRKAKSVVLVIVLGSL----F
FLVCHLVMKNTYINVW---TEECEGNVTWKIKLRNA------MHLSNLTVAMLANLIPFT
LTLISFLLLIYSLCKHLKKMQLHGKGSQDPSTKIHIKALQTVTSFLILLAIYF-LCLITS
FWNS-----KMRPK--EIVLMLCQAFGIIYPSFHSFILIWGNKTLKQTFLSVLWQVTCWA
KGQNQ--------------------STP------------------------
>R49Hbaboon
-----------------------M-------------MSFLHIVFSILVVVAFILGNFAN
GFIALINFIAWVKR-QKISSADQIIAALAVSRVGLLWVILLHWYSTVLNPTSS-NLKVTI
FISNAWAVTNHFSIWLATSLSIFYLLKIVNFSRLIFHHLKRKAKSVVLVIVLGSL----F
FLVCHLVMKNTYINVW---TEEYEGNVTWKIKLRNA------MHLSNLTVAMLANLIPFT
LTLISFLLLIYSLCKHLKKMQLHGKGSQDPSTKIHIKALQTVTSFLILLAIYF-LCLITS
FWNX-----KMRPK--EIVLMLCQAFGIIYPSFHSFILIWGNKTLKQTFLSVLWRVTCWA
KGQNQ--------------------STP------------------------
>R49gorilla
-----------------------M-------------MSFLHIVFSILVVVAFILGNFAN
GFIALINFIAWVKR-QKISSADQIIAALAVSRVGLLWVILLHWYSTVLNPTSS-NLKVTI
FISNAWAVTNHFSIWLAASLSIFYLLKIVNFSRLIFHHLKRKAKSVVLVIVLGSL----F
FLVCHLVMKSTYINVW---TEEYEGNVTWKIKLRNA------MHLSNLTVAMLANLIPFT
LTLISFLLLIYSLCKHLKKMQLHGKGSQDPSTKIHIKALQTVTSFLILLAIYF-LCLITS
FWNS-----KMRPK--EIVLMLCQAFGIIYPSFHSFILIWGNKTLKQTFLSVLWRVTCWA
KGQNQ--------------------STP------------------------
>R49Agibbon
----------------------------------------------ILVVVAFILGNFAN
GFIALINFIAWVKR-QKISSADQIIAALAVSRVGLLWVILLHWYSTVLNPTSS-SLKVTI
FISNAWAVTNHFSIWFATSLSIFYLLKIVNFSRLIFHYLKRKXKSVVLVIVLGSL----F
FLICHLVMENTDINVW---TKEYEGNITWKIKLRNA------MHLSNLTVTMLANLIPFT
LTLISFLLLIYSLCKHLKKMQPHGKGSQDPSTKIHIKALQTVTSFLILLVVYF-LCLITS
FWNS-----KMQPK--ELVLMLCQAFGIIYPSFHSFILIWGNKTLKQTFLSVLWQVT---
-------------------------AGE------------------------
>r49Ngibbon
------------------------------------------------VVVAFILGNFAN
GFIALINFIAWVKR-QKISSADQIIAALAVSRVGLLWVILLHWYSTVLNPTSS-SLKVTI
FISNAWAVTNHFSIWFATSLSIFYLLKIVNFSRLIFHYLKRKAKSVVLVIVLGSL----F
FLICHLVMENTYINVW---TKEYEGNITWKIKLRNA------MHLSNLTVTMLANLIPFT
LTLISFLLLIYSLCKHLKKMQPHGKGSQDPSTKIHIKALQTVTSFLILLVVYF-LCLITS
FWNS-----KMQPK--ELVLMLCQAFGIIYPSFHSFILIWGNK-----------------
----------------------------------------------------
>R49BOrang
-----------------------M-------------MSFLHIVFSILVLVAFILGNFAN
GFIALTNFIAWVKR-QKISSADQIIAALAVSRVGLLWVILLHWYSTVLNPTSS-SLKVTI
FVSNAWAVTNHFSIWLATSLSIFYLRKIVNFSRLIFHHLKRKAKSVVLVIVLGAL----F
FLVCHLVMENTYINVW---TKEYEGNVTWKIKLRNA------MYLSNLTVATLANLIPFT
LTLISVLLLIYSLCKHLKKMQLHGKGSQDPSTKIHIKALQTVTSFLILLAIYF-LCLIIS
FWXS-----ETQLK--ELVLMLCQAVGIIYPSFHSFILIWGNKTLRQTFLSVLWQVTCWS
KGQNQ--------------------STP------------------------
>r49CEmacaq
-----------------------M-------------MSFLPIVFSILVVVAFVLGNFAN
GFIALINFIAWFKR-QKISSADQIIAALAVSRVGLLWVIVLHWYATVLNPNSS-SLKVRI
FLSNAWAVTNHFSIWLATSLSIFYLLKIVNFSRLIFHHLKRKVKSVVLGIVSGAL----L
FLVCDLVAENVYINVW---TKEYERNITWKIKLRNA------TYLSNLIVVTLANLIPFT
LTLISFLLLICSLCKHLKKMKLYGKGSQDPSTKIHIKALQTVTSFLILFAVYF-LCLIVS
FWNY-----KKQQK--ELVLILCQAIGIIYPSFHSFILIWGNKKLKQNFLSILWQVTCWA
KGQNL--------------------STP------------------------
>r49Rmacaq
-----------------------M-------------MSFLPIVFSILVVVAFVLGNFAN
```

```
GFIALINFIAWFKR-QKISSADQIIAALAVSRVGLLWVIVLHWYATVLNPNSS-SLKVRI
FLSNAWAVTNHFSILLATSLSIFYLLKIVNFSRLIFHHLKRKVKSVVLGIVSGAL----L
FLVCDLVAENVYINVW---TKEYERNITWKIKLRNA------TYLSNLIVVTLANLIPFT
LTLISFLLLICSLCKHLKKMKLYGKGSQDPSTKIHIKALQTVTSFLILFAVYF-LCLIVS
FWNY-----KKQQK--ELVLILCQAIGIIYPSFHSFILIWGNKKLKQNFLSILWQVTCWA
KGQNL--------------------STP-----------------------
>R136Canis
----------------------M-------------LPLLQSIFSILVMTEFVLGNFAN
GFIVLVNYIAWVKR-QKISSADQILTGLAVSRIGLLWVILINWYATLLNPALY-SLEVRL
LVHIAWTANNHFSIWLATSLSVFYLFKIANFSNLIFLRLKWRVKSVVFVMLLGSL----F
FLVFHVAVVSIYEQMQ---MKEYEGNITRQTKLRDI------AQLMNMTVFTLMNFVPFA
ISLTSFLLLIFSLWKHLKKMRSGGKRYQDSSTKVHIKAMQTVISFLLLLVCYF-LTLIAI
VWSS-----NRLQN--KLIFLLCKAIGILYPSSHSFILIWGNKKLREDFLSFLWQLKGWL
KKGYKRSIMCLLGENKLMESVIFFSSTSF----SNEYVIEQFPKIYLKKSFL
>r31panda
----------------------M-------------LTLLSGLFSILVITEFVLGNFAN
GFIAVVNCTDWVKR-QKMSSADRILTALAISRISLLWAMLVNWYAVVLNPALY-IFKVRL
LVHVAWTASNHFSIWLATSLSIFYLFKIANFSSLIFLRLKWRVKSVVVVILLESL----F
LLVFQIAVVSMYEKIQ---MKECEGNVTRQTKLGSI------LHLSSMTIFTLANFVPFA
ISLTSFLLLIFSLRKLLKRMQSSGKRSQDPSTKVHIRAMQTVISFLFLLAGHF-TTLIIT
VWSS-----NGLQN--ELFFMICQAFGFVYPSSHSFILIWGNKKLKQAFLSVLYQGKYWL
KEQKL--------------------STP-----------------------
>PANDA_014286
----------------------M-------------LTLLSGLFSILIITEFVLGSFAN
GFIAVVNCTDWVKR-QKMSSADRILTALAISRISLLWVMLMNWYAAMLNPASF-SLEARL
LVHVAWAASNHFSIWLATSLSIFYLFKIANFSSLIFLRLKWRVKSVVVVILLGSL----F
FLVFQIAVVSMYEKIQ---TKDFKGNVTRQTKLGSI------LHLSSMTIFTLANFVPFA
ISLTSFLLLIFSLRKHLKRMQSSGRRSQDPSTKVHIRAMQTVISFLFVLVGYF-PALIVT
IWSS-----KWLPN--KLILLLCKAIGIMYPSSHSFILIWGNKKLRKALLSFVWQLRC--
---------------------------------------------------
>r66Dbat
------------------------------------MALLPAILSSLFTIQFVLGYFAN
GFIAVVNCIDWVKR-QKISCADGILTALAVSRIGLLCVLLLNWYATVFNRAFY-SLEVKL
IVHIAWMTSHHFSLWFATSLSIFYLLKIANFSSFLFLHLKWRAKRVVLMILLGAL----V
FLVFRLAVVSTDEKMQ---MNEYKGNITWETNLRDI------MHLSNHTLFMLSNIVPFT
MSLTAVLLLVFSLWKHLKNMQLSGKGTQDPSTEVHLRAMQTGISFLLTFVIFF-FSQIIS
LWNS-----STQQN--NLVYMVCKVLGILYPSSHSFILIWGNKKLRQAFLSFLWQLRCWL
RKGK-----------------------------------------------
>r46Bbat
------------------------------------MALLPAILSSLFTIQFVLGYFAN
GFIALVNCIDWVKR-QKISCADGILTALAVSRICLLCVLVLNWYAIVFNLAFY-SLDVKL
IFHIAWMTSHHFSLWLATSLSIFYLLKIANFSSLLFLHLKWRAERVVIMILLGAL----V
FLVFHLAVVGTDEKMQ---MNEDKGNITWETNLGDI------MHLSNQTLFMLANFIPFT
MSLAAVLLLIFSMWKHLKNMQLSGKGTQDPSTEVHIRAMQTVISFLLVFFIFF-FTQIIS
LWNS-----STQQN--NSLHMVCKVLGILYPSSHSFILIWGNKKLRQAFLSFLWQLRCWL
RKGK-----------------------------------------------
>R45Bbat
LKVRIVKDSRPIILYLAFF-FSDM-------------IHLLPSILSILIIAQYVLGSFAN
GFIALVNCIDWVKK-HKISCADRILTALAFSRICLLWIIIFNWYGTVLHPAIY-SSEVKT
IVHIAWVASNHFSLWLATSLSILYLLKIANFSCLLFLHLKWRAERVVIMILWGTS----V
FLVFHLAVVGTDEKMK---MNVYKGNITWESKLRDI------THLSNGIIFVLVHFIPFT
TSLTAVLLLIFSMWKHLKKMQVSGKGSQDPSTEVHIRAMQTVISFLLLFVIYF-FAQIIS
FWNF-----SIMQN--NSVPLLCQVFGILYPSSHSFILIWGNKKLRQAFLSLLRQLRCWL
KERR-----------------------------------------------
>r31cattle
----------------------M-------------ITLVSSIISILMVAEFVLGNFVN
GFIALVNCNDWLRK-QKVSLADGILTALAVCRIVLLWTILINWYATMYNPALY-S--LRI
VIRVAWTVSNHFSNWLATSLSIFYLFKIANFSSLIFLHLKWRVKSVVLMMILGTS----V
ILFFQVAVLSIDETIQ---TSEYERNITEKTKLRDI------LHLSNMTLLTLTNFIPFT
MSLVSFLLLIFFLWKHLRKMQLNGKRSQDPSTKVHIKAMQTVISFLFLFATYM-LTVILT
IWNS-----NELQK--ELVQMLFQALAITYPSIHSFILIWTNRKLTQTFLSFLWQPRCWL
KVKGTR---------------------------------------------
>R46Dyak
------------VTLDLD-FLDM-------------ITLVSSIISILMVAEFVLGNFVN
GFIALVNCNDWLRK-QKFSLADGILTALAVCRIVLLWTILINWYATMYNPALY-S--LRI
VIRVAWTVSNHFSNWLATSLSIFYLFKIANFSSLIFLHLKWRVKSVVLMMILGTS----V
```

```
ILFFQVAVLSIDETIQ---TSEYERNITEKTKLRDV------LHLSNMTLLTLTNFIPFT
MSLVSFLLLIFFLWKHLRKMQLNGKRSQDPSTKVHIKAMQTVISFLFLFATYM-LTVILT
IWNS-----NELQK--ELVQMLFQALAITYPSIHSFILIWTNRKLTQTFLSFLWQPRCWL
-----------------------------------------------------
>r31Dyak
-----------------------M-------------IMFMSNIVSILLMTEFVLGNFAN
VLLALVNCNDWTKR-PKIS-ADGILTALAFCRIVMFWAMLINWYVIVYNLTLY-NSEVKM
IVHVALTVSNHFSNWLATSLSIFYLLKIANFSSLIFLHLKWRVKSVVLMMMLGTS----L
FLFFQVAVLSMDEAIQ---TNEYEGNTTQKIKLRDT------LHLSNVTLFTLTNFIPFT
MSLTSFLLLIFSLWKHLRQMQLNGKGSQDPSTKVHIKAMQTVISFLFLFVIYI-LALILS
VWNS-----NQLQK--EPVQMLYDVILIMYPSIHSCILIW--------------------
-----------------------------------------------------
>r45Dyak
-----------------------M-------------ITLLSTIFSILGIIQFVLGNFAN
GFIALVNCIDWVKR-QKISSTDVIVTAMAVSRIVLFCVMLIHWYYILLHPALY-SLKVRT
IFHVAWTISNHYSTWLATSLSIFYLLKIVNFSSLTFLHLKWRVKSVVLMMLLGTS----F
ILVLQVVVISVSGTMQ---RSEFEGNFTQKTKLRDI------LWLSHVTLLILGNLTPFT
MFLISFLLPVFSLWKHLRKMQLNGKGFQDPCTKVHIKAMQTVISFLLLFAFYF-LVLIIS
IWRP-----KKLHE--EPFLLLFPTIEVIYPSVHSFILIWGNRKLTQAFLLFLWQLGCWL
KERK-------------------------------------------------
>r46cattle
-----------------------M-------------ITLLSTIFSILGIIQFVLGNFAN
GFIALVNCIDWVKR-QKISSTDVVVTAMAVSRIVLFCVMLIHWYYILLHPALY-SLKVRT
IFHVAWTISNHYSTWLATSLSIFYLLKIVNFSSLTFLHLKWRVKSVVLMMLLGTS----F
ILVLQVVVISVSGTMQ---RSEFEGNFTQKTKLRDI------LWLSHVTLLILGNLTPFT
MFLISFLLPIFSLCKHLRKMQLNGKGFQDPCTKVHIKAMQTVISFLLLFAFYF-LVLIIS
IWRP-----KKLHE--EPFLLLFPTVKVIYPSVHSFILIWGNRKLTQAFLLFLWQLGCWL
KERK-------------------------------------------------
>T2R20_frog
---------------------------------------MMSVVMTVILIVTWPCGTILN
SSIIAVYLSDWKKG-VKLGECDQISLSLGCTNILLQCFTTFGVASISYGLCFPLIEKVCL
VVFTVLWFPVYLSFWLTAGLSICYCLRLVNLSPKFCIPLKRRLSHIVPPLLVWSV-----
-AISSIIMVPMNWTIA---TDQNTTMMYYNISV-NV------VYL--ILETAFGICLPSI
VTSICIVLSLISLLRHIRRMKQNTQ-FGSPKLKNLIRACRTMFLLMALNFLFF-LIIFSS
MVPP-----YSGDTIWQTVMFSC---VMLNPSGQAVVLIFGNSKLLSAWSITLVPQG---
-----------------------------------------------------
>v1r3_chimp
-------------------------------------MASKDFAIGMI-LSQIMVGFLGN
FFLLYHYSFLHFTR-GMLQSTDLTLKHLTIANSLVILSKGIPQTMAAFGLKDSLSDIGCK
FVFYVHRVGRAVCTGNACLLSVFQVITISSSE-FRWAELKLHAHKYIRSFILVLC---WI
LNTLVNITVPLHVTGK---WNSINSTKTNDYGYCSGGSRSRIPHSLHIVLLSSLDVLCLG
LMTLASGSMVFILHRLKQQVQHIHGTNLSPRSSPESRVTQSILVLVSTLCYFTRSPPSLH
--MS-----LFPNPS-WWPLNASALITACFPTVSPFVLMSRHPRIPRLGSACCGRNPQFP
K---------------------LVR----------------------------
>V1r69_mus
-----------------------MGVATIKENQAQSKMDFRNLAIKIIFLSQTTIGILGN
FSLIIYY-VVYYIV-YTLKPTDLILMHLVTSNALMVLSTGVPHTMAAFGLTHLLNDLGCR
LILYIQRVGRSISIGSTCLLSIFQAIIISHRE-SCYKGQKVKSAKYV-GYSLSLL---WV
LHIFINFILFVDIFIK---RNRNNMTRDRDFKYCYIVGQNEFSDLLYVALVVCPEILSSV
LITYSSGSMIGILYRHKQRVQHIRSFHVSSRNSPEWRATQNILVLVSTFLGFYTLSSILQ
GCIA-----LQNNPN-WWLLNINRLTSMCFPTFAPFVLMSHYSFMPKLSLT---------
---------------------WIRNKTS-------------------------
>v1r_frog
-------------------------------------MDPYLLLKAIGFLLLVIIGIPGN
IFIMMQLTYLRITE-KKLQPNNIILIALAFVNLLVILSRIIPQLINALGVEELLDDTECK
FVIFTYRVNRAMSICLTSFLSCYQCILIAPNT-KLWSYLKHKVTQNV-VAMIFLF---WI
INIAIYPYFILNARTR---RNQTTSPYTLHLVYCDADFLNYMAYIANGTMYSFRDFIFVG
LMVLASSYIVFILLSHERSVKTIRSSDRTQQRSVEYRASRAVILLVALYVLLYGLDNCIW
IYTL-----TLSNVT-PNTNEIRIFLASSYSSLSPIVIKITNPKLQQKLFTCSKKRKHNC
K---------------------ING----------------------------
>V1r_Zebrafish
-------------------------------------MDLCVTIKGVSFLLQAGLGILAN
ALVLLAYAHIRLAE-ARLQPVDAILCHLALVDLLLLLTRGVPQTMTVFGMRNLLDDTGCK
VVIYTYRIARALSVCITCMLSVFQAVTVAPAAGPLLSGVKARLPQLL-APTFAAL---WF
INMAVCIAAPFFSVAP---RNGTVPPFTLNLGFCHVDFHDNLSYVLNGVAVSVRDFAFVG
AMLASSGFILLLLHRHRRQVRAVR---RSQGSTMETRAARTVLMLVILYSVFFGIDNVIW
```

```
IYML-----TVAQVP-PVVAHMRVFFSSCYASLSPFLIISSNRKLKARMVCATSEQERQA
E--------------------DGKNSSGKN---------------------
>V1R_alligator
------------------------------------MGAQFIFDILGFIFLDMVGIPGN
VVILYAFVHTLACH-RKVTLSEIILSKLALSNLLVILSQGIPFTLKIFWTQNVFNNLGCK
ITLYVYCVGRAMTICVTSLLGCFQCLLIIPFP-CRWLHWRESLLKNL-SSIMISL---WC
FNLFVCSTRLTYGSAQGDENSSTLGNYTELYNFCYVMFPTRYFYLGNRLVLVIRDLFFLG
AMTLSSCYLLYMFYQHGKQAKHLT---GLHVKHAEIQAAKAVVALLVLYLFSFGMDSIFW
IFTL-----CVAPGS-MRLVDAQMFFASCYSAIGPLVIILTNKKVQTGLKSTPNRREFAV
A-----------------------ESISKHIH-------------------
>V1r62_mus
---------------------------------------MEVLALQILLLCHVVVGTVGN
ILLFVHNFSPILTD-SRLRPIQVILINLAVANAFMLLLFAYSYDLTDIVPRKPPTDLKCK
LAYFFHLVARGTIMCSTCVLSTYQFVTLVPGT-WARVMFSEISPKVV-SYCCYSC---WL
FSVLNNAYMLMNVSGP---QKSHNDSDSKSNSICSI---SGVSVDMNILRFS-HDIIFLS
IMAWTSVSMVIHLHRHHQRMNHIHKVNQNNRGHAETRAAHTILMLVVTFVSLYILNCISI
LFYI-----SFVESR-LWLRYVTKLLALSFPTISPLLLIFRDSKGHCSLHIMSVWKST--
---------------------------------------------------
>V1r96_rat
-----------------------------MNKVN--ILPSDTNIKITLFSEVSVGISAN
SVLFFAHLCMFFEE-NRSKPIDLCIAFLSLTQLMLLVTMGL-IAADMFMSQGIWDSTTCR
SIIYFHRLLRGFNLCAACLLHILWTFTLSPRS-SCLTKFKHKSPHHI-SCAFFSL---CV
LYMLFSSHLFVLIIAT---SNLTSDHFMYVTQSCSILPMSYSRTTMFSLVMVTREAFLIS
LMALFSGYMVTLLWRHKKQVQHLHSTSLSSKSSPQQRATRTILLLMSFFVVLYILDIVIF
QSRT-----KFKDGS-MF-YSLHIIVSHSYATISPFVFIFSDKRIIKFLGSMSGRIINIC
L-------------------FSDGYGP-----------------------
>v1r_RMacaq
-------------MWNIF----MLLFRLSKKIVNI-LYSSDTTLGILPVFQTGIGLMGN
SLLFIICMYIILFRPHQKNPLDVILI------------------------STFVDTCCK
TGLYIYKMTRDICVCTSSLLSMFQAVIISPCH-SKWA----------------------
----------CTIST---SKSTNVGLGYSLIYCKT----------NILYTTMQRYFKVP
CLLKSSFYMVTIFLRHQKTVFHIHSISLSPQSFPETEATRVVFLLVSCFVFFYGTNMCLS
TYIS-----STYENS-LILENSTSFVSSSYPIICTLMLINYDNRVSRLTCAILNMITSPC
LPHSF---------------LLHQRNPDKNYFNYKRM--HF----------
>T2R_alligator
----------------------M-------------VFDPAYIAAIIIYLSALIGTVGN
LVILFAFISNAIRH-KVLQPLDKIIINMALVNLLLCCYKEIP-GLLFFTNIKIFGEQACR
VLLYTYHTLRLISIWSVENLSFLHLIKIQRPN-HRWSKFIYRHQGLYVNSILVGC---WI
FSILFHIPYLQYDEEM--EKNNNRTIIYLGATSCMS-STGKFITKFTTYSSVSLDLIVII
LVIILNVFIIDLLCKHRRKVRAVSTVD-SGWNKHTAQAAKILLSLLSIYVVCWISSDMVW
IAIVSGLIESNFEHS-IL-NSYYGLLSSIYYSASSYVVVFGYRKVREYLNEAC-------
---WC---------------LKCKKSMVVQTVE-----------------
```

Restored Pseudogenes

```
>BonoboPs8_restored
MITLLPIIFSILVVVTFVLGNFANGLLVLVNSIEWVKRQKISFADQILTALAVSRVGLLWVILLHWYATVLNPGSYSLGVRITTINAWAVTNHFSIW
VATSLSIFYLLKIANFFNFIFLHLKRRIKSVIPVILLGSLLFLVCHLVVVNMDESMWTKEYEGNVSWEIKLSDLTHLSDMTVTTLANLIPFTLSLLS
FLLLICSLCKHLKKMQLHGKGSPDSNTKVHIKALQTVTSFLLLFAVYFLSLITSIWNFRRRLQNEPVLMLCQTTAIIYPSFHSFILIWGSKKLKQTF
LLILCQIKC
>BOrangPs8_restored
TITFLPIIFSILEVVTFVLGNFANGFIALVNSIEWVKRQKISFADQILTALAVSRMGLLWVILLHWNATVLNPGLHSLGVRITTINAWAVTNHFRIW
IATSLSIFYLLKIANFSNFIFLHLKRRIKSVIPVILLGSLLFLVCHIVVVNMDESMWTKEYEGNVSWEIKLSDPTHLSEMTLTMLANLIPFTLSLLS
FLLLICSLSKHLKKMQLHGKGSPDSNTKVHIKVLQMVISFLLLFAVYFLSPITSIWKFRRRLQNEPVLMLCQTTAIIYPSFHSFILIWGSKKLKQTF
LLILCQIKC
>SlutungT2R15p_restored
GNFANGFIALVNSIEWVNRQKISSADQFLTALAVSRVGLHWVILLLWYATVLKPDSYSLAVRITTTNAWAVTNHFSIWVATSLSIFYLFKIANFSNF
IFLHLKRRIKSVIPVILLGSLLFLVCHLVVVNMDGSMWTKEYHGNVSWEIILSDSTHLSDMTVTTLANLIPFNLSLISFLLLICSLNKHLKKMQLHG
KGSPDPNTKVHIKALQTVISFLLLLAVYFLSQITSIWNLRRRLQKEPVLLLCQTTAIIYPSFHSFILIWGSKKLKQTFLLILCQIKC
>RmacaqR44p_restored
MITFLPIICSILVVVTFIIGNFANGFIALVNSTEWVKRQKISFADQILTALAVSRIGLLWVLLLHWYSIVLNPAFYRVEVRITTYNVWAVTSHLSNW
LATSLGIFYLLKVANFSSLIFLHLKRRVKSVILVMLLGPLVFLPCHLFVINMNEIVQTKEYERNMTWKIKLKRTMFLSDTAITMVANLVPFTLTLIS
FLLLICSLCKHLKKMQLHGRGSQDPNAKVHIKALQTVISFLLLCAIYFVFVTISVWSFQTLDNNPVFMFCQAITFSYPSAHPFILIWGNKKLKQTFL
SVLWNVRYW
VKGQKXSSP
```

>HbaboonT2R48p_restored
MYFLFIILSILLVFAFVLGNFANGFIALVNVIDWVKTRKISLADQILTALVIYRIGLLWAILLYWYATLFNSALCSSEVRIFASNISAIINHFSIWL
AASLRIFYLLKIANFSNLIFLHLQKRIKSVVQVMLLGPLVFLICNLAVVTTDEGVWTKEYEGNVTWKIKLKNAIHLSNLTISTLANLIPFTLTLICF
LLLICSLCKHLKKMQLHGKESQDLSTKVPIKSLQTVISFLMLFAIYFLCLITLTWSPWKQQSKLVFLLCQTLAIMYPSFHSFILIMGNRKLKQIFLS
VLWQVT
>BOrangT2R64p_restored
LVFAFVLRNFSNDFIALVNVIDWVKTRKISSADQILTAVVVSRIGLLWVILLHWYANVFNSALYSSEVGIVASNISAIINHFSIWLAASLSIFYLLK
IANFSNLIFLHLKKRIKSVVLVLLLGSLVFLICNLAVITTDDSVRTKEYEGNVTWKIKLRNAIHLSNLTVTMVANLIPFIVTLICFLLLICSLHKHL
KKMQLHSKGSQDLSTKVHIKALQTVISFLMLYAIYFLCLITLTWNLWTQQSKLVFMLCQTLVIMYPSFHSFFLIMGSRKLKQKFLS
>BOrangT2R68p_restored
LVFAFVLRNVASGFIALVNVNDWVKTQKISSTDQIVTALAFSRIGLLWIISLHWHATVFNSALYSLEVRIVPSNVLAIINHFSIWLATSLSIFYLLK
IANFSNFIFLQLKKRIKSVLLVILLGSLVFLICNLAVVTMDESVWTKEYEGNVTWKIKLRNAIHLSNMTVTMLANFTPFILTLISFLLLVCSPCKHL
KMLQLHGKGSQDLNTKDHIKPLQTVISFLMLFAIYFLCLITSTWNPRTQQSNLVFLLYETLAIMYPSFHSFILIMRSRKLK
>CanisT2R44p_restored
MLPLLQSIFSILVMTEFVLGNFANGFIVLVNYIAWVKRQKISSADQILTGLAVSRIGLLWVILINWYATLLNPALYSLEVRLPVHIAWTASNHFSIW
LATSLSVFYLFKIANFSNLIFLRLKWRVKSVVFVMLLGSLFFLVFSCCNGKRIEQMQMKEYEGNITRQTKLRDIAQLMNMTVFTLMNFVPFAISLTS
FLLLIFSLWKHLKKMRSSGKRSQGSSTKVHIRAQTVISFLSLLVCYFLTLIAIVWSSNRLQNGLFFMLCQVFAYAYPSSHSFILIWGNKKLREAFLS
VLYQVKYWL
KDQKLSTQ
>BOrangPs2_restored
MVYFLLIILSILVVFAFVLRNFSNDFIALVNVIDWVKTRKISSADQILTAVVVSRIGLLWVILLHWYANVFNSALYSSEVGIVASNISAIINHFSIW
LAASLSIFYLLKIANFSNLIFLHLKKRIKSVVLVLLLGSLVFLICNLAVITMDDSVRTKEYEGNVTWKIKLRNAIHLSNLTVTMVANLIPFILTLIC
FLLLICSLHKHLKKMQLHSKGSQDLSTKVHIKALQTVISFLMLYAIYFLCLITLTWNLWTQQSKLVFMLCQTLVIMYPSFHSFFLIMGSRKLKQKFL
SVLCQVTCLVKGQRPSTQ
>hT2R15p
MITFLPIIFSILVVVTFVLGNFANGFIVLVNSIEWVKRQKISFADQILTALAVSRVGLLWVILLHWYATVLNPGSYSLGVRITTINAWAVTNHFSIW
VATSLSIFYFLKIANFSNFIFLHLKRRIKSVIPVILLGSLLFLVCHLVVVNMDESMWTKEYEGNVSWEIKLSDPTHLSDMTVTTLANLIPFTLSLLS
FLLLICSLCKHLKKMQFHGKGSPDSNTKVHIKALQTVTSFLLLFAVYFLSLITSIWNFRRRLQNEPVLMLSQTTAIIYPSFHSFILIWGSKKLKQTF
LLILCQIKC
atgataacttttctacccatcatttttccattctagtagtggttacatttgttcttgggaattttgctaatggcttcatagtgttggtaaattcca
ttgagtgggtcaagagacaaaagatctcctttgctgaccaaattctcactgctctggcagtctccagagttggtttgctctgggtaatattattaca
ttggtatgcaactgttttgaatccaggttcatatagtttaggagtaagaattactactattaatgcctgggctgtaaccaaccatttcagcatctgg
gttgctactagcctcagcatatttatttcctcaagattgccaatttctccaactttatttttcttcacttaaaaaggagaattaagagtgtcattc
cagtgatactattggggtctttgttatttttggtttgtcatcttgttgtggtaaacatggatgagagtatgtggacaaaagaatatgaaggaaacgt
gagttgggagatcaaattgagtgatccgacgcacctttcagatatgactgtaaccacgcttgcaaacttaataccctttactctgtccctgttatct
tttctgctcttaatctgttctttgtgtaaacatctcaagaagatgcagttccatggcaaaggatctccagattccaacaccaaggtccacataaaag
ctttgcaaacggtgacctccttcctcttgttatttgctgtttactttctgtccctaatcacatcgatttggaattttaggaggaggctgcagaacga
acctgtcctcatgctcagccaaactactgcaattatatacccttcatttcattcattcatcctaatttggggaagcaagaagctgaaacagaccttt
cttttgatttgtgtcagattaagtgctga
>hT2R64p
MVYFLLIILSILVVFAFVLGNFSNGFIALVNVIDWVKTRKISSADQILTALVVSRIGLLWVILLHWYANVFNSALYSSEVGAVASNISAIINHFSIW
LAASLSIFYLLKIANFSNLIFLHLKKRIRSVVLVILLGPLVFLICNLAVITMDDSVWTKEYEGNVTWKIKLRNAIHLSNLTVSTLANLIPFILTLIC
FLLLICSLHKHLKKMQLHGKGSQDLSTKVHIKALQTVISFLMLYAIYFLYLITLTWNLRTQQNKLVFLLCQTLGIMYPSFHSFFLIMGSRKLKQTFL
SVLCQVTCLVKGQQPSTP
atggtatatttctgctcatcattttatcaattctggtagtgtttgcatttgttcttggaaatttttccaatggcttcatagctctagtaaatgtca
ttgactgggttaagacacgaaagatctcctcagctgaccaaatcctcactgctctggtggtctccagaattggtttactctgggtcatattattaca
ttggtatgcaaatgtgtttaattcagctttatatagttcagaagtaggagctgttgcttctaatatctcagcaataatcaaccatttcagcatctgg
cttgctgctagcctcagcatatttatttgctcaagattgccaatttctccaaccttatttttctccacctaaagaagagaattaggagtgttgttc
tggtgatactgttgggtcccttggtatttttgatttgtaatcttgctgtgataaccatggatgacagtgtgtggacaaaagaatatgaaggaaatgt
gacttggaagatcaaattgaggaatgcaatacacctttcaaacttgactgtaagcacactagcaaacctcatacccttcattctgaccctaatatgt
tttctgctgttaatctgttctctgcataaacatctcaagaagatgcagctccatggcaaaggatctcaagatctcagcaccaaggtccacataaaag
ctttgcaaactgtgatctccttcctcatgttatatgccatttactttctgtatctaatcacattaacctggaatcttcgaacacagcagaacaaact
tgtattcctgctttgccaaactcttggaatcatgtatccttcattccactcattcttcctgattatgggaagcaggaaactaaaacagacgtttctt
tcagttttatgtcaggtcacatgcttagtgaaaggacagcaaccctcaactccatag
>hT2R12p
MSSIWETLFIRILVVFIMGTVGNFIVLVNIIDIRNKVSLIDFILNCLAISRICFLITILATSFNIGYEKMPDSKNLAVSFDILWTGSSYFCLSCTTC
LSVFYFLKVANFSNPIFLWMKWKIHKVLLFIVLEATISFCTTSILKEIIINSLIERVTIKGNLTFNYMDTMHDFTSLFLLQMMFILPFVETLASILL
LILSLWSHTRQMKLHGIYSRDPSTEAHVKPIKAIISFLLLFIVHYFISIILTLACPLLDFVAARTFSSVLVFFHPSGHSFLLILRDSKLKQASLCVL
KKMKYAKKDIISHFYKHA
atgtcaagcatttgggagacactgtttataagaattcttgtagtgtaattcataatggggactgtgggaaattgattcattgtattggttaatatca
ttgactgaatcaggaactgaaaggtctccctgattgattttattctcaactgcttggccatctccaggatatgtttcctgtagataacaattttagc
tacctctttcaatataggctatgagaaaatgcctgattctaagaatcttgcagtaagttttgacattctctggacaggatccagctatttctgcctg
tcctgtaccacttgcctcagtgtcttctatttcctcaaggtagccaacttctccaatcccatttcctctggatgaaatggaaaattcacaaggtgc
ttctctttattgtactagaggcaacgatctctttctgcacaacttccattctgaaggaaataataattaatagtttaatctaagaacgggtaacaat
aaaaggcaacttgacatttaattatatggataccatgcatgatttcacttctctgtttctccttcagatgatgttcatccttccttttgtggaaaca
ctggcttccattcttctcttaatcctctccttatggagccacaccaggcagatgaagctacatggtatttattccagggatcccagcacagaagccc

```
atgtaaaacctataaaagctataatttcatttctactcctctttattgtgcattatttcatcagtatcatactaacattggcctgtcctcttctaga
cttcgttgcggcaaggacttttagtagtgtgctggtattttccatccatctggccattcatttcttctaattttacgggacagcaaactgaagcaa
gcttctctctgtgtcctgaagaagatgaagtatgccaaaaggacataatctctcattttataaacatgcctga
>hT2R18p
MFVGINIFFLVVATRGLVLGMLGNGLIGLVNCIEWAKSWKVSSADFILTSLAIVRIIRLYLILFDSFIMVLSPHLYTIRKLVKLFTILWALINQLSI
FATCLSIFYLLKIANFSHSLFLWLKWRMNGMIVMLLILSLFLLIFDSLVLEIFIDISLNIIDKSNLTLYLDESKTLYDKLSILKTLLSLTYVIPFLL
TLTSLLLLFISLVRHTKNLQLNSLGSRDSSTEAHKRAMKMVIAFLLLFIINFISTLIGDWIFLEVENYQVMMFIMMILLAFPSGHSFIIILGNNKLR
QSSLRLLWHLKFSLKKAKPLTS
atgttcgttggaattaatatttctttctggtggtggcaacaagaggacttgtcttaggaatgctgggaaacgggctcattggactggtaaactgca
ttgagtgggccaagagttggaaggtctcatcagctgatttcatcctcaccagcttggctatagtcagaatcattcgactgtatttaatactatttga
ttcatttataatggtattgtcccctcatctatataccatccgtaaactagtaaaactgtttactattctttgggcattaattaatcagttaagtatc
tagtttgccacctgcctaagcattttctacttgcttaagatagccaatttctcccactcccttttcctctggctgaagtggagaatgaacggaatga
ttgttatgcttcttatattgtctttgttcttactgattttgacagtttagtgctagaaatatttattgatatctcactcaatataatagataaaag
taatctgactttatatttagatgaaagtaaaactctctatgataaactctctattttaaaaactcttctcagcttgacatacgttattccctttctt
ctgactctgacctctttgctcctttatttatatccttagtgagacacaccaagaatttgcagctcaactctctgggctcaagggactccagcacag
aggcccataaaagggccatgaaaatggtgatagccttcctcctcctttttattattaactttatttccactttaataggagattggatcttccttga
ggtagagaattatcaggtcatgatgtttattatgatgattttacttgcctttccctcaggccactcatttattataatttgggaaacaacaagcta
agacagagctccttgagactactgtggcatcttaaattctctctgaaaaaagcaaaacctttaacttcatag
>hT2R63p_segment
CLSNNQAFQHLGVTSLSIFHLLKTANFSNLIFLHLKKRIKNVGLVMLLGPLVFFICNLALITTGESVWTKEYEGNLSWMIKLRNAIQLSNLTVTMPA
NVTPCTLTLISFLLLIYSPCKHVKKMQLHGKGSQHLSTKVHIKALQTVISFLMLFAIYFLCLITSTWNPRTQQSKLVFLLYQTLGFMYLLFHSFILT
MGSRKPKQTFLSAL
atgatatgtttctgctcatcattttatcaattctggtagtgtttgcatttgttcttggaaatgttgccaatggcttcatagctctagtaggtgtcc
ttgagtgggttaagacacaaaagatctcatcagctgaccaaatttctcactgctctggtggtgtccagagttggtttactctgggtcatattattac
attggtatgcaactgtgtttaatttggcttcacatagattagaagtaagaatttttggttctaatgtctcagcaataaccaagcatttcagcatctg
ggtgttactagcctcagcatatttcatttgctcaagactgccaatttctccaaccttatttttctccacctaaagaaaaggattaagaatgttggtt
tggtgatgctgttggggcccttggtatttttcatttgtaatcttgctctgataaccacgggtgagagtgtgtggacaaaagaatatgaaggaaattt
gtcttggatgatcaaattgaggaatgcaatacagctttcaaacttgactgtaaccatgccagcaaacgtcacaccctgcactctgacactaatatct
tttctgctgttaatctattctccatgtaaacatgtcaagaagatgcagctccatggcaaaggatctcaacatctcagcaccaaggtgcacataaaag
ctttgcaaactgtgatctccttccttatgttatttgccatttactttctgtgtctaatcacatcaacttggaatcctaggactcagcagagcaaact
tgtattcctgctttaccaaactcttggattcatgtatcttttgttccactcattcatcctgactatgggaagtaggaagccaaaacagacctttctt
tcagctttgtga


Aligned hT2Rs
>hTAS2R1/1-299
----------------------ML-----------ESHLIIYFLLAVIQFLLGIFTNGIIVVVNGIDLIKHRKMAPLDLL
LSCLAVSRIFLQLFIFYVNVIVIFFIEF-----IMCSANCAILLFINELELWLATWLGVFYCAKVASVRHPLFIWLKMRI
SKLVPWMILGSLLYVSMICVFHSKYAGFMVPYF-----LRKFFSQN--ATIQKEDTLA---IQIFSFVAEFSVPLLIFLF
AVLLLIFSLGRHTRQMRNTVAGSRVPGRGAPISALLSILSFLILYFSHCMIKVFLSSLKFHI-RRFIFLFFILVIGIYPS
GHSLILILGNPKLKQNAKKFLLHSKCCQ-------------------------
>hTAS2R3/1-316
----------------------MM-----------GLTEGVFLILSGTQFTLGILVNCFIELVNGSSWFKTKRMSLSDFI
ITTLALLRIILLCIILTDSFLIEFSPNTH-DSGIIMQIIDVSWTFTNHLSIWLATCLGVLYCLKIASFSHPTFLWLKWRV
SRVMVWMLLGALLLSCGSTASLINEFKLYSVFR---GIEATRNVTE--HFRKKRSEYY---LIHVLGTLWYLPPLIVSLA
SYSLLIFSLGRHTRQMLQNGTSSRDPTTEAHKRAIRIILSFFFLFLLYFLAFLIASFGNFLPKTKMAKMIGEVMTMFYPA
GHSFILILGNSKLKQTFVVMLRCESGHLKPGS----------KGPIFS-----
>hTAS2R4/1-299
----------------------ML-----------RLFYFSAIIASVILNFVGIIMNLFITVVNCKTWVKSHRISSSDRI
LFSLGITRFLMLGLFLVNTIYFVSSNTE--RSVYLSAFFVLCFMFLDSSSVWFVTLLNILYCVKITNFQHSVFLLLKRNI
SPKIPRLLLACVLISAFTTCLYITLSQASPFP------ELVTTRNN--TSFNISEGIL---SLVVSLVLSSSLQFIINVT
SASLLIHSLRRHIQKMQKNATGFWNPQTEAHVGAMKLMVYFLILYIPYSVATLVQYLPFYAGMDMGTKSICLIFATLYSP
GHSVLIIITHPKLKTTAKKILCFKK----------------------------
>hTAS2R5/1-299
----------------------ML-----------SAGLGLLMLVAVVEFLIGLIGNGSLVVWSFREWIRKFNWSSYNLI
ILGLAGCRFLLQWLIILDLSLFPLFQ-----SSRWLRYLSIFWVLVSQASLWFATFLSVFYCKKITTFDRPAYLWLKQRA
YNLSLWCLLGYFIINLLLTVQIGLTFYHPPQ----------GNSS---IRYPFESWQY---LYAFQLNSGSYLPLVVFLV
SSGMLIVSLYTHHKKMKVHSAGRRDVRAKAHITALKSLGCFLLLHLVYIMASPFSITSKTYPPDLTSVFIWETLMAAYPS
LHSLILIMGIPRVKQTCQKILWKTVCARRCWGP--------------------
>hTAS2R7/1-318
----------------------MA-----------DKVQTTLLFLAVGEFSVGILGNAFIGLVNCMDWVKKRKIASIDLI
LTSLAISRICLLCVILLDCFILVLYPDVY-ATGKEMRIIDFFWTLTNHLSIWFATCLSIYYFFKIGNFFHPLFLWMKWRI
DRVISWILLGCVVLSVFISLPATENLNADFRFC--VKAKRKTNLTW--SCRVNKTQHA---STKLFLNLATLLPFCVCLM
SFFLLILSLRRHIRRMQLSATGCRDPSTEAHVRALKAVISFLLLFIAYYLSFLIATSSYFMPETELAVIFGESIALIYPS
SHSFILILGNNKLRHASLKVIWKVMSILKGRK----------FQQHKQI----
>hTAS2R8/1-309
```

```
----------------------MF-----------SPADNIFIILITGEFILGILGNGYIALVNWIDWIKKKKISTVDYI
LTNLVIARICLISVMVVNGIVIVLNPDVY-TKNKQQIVIFTFWTFANYLNMWITTCLNVFYFLKIASSSHPLFLWLKWKI
DMVVHWILLGCFAISLLVSLIAAIVLSCDYRFH--AIAKHKRNITE--MFHVSKIPYF---EPLTLFNLFAIVPFIVSLI
SFFLLVRSLWRHTKQIKLYATGSRDPSTEVHVRAIKTMTSFIFFFFLYYISSILMTFSYLMTKYKLAVEFGEIAAILYPL
GHSLILIVLNNKLRQTFVRMLTCRKIACMI-----------------------
>hTAS2R9/1-312
----------------------MP-----------SAIEAIYIILIAGELTIGIWGNGFIVLVNCIDWLKRRDISLIDII
LISLAISRICLLCVISLDGFFMLLFPGTY-GNSVLVSIVNVVWTFANNSSLWFTSCLSIFYLLKIANISHPFFFWLKLKI
NKVMLAILLGSFLISLIISVPKNDDMWYHLFK-----VSHEENITW--KFKVSKIPGT---FKQLTLNLGVMVPFILCLI
SFFLLLFSLVRHTKQIRLHATGFRDPSTEAHMRAIKAVIIFLLLLIVYYPVFLVMTSSALIPQGKLVLMIGDIVTVIFPS
SHSFILIMGNSKLREAFLKMLRFVKCFLRRRK----------PFVP-------
>hTAS2R10/1-307
----------------------ML-----------RVVEGIFIFVVVSESVFGVLGNGFIGLVNCIDCAKNKL-STIGFI
LTGLAISRIFLIWIIITDGFIQIFSPNIY-ASGNLIEYISYFWVIGNQSSMWFATSLSIFYFLKIANFSNYIFLWLKSRT
NMVLPFMIVFLLISSLLNFAYIAKILNDYK---------MKNDTVW--DLNMYKSEYF---IKQILLNLGVIFFFTLSLI
TCIFLIISLWRHNRQMQSNVTGLRDSNTEAHVKAMKVLISFIILFILyFIGMAIEISCFtVRENKLLLMFGmTTTAIYPW
GHSFILILGNSKLKQASLRVLQQLKCCEKRKN----------LRVT-------
>hTAS2R13/1-303
----------------------ME-----------SALPSIFTLVIIAEFIIGNLSNGFIVLINCIDWVSKRELSSVDKL
LIILAISRIGLIWEILVSWFLALHYLAIF-VSGTGLRIMIFSWIVSNHFNLWLATIFSIFYLLKIASFSSPAFLYLKWRV
NKVILMILLGTLVFLFLNLIQINMHIKDWLDRY-----ERNTTWNF--SMSDFETFSV---SVKFTMTMFSLTPFTVAFI
SFLLLIFSLQKHLQKMQLNYKGHRDPRTKVHTNALKIVISFLLFYASFFLCVLISWISELYQ-NTVIYMLCETIGVFSPS
SHSFLLILGNAKLRQAFLLVAAKVWAKR-------------------------
>hTAS2R14/1-317
----------------------MG-----------GVIKSIFTFVLIVEFIIGNLGNSFIALVNCIDWVKGRKISSVDRI
LTALAISRISLVWLIFGSWCVSVFFPALF-ATEKMFRMLTNIWTVINHFSVWLATGLGTFYFLKIANFSNSIFLYLKWRV
KKVVLVLLLVTSVFLFLNIALINIHINASING-------YRRNKTCSSDSSNFTRFSS---LIVLTSTVFIFIPFTLSLA
MFLLLIFSMWKHRKKMQHTVKISGDASTKAHRGV-KSVITFFLLYAIFSLSFFISVWTSERL-EENLIILSQVMGMAYPS
CHSCVLILGNKKLRQASLSVLLWLRYMFKDGE----------PSGHKEFRESS
>hTAS2R16/1-291
---------------------MIP-----------IQLTVFFMIIYVLESLTIIVQSSLIVAVLGREWLQVRRLMPVDMI
LISLGISRFCLQWASMLNNFCSYFNL------NYVLCNLTITWEFFNILTFWLNSLLTVFYCIKVSSFTHHIFLWLRWRI
LRLFPWILLGSLMITCVTIIPSAIGNYIQIQLL--TMEHLPRNSTV--TDKLE-NFHQ---YQFQAHTVALVIPFILFLA
STIFLM---ASLTKQIQHHSTGHCNPSMKARFTALRSLAVLFIVFTSYFLTILITIIGTLFD-KRCWLWVWEAFVYAFIL
MHSTSLMLSSPTLKRILKGKC--------------------------------
>hTAS2R38/1-333
-------------MLTLTRIRTVS-----------YEVRSTFLFISVLEFAVGFLTNAFVFLVNFWDVVKRQALSNSDCV
LLCLSISRLFLHGLLFLSAIQLTHFQKLSEPLNHSYQAIIMLWMIANQANLWLAACLSLLYCSKLIRFSHTFLICLASWV
SRKISQMLLGIILCSCICTVLCVWCFFSRPHFTVTTVLFMNNNTRL--NWQIKDLNLF---YSFLFCYLWSVPPFLLFLV
SSGMLTVSLGRHMRTMKVYTRNSRDPSLEAHIKALKSLVSFFCFFVISSCAAFISVPLLILWRDKIGVMVCVGIMAACPS
GHAAILISGNAKLRRAVMTILLWAQSSLKVRA----------DHKADSRTLC-
>hTAS2R39/1-338
MLGRCFPPDTKEKQQLRMTKLCDPAES-----ELSPFLITLILAVLLAEYLIGIIANGFIMAIHAAEWVQNKAVSTSGRI
LVFLSVSRIALQSLMMLEITISSTSLSFY-SEDAVYYAFKISFIFLNFCSLWFAAWLSFFYFVKIANFSYPLFLKLRWRI
TGLIPWLLWLSVFISFSHSMFCINICTVYCNNS---FPIHSSNSTK--KTYLSEINVV---GLAFFFNLGIVTPLIMFIL
TATLLLILSLKRHTLHMGSNATGSNDPSMEAHMGAIKAISYFLILYIFNAVALFIYLSNMFDI-NSLWNNLCQIIMAAYPA
SHSILLIQDNPGLRRAWKRLQLRLHLYPKEWTL--------------------
>hTAS2R40/1-323
-------------MATVNTDATDKD--------ISKFKVTFTLVVSGIECITGILGSGFITAIYGAEWARGKTLPTGDRI
MLMLSFSRLLLQIWMMLENIFSLLFRIVY-NQNSVYILFKVITVFLNHSNLWFAAWLKVFYCLRIANFNHPLFFLMKRKI
IVLMPWLLRLSVLVSLSFSFPLSRDVFNVYVNS--SIPIPSSNSTE--KKYFSETNMV---NLVFFYNMGIFVPLIMFIL
AATLLILSLKRHTLHMGSNATGSRDPSMKAHIGAIKATSYFLILYIFNAIALFLSTSNIFDT-YSSWNILCKIIMAAYPA
GHSVQLILGNPGLRRAWKRFQHQVPLYLKGQTL--------------------
>hTAS2R41/1-307
----------------------MQ-----------AALTAFFVLLFSLLSLLGIAANGFIVLVLGREWLRYGRLLPLDMI
LISLGASRFCLQLVGTVHNFYYSAQKVEY-SGGLGRQFFHLHWHFLNSATFWFCSWLSVLFCVKIANITHSTFLWLKWRF
LGWVPWLLLGSVLISFIITLLFFWVNYPVYQEF--LIRKFSGNMTY--KWNTR-IETY---YFPSLKLVIWSIPFSVFLV
SIMLLINSLRRHTQRMQHNGHSLQDPSTQAHTRALKSLISFLILYALSFLSLIIDAAKFISM-QNDFYWPWQIAVYLCIS
VHPFILIFSNLKLRSVFSQLLLLARGFWVA-----------------------
>hTAS2R42/1-314
----------------------MA-----------TELDKIFLILAIAEFIISMLGNVFIGLVNCSEGIKNQKVFSADFI
LTCLAISTIGQLLVILFDSFLVGLASHLY-TTYRLGKTVIMLWHMTNHLTTWLATCLSIFYFFKIAHFPHSLFLWLRWRM
NGMIVMLLILSLFLLIFDSLVLEIFIDISLNII------DKSNLTL--YLDESKTLYDKLSILKTLLSLTSFIPFSLFLT
SLLFLFLSLVRHTRNLKLSSLGSRDSSTEAHRRAMKMVMSFLFLFIVHFFSLQVANGIFFMLWNNKYIKFVMLALNAFPS
CHSFILILGNSKLRQTAVRLLWHLRNYTKTPN----------ALPL-------
>hTAS2R43/1-309
```

```
----------------------MI-----------TFLPIIFSSLVVVTFVIGNFANGFIALVNSIESFKRQKISFADQI
LTALAVSRVGLLWVLLLNWYSTVLNPAFN--SVEVRTTAYNIWAVINHFSNWLATTLSIFYLLKIANFSNFIFLHLKRRV
KSVILVMLLGPLLFLACHLFVINMNEIVRTKE-------FEGNMTW--KIKLKSAMYF---SNMTVTMVANLVPFTLTLL
SFMLLICSLCKHLKKMQLRGKGSQDPSTKVHIKALQTVISFLLLCAIYFLSIMISVWSFGSLENKPVFMFCKAIRFSYPS
IHPFILIWGNKKLKQTFLSVFWQMRYWVKGEK----------TSSP-------
>hTAS2R44/1-309
----------------------MT-----------TFIPIIFSSVVVVLFVIGNFANGFIALVNSIERVKRQKISFADQI
LTALAVSRVGLLWVLLLNWYSTVFNPAFY--SVEVRTTAYNVWAVTGHFSNWLATSLSIFYLLKIANFSNLIFLHLKRRV
KSVILVMLLGPLLFLACQLFVINMKEIVRTKE-------YEGNMTW--KIKLRSAVYL---SDATVTTLGNLVPFTLTLL
CFLLLICSLCKHLKKMQLHGKGSQDPSTKVHIKALQTVIFFLLLCAVYFLSIMISVWSFGSLENKPVFMFCKAIRFSYPS
IHPFILIWGNKKLKQTFLSVLRQVRYWVKGEK----------PSSP-------
>hTAS2R45/1-299
----------------------MI-----------TFLPIIFSILVVVTFVIGNFANGFIALVNSTEWVKRQKISFADQI
VTALAVSRVGLLWVLLLNWYSTVLNPAFC--SVELRTTAYNIWAVTGHFSNWPATSLSIFYLLKIANFSNLIFLRLKRRV
KSVILVVLLGPLLFLACHLFVVNMNQIVWTKE-------YEGNMTW--KIKLRRAMYL---SDTTVTMLANLVPFTVTLI
SFLLLVCSLCKHLKKMQLHGKGSQDPSTKVHIKVLQTVISFFLLRAIYFVSVIISVWSFKNLENKPVFMFCQAIGFSCSS
AHPFILIWGNKKLKQTYLSVLWQMRY--------------------------
>hTAS2R46/1-309
----------------------MI-----------TFLPIIFSILIVVTFVIGNFANGFIALVNSIEWFKRQKISFADQI
LTALAVSRVGLLWVLVLNWYATELNPAFN--SIEVRITAYNVWAVINHFSNWLATSLSIFYLLKIANFSNLIFLHLKRRV
KSVVLVILLGPLLFLVCHLFVINMNQIIWTKE-------YEGNMTW--KIKLRSAMYL---SNTTVTILANLVPFTLTLI
SFLLLICSLCKHLKKMQLHGKGSQDPSMKVHIKALQTVTSFLLLCAIYFLSIIMSVWSFESLENKPVFMFCEAIAFSYPS
THPFILIWGNKKLKQTFLSVLWHVRYWVKGEK----------PSSS-------
>hTAS2R47/1-319
----------------------MI-----------TFLPIIFSILIVVIFVIGNFANGFIALVNSIEWVKRQKISFVDQI
LTALAVSRVGLLWVLLLHWYATQLNPAFY--SVEVRITAYNVWAVTNHFSSWLATSLSMFYLLRIANFSNLIFLRIKRRV
KSVVLVILLGPLLFLVCHLFVINMDETVWTKE-------YEGNVTW--KIKLRSAMYH---SNMTLTMLANFVPLTLTLI
SFLLLICSLCKHLKKMQLHGKGSQDPSTKVHIKALQTVTSFLLLCAIYFLSMIISVCNLGRLEKQPVFMFCQAIIFSYPS
THPFILILGNKKLKQIFLSVLRHVRYWVKDRSLRLHRFTRAALCKG-------
>hTAS2R48/1-299
----------------------MM-----------CFLLIISSILVVFAFVLGNVANGFIALVNVIDWVNTRKISSAEQI
LTALVVSRIGLLWVMLFLWYATVFNSALY--GLEVRIVASNAWAVTNHFSMWLAASLSIFCLLKIANFSNLISLHLKKRI
KSVVLVILLGPLVFLICNLAVITMDERVWTKE-------YEGNVTW--KIKLRNAIHL---SSLTVTTLANLIPFTLSLI
CFLLLICSLCKHLKKMRLHSKGSQDPSTKVHIKALQTVTSFLMLFAIYFLCIITSTWNLRTQQSKLVLLLCQTVAIMYPS
FHSFILIMGSRKLKQTFLSVLWQMTR--------------------------
>hTAS2R49/1-309
----------------------MM-----------SFLHIVFSILVVVAFILGNFANGFIALINFIAWVKRQKISSADQI
IAALAVSRVGLLWVILLHWYSTVLNPTSS--NLKVIIFISNAWAVTNHFSIWLATSLSIFYLLKIVNFSRLIFHHLKRKA
KSVVLVIVLGSLFFLVCHLVMKHTYINVWTEE-------CEGNVTW--KIKLRNAMHL---SNLTVAMLANLIPFTLTLI
SFLLLIYSLCKHLKKMQLHGKGSQDPSTKIHIKALQTVTSFLILLAIYFLCLIISFWNFKMRPKEIVLMLCQAFGIIYPS
FHSFILIWGNKTLKQTFLSVLWQVTCWAKGQN----------QSTP-------
>hTAS2R50/1-299
----------------------MI-----------TFLYIFFSILIMVLFVLGNFANGFIALVNFIDWVKRKKISSADQI
LTALAVSRIGLLWALLLNWYLTVLNPAFY--SVELRITSYNAWVVTNHFSMWLAANLSIFYLLKIANFSNLLFLHLKRRV
RSVILVILLGTLIFLVCHLLVANMDESMWAEE-------YEGNMTG--KMKLRNTVHL---SYLTVTTLWSFIPFTLSLI
SFLMLICSLYKHLKKMQLHGEGSQDLSTKVHIKALQTLISFLLLCAIFFLFLIVSVWSPRRLRNDPVVMVSKAVGNIYLA
FDSFILIWRTKKLKHTFLLILCQIRC--------------------------
>hTAS2R60/1-318
----------------------MNGDHMVLGSSVTDKKAIILVTILLLLRLVAIAGNGFITAALGVEWVLRRMLLPCDKL
LVSLGASRFCLQSVVMGKTIYVFLHPMAF-PYNPVLQFLAFQWDFLNAATLWSSTWLSVFYCVKIATFTHPVFFWLKHKL
SGWLPWMLFSSVGLSSFTTILFFIGNHRMYQNYL-RNHLQPWNVTG--DSIRSYCEKF---YLFPLKMITWTMPTAVFFI
CMILLITSLGRHRKKALLTTSGFREPSVQAHIKALLALLSFAMLFISYFLSLVFSAAGIFPP-LDFKFWVWESVIYLCAA
VHPIILLFSNCRLRAVLKSRRSS-RCGTP-----------------------
```

Sequences for Selecton dN/dS analysis

hT2R10:

```
>AF227136.1hT2R10
ATGCTACGTGTAGTGGAAGGCATCTTCATTTTTGTTGTAGTTAGTGAGTCAGTGTTTGGGGTTTTGGGGA
ATGGATTTATTGGACTTGTAAACTGCATTGACTGTGCCAAGAATAAGTTATCTACGATTGGCTTTATTCT
CACCGGCTTAGCTATTTCAAGAATTTTTCTGATATGGATAATAATTACAGATGGATTTATACAGATATTC
TCTCCAAATATATATGCCTCCGGTAACCTAATTGAATATATTAGTTACTTTTGGGTAATTGGTAATCAAT
CAAGTATGTGGTTTGCCACCAGCCTCAGCATCTTCTATTTCCTGAAGATAGCAAATTTTTCCAACTACAT
ATTTCTCTGGTTGAAGAGCAGAACAAATATGGTTCTTCCCTTCATGATAGTATTCTTACTTATTTCATCG
TTACTTAATTTTGCATACATTGCGAAGATTCTTAATGATTATAAAACGAAGAATGACACAGTCTGGGATC
```

TCAACATGTATAAAAGTGAATACTTTATTAAACAGATTTTGCTAAATCTGGGAGTCATTTTCTTCTTTAC
ACTATCCCTAATTACATGTATTTTTTTAATCATTTCCCTTTGGAGACACAACAGGCAGATGCAATCGAAT
GTGACAGGATTGAGAGACTCCAACACAGAAGCTCATGTGAAGGCAATGAAAGTTTTGATATCTTTCATCA
TCCTCTTTATCTTGTATTTTATAGGCATGGCCATAGAAATATCATGTTTTACTGTGCGAGAAAACAAACT
GCTGCTTATGTTTGGAATGACAACCACAGCCATCTATCCCTGGGGTCACTCATTTATCTTAATTCTAGGA
AACAGCAAGCTAAAGCAAGCCTCTTTGAGGGTACTGCAGCAATTGAAGTGCTGTGAGAAAAGGAAAAATC
TCAGAGTCACATAG

>AY724882.1chimpT2R10
ATGCTACGTGTAGTGGAAGGCATCTTCATTTTTGTTGTAATTAGTGAGTCAGTATTTGGGGTTTTGGGGA
ACGGATTTATTGGACTTGTAAACTGCATTGACTGTGCCAAGAATAAGTTATCTACGATTGGCTTTATTCT
CACCGGCTTAGCTATTTCAAGAATTTTTCTGATATGGATAATAATTACAGATGGATTTATACAGATATTC
TCTCCAAATATATATGCCTCCAGTAACCTAATTGAATATATTAGTTACTTTTGGGTAATTGGTAATCAAT
CAAGTATGTGGTTTGCCACCAGCCTCAGCATCTTCTATTTCCTGAAGATAGCAAATTTTTCCAACTACAT
ATTTCTCTGGTTGAAGAGCAGAACAAATATGGTTCTTCCCTTCATGATAGTATTCTTACTTATTTCATCG
TTACTTAATTTTGCATACATTGCGAAGATTCTTAATGATTATAAAATGAAGAATGACACAGTCTGGGATC
TCAACATGTATAAAAGTGAATACTTTATTAAACAGATTTTGCTAAATCTGGGAGTCATTTTCTTCTTTAC
ACTATCCCTAATTACATGTGTTTTGTTAATCATTTCCCTTTGGAGACACAACAGGCAGATGCAATCGAAT
GTGACAGGATTGAGAGACTCCAACACAGAAGCTCATGTGAAGGCAATGAAAGTTTTGATATCTTTCATCA
TCCTCTTTATCTTGTATTTTATAGGCATGGCCATAGAAATATCATATTTTACTGTGCGAGAAAACAAACT
GCTGCTTATGTTTGGAATGACAACCACAGCCATCTATCCCTGGGGTCACTCATTTATCTTAATTCTAGGA
AACAGCAAGCTAAAGCAAGCCTCTTTGAGGGTACTGCAGCAATTGAAGTGCTGTGAGAAAAGGAAAAATC
TCAGAGTCACATAG
>AY724915.1gorillaT2R10
ATGCTRCGTGTAGTGGAAGGCATCTTCATTTTTGTTGTAATTAGTGAGTTAGTATTTGGGGTTTTGGGGA
ATGGATTTATTGGACTTGTAAACTGCATTGACTGTGCCAAGAATAAGTTATCTACGATTGGCTTTATTCT
CACCGGCTTAGCTATTTCAAGAATTTTTCTGATATGGATAATAATTACAGATGGATTTATACAGATATTC
TCTCCAGATATATATGCCTCCGGTAACCTAATTGAATATATTAGTTACTTTTGGGTAATTGGTAATCAAT
CAAGTATGTGGTTTGCCACCAGCCTCAGCATCTTCTATTTCCTGAAGATAGCAAATTTTTCCAACTACAT
ATTTCTCTGGTTGAAGAGCAGAACAAATATGGTTCTTCCCTTCATGATAGTATTCTTACTTATTTCATCG
TTACTTAATTTTGCACACATTGCGAAGATTCTTAATGATTATAAAATGAAGAATGACACAGTCTGGGATC
TCAACATGTATAAAAGTGAATACTTTATTAAACAGATTTTGCTAAATCTGGGAGTCATTTTCTTCTTTAC
ACTATCCCTAATTACATGTGTTTTTTTAATCATTTCCCTTTGGAGACACAACAGGCAGATGCAATCGAAT
GTGACAGGATTGAGAGACTCCAACACAGAAGCTCATGTGAAGGCAATGAAAGTTTTGATATCTTTCATCA
TCCTCTTTATCTTGTATTTTATAGGCATGGCCATAGAAATATCATGTTTTACTGTGCGAGAAAACAAACT
RCTGCTTATGTTTGGMATGACAACCACAGCCATCTATCCCTGGGGTCACTCATTTATCTTAATTCTAGGA
AACAGCAAGCTAAAGCAAGCCTCTTTGAGGGTACTGCAGCAATTGAAGTGCTGTGAGAAAAGGAAAAATC
TCAGAGTCACATAG
>AY724852.1bonoboT2R10
ATGCTACGTGTAGTGGAAGGCATCTTCATTTTTGTTGTAATTAGTGAGTCAGTATTTGGGGTTTTGGGGA
ACGGATTTATTGGACTTGTAAACTGCATTGACTGTGCCAAGAATAAGTTATCTACGATTGGCTTTATTCT
CACCGGCTTAGCTATTTCAAGAATTTTTCTGATATGGATAATAATTACAGATGGATTTATACAGATATTC
TCTCCAAATATATATGCCTCCAGTAACCTAATTGAATATATTAGTTACTTTTGGGTAATTGGTAATCAAT
CAAGTATGTGGTTTGCCACCAGCCTCAGCATCTTCTATTTCCTGAAGATAGCAAATTTTTCCAACTACAT
ATTTCTCTGGTTGAAGAGCAGAACAAATATGGTTCTTCCCTTCATGATAGTATTCTTACTTATTTCATCG
TTACTTAATTTTGCATACATTGCGAAGATTCTTAATGATTATAAAATGAAGAATGACACAGTCTGGGATC
TCAACATGTATAAAAGTGAATACTTTATTAAACAGATTTTGCTAAATCTGGGAGTCATTTTCTTCTTTAC
ACTATCCCTAATTACATGTGTTTTGTTAATCATTTCCCTTTGGAGACACAACAGGCAGATGCAATCGAAT
GTGACAGGATTGAGAGACTCCAACACAGAAGCTCATGTGAAGGCAATGAAAGTTTTGATATCTTTCATCA
TCCTCTTTATCTTGTATTTTATAGGCATGGCCATAGAAATATCATATTTTACTGTGCGAGAAAACAAACT
GCTGCTTATGTTTGGAATGACAACCACAGCCATCTATCCCTGGGGTCACTCATTTATCTTAATTCTAGGA
AACAGCAAGCTAAAGCAAGCCTCTTTGAGGGTACTGCAGCAATTGAAGTGCTGTGAGAAAAGGAAAAATC
TCAGAGTCACATAG
>AY724827.1HBaboonT2R10
ATGCTAAGTGTAGTGGAAGGCATCCTCATTTTGGTTGTAATTAGTGAGTCAGTATTTGGGGTTTTAGGGA
ATGGATTTATTGGACTTGTAAACTGCATTGACTGTGCCAAGAATAAGTTATCTACAATTGGCTTTATTCT
CACCGGCTTAGCTATTTCTAGAATTTTTCTGATATGGATAATAATTACAGATGGATTCATACAGATATTC
TCTCCAGATGTTTATGCCTCTGGTAACCTAATTGAATATATTAGTTACTTTTGGGTAATTACTAATCAAT
CAAGTATATGGTTTGCCACCAGCCTCAGTATCTTCTATTTCCTGAAGATAGCAAATTTTTCCAACTACAT
ATTTCTCTGGTTGAAGAGTAGAATAAATAGAGTTCTTCCCCTTCTGATGGGATTCTTACTTATTTCATGC
TTACTTAATTTTGCATATATTGTGAAGATTCTTAATGATCTTAAAATGAAGAATGACACAGTCTGGCGTC
TCAACATGTATAAAAGTGAATACTTTATTAAACAGCTTTTGCTAAATCTGGGAGTCATTTTCTTCTTTAC
ACTATCCCTAATTACAAGTGTTTTGTTGATCATTTCCCTTTGGAGACACAACAGGCAGATGCAATCGAAT
GTGACAGGACTGAGAGACTCCATCACTGAAGCTCATGTGAAGGCAATGAAAGTTTTAATATCTTTCATCA
TCCTCTTTATCTTGTATTTTATAGGCATAGCCATAGAAATATCGTATTTTACTGTGCCAGAAAACAAACT
GTTGCTTATATTTGGAATGACAACCACAGCCATCTATCCCTGGGGTCACTCATTTATCTTAATTCTAGGA

AACAGCAAGCTAAAGCAAGCCTCTTTGAGGGTACTACAGCAATTGAAGTGCTGTGAGGAAAGGAAAAATC
TCAGAGCCACATAG
>AY724977.1BOrangT2R10
ATGCTAAGTGTAGTGGAAGGCATCTTCATTTTTGTTGTAATTAGTGAGTCAGTATTTGGGGTTTTGGGGAATGGATTTAT
TGGACTTGTAAACTGCATTGACTGTGCCAAGAATAAGTTATCTACGATTGGCTTTATTCTCACCGGCTTAGCTATTTCAA
GAATTTTTCTGATATGGGTAATAATTACAGATGGATTTATACAGATATTCTCTCCAGATATATATGCCTCCGGTAACCTA
ATTGAATATATTAGTTACATTTGGGTAATTGGTAATCAATCAAGTATGTGGTTTGCCACCAGCCTCAGCATCTTCTATTT
CCTGAAGATAGCAAATTTTTCCAACTACATATTTCTCTGGTTGAAGAGTAGAACAAATATGGTTCTTCCCTTTATGATGG
CATTCTTACTTATTTCATCGTTACTTAATTTTGCACACATTGTGAAGATTCTTAATGATCACAAAATGAAGAATGACACA
GTCTGGCATCTCAACATGTATAAAAGTGAATACTTTATTAAACAGATTTTGCTAAATCTGGGAGTCATTTTCTTCTTTAC
ACTATCCCTAATTACATGCGTTTTGTTAATCATTTCCCTTTGGAGACACAACAGGCAGATGCAATCGAATGTGACAGGAT
TGAGAGACTCCAACACAGAAGCTCACGTGAAGGCAATGAAAGTTTTGATATCTTTCATCATCCTTTTTATCTTGTATTTT
ATAGGCATGGCCTTAGAAATATCACGTTTTACTGTGCCAGAAAACAAACTGCTGCTTATGTTTGGAATGACAACCACAGC
CATCTATCCCTGGGGTCACTCATTTATCTTAATTCTAGGAAACAGCAAGCTAAAGCAAGCCTCTTTGAGGGTACTACAGC
AATTGAAGTGCTGTGAGAAAAGGAAAAAATCTCAGAGTCACATA
>AY724827.1HbaboonT2R10
ATGCTAAGTGTAGTGGAAGGCATCCTCATTTTGGTTGTAATTAGTGAGTCAGTATTTGGGGTTTTAGGGAATGGATTTAT
TGGACTTGTAAACTGCATTGACTGTGCCAAGAATAAGTTATCTACAATTGGCTTTATTCTCACCGGCTTAGCTATTTCTA
GAATTTTTCTGATATGGATAATAATTACAGATGGATTCATACAGATATTCTCTCCAGATGTTTATGCCTCTGGTAACCTA
ATTGAATATATTAGTTACTTTTGGGTAATTACTAATCAATCAAGTATATGGTTTGCCACCAGCCTCAGTATCTTCTATTT
CCTGAAGATAGCAAATTTTTCCAACTACATATTTCTCTGGTTGAAGAGTAGAATAAATAGAGTTCTTCCCCTTCTGATGG
GATTCTTACTTATTTCATGCTTACTTAATTTTGCATATATTGTGAAGATTCTTAATGATCTTAAAATGAAGAATGACACA
GTCTGGCGTCTCAACATGTATAAAAGTGAATACTTTATTAAACAGCTTTTGCTAAATCTGGGAGTCATTTTCTTCTTTAC
ACTATCCCTAATTACAAGTGTTTTGTTGATCATTTCCCTTTGGAGACACAACAGGCAGATGCAATCGAATGTGACAGGAC
TGAGAGACTCCATCACTGAAGCTCATGTGAAGGCAATGAAAGTTTTAATATCTTTCATCATCCTCTTTATCTTGTATTTT
ATAGGCATAGCCATAGAAATATCGTATTTTACTGTGCCAGAAAACAAACTGTTGCTTATATTTGGAATGACAACCACAGC
CATCTATCCCTGGGGTCACTCATTTATCTTAATTCTAGGAAACAGCAAGCTAAAGCAAGCCTCTTTGAGGGTACTACAGC
AATTGAAGTGCTGTGAGGAAAGGAAAAATCTCAGAGCCACATAG
>KT426847.1fenecFoxT2R10
ATGCTAAGCATACTGGAAAGGCTCTCATTTTTTATAGCTGTTAGTGAATCAATACTGGGAGTTTTAGGGA
ATGGATTTATTGGACTTGTCAATTGTATTGACTGTGTGAAGAACAAAAAGATTTCTATGGTTGGCTTTAT
TCTCACTGGCTTAGCTACTTCCAGAATTTGTCTGATATTGATAATAATTACAGATGGATTTATAAAGATA
TTCTCTCCAGATATGTATTCCTCCGGTAACTTAATTGATTATATTAGTTACCTATGGGTAATTATCAATC
AATCAAGTATCTGGTTTGCCACCAGCCTCAGCATCTTCTATTTCCTGAAGATAGCAAATTTTTCCCACCA
CATTTTTCTCTGGCTGAAGGGTAGAATCAATAGCGTTCTTCCCCTTCTGATGGGATCCTTGTTTATTTCA
TGGTTATTTACTTTTCCACAAATTGTGAAGATTATTAATGATAATAGAATGAAGAGTAGAAATACAACCT
GGCAGCTCAACATGCAGAAAAGTGAATTCTTTACGAAGCAGATTTTACTCAACCTAGGAGTCATTCTTCT
CTTTACTCTATGCCTGATTACATGTTTCTTGCTAATCATTTCCCTTTGGAGACACAACAGGCACATGCAA
TTGAATGTCACTGGACTCCGAGACCCCAGTACAGAAGCACATGTGAAAGCAATGAAAATTTTGGTATCTT
TTATCATCCTCTTTATCTTGTATTTTACAGGCATTGCCATAGAAATATCATGTTTCATTCTGCCAGAAAA
CAAACTGTTGTTTATTTTTGGTATGATGACCACAGCCATCTATCCCTGGGGTCATTCATTTATCCTAATT
CTAGGAAACAGCAAGCTAAAGCAAGCTTCTTTGAAGACTCTGCAGCAACTCAAGTGCTGTGAGGCAAGGA
GACTGCTCACAGCTGCACAGATCCACGTGGGGGGAAATGGATGTTCCAGGAGAATAATCTAG
>KT426827.1redFoxT2R10
ATGCTAAGCATACTGAAGAGCCCTCTCATTTTTATAGCTGTTAGTGAATCAATACTGGGAGTTTTAGGGA
ATGGATTTATTGGACTTGTCAATTGTATTGACTGTGTGAAGAACAAAAAGTTTTCTATGGTTGGCTTTAT
TCTCACTGGCTTAGCTACTTCCAGAATTTGTCTGATATTGATAATAATTACAGATGGATTTATAAAGATA
TTCTCTCCAGATATGTATTCCTCTGGTAACTTAATTGATTATATTAGTTACCTATGGGTAATTATCAATC
AATCAAGTATCTGGTTTGCCACCAGCCTCAGCATCTTCTATTTCCTGAAGATAGCAAATTTTTCCCACCA
CATTTTTCTCTGGCTGAAGGGTAGAATCAATAGCGTTCTTCCCCTTCTGATGGGATCCTTGTTTATTTCA
TGGTTATTTACTTTTCCACAAATTGTGAAGATTATTAATGATAATAGAATGAAGAGTAGAAATACAACCT
GGCAGCTCAACATGCAGAAAAGTGAATTCTTTACTAAGCAGATTTTACTCAACCTAGGAGTCATTCTTCT
CTTTACTCTATGCCTGATTACATGTTTCTTGCTAATCATTTCCCTTTGGAGACACAACAGGCACATGCAA
TTGAATGTCACTGGACTCCGAGACCCCAGTACAGAAGCACATGTGAAAGCAATGAAAATTTTGGTATCTT
TTATCATCCTCTTTATCTTGTATTTTATAGGCATTGCCATAGAAATATCATGTTTCATTCTGCCAGAAAA
CAAACTGTTGTTTATTTTTGGTATGATGACCACAGCCATCTATCCCTGGGGTCATTCATTTATCCTAATT
CTAGGAAACAGCAAGCTAAAGCAAGCTTCTTTGAAGACTCTGCAGCAACTCAAGTGCTGTGAGGCAAGGA
GACTGCTCACAGCTGCACAGATCCATGTGGGGGGAAATGGATGTTCCAGGAGAATAATCTAG
>KT426807.1tibetanFoxT2R10
ATGCTAAGCATACTGGAAGGCCTCCTCATTTTTATAGCTGTTAGTGAATCAATACTGGGAGTTTTAGGGA
ATGGATTTATTGGACTTGTCAATTGTATTGACTGTGTGAAGAACAAAAAGTTTTCTATGGTTGGCTTTAT
TCTCACTGGCTTAGCTACTTCCAGAATTTGTCTGATATTGATAATAATTACAGATGGATTTATAAAGATA
TTCTCTCCAGATATGTATTCCTCTGGTAACTTAATTGATTATATTAGTTACCTATGGGTAATTATCAATC
AATCAAGTATCTGGTTTGCCACCAGCCTCAGCATCTTCTATTTCCTAAAGATAGCAAATTTTTCCCACCA
CATTTTTCTCTGGCTGAAGGGTAGAATCAATAGCGTTCTTCCCCTTCTGATGGGATCCTTGTTTATTTCA
TGGTTATTTACTTTTCCACAAATTGTGAAGATTATTAATGATAATAGAATGAAGAGTAGAAATACAACCT

GGCAGCTCAACATGCAGAAAAGTGAATTCTTTACTAAGCAGATTTTACTCAACCTAGGAGTCATTCTTCT
CTTTACTCTATGCCTGATTACATGTTTCTTGCTAATCATTTCCCTTTGGAGACACAACAGGCACATGCAA
TTGAATGTCACTGGACTCCGAGACCCCAGTACAGAAGCACATGTGAAAGCAATGAAAATTTTGGTATCTT
TTATCATCCTCTTTATCTTGTATTTTATAGGCATTGCCATAGAAATATCATGTTTCATTCTGCCAGAAAA
CAAACTGTTGTTTATTTTTGGTATGATGACCACAGCCATCTATCCCTGGGGTCATTCATTTATCCTAATT
CTAGGAAACAGCAAGCTAAAGCAAGCTTCTTTGAAGACTCTGCAGCAACTCAAGTGCTGTGAGGCAAGGA
GACTGCTCACAGCTGCACAGATCCATGTGGGGGGAAATGGATGTTCCAGGAGAATAATCTAG
>KT426787.1corsacFoxT2R10
ATGCTAAGCATACTGGAAGTCCTCCTCATTTTTATAGCTGTTAGTGAATCAATACTGGGAGTTTTAGGGA
ATGGATTTATTGGACTTGTCAATTGTATTGACTGTGTGAAGAACAAAAAGTTTTCTATGGTTGGCTTTAT
TCTCACTGGCTTAGCTACTTCCAGAATTTGTCTGATATTGATAATAATTACAGATGGATTTATAAAGATA
TTCTCTCCAGATATGTATTCCTCCGGTAACTTAATTGATTATATTAGTTACCTATGGGTAATTATCAATC
AATCAAGTATCTGGTTTGCCACCAGCCTCAGCATCTTCTATTTCCTGAAGATAGCAAATTTTTCCCACCA
CATTTTTCTCTGGCTGAAGGGTAGAATCAATAGCGTTCTTCCCCTTCTGATGGGATCCTTGTTTATTTCA
TGGTTATTTACTTTTCCACAAATTGTGAAGATTATTAATGATAATAGAATGAAGAGTAGAAATACAACCT
GGCAGCTCAACATGCAGAAAAGTGAATTCTTTACTAAGCAGATTTTACTCAACCTAGGAGTCATTCTTCT
CTTTACTCTATGCCTGATTACATGTTTCTTGCTAATCATTTCCCTTTGGAGACACAACAGGCACATGCAA
TTGAATGTCACTGGACTCCGAGACCCCAGTACAGAAGCACATGTGAAAGCAATGAAAATTTTGGTATCTT
TTATCATCCTCTTTATCTTGTATTTTATAGGCATTGCCATAGAAATATCATGTTTCATTCTGCCAGAAAA
CAAACTGTTGTTTATTTTTGGTATGATGACCACAGCCATCTATCCCTGGGGTCATTCATTTATCCTAATT
CTAGGAAACAGCAAGCTAAAGCAAGCTTCTTTGAAGACTCTGCAGCAACTCAAGTGCTGTGAGGCAAGGA
GACTGCTCACAGCTGCACAGATCCATGTGGGGGGAAATGGATGTTCCAGGAGAATAATCTAG
>KT426732.1manedWolfT2R10
ATGCTAAGCATACTGGAAGGCCTCCTCATTTTTATAGCTGTTAGTGAATCAATACTGGGAGTTTTAGGGA
ATGGATTTATTGGACTTGTCAATTGTATTGACTGTGTGAAGAACAAAAAGTTTTCTATGGTTGGCTTTAT
TCTCACTGGCTTAGCTACTTCCAGAATTTGTCTGATATTGATAATAATTACAGATGGATTTATAAAGATA
TTCTCTCCAGATATGTATTCCTCTGGTAACTTAATTGATTATATTAGTTACCTATGGGTAATTATCAATC
AATCAAGTATCTGGTTTGCCACCAGCCTCAGCATCTTCTATTTCCTGAAGATAGCAAATTTTTCCCACCA
CATTTTTCTCTGGCTGAAGGGTAGAATCAATAGCGTTCTTCCCCTTCTGATGGGATCCTTGTTTATTTCA
TGGTTATTTACTTTTCCACAAATTGTGAAGATTATTAATGATAATAGAATGAAGAGTAGAAATACAACCT
GGCAGCTCAACATGCAGAAAAGTGAATTCTTTACTAAGCAGATTTTACTCAACCTAGGAGTCATTCTTCT
CTTTACTCTATGCCTGATTACATGTTTCTTGCTAATCGTTTCCCTTTGGAGACACAACAGGCACATGCAA
TTGAATGTCACTGGACTCCGAGACCCCAGTACAGAAGCACATGTGAAAGCAATGAAAATTTTGGTATCTT
TTATCATCCTCTTTATCTTGTATTTTATAGGCATTGCCATAGAAATATCATGTTTCATTCTGCCAGAAAA
CAAACTGCTGTTTATTTTTGGTATGATGACCACAGCCATCTATCCCTGGGGTCATTCATTTATCCTAATT
CTAGGAAACAGCAAGCTAAAGCAAGCTTCTTTGAAGACCCTGCAGCAACTCAAGTGCTGTGAGGCAAGGA
GACTGCTCACAGCTGCACAGATCCATGTGGGGGGAAATGGATGTTCCAGGAGAATAATCTAG
>KT426711.1wolfT2R10
ATGCTAAGCATACTGGAAGGCCTCCTCATTTTTATAGCTGTTAGTGAATCAATACTTGGGAGTTTAGGGA
ATGGATTTATTGGACTTGTCAATTGTATTGACTGTGTGAAGAACAAAAAGTTTTCTATGGTTGGCTTTAT
TCTCACTGGCTTAGCTACTTCCAGAATTTGTCTGATATTGATAATAATTACAGATGGATTTATAAAGATA
TTCTCTCCAGATATGTATTCCTCTGGTAACTTAATTGATTATATTAGTTACCTATGGGTAATTATCAATC
AATCAAGTATCTGGTTTGCCACCAGCCTCAGCATCTTCTATTTCCTGAAGATAGCAAATTTTTCCCACCA
CATTTTTCTCTGGCTGAAGGGTAGAATCAATAGCGTTCTTCCCCTTCTGATGGGATCCTTGTTTATTTCA
TGGTTATTTACTTTTCCACAAATTGTGAAGATTATTAATGATAATAGAATGAAGAGTAGAAATACAACCT
GGCAGCTCAACATGCAGAAAAGTGAATTCTTTACTAAGCAGATTTTACTCAACCTAGGAGTCATTCTTCT
CTTTACTCTATGCCTGATTACATGTTTCTTGCTAATTGTTTCCCTTTGGAGACACAACAGGCACATGCAA
TTGAATGTCACTGGACTCCGAGACCCCAGTACAGAAGCACATGTGAAAGCAATGAAAATTTTGGTATCTT
TTATCATCCTCTTTATCTTGTATTTTATAGGCATTGCCATAGAAATATCATGTTTCATTCTGCCAGAAAA
CAAACTGCTGTTTATTTTTGGTATGATGACCACAGCCATCTATCCCTGGGGTCATTCATTTATCCTAATT
CTAGGAAACAGCAAGCTAAAGCAAGCTTCTTTGAAGACCCTGCAGCAACTCAAGTGCTGTGAGGCAAGGA
GACTGCTCACAGCTGCACAGATCCATGTGGGGGGAAATGGATGTTCCAGGAGAATAATCTAG
>NM_001145497.1dogT2R10
ATGCTAAGCATACTGGAAGGCCTCCTCATTTTTATAGCTGTTAGTGAATCAATACTGGGAGTTTTAGGGA
ATGGATTTATTGGACTTGTCAATTGTATTGACTGTGTGAAGAACAAAAAGTTTTCTATGGTTGGCTTTAT
TCTCACTGGCTTAGCTACTTCCAGAATTTGTCTGATATTGATAATAATTACAGATGGATTTATAAAGATA
TTCTCTCCAGATATGTATTCCTCTGGTAACTTAATTGATTATATTAGTTACCTATGGGTAATTATCAATC
AATCAAGTATCTGGTTTGCCACCAGCCTCAGCATCTTCTATTTCCTGAAGATAGCAAATTTTTCCCACCA
CATTTTTCTCTGGCTGAAGGGTAGAATCAATAGCGTTCTTCCCCTTCTGATGGGATCCTTGTTTATTTCA
TGGTTATTTACTTTTCCACAAATTGTGAAGATTATTAATGATAATAGAATGAAGAGTAGAAATACAACCT
GGCAGCTCAACATGCAGAAAAGTGAATTCTTTACTAAGCAGATTTTACTCAACCTAGGAGTCATTCTTCT
CTTTACTCTATGCCTGATTACATGTTTCTTGCTAATCGTTTCCCTTTGGAGACACAACAGGCACATGCAA
TTGAATGTCACTGGACTCCGAGACCCCAGTACAGAAGCACATGTGAAAGCAATGAAAATTTTGGTATCTT
TTATCATCCTCTTTATCTTGTATTTTATAGGCATTGCCATAGAAATATCATGTTTCATTCTGCCAGAAAA
CAAACTGCTGTTTATTTTTGGTATGATGACCACAGCCATCTATCCCTGGGGTCATTCATTTATCCTAATT
CTAGGAAACAGCAAGCTAAAGCAAGCTTCTTTGAAGACCCTGCAGCAACTCAAGTGCGAGGCAAGGAGAC

TGCTCACAGCTGCACAGATCCATGTGGGGGAAATGGATGTTCCAGGAGAATAATCTAG
>XM_003126498.1pigT2R10
ATGCTAAGTATAGTAGAAAGCCTCCTCATTTTTATATCAGTTAGTCAGTCAATATTGGGGTTTTTAGGGA
ATGGATTTATTGGACTTGTAACCTGCATTGACTGTGTGAAAAACAAGAACATCTCTATGATCAGCTTTAT
TCTCACTGGCTTAGCTACTTCAAGAATTTGTCTGATATGGTTAATAATTATAGATGGATTTATAAAGATA
TTCTTTCCAGATTTATATATTTCTGGTAAACTAACTGAATACATTAGTTACTCATGGGTAATTGTCAATC
ATTCAAGTATCTGGTTTGCCACCAGCCTCAGCATCTTCTATTTCCTGAAGATAGCAAATTTTTCCCACCA
CATTTTTCTTTGGTTGAAGGGTAAAATCAATAGGGTTCTTCTCATTCTGATGGGATACTTGTTTATTTCA
TGGTTATTTACTTTTCCACAAGTTGTGAAGATTATTAGTGACAGTAAAAAGAAGAATGGAAGTTCATTCT
GGCCACTCAACATGCATAAACTTGAATACTTTATGAGCCAGTTTTTGCTCAATCTGGGTGTCATTCTCCT
CTTTATACTATGCATGATTACATGTTTCTTATTGATCATTTCTCTTTGGAGGCACAACAGGCAGATGCAA
TCGAATGCCACTGGATTCAGAGACCCCAGCACAGAAGCACATATTAAAGCAATGAAAATTGTGATATCTT
TTATCATCCTCTTTATCTTGTATTTTATAGGCGTTGCCATAGAAATATCATGTGGTACTCAGCCAGAAAA
CAAACTGCTGTTTATTTTTGGTATGATAACCACAGCCATTTTTCCTTGGGGTCACTCATTTATCCTAATT
CTAGGAAACAAGAAGCTAAAGCAAGCCTCTTTGAAGGTACTGAAGCAATTAAAGTGCTGGGGAAAAGAGA
AACTTCTCAGAACTCCATGA
>NM_001166677.1ratT2R110
ATGTTCTTACACACAATAAAGCAACGTGATATTTTTACTTTGATAATCATATTTTTTGTGGAAATAACAA
TGGGAATCTTAGGAAATGGATTCATAGCACTAGTGAATATTGTGGACTGGATCAAGAGAAGAAGGATTTC
TTCAGTGGATAAGATTCTCACTACCTTGGCCCTTACCAGACTCATTTATGCGTGGTCTATGCTCATTTTT
ATATTGTTATTCATACTGGGCCCGCATTTGATTATGAGATCAGAAATACTTACATCAATGGGTGTTATCT
GGGTGGTGAACAATCACTTCAGCATCTGGCTTGCTACATGCCTCGGTGTCTTTTATTTTCTCAAGATAGC
CAATTTTTCTAACTCTTTGTTTCTTTACCTAAAGTGGAGAGTTAAAAAAGTGGTTTTAATGATAATAGTA
GTGTCATTGATTTTCTTGATATTAAACATTTTTTCATTAGAGATTTATGATCATTTCTCAATTGATGTTT
ATGAAGGAAATATGTCTTATAGCTTGGGGGATTCAACACATTTTCCCAGAATTTTCTTATTCGCAAACTC
ATCTAAGGTCTTCTTAATCACCAATTCATCCCAGGTTTTCTTACCCATCAACTCACTCTTCATGCTCATA
CCCTTCACAGTTTCCCTGGTAGCTTTTTTCATGCTTATCTTCTCACTGTGGAAGCATCACAAGAAGATGG
AGGTCAATGCCAAAGGACCCAGAGATGCCAGCACCACGGCCCACATTAAAGCTTTACAAACTGGGCTCTC
CTTCCTGCTGCTGTATGCAATATACTTACTTTTTATTGTCATAGGAATTTTGAGTCATAAATTTATGGGG
GGGAAATTGATACTCATATTTGACCACATTTGTGCAATAGTTTTTCCTATAAGCCACTCATTTGTGCTGA
TTCTGGGAAATAGTAAACTGAGACGAAGCACTCTTTCTGTGCTGCGTTTTCTGAGGTGCCGATCCAAGCA
TATACACATCATGGATCCCTAA
>NM_199155.2mouseT2R110
ATGTTCTCACAGATAATAAGCACCAGTGATATTTTTACTTTTACAATAATATTATTTGTGGAATTAGTAA
TAGGAATTTTAGGAAATGGATTCATAGCACTAGTGAATATCATGGACTGGACCAAGAGAAGAAGCATTTC
ATCAGCGGATCAGATTCTCACTGCTTTGGCCATTACCAGATTTCTCTATGTGTGGTTTATGATCATTTGT
ATATTGTTATTCATGCTGTGCCCACATTTGCTTACAAGATCAGAAATAGTAACATCAATTGGTATTATTT
GGATAGTGAATAACCATTTCAGCGTTTGGCTTGCCACATGCCTCGGTGTCTTTTATTTTCTGAAGATAGC
CAATTTTTCTAACTCTTTGTTTCTTTACCTAAAGTGGAGAGTTAAAAAAGTAGTTTTAATGATAATACAG
GTATCAATGATTTTCTTGATTTTAAACCTGTTATCTCTAAGCATGTATGATCAGTTCTCAATTGATGTTT
ATGAAGGAAATACATCTTATAATTTAGGGGATTCAACCCCATTTCCCACAATTTCCTTATTCATCAATTC
ATCAAAAGTTTTCGTAATCACCAACTCATCCCATATTTTCTTACCCATCAACTCCCTGTTCATGCTCATA
CCCTTCACAGTGTCCCTGGTAGCCTTTCTCATGCTCATCTTCTCACTGTGGAAGCATCACAAAAAGATGC
AGGTCAATGCCAAACCACCTAGAGATGCCAGCACCATGGCCCACATTAAAGCCTTGCAAACAGGGTTCTC
CTTCCTGCTGCTGTATGCAGTATACTTACTTTTTATTGTCATAGGAATGTTGAGCCTTAGGTTGATAGGA
GGAAAATTAATACTTTTATTTGACCACATTTCTGGAATAGGTTTTCCTATAAGCCACTCATTTGTGCTGA
TTCTGGGAAATAACAAGCTGAGACAAGCCAGTCTTTCAGTGTTGCATTGTCTGAGGTGCCGATCCAAAGA
TATGGACACCATGGGTCCATAA
>AY737671.1RmacaqT2r10partial
TGTGCCAAGAATAAGTTATCTACAATTGGCTTTATTCTCACCAGCTTAGCTATTTCTAGAATTTTTCTGATATGGCTAAT
AATTACAGATGGATTCATACGGATATTCTCTCCAGATATATATGCTTCTGGTAACCTAATTGAATATATTAGTTACTTTT
GGGTAATTAGTAATCAATCAAGTATGTGGTTTGCCACCAGCCTCAGCATCTTCTATTTCCTGAAGATAGCAAATTTTTCC
AACTACATATTTCTCTGGTTGAAGAGTAGAACAAATAGGGTTCTTCCCCTTCTGATGGCATTCTTACTTATTTCATGCTT
ACTTAATTTTGCATATATTGCGAAGATTCTTAATGATTTTAAAATGAAGAATGACACAGTCTGGCGTCTCAACATATTTA
AAAATGAATACTTTGTTAAGCAGATTTTGCTAAATCTGGGAGTCATTTTCTTCTTTACACTATCCCTAATTACAAGTGTT
TTGTTGATCATTTCCCTTTGGAGACACAACAGGCAGATGCAATCAAATGTGACAGGACTGAGAGACTCCAACACTGAAGC
TCATGTGAAGGCAATGAAAGTTTTAATATCTTTCATCATCCTCTTTATCTTGTATTTTATAGGCATAGCCATAGAAATAT
CGTATTTTACTGTGCCAGAAAACAAACTGTTGCTTATGTTTGGAATGACA

>AB199083.1cebusT2R10partial
ATTGACTGTGCCAAGCATAAGTTCTCTACGATTGGCTTTATTCTAACTGGCTTAGCTATTTCTAGAATTTGTCTGATATG
GTTACTAATTGCAGATGGATTTATGCATATATTCTCTCCAGATATATATTTATCTAATGCATTCAACCTAGTTGAAGGTA
TTAGTTACTTTTGGTTAATTATTAATCAATCAAGTATATGGTTTGCCACAAGCCTCAGCATCTTCTATTTCCTGAAGATA
GCAAATTTTTCCAACACCATATTTCTCTGGTTGAAGAACAGAATAAACAGGATTCCTACCTTTCTGATGGGATGCTTACT
TATTTCATGGTTACTTAGTTTTCCACACACTGTGAAGATGTTTAATGATCATAAAATGAAGAATGCCACAGTCTGGCTTT
TCAACATGAATAGAAGTGAATACTTTATTAAACAGGTTTTGCTAAATCTGGGAGTCATTTTTTCCTTTACACTATCCCTA

ATTACATGTGTTTTGTTAATCATTTCCCTTTGGAGACACAACAGGCAGATGCAATTGAATCTGACAGGATTCAGGGATGT
CAACACAGAAGCTCATGTGAAGGCAATGAAAGTTTTGGTATCTTTCATCATCCTCTTTATCTTGTATTTTGTAAGCATTA
TCATAGAAATATCAATTTTTTCTGTGCCAGAAAACAAACTTCTGCTTATGTTTGGAATAACGACCAGTGTCGTCTATCCC
TGGGGTCATTCATTTATCTTAATTCTAGGA
>AB199082.1cmarmosetT2R10partial
ATTAACTGTGTCAAGCATAAGTTCTCTTTGATTGGGTTTATTCTAACTGGCTTAGCTATTTCTAGAATTTGTCTGATATG
GTTACTAATTACGGATGGATTTATGCATATATTCTCTCCAGATATATATTTCTCTAGTAACCTAATTGAATGTATTAGTT
ACTTTTGGTTAATTATTAATCAGTCAAGCATATGGTTTGCCACATGCCTCAGCATCTTCTATTTCCTGAAGATAGCAAAT
TTTTCCAACACCATATTTCTCTGGTTGAAGAGCAGAATAAACAGAATTCCTGCCTTTTTGTTGGGATGCTTACTTATTTC
ATGGTTATTTAGTTTTCCACACACTGTGAAGATGTTTAAAGATCATAAACTGAAGAATGCCACAGACTGGCATTTCAACG
TGAGTAAAAGTGAATACTTTATTAAACAGGTTTTGCTAAACCTGGGAGTCATCTTTTCATTTACACTATCCCTAACTACA
TGTGTTTTGTTAATCATTTCCCTTTGGAGACACAACAGGCAGATGCAATTGAATCTGACAGGATTCAGAGATGTCAACAC
AGAAGCTCATGTGAAGGCAATGAAAGTTTTGATATCTTTCATCATCCTCTCTATCTTGTATTTTGTAGGCATTATCATAG
AAATAACATTTTTTTCTGTGCCAGAACACAAACTTCTGCTTATGTTTGGAATAACAACCAGTGTCCTCTATCCCTGGAGT
CATTCATTTATTTTAATTCTAGGA
>AB199090.1lemurT2R10partial
ACTGACTGTGTGAAGAATAAGTTCTCTATGCTTAGCTTTATTCTCACTGGCCTAGCTATTTCTAGAATTTGTCTGATATG
GCTGATAATAATAAATGGATTTATGCAGTTATTCTCTCCAGATACGTATTCCTCTGGTAAACTAATTGAATATATTAGTT
ACATGTGGGCAATTATCAATCAATCAAGTATCTGGTTTGCCACTAGCCTTAACATCTTCTATTTCCTGAAGATAGCAAAT
TTTTCCCACTATGTATTTCTCTGGTTGAAAAGCAGAATCAATAGAATTCTTCTTCTTCTGGTGGGATCCTTGCTTATTTC
GTGGTTACTTACTTTTCCACAACTTGTGAAGATTTTTAATGATCATAAAATGAAGACTAGAAACACAACCTGCCCATTCA
AGACCGTTAAAAGTGAATACTTTATTCATCAGGTTTTGCTAAATCTCGGAGTCATTTTCTTCTTTATACTATCCTTAATT
ACATGTTTCTTGTTAATCATTTCCCTCTGGAGACACAATAGACAAATGCAATCGAATGCCACAGGATTCAGAGACCCCAG
CACAGAAGCTCATGTGAAGGCAATGAAAGTTTTGATATCTTTCATCATTCTCTTTATCTTGTATTTTATAGGCTTTGCCA
TACTAATGTCATATTTTGCTGCTCCAGAAAACAAACTGCTGGTGATTTTTGGAATGATAACCACAACCATCTATCCCTGG
GGTCACTCATTTATCCTAATCCTAGGA
>PantheraPardusT2R10
ATGCTAAGCATAGTGGAAGGCCTTCTCATTTTTATAGCAGTTAGTGAATCAGTACTGGGGGTTTTAGGGAATGGATTTATTGGACTTGTAAACTGTA
TGGACTGTGTGAAGAACAAAAAGTTTTCTATGATTGGCTTCATCCTCACCGGCTTAGCTACTTCCAGAATTTGTCTGATATTGATAGTAATTGTAGA
TGGATTTATAAAGATATTCTCTCCAGATATGTACTCTTCTGGTCACCTAATTGATTATATTAGTTACTTATGGATAATTATCAATCAATCAAACATC
TGGTTTGCCACCAGCCTCAGCACCTTCTACTTCCTGAAGATAGCAAATTTTTCCCACCACATGTTTCTCTGGTTGAAGGGTAGAATCAATTGGGTTC
TTCCCCTTCTGATGGGATCCTTGTTTATTTCATGGCTCTTTACGTTCCCTCAAATTGTGAAGATTCTTAGAGATAGTAAAGTGGTGAATGGAAACTC
AACCTGGCAGCTCAACATGCTGAAGAGTGAGTTCTTTACTAAGCAGATTTTGGTCAATGTAGGAGTCCTTCTCCTCTTCACGCTATTCCTGATTACA
TGTTTCCTGTTAATCATTTCCCTTTGGAGACACAGCAGGCGGATGCAATTGAATGTCACCGGATTCCAAGACCCCAGTACAGAAGCGCATATGAAAG
CCATGAAAGTTTTGATATCTTTTCTCATCCTCTTTATCTTGCATTTTATAGGCCTGGCCATAGAAATAGCATGCTTCACAATGCCAGAAAAAAAATT
GCTGTTTATTTTTGGTATGATGACCACAGTCTTGTACCCCTGGGGTCACTCATTTATCCTCATTCTCGGAAACAGCAAGCTAAAGCAAGCCTCTCTG
AGAGCATTGCAGCAGGTCAAGTGCTATTAA
>AcinonyxJubatusT2R10
ATGCTAAGCATAGTGGAAGGCCTTCTCATTTTTATAGCAGTTAGTGAATCAGTACTGGGGGTTTTAGGGAATGGATTTATTGGACTTGTAAACTGTA
TGGACTGTGTGAAGAACAAAAAGTTTTCTATGTTTGGCTTCATCCTCACTGGCTTAGCTACTTCCAGAATTTGTCTGATATTGATAGTAATTGCAGA
TGGATTTATAAAGATATTCTCTCCAGATATGTACTCTTCTGGTCACCTAATTGATTATATTAGCTACTTATGGATAATTATCAATCAATCAAACATC
TGGTTTGCCACCAGCCTCAGCACCTTCTATTTCCTGAAGATAGCAAATTTTTCCCACCACATGTTTCTCTGGTTGAAGGGTAGAATCAATTGGGTTC
TTCCCCTTCTGATGGGATCCTTGTTTATTTCATGGCTCTTTACATTCCCTCAAATTGTGAAGATTCTTAGTGACAGTAAAGTGGGGAATGGAAACAC
AACCTGGCAGCTCAACATGCCGAAGAGTGAGTTCTTTACTAAGCAGATTTTGGTCAACGTAGGAGTCCTTCTCCTCTTCACGCTATTCCTGATTACA
TGTTTCCTGTTAATCATTTCCCTTTGGAGACACAGCAGGCGGATGCAATTGAATGTCACTGGATTCCAAGACCCCAGTACAGAAGCGCATATGAAAG
CCATGAAAGTTTTGATATCTTTTATCATCCTCTTTATCTTGCATTTTATAGGCCTGGCCATAGAAATAGCATGCTTCACAATGCGAGAAAAAAAATT
GCTGTTTATTTTTGGTATGATGACCACAGTCTTGTACCCCTGGGGTCACTCATTTATCCTCATTCTCGGAAACAGCAAGCTAAAGCAAGCCTCTCTG
AGAGCATTGCAGCAGGTCAAGTGCTGTTAA
>HuntingdogT2R10
ATGCTAAGCATACTGGAAGGCCTCCTCATTTTTATAGCTGTTAGTGAATCAATACTGGGAGTTTTAGGGAATGGATTTATTGGACTTGTCAATTGTA
TTGACTGTGTGAAGAACAAAAAGTTTTCTATGGTTGGCTTTATTCTCACTGGCTTAGCTACTTCCAGAATTTGTCTGATATTGATAATAATTACAGA
TGGATTTATAAAGATATTCTCTCCAGATATGTATTCCTCTGGTAACTTAATTGATTATATTAGTTACCTATGGGTAATTATCAATCAATCAAGTATC
TGGTTTGCCACCAGCCTCAGCATCTTCTATTTCCTGAAGATAGCAAATTTTTCCCACCACATTTTTCTCTGGCTGAAGGGTAGAATCAATAGCGTTC
TTCCCCTTCTGATGGGATCCTTGTTTATTTCATGGTTATTTACTTTTCCACAAATTGTGAAGATTATTAATGATAATAGAATGAAGAGTAGAAATAC
AACCTGGCAGCTCAACATGCAGAAAAGTGAATTCTTTACTAAGCAGATTTTACTCAACCTAGGAGTCATTCTTCTCTTTACTCTATGCCTGATTACA
TGTTTCTTGCTAATCGTTTCCCTTTGGAGACACAACAGGCACATGCAATTGAATGTCACTGGACTCCGAGACCCCAGTACAGAAGCACATGTGAAAG
CAATGAAAATTTTGGTATCTTTTATCATCCTCTTTATCTTGTATTTTATAGGCATTGCCATAGAAATATCATGTTTCATTCTGCCAGAAAACAAACT
GCTGTTTATTTTTGGTATGATGACCACAGCCATCTATCCCTGGGGTCATTCATTTATCCTAATTCTAGGAAACAGCAAGCTAAAGCAAGCTTCTTTG
AAGACCCTGCAGCAACTCAAGTGCGAGGCAAGGAGACTGCTCACAGCTGCACAGATCCATGTGGGGGGAAATGGATGTTCCAGGAGAATAATCTAG

hT2R46:
>hT2R43
ATGATAACTTTTCTACCCATCATTTTTTCCAGTCTGGTAGTGGTTACATTTGTTATTGGA
AATTTTGCTAATGGCTTCATAGCACTGGTAAATTCCATTGAGTCGTTCAAGAGACAAAAG
ATCTCCTTTGCTGACCAAATTCTCACTGCTCTGGCGGTCTCCAGAGTTGGTTTGCTCTGG
GTATTATTATTAAACTGGTATTCAACTGTGTTGAATCCAGCTTTTAATAGTGTAGAAGTA

AGAACTACTGCTTATAATATCTGGGCAGTGATCAACCATTTCAGCAACTGGCTTGCTACT
ACCCTCAGCATATTTTATTTGCTCAAGATTGCCAATTTCTCCAACTTTATTTTTCTTCAC
TTAAAGAGGAGAGTTAAGAGTGTCATTCTGGTGATGTTGTTGGGGCCTTTGCTATTTTTG
GCTTGTCATCTTTTTGTGATAAACATGAATGAGATTGTGCGGACAAAAGAATTTGAAGGA
AACATGACTTGGAAGATCAAATTGAAGAGTGCAATGTACTTTTCAAATATGACTGTAACC
ATGGTAGCAAACTTAGTACCCTTCACTCTGACCCTACTATCTTTTATGCTGTTAATCTGT
TCTTTGTGTAAACATCTCAAGAAGATGCAGCTCCGTGGTAAAGGATCTCAAGATCCCAGC
ACGAAGGTCCACATAAAAGCTTTGCAAACTGTGATCTCCTTCCTCTTGTTATGTGCCATT
TACTTTCTGTCCATAATGATATCAGTTTGGAGTTTTGGAAGTCTGGAAAACAAACCTGTC
TTCATGTTCTGCAAAGCTATTAGATTCAGCTATCCTTCAATCCACCCATTCATCCTGATT
TGGGGAAACAAGAAGCTAAAGCAGACTTTTCTTTCAGTTTTTTGGCAAATGAGGTACTGG
GTGAAAGGAGAGAAGACTTCATCTCCATGA
>hT2R44
ATGACAACTTTTATACCCATCATTTTTTCCAGTGTGGTAGTGGTTCTATTTGTTATTGGA
AATTTTGCTAATGGCTTCATAGCATTGGTAAATTCCATTGAGCGGGTCAAGAGACAAAAG
ATCTCTTTTGCTGACCAGATTCTCACTGCTCTGGCGGTCTCCAGAGTTGGTTTGCTCTGG
GTATTATTATTAAATTGGTATTCAACTGTGTTTAATCCAGCTTTTTATAGTGTAGAAGTA
AGAACTACTGCTTATAATGTCTGGGCAGTAACCGGCCATTTCAGCAACTGGCTTGCTACT
AGCCTCAGCATATTTTATTTGCTCAAGATTGCCAATTTCTCCAACCTTATTTTTCTTCAC
TTAAAGAGGAGAGTTAAGAGTGTCATTCTGGTGATGCTGTTGGGGCCTTTACTATTTTTG
GCTTGTCAACTTTTTGTGATAAACATGAAAGAGATTGTACGGACAAAAGAATTTGAAGGA
AACATGACTTGGAAGATCAAATTGAAGAGTGCAATGTACTTTTCAATATGCACTGTAACC
ACCTTAGGAAACTTAGTACCCTTTACTCTGTCCCTGATATCTTTTCTGATGCTAATCTGT
TCTCTGTGTAAACATCTCAAGAAGATGCAGCTCCATGGAGAAGGATCGCAAGATCTCAGC
ACCAAGGTCCACATAAAAGCTTTGCAAACTCTGATCTCCTTCCTCTTGTTATGTGCCATT
TTCTTTCTATTCCTAATCGTTTCGGTTTGGAGTCCTAGGAGGCTGCGGAATGACCCGGTT
GTCATGGTTAGCAAGGCTGTTGGAAACATATATCTTGCATTCGACTCATTCATCCTAATT
TGGAGAACCAAGAAGCTAAAACACACCTTTCTTTTGATTTTGTGTCAGATTAGGTGCTGA
>hT2R45
ATGATAACTTTTCTGCCCATCATATTTTCCATTCTAGTAGTGGTTACATTTGTTATTGGA
AATTTTGCTAATGGCTTCATAGCGTTGGTAAATTCCACCGAGTGGGTGAAGAGACAAAAG
ATCTCCTTTGCTGACCAAATTGTCACTGCTCTGGCGGTCTCCAGAGTTGGTTTGCTCTGG
GTGTTATTATTAAATTGGTATTCAACTGTGTTGAATCCAGCTTTTTGTAGTGTAGAATTA
AGAACTACTGCTTATAATATCTGGGCAGTAACCGGCCATTTCAGCAACTGGCCTGCTACT
AGCCTCAGCATATTTTATTTGCTCAAGATTGCCAATTTCTCCAACCTTATTTTTCTTCGC
TTAAAGAGGAGAGTTAAGAGTGTCATTCTGGTGGTGCTGTTGGGGCCTTTGCTATTTTTG
GCTTGTCATCTTTTTGTGGTAAACATGAATCAGATTGTATGGACAAAAGAATATGAAGGA
AACATGACTTGGAAGATCAAATTGAGGCGTGCAATGTACCTTTCAGATACGACTGTAACC
ATGCTAGCAAACTTAGTACCCTTTACTGTAACCCTGATATCTTTTCTGCTGTTAGTCTGT
TCTCTGTGTAAACATCTCAAGAAGATGCAGCTCCATGGCAAAGGATCTCAAGATCCCAGT
ACCAAGGTCCACATAAAAGTTTTGCAAACTGTGATCTCCTTCTTCTTGTTACGTGCCATT
TACTTTGTGTCTGTAATAATATCAGTTTGGAGTTTTAAGAATCTGGAAAACAAACCTGTC
TTCATGTTCTGCCAAGCTATTGGATTCAGCTGTTCTTCAGCCCACCCGTTCATCCTGATT
TGGGGAAACAAGAAGCTAAAGCAGACTTATCTTTCAGTTTTGTGGCAAATGAGGTACTGA
>NM_176887.2hT2R46
ATGATAACTTTTCTGCCCATCATTTTTTCCATTCTAATAGTGGTTACATTTGTGATTGGA
AATTTTGCTAATGGCTTCATAGCATTGGTAAATTCCATTGAGTGGTTTAAGAGACAAAAG
ATCTCTTTTGCTGACCAAATTCTCACTGCTCTGGCAGTCTCCAGAGTTGGTTTACTCTGG
GTATTAGTATTAAATTGGTATGCAACTGAGTTGAATCCAGCTTTTAACAGTATAGAAGTA
AGAATTACTGCTTACAATGTCTGGGCAGTAATCAACCATTTCAGCAACTGGCTTGCTACT
AGCCTCAGCATATTTTATTTGCTCAAGATTGCCAATTTCTCCAACCTTATTTTTCTTCAC
TTAAAGAGGAGAGTTAAGAGTGTTGTTCTGGTGATACTATTGGGGCCTTTGCTATTTTTG
GTTTGTCATCTTTTTGTGATAAACATGAATCAGATTATATGGACAAAAGAATATGAAGGA
AACATGACTTGGAAGATCAAACTGAGGAGTGCAATGTACCTTTCAAATACAACGGTAACC
ATCCTAGCAAACTTAGTTCCCTTCACTCTGACCCTGATATCTTTTCTGCTGTTAATCTGT
TCTCTGTGTAAACATCTCAAAAAGATGCAGCTCCATGGCAAAGGATCTCAAGATCCCAGC
ATGAAGGTCCACATAAAAGCTTTGCAAACTGTGACCTCCTTCCTCTTGTTATGTGCCATT
TACTTTCTGTCCATAATCATGTCAGTTTGGAGTTTTGAGAGTCTGGAAAACAAACCTGTC
TTCATGTTCTGCGAAGCTATTGCATTCAGCTATCCTTCAACCCACCCATTCATCCTGATT
TGGGGAAACAAGAAGCTAAAGCAGACTTTTCTTTCAGTTTTGTGGCAAATGAGGTACTGA
>hT2R47
ATGATAACTTTTCTACCCATCATTTTTTCCATTCTGGTAGTGGTTACATTTGTTCTTGGA
AATTTTTCCAATGGCTTCATAGCTCTAGTAAATTCCATTGAGTGGGTCAAGACACGAAAG
ATCTCCTCAGCTGACCAAATCCTCACTGCTCTGGTGGTCTCCAGAGTTGGTTTACTCTGG
GTCATATTATTACATTGGTATGCAAATGTGTTTAATTCAGCTTTATATAGTTCAGAAGTA
GGAGCTGTTGCTTCTAATATCTCAGCAATAATCAACCATTTCAGCATCTGGCTTGCTACT

```
AGCCTCAGCATATTTTATTTGCTCAAGATTGCCAATTTCTCCAACCTTATTTTTCTCCAC
TTAAAGAAGAGAATTAGGAGTGTTGTTCTGGTGATACTGTTGGGTCCCTTGGTATTTTTG
ATTTGTAATCTTGCTGTGATAACCATGGATGACAGTGTGTGGACAAAAGAATATGAAGGA
AATGTGACTTGGAAGATCAAATTGAGGAATGCAATACACCTTTCAAATATGACTGTAAGC
ACACTAGCAAACCTCATACCCTTCATTCTGACCCTAATATGTTTTCTGCTGTTAATCTGT
TCTCTGTGTAAACATCTCAAGAAGATGCAGCTCCATGGCAAAGGATCTCAAGATCCCAGC
ACCAAGGTCCACATAAAAGCTTTGCAAACTGTGACCTCCTTTCTTCTGTTATGTGCCATT
TACTTTCTGTCCATGATCATATCAGTTTGTAATTTTGGGAGGCTGGAAAAGCAACCTGTC
TTCATGTTCTGCCAAGCTATTATATTCAGCTATCCTTCAACCCACCCATTCATCCTGATT
TTGGGAAACAAGAAGCTAAAGCAGATTTTTCTTTCAGTTTTGCGGCATGTGAGGTACTGG
GTGAAAGACAGAAGCCTTCGTCTCCATAGATTCACAAGAGGGGCATTGTGTGTCTTCTAG
>AY677150.1bonoboT2R46
ATGATAACTTTTCTGCCCATCATTTTTTCCATTCTAATAGTGGTTACATTTGTGATTGGAAATTTTGCTAATGGCTTCAT
AGCATTGGCAAATTCCATTGAGTGGTTCAAGAGACAAAAGATCTCTTTTGCTGACCAAATTCTCACTGCTCTGGCAGTCT
CCAGAGTTGGTTTACTCTGGGTATTATTATTAAATTGGTATGCAACTGAGTTGAATCCAGCTTTTTACAGTATAGAAGTA
AGAATTACTGCTTATAATCTCTGGGCAGTAATCAACCATTTCAGCAACTGGCTTGCTACTAGCCTCAGCATATTTTATTT
GCTCAAGATTGCCAATTTCTCCAACCTTATTTTTCTTCGCTTAAAGAGGAGAGTTAAGAGTGTTGTTCTGGTGATACTAT
TGGGGCCTTTGCTATTTTTGGTTTGTCATCTTTTTGTGATAAACATGAATCAGATTATATGGACAAAAGAATATGAAGGA
AACATGACTTGGAAGATCAAATTGAGGAGTGCAATGTACCTTTCAAATACAACGGTAACCATCCTAGCAAACTTAGTTCC
CTTCACTCTGACCCTGATATCTTTTCTGCTGTTAATCTGTTCTCTGTGTAAACATCTCAAGAAGATGCAGCTCCATGGCA
AAGGATCTCAAGATCCCAGCATGAAGGTCCACATAAAAGCTTTGCAAACTGTGACCTCCTTCCTCTTGTTATGTGCCATT
TACTTTCTGTCCATAATCATGTCAGTTTGGAGTTTTGAGAGTCTGGAAAACAAACCTGTCTTCATGTTCTGCGAAGCTAT
TACATTCAGCTATCCTTCAACCCACCCATTCATCCTGATTTGGGGAAACAAGAAGCTAAAGCAGACTTTTCTTTCAGTTT
TGTGGCATGTGAGGTACTGGGTGAAAGGAGAGAAGCCTTCATCTTCATAG
>NM_001046629.1cowTAS2R46
ATGATAACTCTACTATCAACCATTTTTTCCATCCTAGGAATAATACAATTTGTTCTGGGAAATTTTGCCA
ATGGCTTCATAGCCCTGGTGAACTGCATTGACTGGGTCAAGAGACAAAAGATCTCCTCAACTGATGTGGT
TGTCACTGCTATGGCAGTCTCCAGAATTGTTTTGTTCTGTGTAATGTTAATACATTGGTATTATATTTTG
CTTCATCCAGCTTTATATAGTTTAAAAGTAAGAACTATTTTTCATGTTGCCTGGACAATAAGCAATCATT
ATAGCACCTGGCTTGCTACTAGCCTCAGTATATTTTATTTGTTGAAGATAGTCAATTTCTCCAGCCTAAC
TTTTCTTCACCTGAAGTGGAGAGTTAAAAGTGTAGTTCTCATGATGCTTCTGGGAACTTCATTCATTTTG
GTTTTACAAGTTGTAGTTATAAGCGTAAGTGGGACTATGCAGAGAAGTGAATTTGAAGGAAACTTCACAC
AGAAGACCAAACTGAGGGATATTTTATGGCTTTCACATGTGACCCTGCTCATTCTAGGAAACCTCACACC
CTTTACTATGTTCTTAATATCTTTTCTGCTACCAATCTTTTCCCTGTGTAAACATCTCAGGAAGATGCAG
CTCAATGGCAAAGGATTCCAAGATCCCTGTACGAAGGTCCACATAAAAGCCATGCAAACTGTCATCTCCT
TTCTCTTGCTATTTGCCTTTTACTTTCTGGTTCTAATCATATCAATCTGGAGGCCTAAAAAACTGCATGA
GGAACCATTTCTCTTGCTTTTCCCAACAGTCAAAGTCATCTATCCTTCAGTCCACTCATTTATCCTGATT
TGGGGAAACAGAAAGTTAACACAGGCCTTTCTGTTGTTTCTGTGGCAGCTGGGGTGCTGGCTGAAAGAGA
GGAAATAG
>AY724877.1chimpT2R46
ATGATAACTTTTCTGCCCATCATTTTTTCCATTCTAATAGTGGTTACATTTGTGATTGGAAATTTTGCTA
ATGGCTTCATAGCATTGGCAAATTCCATTGAGTGGTTCAAGAGACAAAAGATCTCTTTTGCTGACCAAAT
TCTCACTGCTCTGGCAGTCTCCAGAGTTGGTTTACTCTGGGTATTATTATTAAATTGGTATGCAACTGAG
TTGAATCCAGCTTTTTACAGTATAGAAGTAAGAATTACTGCTTATAATCTCTGGGCAGTAATCAACCATT
TCAGCAACTGGCTTGCTACTAGCCTCAGCATATTTTATTTGCTCAAGATTGCCAATTTCTCCAACCTTAT
TTTTCTTTGCTTAAAGAGGAGAGTTAAGAGTGTTGTTCTGGTGATACTATTGGGGCCTTTGCTATTTTTG
GTTTGTCATCTTTTTGTGATAAACATGAATCAGATTATATGGACAAAAGAATATGAAGGAAACATGACTT
GGAAGATCAAATTGAGGAGTGCAATGTACCTTTCAAATACAACGGTAACCATCCTAGCAAACTTAGTTCC
CTTCACYCTGACCCTGATATCTTTTCTGCTGTTAATCTGTTCTCTGTGTAAACATCTCGAGAAGATGCAG
CTCCATGGCAAAGGATCTCAAGATCCCAGCATGAAGGTCCACATAAAAGCTTTGCAAACTGTGACCTCCT
TCCTCTTGTTATGTGCCATTTACTTTCTGTCCATAATCATGTCAGTTTGGAGTTTTGAGAGTCTGGAAAA
CAAACCTGTCTTCATGTTCTGCGAAGCTATTACATTCAGCTATCCTTCAACCCACCCATTCATCCTGATT
TGGGGAAACAAGAAGCTAAAGCAGACTTTTCTTTCAGTTTTGTGGCATGTGAGGTACTGGGTGAAAGGAG
AGAAGCCTTCATTTCCATAG
>AY724911.1gorillaT2R46
ATGATAACTTTTCTGCCCATCATTTTTTCCATTCTAATAGTGGTTACATTTGTGATTGGAAATTTTGCTAATGGCTTCAT
AGCATTGGCAAATTCCATTGAGTGGTTCAAGAGACAAAAGATCTCTTTTGCTGACCAAATTCTCACTGCTCTGGCAGTCT
CCAGAGTTGGTTTACTCTGGGTATTATTATTAAATTGGTATGCAACTGAGTTGAATCCAGCTTTTTACAGTATAGAAGTA
AGAATTACTGCTTATAATGTCTGGGCAGTAATCAGCCATTTCAGCAACTGGCTTGCTACTAGCCTCAGCATATTTTATTT
GCTCAAGATTGCCAATTTCTCCAACCTTATTTTTCTTCGCTTAAAGAGGAGAGTTAAGAGTGTTGTTCTGGTGATACTAT
TGGGGCCTTTGTTATTTTTGGTTTGTCATCTTTTTGTGATAAACATGAATCAGATTATATGGACAAAAGAATATGAAGGA
AACATGACTTGGAAGATCAAATTGAGGAGTGCAATGTACCTTTCAGATACAACGGTAACCATCCTAGCAAACTTAGTTCC
CTTCACTCTGACTCTGATATCTTTTCTGCTGTTAATCTGTTCTCTGTGTAAACATCTCAAGAAGATGCAGCTCCATGGCA
AAGGATCTCAAGATCCCAGCATGAAGGTCCACATAAAAGCTTTGCAAACTGTGACCTCCTTCCTCTTGTTATGTGCCATT
TACTTTCTGTCCGTAATCATGTCAGTTTGGAGTTTTGAGAGTCTGGAAAACAAACCTGTCTTCATGTTCTGTGAAGCTAT
TACATTCAGCTATCCTTCAACCCACCCATTCATCCTGATTTGGGGAAACAAGAAGCTAAAGCAGACTTTTCTTTCAGTTT
```

TGTGGCATGTGAGGTACTGGGTGAAAGGAGAGAAGCCTTCATCTTCATAG
>AY677161.1bonoboT2R66
ATGATAACTTTTCTGCCCATCATTTTTTCCATTCTAATAGTGGTTACATTTGTGATTGGAAATTTTGCTA
ATGGCTTCATAGCATTGGCAAATTCCATTGAGTGGTTCAAGAGACAAAAGATCTCTTTTGCTGACCAAAT
TCTCACTGCTCTGGCAGTCCCCAGAGTTGGTTTACTCTGGGTATTATTATTAAATTGGTATGCAACTGAG
TTGAATCCAGCTTTTTACAGTATAGAAGTAAGAATTACTGCTTATAATCTCTGGGCAGTAATCAACCATT
TCAGCAACTGGCTTGCTACTAGCCTCAGCATATTTTATTTGCTCAAGATTGCCAATTTCTCCAACCTTAT
TTTTCTTCGCTTAAAGAGGAGAGTTAAGAGTGTTGTTCTGGTGATACTGTTGGGGCCTTTGCTATTTTTG
GTTTGTCATCTTTTTGTGATAAACATGAATCAGATTATATGGACAAAAGAATATGAAGGAAACATGACTT
GGAAGATCAAATTGAGGAGTGCAATGTACCTTTCAAATACAACGGTAACCATCCTAGCAAACTTAGTTCC
CTTTACTGTAACCCTGATATCTTTTCTGCTGTTAGTCTGTTCTCTGTGTAAACATCTCAAGAAGATGCAA
CTCCATGGCAAAGGATCTCAAGATCCCAGTACCAAGGTCCACATAAAAGCTTTGCAAACTGTGATCTCCT
TCCTCTTGTTATGTGCCATTTACTTTGTGTCTGTAATAATATCAGTTTGGAGTTTTAAGAATCTGGAAAA
CAAACCTGTCTTCATGTTCTGCCAAGCTATTGGATTCAGCTGTTCTTCAGCCCACCCGTTCATCCTGATT
TGGGGAAACAAGAAGCTAAAGCAGCCTTTTCTTTCAGTTTTGTGGCAAATGAGGTACTGGGTGAAAGGAG
AGAAGCCTTCATCTTCATAG
>AB713290.1chimpTAS2R47haplotype1
ATGATAACTTTTCTGCCCATCATTTTTTCCATTCTAATAGTGGTTATATTTGTTATTGGAAATTTTGCTAATGGCTTCAT
AGCATTGGTAAATTCCATTGAGTGGGTCAAGAGACAAAAGATCTCCTTTGTTGACCAAATTCTCACTGCTCTGGCGGTCT
CCAGAGTTGGTTTGCTCTGGGTGTTATTACTACATTGGTATGCAACTCAGTTGAATCCAGCTTTTTATAGTGTAGAAGTA
AGAATTACTGTTTATAATGTCTGGGCAGTAACCAACCATTTCAGCAGCTGGCTTGCTACTAGCCTCAGCATGTTTTATTT
GCTCAAGATTGCCAATTTCTCCAACCTTATTTTTCTTCGCATAAAGAGGAGAGTTAAGAGTGTTGTTCTGGTGATACTGT
TGGGGCCTTTGCTATTTTTGGTTTGTCATCTTTTTGTGATAAACATGGATGAGACTATATGGACAAAAGAATATGAAGGA
AACATGACTTGGAAGATCAAATTGAGGAGTGCAATGTACCATTCAAATATGACTCTAACCATTCTAGCAAACTTTGTACC
CCTCACTCTGACCCTGATATCTTTTCTGCTGTTAATCTGTTCTCTGTGTAAACATCTCAAGAAGATGCAGCTCCATGGCA
AAGGATCTCAAGATCCCAGCACCAAGGTCCACATAAAAGCTTTGCAAACTGTGACCTCCTTCCTCTTGTTATGTGCCATT
TACTTTCTGTCCATGATCATATCAGTTTGTAATTTAGGGAGGCTGGAAAAGCAACCTGTCTTCATGTTCTGCCAAGCTAT
TATATTCAGCTATCCTTCAACCCACCCATTCATCCTGATTTTGGGAAACAAGAAGCTAAAGCAGATTTTTCTTTCAGTTT
TGTGGCATGTGAGGTACTGGGTGCAAGACAGAAGCCTTCGTCTCCATAGATTCACAAGAGGGGCATTGTGTGTCTTCTAG
>AB713339.1chimpTAS2R43haplotype5
ATGATAACTTTTCTACCCATCATTTTTTCCAGTCTGGTAGTGGTTACATTTGTTATTGGAAATTTTGCTAATGGCTTCAT
AGCATTGGTAAATTCCATTGAGTGGTTCAAGAGACAAAAGATCTCCTTTGCTGACCAAATTCTCACTGCTCTGGCGGTCT
CCAGAGTTGGTTTGCTCTGGGTATTATTATTAAACTGGTATTCAACTGTGTTGAATCCAGCTTTTAATAGTGTAGAAGTA
AGAACTACTGCTTATAATATCTGGGCAGTAATCAACCATTTCAGCAACTGGCTTGCTACTAGCCTCAGCATATTTTATTT
GCTCAAGATTGCCAATTTCTCCAACTTTATTTTTCTTCACTTAAAGAGGAGAGTTAAGAGTGTCATTCTGGTGATGTTGT
TGGGGCCTTTGCTATTTTTGGCTTGTCATCTTTTTGTGATAAACATGAATGAGATTGTGCGGACAAAAGAATTTGAAGGA
AACATGACTTGGAAGATCAAATTGAAGAGTGCAATGTACTTTTCAAATATGACTGTAACCATGGTAGCAAACTTAGTACC
CTTCACTCTGACCCTACTATCTTTTTTGCTGTTAATCTGTTCTTTGTGTAAACATCTCAAGAAGATGCAGCTCCATGGTA
AAGGATCTCAAGATCCCAGCACGAAGGTCCACATAAACGCTTTGCAAACTGTGATCTCCTTCCTCTTGTTATGTGCCATT
TACTTTCTGTCCATAATGATATCAGTTTGGAGTTTTGGAAGTCTGGAAAACAAACCTGTCTTCATGTTCTGCAAAGCTAT
TAGATTCAGCTATCCTTCAATCCACCCATTCATCCTGATTTGGGGAAACAAGAAGCTAAAGCAGACTTTTCTTTCAGTTT
TTTGGCAAATGAGGTACTGGGTGAAAGGAGAGAAGACTTCATCTCCATAG
>AY677151.1bonoboT2R47
ATGATAACTTTTCTGCCCATCATTTTTTCCATTCTAATAGTGGTTATATTTGTTATTGGAAATTTTGCTAATGGCTTCAT
AGCATTGGTAAATTCCATTGAGTGGGTCAAGAGACAAAAGATCTCCTTTGTTGACCAAATTCTCACTGCTCTGGCGGTCT
CCAGAGTTGGTTTGCTCTGGGTGTTATTACTACATTGGTATGCAACTCAGTTGAATCCAGCTTTTTATAGTGTAGAAGTA
AGAATTACTGTTTATAATGTCTGGGCAGTAACCAACCATTTCAGCAGCTGGCTTGCTACTAGCCTCAGCATGTTTTATTT
GCTCAAGATTGCCAATTTCTCCAACCTTATTTTTCTTCGCATAAAGAGGAGAGTTAAGAGTGTTGTTCTGGTGATACTGT
TGGGGCCTTTGCTATTTTTGGTTTGTCATCTTTTTGTGATAAACATGGATGAGACTATATGGACAAAAGAATATGAAGGA
AACATGACTTGGAAGATCAAATTGAAGAGTGCAATGTACCATTCAAATATGACTCTAACCATTCTAGCAAACTTTGTACC
CCTCACTCTGACCCTGATATCTTTTCTGCTGTTAATCTGTTCTCTGTGTAAACATCTCAAGAAGATGCAGCTCCATGGCA
AAGGATCTCAAGATCCCAGCACCAAGGTCCACATAAAAGCTTTGCAAACTGTGACCTCCTTCCTCTTGTTATGTGCCATT
TACTTTCTGTCCATGATCATATCAGTTTGTAATTTAGGGAGGCTGGAAAAGCAACCTGTCTTCATGTTCTGCCAAGCTAT
TATATTCAGCTATCCTTCAACCCACCCATTCATCCTGATTTTGGGAAACAAGAAGCTAAAGCAGATTTTTCTTTCAGTTT
TGTGGCATGTGAGGTACTGGGTGAAAGACCGAAGCCTTCGTCTCCAT
>AY724910.1gorillaT2R47
ATGATAACTTTTCTGCCCATCATTTTTTCCATTCTAATAGTGGTTATATTTGTGATTGGAAATTTTGCTAATGGCTTCAT
AGCATTGGTAAATTCCATTGAGTGGGTCAAGAGACAAAAGATCTCCTTTGTTGACCAAATTCTCACTGCTCTGGCGGTCT
CCAGAGTTGGTTTGCTCTGGGTGTTATTACTACATTGGTATGCAACTCAGTTGAATCCAGCTTTTTATAGTGTAGAAGTA
AGAATTACTGCTTATAATGTCTGGGCAGTAACCAACCATTTCAGCAGCTGGCTTGCTACTAGCCTCAGCATGTTTTATTT
GCTCAAGATTGCCAATTTCTCCAACCTTGTTTTTCTTCGCATAAAGAGGAGAGTTAAGAGTGTTGTTCTGGTGATACTGT
TGGGGCCTTTGCTATTTTTGGTTTGTCATCTTTTTGTGATAAACATGGATGAGACTATATGGACAAAAGAATATGAAGGA
AACATGACTTGGAAGATCAAATTGAGGAGTGCAATGTACCATTCAAATATGACTCTAACCATGCTAGCAAACTTTGTACC
CCTCACTCTGACACTGATATCTTTTCTGCTGTTAATCTGTTCTCTGTGTAAACATCTCAAGAAGATGCAGCTCCATGGCA
AAGGATCTCAAGATCCCAGCACCAAGGTCCACATAAAAGCTTTGCAAACTGTGACCTCCTTTCTCCTGTTATGTGCCATT
TACTTTCTGTCCATGATCATATCAGTTTGTAATTTTGGGAGGCTGGAAAAGCAACCTGTCTTCATGTTCTGCCAAGCTAT

TATATTCAGCTATCCTTCAACCCACCCATTCATCCTGATTTTGGGAAACAAGAAGCTAAAGCAGATTTTTCTTTCAGTTT
TGTGGCATGTGAGGTACTGGGTGAAAGACAGAAGCCTTCGTCTCCATAGATTCACAAGAGCAGCATTGTGTAAAGGG
>AY677147.1bonoboT2R43
ATGATAACTTTTCTACCCATCATTTTTTCCAGTCTGGTAGTGGTTACATTTGTTATTGGAAATTTTGCTAATGGCTTCAT
AGCATTGGTAAATTCCATTGAGTGGTTCAAGAGACAAAAGATCTCCTTTGCTGACCAAATTCTCACTGCTCTGGCGGTCT
CCAGAGTTGGTTTGCTCTGGGTATTATTATTAAACTGGTATTTAACTGTGTTGAATCCAGCTTTTAATAGTGTAGAAGTA
AGAACTACTGCTTATAATATCTGGGCAGTAATCAACCATTTCAGCAACTGGCTTGCTACTAGCCTCAGCATATTTTATTT
GCTCAAGATTGCCAATTTCTCCAACTTTATTTTTCTTCACTTAAAGAGGAGAGTTAAGAGTGTCATTCTGGTGATGTTGT
TGGGGCCTTTGCTATTTTTGGCTTGTCATCTTTTTATGATAAACATGAATGAGATTGTGCGGACAAAAGAATTTGATGGA
AACATGACTTGGAAGATCAAATTGAAGAGTGCAATGTACTTTTCAAATATGACTGTAACCATGGTAGCAAACTTAGTACC
CTTCACTCTGACCCTACTATCTTTTTTGCTGTTAATCTGTTCTTTGTGTAAACATCTCAAGAAGATGCAGCTCCATGGTA
AAGGATCTCAAGATCCCAGCACGAAGGTCCACATAAAAGCTTTGCAAACTGTGATCTCCTTCCTCTTGTTATGTGCCATT
TACTTTCTGTCCATAATGATATCAGTTTGGAGTTTTGGAAGTCTGGAAAACAAACCTGTCTTCATGTTCTGCAAAGCTAT
TAGATTCAGCTATCCTTCAATCCACCCATTCATCCTGATTTGGGGAAACAAGAAGCTAAAGCAGACTTTTCTTTCAGTTT
TTTGGCAAATGAGGTACTGGGTGAAAGGAGAGAAGACTTCATCTCCATGA
>AY724889.1chimpT2R43
ATGATAACTTTTCTACCCATCATTTTTTCCAGTCTGGTAGTGGTTACATTTGTTATTGGAAATTTTGCTAATGGCTTCAT
AGCATTGGTAAATTCCATTGAGTGGTTCAAGAGACAAAAGATCTCCTTTGCTGACCAAATTCTCACTGCTCTGGCGGTCT
CCAGAGTTGGTTTGCTCTGGGTATTATTATTAAACTGGTATTCAACTGTGTTGAATCCAGCTTTTAATAGTGTAGAAGTA
AGAACTACTGCTTATAATATCTGGGCAGTAATCAACCATTTCAGCAACTGGCTTGCTACTAGCCTCAGCATATTTTATTT
GCTCAAGATTGCCAATTTCTCCAACTTTATTTTTCTTCACTTAAAGAGGAGAGTTAAGAGTGTCATTCTGGTGATGTTGT
TGGGGCCTTTGCTATTTTTGGCTTGTCATCTTTTTGTGATAAACATGAATGAGATTGTGCGGACAAAAGAATTTGAAGGA
AACATGACTTGGAAGATCAAATTGAAGAGTGCAATGTACTTTTCAAATATGACTGTAACCATGGTAGCAAACTTAGTACC
CTTCACTCTGACCCTACTATCTTTTTTGCTGTTAATCTGTTCTTTGTGTAAACATCTCAAGAAGATGCAGCTCCATGGTA
AAGGATCTCAAGATCCCAGCACCAAGGTCCACATAAAAGTTTTGCAAACTGTGATCTCCTTCCTCTTGTTATGTGCCATT
TACTTTCTGTCCATAATGATATCAGTTTGGAGTTTTGGGAGTCTGAAAAACAAACCTGTCTTCATGTTCTGCAAAGCTAT
GAGATTCAGCTATCCTTCAATCCACCCATTCATCCTGATTTGGGGAAACAAGAAGCTAAAGCAGACTTTTCTTTCAGTTT
TTTGGCAAATGAGGTACTGGGTGAAAGGAGAGAAGACTTCATCTCCATAG
>AB713347.1chimpT2R45haplotype1
ATGATAACTTTTCTGCCCATCATATTTTCCATTCTAGTAGTGGTTACATTTGTTATTGGAAATTTTGCTAATGGCTTCAT
AGCGTTGGTAAATTCCACCGAGTGGGTGAAGAGACAAAAGATCTCCTTTGCTGACCAAATTGTCACTGCTCTGGCGGTCT
CCAGAGTTGGTTTGCTCTGGGTGTTATTATTAAATTGGTATTCAACTGTGTTGAATCCAGCTTTTTATAGTGTAGAATTA
AGAACTACTGCTTATAATATCTGGGCAGTAACCGGCCATTTCAGCAACTGGCTTGCTACTAGCCTCAGCATATTTTATTT
GCTCAAGATTGCCAATTTCTCCAACCTTATTTTTCTTCGCTTAAAGAGGAGAGTTAAGAGTGTCATTCTGGTGATGCTGT
TGGGGCCTTTGCTATTTTTGGCTTGTCATCTTTTTGTGGTAAACATGAATCAGATTGTGTGGACAAAAGAATATGAAGGA
AACATGACTTGGAAGATCAAATTGAGGCGTGCAATGTACCTTTCAGATACGACTGTAACCATGCTAGCAAACTTAGTACC
CTTTGCTCTAACCCTGATATCTTTTCTGCTGTTAGTCTGTTCTCTGTGTAAACATCTCAAGAAGATGCAACTCCATGGCA
AAGGATCTCAAGATCCCAGTACCAAGGTCCACATAAAAGCTTTGCAAACTGTGATCTCCTTCCTCTTGTTATGTGCCATT
TACTTTGTGTCTGTAATAATATCAGTTTGGAGTTTTAAGAATCTGGAAAACAAACCTGTCTTCATGTTCTGCCAAGCTAT
TGGATTCAGCTGTTCTTCAGCCCACCCGTTCATCCTGATTTGGGGAAACAAGAAGCTAAAGCAGACTTTTCTTTCAGTTT
TGTGGCAAATGAGGTACTGGGTGAAAGGAGAGAAGCCTTCATCTCCATAG
>AY724973.1BOrangT2R47partial
ATGATAACTTTTCTGCCCATCATTTTTTCCATTCTAATAGTGGTTATATTTGTTATTGGAAATTTTGCTAATGGCTTCAT
AGCATTGGTAAATTCCATTGAGTGGGTCAAGAGACAAAAGATCTCCTTTGCTGACCAAATTCTCATTGCTCTGGCGGTCT
CCAGAGTTGGTTTGCTCTGGGCATTATTACTACATTGGTATGCAACTGAGTTGAATCTAGCTTTTTATAGTGTAGAAGTA
AGAATTACTGCTTATAATGTCTGGGCAGTAACCAACCATTTCAGCAACTGGCTTGCTACTAGTCTCAGCATGTTTTATTT
GCTCAAGATTGCCAATTTCTCCAACCTTATTTTTCTTCGCATAAAGAGGAGAGTTAAAAGTGTCATTCTGGTGATACTGT
TGGGGCCTTTACTGTTTTTGGTTTGTCATCTTTTTGTGATAAACATGAATGAGATTGTATGGACAAAAGAATATGAAGGA
AATTTGACTTGGAAGATCAAATTGAGGAATGCAGTGTTCCTTTCAAATATGACTCTAACCATGCTAGCAAACTTTGTACC
CCTCACTCTGACCCTGATATCTTTTCTGCTGTTAATCTGTTCTCTGTGTAAACATCTCAAGAAGATGCAGCTCCATGGCA
AAGGATCTCAAGACCCCAGCACCAAGGTCCACATAAAAGCTTTGCAAACTGTGACCTGCTTTCTCCTGTTATGTGCCATT
TACTTTCTGTCCATGATCATATCAGTTTATAATTTTGGGAGGCTGGAAAAGAAACCTGTCTTCATGTTCTGCCAAGCTAT
TACATTCAGCTATCCTTCAACCCATGCATTCATCCTGATTTGGGGAAACAAGAAGCTAAAGCAGATTTTTCTTTCAGTTT
TGTGGCATGTGAGGTACTGGGTGAAAGACAGAAGCCTTCGTCTCCATAGATTCACAAGAGCAGCATTGTGTAAAGGG
>AY724878.1chimpT2R44haplotype1
ATGACAACTTTTATACCCATAATTTTTTCCAGTCTGGTAGTGGTTATATTTGTTATTGGAAATTTTGCTAATGGCTTCAT
AGCATTGGTAAATTCCATTGAGTGGTTCAAGAGACAAAAGATCTCTTTTGCTGACCAAATTCTCACTGCTCTGGCGGTCT
CCAGAGTTGGTTTGCTCTGGGTATTATTATTAAATTGGTATTCAACTGTGTTGAATCCAGCTTTTTATAGTGTAGAAGTA
AGAACTACTGCTTATAATGTCTGGGCAGTAACCGGCCATTTCAGCAACTGGCTTGCTACTAGCCTCAGCATATTTTATTT
GCTCAAGATTGCCAATTTCTCCAACCTTATTTTTCTTCACTTAAAGAGGAGAGTTAAGAGTGTCATTCTGGTGATGCTGT
TGGGGCCTTTACTATTTTTGGCTTGTCAACTTTTTATGATAAACATGAAAGAGATTGTACGGACAAAAGAATATGAAGGA
AACATGACTTGGAAGATCAAATTGAGGAGTGCAGTGTACCTTTCAGATGCGACTGTAACCACGCTAGGAAACTTAGTGCC
CTTCACTCTGACCCTACTATGTTTTTTGCTGTTAATCTGTTCTCTGTGTAAACATCTCAAGAAGATGCAGCTCCATGGTA
AAGGATCTCAAGATCCCAGCACCAAGGTCCACATAAAAGTTTTGCAAACTGTGATCTCCTTCCTCTTGTTATGTGCCATT
TACTTTCTGTCCATAATGATATCAGTTTGGAGTTTTGGGAGTCTGAAAAACAAACCTGTCTTCATGTTCTGCAAAGCTAT
GAGATTCAGCTATCCTTCAATCCACCCATTCATCCTGATTTGGGGAAACAAGAAGCTAAAGCAGACTTTTCTTTCAGTTT

TGCGGCAAGTGAGGTACTGGGTGAAAGGAGAGAAGCCTTCATCTCCATAG
>AY724849.1bonoboT2R44
ATGACAACTTTTCTACCCATCATTTTTTCCAGTCTGGTAGTGGTTATATTTGTTATTGGAAATTTTGCTA
ATGGCTTCATAGCATTGGTAAATTCCATTGAGTGGTTCAAGAAACAAAAGATCTCTTTTGCTGACCAAAT
TCTCACTGCTCTGGCGGTCTCCAGAGTTGGTTTGCTCTGGGTATTATTATTAAATTGGTATTCAACTGTG
TTGAATCCAGCTTTTTATAGTGTAGAAGTAAGAACTACTGCTTATAATGTCTGGGCAGTAACCGGCCATT
TCAGCAACTGGCTTGCTACTAGCCTCAGCATATTTTATTTGCTCAAGATTGCCAATTTCTCCAACTTTAT
TTTTCTTCACTTAAAGAGGAGAGTTAAGAGTGTCATTCTGGTGATGCTGTTGGGGCCTTTACTATTTTTG
GCTTGTCAACTTTTTATGATAAACATGAAAGAGATTGTACGGACAAAAGAATATGAAGGAAACATGACTT
GGAAGATCAAATTGAGGAGTGCAGTGTACCTTTCAGATGCGACTGTAACCACGCTAGGAAACTTAGTGCC
CTTCACTCTGACCCTACTATGTTTTTTGCTGTTAATCTGTTCTCTGTGTAAACATCTCAAGAAGATGCAG
CTCCATGGTAAAGGATCTCAAGATCCCAGCACCAAGGTCCACATAAAAGTTTTGCAAACTGTGATCTCCT
TCCTCTTGTTATGTGCCATTTACTTTCTGTCCATAATGATATCAGTTTGGAGTTTTGGGAGTCTGAAAAA
CAAACCTGTCTTCATGTTCTGCAAAGCTATTAGATTCAGCTATCCTTCAATCCACCCATTCATCCTGATT
TGGGGAAACAAGAAGCTAAAGCAGACTTTTCTTTCAGTTTTGCGGCAAGTGAGGTACTGGGTGAAAGGGG
AGAAGCCTTCATCTCCATAG
>AY677149.1bonoboT2R45
ATGATAACTTTTCTGCCCATCATATTTTCCATTCTAGTAGTGGTTACATTTGTTATTGGAAATTTTGCTAATGGCTTCAT
AGCGTTGGTAAATTCCACCGAGTGGGTGAAGAGACAAAAGATCTCCTTTGCTGACCAAATTGTCACTGCTCTGGCGGTCT
CCAGAGTTGGTTTGCTCTGGGTGTTATTACTAAATTGGTATTCAACTGTGTTGAATCCAGCTTTTTATAGTGTAGAATTA
AGAACTACTGCTTATAATATCTGGGCAGTAACCGGCCATTTCAGCAACTGGCTTGCTACTAGCCTCAGCATATTTTATTT
GCTCAAGATTGCCAATTTCTCCAACCTTATTTTTCTTCACTTAAAGAGGAGAGTTAAGAGTGTCATTCTGGTGATGCTGT
TGGGGCCTTTGCTATTTTTGGCTTGTCATCTTTTTGTGGTAAACATGAATCAGATTGTGTGGACAAAAGAATATGAAGGA
AACATGACTTGGAAGATCAAATTGAGGCGTGCAATGTACCTTTCAGATACGACTGTAACCATGCTAGCAAACTTAGTACC
CTTTACTGTAACCCTGATATCTTTTCTGCTGTTAGTCTGTTCTCTGTGTGAACATCTCAAGAAGATGCAACTCCATGGCA
AAGGATCTCAAGATCCCAGTACCAAGGTCCACATAAAAGCTTTGCAAACTGTGATCTCCTTCCTCTTGTTATGTGCCATT
TACTTTGTGTCTGTAATAATATCAGTTTGGAGTTTTAAGAATCTGGAAAACAAACCTGTCTTCATGTTCTGCCAAGCTAT
TGGATTCAGCTGTTCTTCAGCCCACCCGTTCATCCTGATTTGGGGAAACAAGAAGCTAAAGCAGCCTTTTCTTTCAGTTT
TGTGGCAAATGAGGTACTGGGTGAAAGGAGAGAAGCCTTCATCTTCATAG
>AY724974.1BOrangT2R44
ATGATAACTTTTCTACCCACCATTTTTTCCATTCTGGTGGTGGTTATATTTGTTATTGGAAATTTTGGTAATGGCTTCAT
AGCACTGGTAAATTCCATTGAGTGGGTCAAGAGACAAAAGATCTCCTTTGCTGACCAAATTCTCACTGCTCTGGCAGTCT
CCAGAGTTGGTTTGCTCTGGGCATTATTATTAAATTGGTATTCAACTGTGTTTAATCCAGCTTTTTATAGTGTAGGAGTA
AGAACTACTGTTTATGATGTCTGGACAGTAACCGGCCATTTCAGCAACTGGCTTGCTACTAGCCTCAGCATATTTTATTT
GCTCAAGATTGCCAATTTCTCCAACCTTATTTTTCTTCACTTAAAGAGGAGAGTTAAGAGTGTCATTCTGGTGATGCTGT
TGGGGCCTTTGCTATTTTTGGCTTGTCAACTTTTTGTGATAAACATGAAAGAGATTCTACGGACAAAAGAATATGAAGGA
AACATGACTTGGAAGATCAAATTGAGGAGTGCAATGTACCTTTCAGATGCGACTATAACCACGCTAGCAAACTTAGTACC
CTTCACTCTGACCCTACTGTCTTTTTTGCTGTTAATCTGTTCTTTGTGTAAACATCTCAACAAGATGCAGCTCCATGGTA
AAGGATCTCAAGATCCCAGCACCAAGGTCCACATAAAAGTTTTGCAAACTGTGATCTCCTTCCTCTTGTTATGTGCCATT
TACTTTCTGTCCATAATGATATCAGTTTGGAGTTTTGGGAGTCTGGAAAACAAACCTGTCTTCATGTTCTGCAAAGCTAT
TAGATTCAGCTATCCTTCAATCCACCCATTCATCCTGATTTGGGGAAACAAGAAGCTAAAGCAGACTTTTCTTTCAGTTT
TGCGGCAAGTGAGGTACTGGGTGAAAGGAGAGAAGCCTTCATCTCCATAG
>AY725007.1RmacaqT2R43
ATGATAACTTTTTTACCCATCATTTTTTCCATTCTAGTAGTGTTTACATTTGTTATTGGAAATTTTGCTAATGGTTTCAT
AGCATTGGTAAATTCCATTGAGTGGGTCAAGAGACAAAAGATCTCCTTTGCTGACCAAATTCTCACTGCTCTGGCAGTCT
CCAGAGTTGGTTTGCTCTGGATATTATTATTAAATTGGTATTCAACTGTTTTGAATCCAGCTTTTTATAGTGTAGAAGTA
AGAACTATTGCTTATAATCTCTGGGCAGTAATCAACCATTTCAGCAACTGGCTTGCTACTAGCCTAAGCATATTTTACTT
ACTCAAGATTGCCAATTTCTCCAACCTTATATTTCTTCACTTAAGGAGGAGAGTTAAGAGTGTCGTTCTGGTGATACTGT
GGGGGCCTTTGCTATTTTTGGTTTGTCATCTTTTTGTGGTAAACATGAATGAGATTATACAGACAAAAGAATATGAAGGA
AACATGACTTGGAAGAGCAAATTGAGGAGTGCAATGTACCTTTCAAATACGACTGTAACCATACTAGCAAACTTAGTACC
CTTCATTCTGACCCTAATATCTTTTCTGCTGTTAATCTGTTCTCTGTGTAAACATCTCAAGAAGATGCAGCTCCGTGACA
AAGGCTCTCAAGATCCCAGCACCAAGGTCCACATAAAAGCTTTGCAAACTGTCATCTCCTTGTCGTTATGTGCCATTTAC
TTTCTGTCCATAATGATATCAAGTTGGAGTTTGGGAAGGGTGGAAAACAAAGCTATCTTCATGTTCTGCAAAGCTATTAG
ATTCAGCTATCCTTCAGCCCACGCATTCATACTGATTTGGGGAAACAAGAAGCTAAAGCAGACTCTTCTTTCAGTTTTGT
GGAACGTGAGGTACTGCGTGAAAGGACAGAAGCTTCAATCTCCATAG
>AY724912.1gorillaT2R44
ATGACAACTTTTATACCCATCATTTTTTCCAGTCTGGTAATGGTTATGTTTGTTACTGGAAATTTTGCTA
ATGGCTTCATAGCATTGGTAAATTCCATTGAGTCGGTCAAGAGACAAAAGATCTCTTATGCTGACCAAAT
TCTCACTGCTCTGGCAGTCTCCAGAATTGGTTTGCTCTGGGTATTATTATTAAATTGGTATTCAACTGTG
TTGAATCCAGCTTTTTATAGTGTAGAAGTAAGAACTACTGCTTATAATGTCTGGGCAGTAACCGGCCATT
TCAGCAACTGGCTTGCTACTAGCCTCAGCATATTTTATTTGCTCAAGATTGCCAATTTCTCCAACCTTAT
TTTTCTTCACTTAAAGAGGAGAGTTAAGAGTGTCATCCTGGTGATGCTGTTGGGGCCTTTACTATTTTTG
GCTTGTCAACTTTTTGTGATAAACATGAAAGAGATTGTACAGACAAAAGAATATGAAGGAAACATGACTT
GGAAGATCAAATTGAGGAGTGCAGTGTACCTTTCAGATGCGACTGTAACCACGCTAGGAAACTTAGTGCC
CTTCACTCTGACCCTACTATGTTTTTTGCTGTTAATCTGTTCTCTGTGTAAACATCTCAAGAAGATGCAG
CTCCATGGCAAAGGATCTCAAGATCCCAGCATGAAGGTCCACATAAAAGCTTTGCAAACTGTGACCTCCT

TCCTCTTGTTATGTGCCATTTACTTTCTGTCCATAATGATATCAGTTTGGAGTTTGGGGAGTCTGAAAAA
CAAACCTGTCTTCATGTTCTGCAAAGCTATGAGATTCAGCTATCCTTCAATCCACCCATTCATCCTGATT
TGGGGAAACAAGAAGCTAAAGCAGACTTTTCTTTCAGTTTTGCAGCAAGTGAGGTACTGGGTGAAAGGAG
AGAAGCCTTCATCTCCATAG
>AY724823.1HbaboonT2R44
ATGATAACTTTTCTACCCATCATTTTTTCCATTCTAGTAGTGGTTACATTTGTTATTGGAAATTTTGCTAATGGCTTCAT
AGCATTGGTAAATTCCACTGAGTGGGTCAAGAGACAAAAGATCTCCTTTGCTGACCAAATTCTCACTGCTCTGGCGGTTT
CCAGAGTTGGTTTGCTCTGGGTATTATTATTAAATTGGTATGCAACTGTGTTGAATCCAGCGTTTTATAGTGTAGAAGTA
AGAACTACCACTTATAATGTCTGGGCAGTAACCAACCATTTCAGCAACTGGCTTGCTACTAGTCTCAGCATATTTTATTT
GCTCAAGATTGCCAATTTCTCCAACCTTATTTTTCTTCACTTAAAGAGGAGAGTTAAGAATGTCATTCTGGTGATGCTGT
TGGGGCCTTTGCTCATTTTGGCTTGTCATCTTTTTATGGTAAACATGAATGAGATTGTACGGACAAAAGAGTATGAAGAA
AACATGACTTGGAAGTACATATTGAGGAATGCGATTTACCATCCAGGTATGACTGTAACCACGCTACAGAACTTAGTACC
TTTCACTCTGACCCTGATATCCTTTCTGCTGTTAATCTGTTCTCTGTGTAAACATCTCAAGAAGATGCAGCTCCATGGCA
AAGGACCTCAAGATCCCAGCACCAAGGTCCACATAAAAGCTTTGCAAATTGTGATCTCCTTCCTCTTGTTATGTGTCATT
TACTTTGTGTCTGTAATTATATCAATTTGGAGTTTTGAGAGTCTGGGAAACAAACCTGTCTTCATGTTCTGCCAAGCTAT
TAGATTCAGCTATCCTTCAGCCCACCCATTCATCGTGATTTGGGGAAACAAGAAGCTAAAGCAGACTTTTCTTTCAGTTT
TGTGGAACGTGAGGTACTGGGTGAAAGGACAGAAGCCTTCATCTCTGTAG
>AY724822.1HbaboonT2R46
ATGATAACTTTTCTGCCCATCACTTTTTCCATTCTAATAGTGGTTATATTTTTTATTGGAAATTTTGCTAATGGCTTCAT
AGCATTGATAAATTCTATTGAGTGGGTCAAGAGACAAAAGATCTCCTTTGCTGGCCAAATTCTCACTGCTCTGGCGGTCT
CCAGAGTTGGTTTGCTCTGGGTATTATCACTACATTGGTATGCAACTGAGTTTAATCTAGCTTTTCATAGTGTAGAAGTA
AGAAGTACTGCTTATAATGTCTGGGTAGTGACCAACCATTTCAGCAACTGGCTTTCTACTAGCCTCAGCATGTTTTATTT
GCTCAGAATTGCCACTTTCTCCAACCTGATTTTTCTTCACTTAAATAGGAGAGTAAAGAGTGTCATTCTGGTGACACTGT
TAGGGCCTTTGCTGTTTTTGGTTTGTCAACTTTTTGTGATGAACATGAATCAGATTGTACGGACAAAAGAATATGAAGGA
AACATGACTTGGAAGATCAAATTGAAGAGTGCAATGTACCTTTCAAATACAACTGTAGCCATGCTAGCAAACTTTGTACC
CCTCACTCTGACCCTGATATCTTTTCTGCTGTTAATCTGTTCTCTGTGTAAACATCTCAAGAAGATGCGGGTCCATGGCA
AAGGATCTCAAGATCCCAGCACCAAGGTCCACACAAAAGCTTTGCAAATTGTGACCTCCTTTCTCCTGGTATGTGCCATT
TACTTTCTGTCCATAATCCTATCAGTTTGGAATTCTGGGGGGCTGGAAAACAAACCTTTCTTCATGTTCTGCCAAGCTAT
TAAATTCAGCTATCCTTCCACCCACCCATTCATCCTGATTTGGGGAAACAAGACACTAAAGCAGACTTTTCTTTCAGTTT
TAAGGAATGTGAGGTACTGGGTGAAAGGACAGAAGCCTTCATCTCCATAG
>NM_001080757.2RmacaqT2R46
ATGATAACTTTTCTGTCCATCACTTTTTCCATTCTAGTAGGGGTTATATTTGTTATTGGAAATTTTGCTAATGGCTTCAT
AGCATTGGTAAATTCCATTGAGTGGGTGAAAAGACAAAAAATCTCCTTTGCTGACCAAATTCTCACTGGTCTGGCTGTCT
CCAGAGTTGGTTTGCTCTGGGTATTATTACTACATTTGTATGCAACTGAGTTTAATCTAGCTTTTTATAGTGTAGAAGTA
AGAATCACTGCTTATAATGTCTGGATAGTGACCAACCATTTCAGCAACTGGCTTTCTACTAGCCTCAGCATGTTTTATTT
GCTCAAGATTGCCACTTTCTCCAACCTGATTTTTCTTCACTTAAAGAGGAAAGTTAAGAGTGTCATTCTGGTGACACTGT
TGGGGCCTTTGCTATTTTTGGTTTGTCATCTTTTTGTGATGAACATGAATCATATTGTGTGGAGAAAAGAATATGAAGGA
AACATTACTTGGAGGATCAAATTGAGGAGTGCAATGTACCTTTCAAATGTGACTGTAACCATGCTAGCAAACCTTATACC
CCTCACTCTGACCCTGATGTCTTTTCTGCTGTTAATCTGTTCTCTGTGTAAACATCTCAAGAAGATGCAGGTCCACGGCA
AAGGATCTCAAGATCCCAGCACCAAGGTCCACATAAAAGCTTTGCAAACTGTGACCTCCTTTCTCCTGTTATGTGCCATT
TACTTTCTGTCCATGATCCTATCAGTTTGGAATTTTGAGCTGGAAAAGAAACCTGTCTTCATGTTCTGCCAAGCTGTCAT
ATTCAGCTATCCTTCAACCCACCCACTCATCCTGATTTGGGGAAACAAGAAGCTAAAGCAGATTTTTCTTTCAGTTTTGT
GGAACGTGAGATACTGGGTGAAAGGACAGAAGCCTTCATCTCCATAG
>NM_001009143.1chimpT2R50
ATGATAACTTTTCTGTACATTTTTTTTTCAATTCTAATAATGGTTTTATTTGTTCTCGGAAACTTTGCCA
ATGGCTTCATAGCACTGGTAAATTTCATTGACTGGGTGAAGAGAAAAAAGATCTCCTCAGCTGACCAAAT
TCTCACTGCTCTGGCGGTCTCCAGAATTGGTTTGCTCTGGACATTATTATTAAATTGGTATTTAACTGTG
TTGAATCCAGCTTTTTATAGTGTAGAATTAAGAATTACTTCTTATAATGCCTGGGTTGTAACCAACCATT
TCAGCATGTGGCTTGCTGCTAGCCTCAGCATATTTTATTTGCTCAAGATTGCCAATTTCTCCAACCTTAT
TTTTCTTCATTTAAAGAGGAGAGTTAGGAGTGTCATTCTGGTGATACTGTTGGGGACTTTGATATTTTTG
GTTTGTCATCTTCTTGTGGCAAACATGGATGAGAGTATGTGGGCAGAAGAATATGAAGGAAACATAACTG
GGAAGATGAAATTGAGGAATACAGTACATCTTTCRTATTTGACTGTAACTACCCTATGGAGCTTCATACC
CTTTACTCTGTCCCTGATATCTTTTCTGATGCTAATCTGTTCTCTGTGTAAACATCTCAAGAAGATGCAG
CTCCATGGAGAAGGATCTCAAGATCTCAGCACCAAGGTCCACATAAAAGCTTTGCAAACTCTGATCTCCT
TCCTCTTGTTATGTGCCATTTTCTTTCTATTCCTAATCATTTCGGTTTGGAGTCCTAGGAGGCTGCGGAA
TGACCCAGTTGTCATGGTTAGCAAGGCTGTTGGAAACATATATCTTGCATTCGACTCATTCATCCTAATT
TGGAGAACCAAGAAGCTAAAACACACCTTTCTTTTGATTTTGTGTCAGATTAGGTGCTGA

Phylogenetic Tree constructed using RaxML-HPC using 105IG (w/o Pseudogenes)
((((((R45Bbat:0.16785508619059169577,(r66Dbat:0.06476287012952890165,r46Bbat:0.04257770784011617043):0.10949670668196873069):0.14412686880673800105,((PANDA_014286:0.09097775310745906396,r31panda:0.09594761764246795277):0.08744809125793295834,R136Canis:0.16249011442857910770):0.09328353150456987486):0.02751754081864728890,(((r31cattle:0.00000100000050002909,R46Dyak:0.00693149434296178469):0.15045612736790164865,r31Dyak:0.15938839958728079349):0.08684603368601805096,(r46cattle:0.014

01480729405889941,r45Dyak:0.00312181697565917446):0.25903158910957430061):0.08646524778647958487)
:0.04525779780570217403,(((((r50CEmacaq:0.00348038390573997660,R50Rmacaq:0.00000100000050002909):
0.05644030515998799125,(r50Ngibbon:0.01199582290184376607,((R50gorilla:0.00000100000050002909,(hT
2R50:0.01386868982935177130,r50chimp:0.01039039965234317582):0.00693021356891516515):0.0030210461
0680323585,R50BOrang:0.04281951018870267661):0.00681635693680104123):0.03910769940121210098):0.18
443351496884594076,(((((R47gorilla:0.00342601753837432554,(hT2R47:0.01235778549665817773,(R47chim
p:0.00000100000050002909,R47bonobo:0.00325773062264037927):0.01648389514535455319):0.003112979537
72996005):0.03206800657912484143,(R47BOrang:0.02456340792996643610,(R47Agibbon:0.0168265145333047
7956,R47Ngibbon:0.00969635554000993112):0.02951230052649510618):0.01082202442487545727):0.0603962
3558825493732,(R46CEmacaq:0.06204079045917219870,(R47Rmacaq:0.09694512608998660796,R46Hbaboon:0.1
0497586331770292212):0.01602231379615330284):0.02001657670659557145):0.0239903645849294984,(((R4
6chimp:0.02659868583933879330,(R66bonobo:0.06136582692747305501,R46bonobo:0.00000100000050002909)
:0.00000100000050002909):0.00326355460265260631,R46gorilla:0.00978041685421726127):0.007440485265
25010936,hT2R46:0.00893635866728705909):0.04904799238888805230):0.01851309596711641151,(((((hT2R4
3:0.01298560771213007857,(R43chimp:0.00323638495704920695,R43bonobo:0.00978971548860215444):0.000
00100000050002909):0.04261946397904926159,((hT2R44:0.01975311769303322862,(R44gorilla:0.039775322
09917884060,(44bonobo:0.00380101357154824212,R44chimp:0.00602120711856811864):0.00989951770865945
060):0.00360104506152724704):0.01890684952506720140,(R44BOrang:0.02628218520390722054,R44Agibbon:
0.01167232475677889778):0.01470460498891772855):0.02718529283889163997):0.01299894786619518183,((
R43Hbaboon:0.01338440404993520259,(R43Rmacaq:0.00973023388299498748,R46Rmacaq:0.00000100000050002
909):0.02289238091378816703):0.07542946586825770294,((R45Rmacaq:0.03351222268265523219,r43Ngibbon
:0.03145598574500645117):0.03893679655386318023,(R43Slutung:0.02749674885438497043,R45Slutung:0.0
7646464488196827447):0.01178157157618900559):0.03800845963670343441):0.01559713252996247981):0.01
174028303184796417,(((R45bonobo:0.00872616765153705863,((R45BOrang:0.03086946580623557701,R45gori
lla:0.00000100000050002909):0.01090840413521744316,hT2R45:0.02415792662706062188):0.0033541534148
7437868):0.00440343837462973172,R45chimp:0.00323790394320530714):0.05827296443043478852,(R44Hbabo
on:0.00574755039620019822,R8Rmacaq:0.02341199595992762839):0.07646227510282604944):0.010674499566
98822595):0.01632180911198055639,r2Rhowler:0.13556624208180853564):0.00772742569828340116):0.0987
3694017064019168):0.03201368210175500190,(R15BOrang:0.11263167448838637408,r3Rhowler:0.0915874856
0789126136):0.08435481038651350405):0.01750904577969297077,(((r49CEmacaq:0.00000100000050002909,r
49Rmacaq:0.00325187043473366185):0.08084075644090416013,(R49BOrang:0.03264718134464093813,((r49Ng
ibbon:0.00000100000050002909,R49Agibbon:0.00475378662941416171):0.03809311636101225113,((hT2R49:0
.00966691264791887626,r49chimp:0.00000100000050002909):0.00641986373629082827,(R49gorilla:0.00640
166299437595378,R49Hbaboon:0.00000100000050002909):0.00319335387979864270):0.01664174826013561231
):0.01894922786721564903):0.04628993758459305774):0.13346702152109379824,((r48chimp:0.00221583978
900921847,(R48bonobo:0.00675311284964974327,((hT2R48:0.01363775607287482511,R48BOrang:0.067838963
34235400838):0.00664261151676752860,R48gorilla:0.00340225165739667986):0.01373827827156333045):0.
00804607661341410867):0.04445141436782883843,((r64chimp:0.01347797422335973543,R64bonobo:0.037578
87816893402261):0.03337388615503653760,(R64Rmacaq:0.00775018544526102221,(r48Rmacaq:0.00000100000
050002909,r48CEmacaq:0.00345063004733767642):0.00000100000050002909):0.13829267624983632157):0.01
852147002659353808):0.15350346008495432959):0.03325441227615251460):0.15866789675451375974):0.390
84835652529620331,(pigT2R10:0.11802640102119846266,((((AcinonyxJubatusR10:0.00897175188736000322,
PantheraPardusR10:0.02279229279692889312):0.10090728875795866581,(R10panda:0.07714312556707239588
,((TibetanFoxR10:0.00000100000050002909,((RedFoxR10:0.00921529772248975802,CorsacFoxR10:0.0030702
5504757614618):0.00000100000050002909,FennecFoxR10:0.01544674233933003918):0.00000100000050002909
):0.00000100000050002909,((ManedWolfR10:0.00000100000050002909,(HuntingDogR10:0.00000100000050002
909,R10Canis:0.00000100000050002909):0.00000100000050002909):0.00000100000050002909,WolfR10:0.003
07699327065548986):0.00306591242716984860):0.03698326577255928865):0.03272019741161429074):0.0302
3062072191510033,((R10CMarmos:0.17802244026560018475,((R10HBaboon:0.03589182936059823886,R10Rmaca
q:0.03133137229643926519):0.01697901239198852663,((((hT2R10:0.01630498653662823885,R10gorilla:0.0
0000100000050002909):0.00650231460432945402,R10chimp:0.00975168558463896003):0.014199929320126937
14,R10NGibbon:0.02859704653729683299):0.01197713697902935692,R10BOrang:0.03155626183128412005):0.
01701513276050084164):0.07262412758409245161):0.05511136638330126764,((R107Rat:0.0950236237521145
5270,R107Mus:0.07655971983606278675):0.30604723707914832653,(T7Gmolerat:0.36304224398333151402,(R
106Mus:0.06175205110843865614,R106Rat:0.11163871231443148191):0.23151373832902352734):0.037549439
30375453931):0.16384472180919587569):0.06865592076078305683):0.06867710106316346486,(R10sheep:0.1
5596508049546503605,R10cattle:0.10223518806747364240):0.15566788615305804377):0.03658290176263843
257):0.56260564644257815559):0.33444998687689120320,(T2R20_frog:1.38169342296592456698,(T2R_allig
ator:1.41637326001478558446,((((V1r69_mus:0.64089091363909833632,v1r3_chimp:0.4099574260367919720
2):0.30139798748998114508,V1r62_mus:1.21377588811888648124):0.22722228179968001482,(V1r96_rat:1.0
3169333555393683000,v1r_RMacaq:1.10403442205472623883):0.11217201312090271936):0.4201977404830389
8395,(V1r_Zebrafish:0.52508611701573437713,(V1R_alligator:1.10424726801482431249,v1r_frog:0.55569
103288371557436):0.19773023603799655401):0.17331377793303751056):0.49069189796693885031):1.144699
41532197516665):0.33444998687689120320);

Phylogenetic Tree with Reconstructed Ancestral Sequences using FastML - 105IG, w/o Pseudogenes

```
((((((r45Mbr:0.170044,(r66Mda:0.065432,r46Mbr:0.043134)N7:0.111186)N6:0.146409,((t2rAme:0.092284,
r31Ame:0.097170)N9:0.088813,r136Clu_fa:0.164252)N8:0.093948)N5:0.027835,(((r44Bta:0.000000,r46Bmu
:0.007027)N12:0.152271,r44Bmu:0.161728)N11:0.088115,(r46Bta:0.014294,r45Bmu:0.003075)N13:0.262550
)N10:0.087151)N4:0.046134,(((((r50Mfa:0.003529,R50Mmu:0.000000)N18:0.057230,(r50Nle:0.012144,((r5
0Ggo:0.000000,(hT2R50Hsa:0.014065,r50Ptr:0.010537)N22:0.007027)N21:0.003084,r50Ppy:0.043444)N20:0
.006896)N19:0.039676)N17:0.187167,(((((r47Ggo:0.003473,(hT2R47Hsa:0.012521,(r47Ptr:0.000000,r47Pp
a:0.003301)N29:0.016706)N28:0.003139)N27:0.032478,(r47Ppy:0.024881,(r47Hag:0.017049,r47Nle:0.0098
36)N31:0.029948)N30:0.010965)N26:0.061248,(r46Mfa:0.062950,(r47Mmu:0.098245,r46Pha:0.106449)N33:0
.016207)N32:0.020343)N25:0.024263,(((r46Ptr:0.026961,(r66Ppa:0.062237,r46Ppa:0.000000)N37:0.00000
0)N36:0.003306,r46Ggo:0.009906)N35:0.007532,hT2R46Hsa:0.009058)N34:0.049752)N24:0.018863,(((((hT2
R43Hsa:0.013146,(r43Ptr:0.003277,r43Ppa:0.009907)N43:0.000000)N42:0.043212,((hT2R44Hsa:0.019998,(
r44Ggo:0.040286,(r44Ppa:0.003848,r44Ptr:0.006098)N47:0.010029)N46:0.003646)N45:0.019143,(r44Ppy:0
.026590,r44Hag:0.011803)N48:0.014899)N44:0.027555)N41:0.013158,((r43Pha:0.013568,(r43Mmu:0.009859
,r46Mmu:0.000000)N51:0.023193)N50:0.076497,((r45Mmu:0.033957,r43Nle:0.031848)N53:0.039468,(r43Tcr
:0.027872,r45Tcr:0.077483)N54:0.011876)N52:0.038628)N49:0.015694)N40:0.012034,(((r45Ppa:0.008841,
((r45Ppy:0.031251,r45Ggo:0.000000)N59:0.011047,hT2R45Hsa:0.024500)N58:0.003408)N57:0.004464,r45Pt
r:0.003282)N56:0.058912,(r44Pha:0.005793,r8Mmu:0.023760)N60:0.077547)N55:0.010823)N39:0.016573,r2
Ase:0.137313)N38:0.007781)N23:0.099958)N16:0.032477,(r15Ppy:0.114076,r3Ase:0.093098)N61:0.085480)
N15:0.017836,(((r49Mfa:0.000000,r49Mmu:0.003297)N64:0.081770,(R49Ppy:0.033202,((r49Nle:0.000000,r
49Hag:0.004812)N67:0.038571,((hT2R49Hsa:0.009796,r49Ptr:0.000000)N69:0.006503,(r49Ggo:0.006485,r4
9Pha:0.000000)N70:0.003235)N68:0.016848)N66:0.019077)N65:0.047209)N63:0.135170,((r48Ptr:0.002247,
(r48Ppa:0.006855,((hT2R48Hsa:0.013843,r48Ppy:0.068813)N75:0.006745,r48Ggo:0.003453)N74:0.013940)N
73:0.008167)N72:0.045313,((r64Ptr:0.013751,r64Ppa:0.038075)N77:0.033836,(r64Mmu:0.007863,(r48Mmu:
0.000000,r48Mfa:0.003506)N79:0.000000)N78:0.140523)N76:0.018743)N71:0.155982)N62:0.033634)N14:0.1
60901)N3:0.396003,(r10Ssc:0.119424,((((r10Aju:0.009088,r10Ppard:0.023109)N84:0.102579,(r10Ame:0.0
78258,((r10Vfe:0.000000,((r10Vvu:0.009335,r10Vco:0.003110)N89:0.000000,r10Vze:0.015646)N88:0.0000
00)N87:0.000000,((r10Cbr:0.000000,(r10Lpi:0.000000,r10Clu.fa:0.000000)N92:0.000000)N91:0.000000,r
10Clu:0.003118)N90:0.003106)N86:0.037394)N85:0.033015)N83:0.030665,((r10Cja:0.180247,((r10Pha:0.0
36329,r10Mmu:0.031718)N96:0.017238,((((hT2R10Hsa:0.016512,r10Ggo:0.000000)N100:0.006585,r10Ptr:0.
009879)N99:0.014367,r10Nle:0.028960)N98:0.012158,r10Ppy:0.031956)N97:0.017189)N95:0.073756)N94:0.
055627,((r107Rno:0.096614,r107Mmus:0.077382)N102:0.310528,(r7Sga:0.368648,(r106Mmus:0.062730,r106
Rno:0.112929)N104:0.234679)N103:0.037549)N101:0.166386)N93:0.069787)N82:0.069516,(r10Oar:0.158411
,r10Bta:0.103365)N105:0.157859)N81:0.037681)N80:0.570078)N2:0.337497,(t2r20Xtr:1.398145,(t2rAmi:1
.446799,((((v1r69Mmus:0.650163,v1r3Ptr:0.414979)N111:0.304212,v1r62Mmus:1.234586)N110:0.230333,(v
1r96Rno:1.045881,v1rMmu:1.117002)N112:0.113645)N109:0.423267,(v1rDre:0.534635,(v1rAmi:1.123651,v1
rXtr:0.566569)N114:0.199520)N113:0.174532)N108:0.495801)N107:1.162408)N106:0.337496)N1;
```

Phylogenetic Tree constructed using RaxML-HPC using 116 IG (Incl. Pseudogenes)

```
(((((((SlutungT2R15p_restored:0.07150222797375166417,((hT2R15p:0.01015052425440240470,BonoboPs8_r
estored:0.01702038238344613305):0.00820224024294758172,(BOrangPs8_restored:0.00000100000050002909
,R15BOrang:0.00000100000050002909):0.05809783389206447241):0.01777077934134023776):0.035016795095
88477568,r3Rhowler:0.09859838647681080925):0.07848375243369305754,((((R50BOrang:0.042958453739486
09797,((hT2R50:0.01391030734461921534,r50chimp:0.01041947601509846805):0.00694939060494071791,R50
gorilla:0.00000100000050002909):0.00303943074074589136):0.00655871698071374114,r50Ngibbon:0.01232
080709636736507):0.04017360503972346175,(r50CEmacaq:0.00349560226638169795,R50Rmacaq:0.0000010000
0050002909):0.05591489609364295316):0.18053085022030845863,((((R46CEmacaq:0.06078655259504672087,
(R46Hbaboon:0.10748383200889240208,R47Rmacaq:0.09525005789837784342):0.01618832923646499472):0.01
946286738285586682,((R47gorilla:0.00345357853041207115,(hT2R47:0.01243767243732391524,(R47chimp:0
.00000100000050002909,R47bonobo:0.00327946497687316412):0.01659506007938164537):0.003130759030630
16235):0.03218219418750779132,((R47Ngibbon:0.00975935918002159876,R47Agibbon:0.016903609352369345
50):0.02966235112319243541,R47BOrang:0.02473481888270688198):0.01098984287702932210):0.0633224718
8938075477):0.02366390370515329342,((R46gorilla:0.00984192770067276144,(R46chimp:0.02675631116712
215776,(R46bonobo:0.00000100000050002909,R66bonobo:0.06176493691763168536):0.00000100000050002909
):0.00328410072678927923):0.00800302278502447104,hT2R46:0.00847160409457877600):0.052131478042771
47579):0.01559181536411483396,(r2Rhowler:0.13347578913424676395,(((R44Hbaboon:0.00524715304775904
591,R8Rmacaq:0.02391877020275402430):0.07447351654223893780,((R45chimp:0.00325911227672499252,(R4
5bonobo:0.00878849059445775335,((R45BOrang:0.03100013412295514470,R45gorilla:0.000001000000500029
09):0.01095464553345949950,hT2R45:0.02428648756824151878):0.00337234139390125151):0.0044309479108
6957093):0.05159675735255470769,RmacaqR44p_restored:0.11638994366794212787):0.0087905814137443361
0):0.01421814538014497774,((((R44BOrang:0.02661382762106693015,R44Agibbon:0.01171002697804720410)
:0.01490336159585244069,(hT2R44:0.01987804094590779960,(R44gorilla:0.04002118566029148200,(R44chi
mp:0.00605617257058114426,44bonobo:0.00382629320221787280):0.00995469598402716309):0.003622299240
67895229):0.01874513469892316944):0.02704280852469627058,((R43bonobo:0.00984496702848441235,R43ch
imp:0.00325573756112506685):0.00000100000050002909,hT2R43:0.01306301671615711819):0.0434145380486
1493404):0.01813694003566038507,((R43Hbaboon:0.01346428840883857198,(R46Rmacaq:0.0000010000005000
```

2909,R43Rmacaq:0.00979730675577085257):0.02305212028889114598):0.07526942529884217414,(R45Slutung
:0.07381517455874367362,(R43Slutung:0.02809286574564690692,(R45Rmacaq:0.03309539895451422964,r43N
gibbon:0.03207915601168351977):0.04178562640171677722):0.00988763884644845367):0.0367540238832952
5316):0.01415537175532270188):0.00768139637523116217):0.01664248077123203426):0.01085238108728216
883):0.10359961049761676199):0.03147193978042144707):0.02182776687205807697,((BOrangT2R68p_restor
ed:0.14048854773522442829,((HbaboonT2R48p_restored:0.02038638227457107571,((R64Rmacaq:0.007787050
24408629914,r48Rmacaq:0.00000100000050002909):0.00000100000050002909,r48CEmacaq:0.00347107602627 1
37616):0.02233639669103579944):0.09704060635161168569,((r64chimp:0.00671179901000506542,(R64bonob
o:0.00694531206363778724,((BOrangT2R64p_restored:0.01089951636004734235,BOrangPs2_restored:0.0000
0100000050002909):0.06449506986374080320,hT2R64p:0.00333962628536393306):0.01273045668166887176):
0.03850795538412741387):0.03874699980333034394,(r48chimp:0.00000100000050002909,(R48bonobo:0.0067
7504046419503177,(R48gorilla:0.00342034316119968701,(R48BOrang:0.06805382527604715570,hT2R48:0.01
369598566253192075):0.00666456338642519141):0.01379290995267153791):0.01032768347776944450):0.045
70192451701141079):0.03831914526898507617):0.02796457978016956072):0.12256872169175980769,((R49BO
rang:0.03543254292669256039,(((hT2R49:0.00973565479578494299,r49chimp:0.00000100000050002909):0.0
0646626915293683092,(R49Hbaboon:0.00000100000050002909,R49gorilla:0.00644726053923489265):0.00321
605044916715771):0.01675619188708461077,(r49Ngibbon:0.00000100000050002909,R49Agibbon:0.004777648
02933130714):0.03822662019885521983):0.01675518161817857871):0.04491557344301820576,(r49CEmacaq:0
.00000100000050002909,r49Rmacaq:0.00327116130721204950):0.08308093013358479328):0.133887919347190
38168):0.03387044588631345915):0.14713871008043552524,((((R136Canis:0.03745428601942368818,CanisT
2R44p_restored:0.09030338045791860835):0.12392777490740494728,(r31panda:0.09249842330176809291,PA
NDA_014286:0.09572723049424873565):0.08803098937872605445):0.08511988719485048815,((r66Dbat:0.065
09724212098089158,r46Bbat:0.04269869022417253912):0.11171342455253266412,R45Bbat:0.16662265869301
476351):0.14609151355985686149):0.03684158792531351534,((r46cattle:0.01414137986257419169,r45Dyak
:0.00310023178676827049):0.25710371190829894861,(r31Dyak:0.16539221505638454501,(r31cattle:0.0000
0100000050002909,R46Dyak:0.00696560068831487186):0.14631698591591482672):0.09018590058987732694):
0.08589815347198533069):0.04897124683714125515):0.41314146948462898701,(pigT2R10:0.10711445241392
807126,((R10cattle:0.10338038986643162398,R10sheep:0.15864060940621993456):0.15691612109763677929
,(((R10CMarmos:0.18059024647703367239,((R10Rmacaq:0.03175454284787231074,R10HBaboon:0.03642073647
171084616):0.01719592672372479911,((R10NGibbon:0.02900741194163944034,((hT2R10:0.0165177009544224
2278,R10gorilla:0.00000100000050002909):0.00658781304170116561,R10chimp:0.00988135816308866738):0
.01438344548858992537):0.01213840057000728674,R10BOrang:0.03197458663271914719):0.017247481840486
03347):0.07373858512144433608):0.05571324392503729933,((T7Gmolerat:0.36857468067126825106,(R106Ra
t:0.11341511197441750403,R106Mus:0.06238952024579857286):0.23493184783720877373):0.03768238685182
930675,(R107Mus:0.07744693653124083699,R107Rat:0.09663036147815666499):0.31063295709731947314):0.
16689856000348859966):0.06907818448195353345,((R10panda:0.07835646550853755943,(((ManedWolfR10:0.
00000100000050002909,(R10Canis:0.00000100000050002909,HuntingDogR10:0.00000100000050002909):0.000
00100000050002909):0.00000100000050002909,WolfR10:0.00311759602032315010):0.00310689939071742650,
(TibetanFoxR10:0.00000100000050002909,(FennecFoxR10:0.01565123233544761863,(CorsacFoxR10:0.003110
99387998588000,RedFoxR10:0.00933857833750615686):0.00000100000050002909):0.00000100000050002909):
0.00000100000050002909):0.03710106962736015729):0.03316778003676362080,(PantheraPardusR10:0.02307
473981902258275,AcinonyxJubatusR10:0.00906414935133559134):0.10227019896152164669):0.030967625059
05431562):0.07004598101264550292):0.04920340145663984477):0.56756524833833343902):0.3330121335288
1725571,((T2R_alligator:1.36566472999328269999,(((v1r_RMacaq:1.08724906144424671162,V1r96_rat:1.0
1967331265949101571):0.13358576931292459600,(V1r62_mus:1.21984417129865052587,(V1r69_mus:0.651967
44666521755374,v1r3_chimp:0.41277570843704280978):0.31473892877985371053):0.22334339246918261113)
:0.41239362098587217798,((V1R_alligator:1.13130323939869392724,v1r_frog:0.55092190261117968131):0
.20587564410974371065,V1r_Zebrafish:0.52663377269428857108):0.21002175219399132899):0.52018348478
339015983):1.08316431790780498545,T2R20_frog:1.38605639396150248643):0.33301213352881725571);

Phylogenetic Tree with Reconstructed Ancestral Sequences using FastML - 116IG, Incl. Pseudogenes

(((((((SlutungT2R15p_restored:0.072563,((hT2R15p:0.010313,BonoboPs8_restored:0.017291)N9:0.008342
,(BOrangPs8_restored:0.000000,R15BOrang:0.000000)N10:0.058991)N8:0.018001)N7:0.035651,r3Rhowler:0
.100259)N6:0.079975,((((R50BOrang:0.043632,((hT2R50:0.014133,r50chimp:0.010586)N16:0.007060,R50go
rilla:0.000000)N15:0.003121)N14:0.006642,r50Ngibbon:0.012498)N13:0.040885,(r50CEmacaq:0.003553,R5
0Rmacaq:0.000000)N17:0.056742)N12:0.183292,((((R46CEmacaq:0.061737,(R46Hbaboon:0.109090,R47Rmacaq
:0.096634)N22:0.016387)N21:0.019795,((R47gorilla:0.003503,(hT2R47:0.012612,(R47chimp:0.000000,R47
bonobo:0.003326)N26:0.016832)N25:0.003164)N24:0.032624,((R47Ngibbon:0.009907,R47Agibbon:0.017146)
N28:0.030127,R47BOrang:0.025072)N27:0.011150)N23:0.064264)N20:0.023895,((R46gorilla:0.009977,(R46
chimp:0.027145,(R46bonobo:0.000000,R66bonobo:0.062700)N32:0.000000)N31:0.003330)N30:0.008031,hT2R
46:0.008674)N29:0.052961)N19:0.016093,(r2Rhowler:0.135245,(((R44Hbaboon:0.005316,R8Rmacaq:0.02428
0)N36:0.075544,((R45chimp:0.003307,(R45bonobo:0.008912,((R45BOrang:0.031422,R45gorilla:0.000000)N
41:0.011109,hT2R45:0.024661)N40:0.003431)N39:0.004497)N38:0.052377,RmacaqR44p_restored:0.118240)N
37:0.008809)N35:0.014408,((((R44BOrang:0.026967,R44Agibbon:0.011861)N45:0.015121,(hT2R44:0.020152
,(R44gorilla:0.040577,(R44chimp:0.006141,44bonobo:0.003880)N48:0.010098)N47:0.003673)N46:0.019002
)N44:0.027421,((R43bonobo:0.009979,R43chimp:0.003301)N50:0.000000,hT2R43:0.013244)N49:0.044110)N4

3:0.018477,((R43Hbaboon:0.013664,(R46Rmacaq:0.000000,R43Rmacaq:0.009940)N53:0.023387)N52:0.076358
,(R45Slutung:0.074887,(R43Slutung:0.028476,(R45Rmacaq:0.033605,r43Ngibbon:0.032497)N56:0.042421)N
55:0.010058)N54:0.037355)N51:0.014207)N42:0.007848)N34:0.016954)N33:0.010831)N18:0.105211)N11:0.0
31897)N5:0.022318,((BOrangT2R68p_restored:0.142639,((HbaboonT2R48p_restored:0.020721,((R64Rmacaq:
0.007907,r48Rmacaq:0.000000)N62:0.000000,r48CEmacaq:0.003530)N61:0.022703)N60:0.098581,((r64chimp
:0.006849,(R64bonobo:0.007069,((BOrangT2R64p_restored:0.011055,BOrangPs2_restored:0.000000)N67:0.
065475,hT2R64p:0.003391)N66:0.012916)N65:0.039084)N64:0.039283,(r48chimp:0.000000,(R48bonobo:0.00
6885,(R48gorilla:0.003475,(R48BOrang:0.069101,hT2R48:0.013912)N71:0.006778)N70:0.014009)N69:0.010
490)N68:0.046536)N63:0.038984)N59:0.028197)N58:0.124917,((R49BOrang:0.036059,(((hT2R49:0.009878,r
49chimp:0.000000)N76:0.006559,(R49Hbaboon:0.000000,R49gorilla:0.006540)N77:0.003262)N75:0.016979,
(r49Ngibbon:0.000000,R49Agibbon:0.004844)N78:0.038768)N74:0.016867)N73:0.045822,(r49CEmacaq:0.000
000,r49Rmacaq:0.003321)N79:0.084192)N72:0.135632)N57:0.034249)N4:0.149469,((((R136Canis:0.037931,
CanisT2R44p_restored:0.091372)N83:0.125542,(r31panda:0.093824,PANDA_014286:0.097289)N84:0.089436)
N82:0.086034,((r66Dbat:0.065918,r46Bbat:0.043348)N86:0.113559,R45Bbat:0.169207)N85:0.148462)N81:0
.037000,((r46cattle:0.014438,r45Dyak:0.003072)N88:0.260873,(r31Dyak:0.167891,(r31cattle:0.000000,
R46Dyak:0.007073)N90:0.148580)N89:0.091747)N87:0.086936)N80:0.049905)N3:0.417419,(pigT2R10:0.1085
57,((R10cattle:0.104721,R10sheep:0.161230)N93:0.159113,(((R10CMarmos:0.183045,((R10Rmacaq:0.03219
1,R10HBaboon:0.036907)N98:0.017482,((R10NGibbon:0.029403,((hT2R10:0.016749,R10gorilla:0.000000)N1
02:0.006681,R10chimp:0.010021)N101:0.014567)N100:0.012336,R10BOrang:0.032416)N99:0.017436)N97:0.0
74989)N96:0.056235,((T7Gmolerat:0.374384,(R106Rat:0.114995,R106Mus:0.063312)N105:0.238242)N104:0.
037682,(R107Mus:0.078230,R107Rat:0.098404)N106:0.315364)N103:0.169905)N95:0.070373,((R10panda:0.0
79663,(((ManedWolfR10:0.000000,(R10Canis:0.000000,HuntingDogR10:0.000000)N112:0.000000)N111:0.000
000,WolfR10:0.003164)N110:0.003152,(TibetanFoxR10:0.000000,(FennecFoxR10:0.015876,(CorsacFoxR10:0
.003156,RedFoxR10:0.009473)N115:0.000000)N114:0.000000)N113:0.000000)N109:0.037479)N108:0.033483,
(PantheraPardusR10:0.023421,AcinonyxJubatusR10:0.009184)N116:0.104117)N107:0.031468)N94:0.070891)
N92:0.050543)N91:0.575726)N2:0.335909,((T2R_alligator:1.395138,(((v1r_RMacaq:1.097785,V1r96_rat:1
.031576)N121:0.135238,(V1r62_mus:1.239197,(V1r69_mus:0.662330,v1r3_chimp:0.418335)N123:0.317172)N
122:0.226676)N120:0.416326,((V1R_alligator:1.151741,v1r_frog:0.562581)N125:0.208164,V1r_Zebrafish
:0.535519)N124:0.211454)N119:0.525967)N118:1.098347,T2R20_frog:1.401225)N117:0.335908)N1;

Phylogenetic trees tested using different IG and OG samplings
Same IGs (55% identity cutoff); 5OG (V1r_Zebrafish, V1R_alligator, V1r69_mus, V1r96_rat, v1r_RMacaq)

(((((((r49CEmacaq:0.00000100000050002909,r49Rmacaq:0.00335494963839445609):0.08294212534171366280
,(R49BOrang:0.03590111934298397672,(((hT2R49:0.00997487813610501914,r49chimp:0.000001000000500029
09):0.00662337847395584600,(R49Hbaboon:0.00000100000050002909,R49gorilla:0.00660494989858682966):
0.00329557083909278077):0.01716176935334691803,(r49Ngibbon:0.00000100000050002909,R49Agibbon:0.00
489886156672690240):0.03925681660212126250):0.01745365315967207370):0.04928051071073205613):0.138
62647489544324686,(BOrangT2R68p_restored:0.14153431342693231776,((r48chimp:0.00265277982473241048
,(R48bonobo:0.00698224450697568656,((R48BOrang:0.06974484803162879498,hT2R48:0.01405368039650164470):0.00683495642541405175,R48gorilla:0.00350532318617745786):0.01412364232981187274):0.007937545
17850855067):0.06918698889345058223,(((R64Rmacaq:0.00797083428495500186,(r48CEmacaq:0.00356103351
476436255,r48Rmacaq:0.00000100000050002909):0.00000100000050002909):0.02565611593479319441,Hbaboo
nT2R48p_restored:0.01820187221716683054):0.09031061811449783705,(((r64chimp:0.0464476519307577689
9,R64bonobo:0.00685056093528561057):0.01251045328441420870,hT2R64p:0.00437798203077353985):0.0198
4463303797072073,(BOrangPs2_restored:0.00000100000050002909,BOrangT2R64p_restored:0.0111692596074
5748098):0.04845421029580748024):0.03465811137533313796):0.02177450687665588580):0.0252721033550419
8255):0.13252261056254893701):0.03483414431226161678,((((r50CEmacaq:0.00358426605821144913,R50R
macaq:0.00000100000050002909):0.05749245422165258357,(r50Ngibbon:0.01253048637564966976,((R50gori
lla:0.00000100000050002909,(hT2R50:0.01427083274171516043,r50chimp:0.01069544261840828236):0.0071
2694360970200086):0.00314277116358713813,R50BOrang:0.04404767335659470762):0.00678946025826059930
):0.04091399199772738360):0.18830431114474280307,((((R46CEmacaq:0.06257077681816471115,(R47Rmacaq
:0.09779900750442097057,R46Hbaboon:0.10976635306504467504):0.01663450436610759697):0.020062991758
82077235,((R47gorilla:0.00354435228801784977,(hT2R47:0.01273143488773018951,(R47chimp:0.000001000
00050002909,R47bonobo:0.00335539655868646695):0.01700601629394690381):0.00319504939544760754):0.0
3289088773846923081,((R47Agibbon:0.01727676923462008116,R47Ngibbon:0.00997707772179756355):0.0304
1070450024808275,R47BOrang:0.02531051733708762283):0.01132389189984606657):0.06407660596630529448
):0.02422238959091345400,((R46gorilla:0.01006375228059063105,(R46chimp:0.02738620757913442713,(R6
6bonobo:0.06322843238017933354,R46bonobo:0.00000100000050002909):0.00000100000050002909):0.003357
78528148187368):0.00823856371083282572,hT2R46:0.00860844865413712933):0.05291419832565094628):0.0
1396566337627725904,((((((R44gorilla:0.04095757433941690784,(R44chimp:0.00619752205699227246,44bo
nobo:0.00390917431113033034):0.01018974635928644561):0.00366841813932468125,hT2R44:0.020351510213
16115513):0.01911184107214661948,(R44BOrang:0.02727017571898646131,R44Agibbon:0.01197377033141401
419):0.01527986417830007153):0.02776278578802153507,(R43bonobo:0.01007754760421518576,(hT2R43:0.0
1337135259951803719,R43chimp:0.00333218497629729883):0.00000100000050002909):0.04402773217485449891):0.02000871071764188588,((((R44Hbaboon:0.00498385201548374752,R8Rmacaq:0.02461577389652467446)

:0.07736001345838942889,RmacaqR44p_restored:0.11658811902995877552):0.00369542589935805766,((R45b
onobo:0.00899053763609488840,(hT2R45:0.02489373656291922129,(R45BOrang:0.03170568873416287903,R45
gorilla:0.00000100000050002909):0.01119250024011948265):0.00347033817387982276):0.004528371696629
53683,R45chimp:0.00333836482762190365):0.05453030491066541974):0.01755823847682648486,((R43Hbaboo
n:0.01047456828406119998,(R46Rmacaq:0.00000100000050002909,R43Rmacaq:0.01002717996659188184):0.02
689407009761695180):0.07870328445985158572,(R45Slutung:0.07125272930922202375,(R43Slutung:0.03149
064565601179810,(r43Ngibbon:0.03619877014329685866,R45Rmacaq:0.03043785391489207612):0.0434068199
2955484902):0.00926768223800537687):0.03475831049525574673):0.01349052981984976383):0.01041899164
938660596):0.01538002910060664967,r2Rhowler:0.13533317885846887774):0.01415609766954686009):0.105
43574681807470206):0.03280989911950592947,(r3Rhowler:0.10196305595191886006,(SlutungT2R15p_restor
ed:0.07503575749188305666,((BonoboPs8_restored:0.01746127837632336158,hT2R15p:0.01041267076243709
844):0.00842981630182098804,(BOrangPs8_restored:0.00000100000050002909,R15BOrang:0.00000100000050
002909):0.05959237998196011826):0.01666521470297758004):0.03685657998101843358):0.079033994457696
72405):0.01937050903485565759):0.14004034980226737206,(((R45Bbat:0.17087655472267029855,(r66Dbat:
0.06665071085079483926,r46Bbat:0.04398259921676806600):0.11533010563850469576):0.1486549286961785
7874,((CanisT2R44p_restored:0.09237437136679704786,R136Canis:0.03792362530714397245):0.1264696687
7144028008,(r31panda:0.09474580551218970970,PANDA_014286:0.09814291707556391242):0.09038126185191
200368):0.09002255550120934202):0.03349584527350462498,((r45Dyak:0.00307836697269762765,r46cattle
:0.01458763969859750917):0.26983194931526566229,(r31Dyak:0.16595716089565065721,(r31cattle:0.0000
0100000050002909,R46Dyak:0.00716020668572055902):0.15276652241763477580):0.08767416051554476064):
0.09045046418337358840):0.06586204393594431716):0.56823676837245462590,((((R106Mus:0.065407139624
76790710,R106Rat:0.11286507942562855333):0.24255506364385673312,T7Gmolerat:0.37841571013805569423
):0.04149598111276908696,(R107Mus:0.07843482447221997167,R107Rat:0.09852614460820510733):0.316444
01823253337502):0.11972407402882760674,((R10CMarmos:0.18198562341909110818,((R10HBaboon:0.0370417
1390703368816,R10Rmacaq:0.03200346079985067621):0.01733662321411386098,((((hT2R10:0.0167240227435
9945790,R10gorilla:0.00000100000050002909):0.00666723669534044234,R10chimp:0.01000337519467440692
):0.01457157501166667785,R10NGibbon:0.02938668686393858948):0.01213475547614099002,R10BOrang:0.03
252780087165724199):0.01758966917273674033):0.07523653964659246518):0.04795033019141432817,((R10s
heep:0.18075627380434594604,R10cattle:0.09203529778142820195):0.19965264046076716675,(((WolfR10:0
.00316402906526221540,(ManedWolfR10:0.00000100000050002909,(HuntingDogR10:0.00000100000050002909,
R10Canis:0.00000100000050002909):0.00000100000050002909):0.00000100000050002909):0.00315515406513
064336,(TibetanFoxR10:0.00000100000050002909,((CorsacFoxR10:0.00315821144642048452,RedFoxR10:0.00
948529970763747378):0.00000100000050002909,FennecFoxR10:0.01590125076629602188):0.000001000000500
02909):0.00000100000050002909):0.02674379304235904170,R10panda:0.09090192753735205911):0.08675473
201112200716):0.06223686342310691400):0.06086713010804659096):0.42496368045483812192):1.102929540
61552750389,((V1r_Zebrafish:0.67281576148426802675,V1R_alligator:1.09032500883176775019):0.171855
29398519067135,(V1r69_mus:1.04390776106286198299,(V1r96_rat:1.07089045807571281443,v1r_RMacaq:1.1
2359420364583462160):0.10859062458177043409):0.36184252562175223122):1.10292954061552750389);

Same IGs (55% identity cutoff); OG = V1r69_mus, V1r62_mus

((((T7Gmolerat:0.40460049429257449738,((R106Rat:0.11569897238169855880,R106Mus:0.0609157869667477
3002):0.25636132147651835567,(R107Mus:0.07663572833395471129,R107Rat:0.09934754852785689094):0.30
968016242591722698):0.03165342852005314372):0.09303725231717713262,(((R10panda:0.0938835669497825
1815,((((CorsacFoxR10:0.00312126370423203466,RedFoxR10:0.00937403895753414693):0.0000010000005000
2909,FennecFoxR10:0.01570472312163739836):0.00000100000050002909,TibetanFoxR10:0.0000010000005000
2909):0.00000100000050002909,(WolfR10:0.00312876635669616160,(ManedWolfR10:0.00000100000050002909
,(R10Canis:0.00000100000050002909,HuntingDogR10:0.00000100000050002909):0.00000100000050002909):0
.00000100000050002909):0.00311798388039987839):0.02244877323868816818):0.08396641121134942876,(R1
0sheep:0.17913529221405105618,R10cattle:0.09159301200373033691):0.19891334873759267121):0.0622864
2825558720203,(R10CMarmos:0.18462171062842624991,((R10Rmacaq:0.03152420111663671587,R10HBaboon:0.
03652934624104517414):0.01690184575282074539,((((hT2R10:0.01649757373427866713,R10gorilla:0.00000
100000050002909):0.00658143397318231797,R10chimp:0.00986577841388321976):0.01437140073443935254,R
10NGibbon:0.02898575687941167683):0.01180986762998431985,R10BOrang:0.03234048975680698518):0.0176
8422448323528351):0.07057636936990450971):0.05262051402659257554):0.08685411843835540158):0.55936
222630714893533,((((BOrangT2R68p_restored:0.14133459982059526361,((HbaboonT2R48p_restored:0.02063
814436742553854,(r48CEmacaq:0.00350410401440746314,(R64Rmacaq:0.00779442152308524715,r48Rmacaq:0.
00000100000050002909):0.00000100000050002909):0.02253356543842291448):0.09931261464044970066,(((R
64bonobo:0.00699806485654421752,(hT2R64p:0.00336773607585132573,(BOrangT2R64p_restored:0.01095880
686401124772,BOrangPs2_restored:0.00000100000050002909):0.06530230865857775124):0.012848440111850
80730):0.03927144947100533884,r64chimp:0.00676866532694185823):0.03931140603549657803,(r48chimp:0
.00000100000050002909,(R48bonobo:0.00683512952471765096,((R48BOrang:0.06892685777039546380,hT2R48
:0.01383283810448998445):0.00673963144381329281,R48gorilla:0.00346075391549281438):0.013949184652
77457213):0.01045440506932795652):0.04628997042560771547):0.03835204825040285093):0.0284750980825
6737038):0.12453937686378326044,((r49Rmacaq:0.00330688851581178393,r49CEmacaq:0.00000100000050002
909):0.08264517288589832977,(R49BOrang:0.03566856675388793696,((r49Ngibbon:0.00000100000050002909
,R49Agibbon:0.00435167210090179387):0.03473133021523688163,((hT2R49:0.00984153177153494242,r49chi

mp:0.00000100000050002909):0.00653351618286638746,(R49Hbaboon:0.00000100000050002909,R49gorilla:0
.00651362228989392673):0.00325129815791668376):0.01695984814470295407):0.01708465344159517535):0.
04678903907974114873):0.13680153133460037918):0.03353373288660073392,(((((hT2R46:0.00852394244433
934446,(R46gorilla:0.00989997961250071307,(R46chimp:0.02695079340983908001,(R46bonobo:0.000001000
00050002909,R66bonobo:0.06221964113330556018):0.00000100000050002909):0.00330346887096192188):0.0
0805367315034921302):0.05218761638291597588,(((R47BOrang:0.02485068723451496278,(R47Ngibbon:0.009
76215263987988482,R47Agibbon:0.01698343101973694494):0.02985772921768290594):0.01122252083433687
31,(R47gorilla:0.00349115953848028225,((R47chimp:0.00000100000050002909,R47bonobo:0.0032998971248
0688053):0.01671229102223947133,hT2R47:0.01250920867694607988):0.00313004058685827308):0.03222461
713288281987):0.06312405234596195347,(R46CEmacaq:0.06188406589744088748,(R47Rmacaq:0.096039080397
18954404,R46Hbaboon:0.10819709322186760181):0.01627294543410417668):0.01967902128100348230):0.023
87018015308035868):0.01382713073897025476,(r2Rhowler:0.13254731580343304120,((((hT2R44:0.02005325
057797903432,(R44gorilla:0.04035557495481630758,(R44chimp:0.00609508689118632655,R44bonobo:0.0038
5827901662936764):0.01001351133183522868):0.00363144071977829436):0.01883756749959278678,(R44Agib
bon:0.01176399785415945150,R44BOrang:0.02688117958157542828):0.01502529393053542855):0.0273694667
4782500533,(R43bonobo:0.00990976404461932532,(R43chimp:0.00327625640379433040,hT2R43:0.0131487159
2588316013):0.00000100000050002909):0.04331725784837053839):0.01971915881872430068,(((R45chimp:0.
0032878617220691874 2,(R45bonobo:0.00884606279051950058,(hT2R45:0.02448074582380741632,(R45BOrang:
0.03110328511565053564,R45gorilla:0.00000100000050002909):0.01099749072754508360):0.0034117743816
9008472):0.00445709233799423954):0.05363114087785236100,(RmacaqR44p_restored:0.114823086960144801
76,(R8Rmacaq:0.02407222750758613097,R44Hbaboon:0.00491883167002941848):0.07608842680421956661):0.
0036031494874178837 7):0.01733395432737167702,((R43Hbaboon:0.01032366628310770612,(R46Rmacaq:0.000
00100000050002909,R43Rmacaq:0.00985241133229227545):0.02642263561353041038):0.0776083989515638089
7,(R45Slutung:0.06993519551335761197,(R43Slutung:0.03087350662877861793,(r43Ngibbon:0.03539983665
794128181,R45Rmacaq:0.02994335819952776562):0.04262692916938178034):0.00918899013898403019):0.034
18311402705601076):0.01326704876525585351):0.01021406837875001201):0.01520913509971714636):0.0138
7456870331920528):0.10645404167973145093,((r50CEmacaq:0.00352875834789785755,R50Rmacaq:0.00000100
000050002909):0.05716161573682091573,(r50Ngibbon:0.01236110769441281163,(R50BOrang:0.043419958477
53576249,(R50gorilla:0.00000100000050002909,(r50chimp:0.01051511184804161322,hT2R50:0.01402900585
402305951):0.00701368379323764196):0.00305704242985594971):0.00658967247104380247):0.039936324108
31150087):0.18551296855268026298):0.03134692930416138612,(r3Rhowler:0.09912834153100695544,(Slutu
ngT2R15p_restored:0.07263410736739768503,((R15BOrang:0.00000100000050002909,BOrangPs8_restored:0.
00000100000050002909):0.05858383288573836001,(BonoboPs8_restored:0.01714003910365255109,hT2R15p:0
.01022138157276187079):0.00829787694857971915):0.01720867263934808614):0.03580898450392484833):0.
07952471910664432597):0.02210400604981465136):0.12291920754304609675,(((R45Bbat:0.168165154930195
34855,(r46Bbat:0.04316831760778497284,r66Dbat:0.06570200015121294146):0.11421387766677659759):0.1
5066041096049748171,((CanisT2R44p_restored:0.08504436584151353618,R136Canis:0.0553434132604036510
5):0.10896171257169931512,(r31panda:0.08978267893555637313,PANDA_014286:0.09767987885479297028):0
.08687589567275853797):0.08661096225511483171):0.03408868660397581379,(((r31cattle:0.000001000000
50002909,R46Dyak:0.00701522487671921331):0.15104836519846023823,r31Dyak:0.16364645912728723043):0
.08680888012171122936,(r46cattle:0.01421124253518209375,r45Dyak:0.00313965819946455862):0.2687787
0437948742088):0.09328031025470602533):0.07227126621495433278):0.51492373132882129916):1.17709406
608983324638,(V1r62_mus:1.09920907810662837889,V1r69_mus:0.87086375380038238969):1.17709406608983
324638);

55% identity cutoff; OG = alligator T2R & V1R

(((((R107Mus:0.07714779588406359134,R107Rat:0.09755788429253262062):0.30989125543937412477,(T7Gmo
lerat:0.38760758046752080697,(R106Rat:0.11401512506409654246,R106Mus:0.06220913785791390427):0.24
764866370700583387):0.03795688665790183935):0.10481871227098372990,(((R10panda:0.0929461923164651
1492,((((CorsacFoxR10:0.00310696691875666934,RedFoxR10:0.00932962352137242830):0.0000010000005000
2909,FennecFoxR10:0.01563299902052484036):0.00000100000050002909,TibetanFoxR10:0.0000010000005000
2909):0.00000100000050002909,((ManedWolfR10:0.00000100000050002909,(HuntingDogR10:0.0000010000005
0002909,R10Canis:0.00000100000050002909):0.00000100000050002909):0.00000100000050002909,WolfR10:0
.00311411839073560395):0.00310345643289602641):0.02277624007960177277):0.08221450013727919781,(R1
0sheep:0.17854397515673803287,R10cattle:0.09111367656404774362):0.19991727051361107836):0.0632453
1746570648261,(((R10HBaboon:0.03639322133767570328,R10Rmacaq:0.03123663267912774738):0.0170313580
5177199980,(R10BOrang:0.03223233277502211835,(((hT2R10:0.01642633361561801272,R10gorilla:0.000001
00000050002909):0.00655076517484783679,R10chimp:0.00982416592437072335):0.01432135445942701348,R1
0NGibbon:0.02884638209423210156):0.01172105154846331439):0.01734523613458963803):0.07054133260569
302177,R10CMarmos:0.18357156106643440774):0.05087954708253641933):0.07567331441287719618):0.64922
704121732588423,((((r45Dyak:0.00306416738656054618,r46cattle:0.01422035197266707102):0.2665909115
8372261177,((R46Dyak:0.00700578861918240391,r31cattle:0.00000100000050002909):0.15037287244314706
536,r31Dyak:0.16342644303282308216):0.08700399252423791918):0.09306623021011634078,(((R136Canis:0
.05507781949213021166,CanisT2R44p_restored:0.08473065698407301893):0.10811438792160642242,(PANDA_
014286:0.09740671344939878795,r31panda:0.08915923364151639996):0.08693519788903712742):0.08672962
911113271667,(R45Bbat:0.16789502636159134297,(r46Bbat:0.04301413675261495789,r66Dbat:0.0652730555

0006737289):0.11336238294787698289):0.15042465832787363378):0.03373827313111835147):0.06638099148
595134891,((((r49Rmacaq:0.00329273912587091811,r49CEmacaq:0.00000100000050002909):0.0823959715976
8148604,(R49BOrang:0.03552395059412750272,((r49Ngibbon:0.00000100000050002909,R49Agibbon:0.004323
18687830225961):0.03453257604352692051,((hT2R49:0.00978579056942068329,r49chimp:0.000001000000500
02909):0.00649863681014827260,(R49gorilla:0.00647822476455237137,R49Hbaboon:0.0000010000005000290
9):0.00323366641715515466):0.01686050417387834066):0.01696216594763312255):0.04653375357354824771
):0.13612607999215309285,(BOrangT2R68p_restored:0.14093707898968804715,(((r48chimp:0.000001000000
50002909,((R48gorilla:0.00344487195274283987,(R48BOrang:0.06855477391102031415,hT2R48:0.013774829
36416369631):0.00670156561963881816):0.01388043246108100308,R48bonobo:0.00680748422368389946):0.0
1040191523212827610):0.04601026817437339073,(r64chimp:0.00672789753006612038,((hT2R64p:0.00335022
183014463707,(BOrangPs2_restored:0.00000100000050002909,BOrangT2R64p_restored:0.01090783971330665
583):0.06508251904384355913):0.01280360645373772484,R64bonobo:0.00696824810541874905):0.039122288
32202307299):0.03914086686005239374):0.03806877169140408107,(HbaboonT2R48p_restored:0.02054412695
564450039,((r48CEmacaq:0.00349110349932879767,r48Rmacaq:0.00000100000050002909):0.000001000000500
02909,R64Rmacaq:0.00779630964325886151):0.02246293624472475020):0.09898694058819107466):0.0283454
173917071158):0.12348962149906056451):0.03401935962167305233,((r3Rhowler:0.09883104949248554261,
(((BOrangPs8_restored:0.00000100000050002909,R15BOrang:0.00000100000050002909):0.0582324230732061
2710,(BonoboPs8_restored:0.01705275673782995563,hT2R15p:0.01016693662490769992):0.00827166967401467106):0.01696344253251066622,SlutungT2R15p_restored:0.07256922172836841045):0.03552680622282303885):0.07920914415905150330,(((r50CEmacaq:0.00351490225678579302,R50Rmacaq:0.00000100000050002909)
:0.05674961712560584737,((((r50chimp:0.01045645691395530492,hT2R50:0.01395182379255978898):0.0069
755627003266 2247,R50gorilla:0.00000100000050002909):0.00304045225297845017,R50BOrang:0.0431971048
4780946158):0.00656112709568858286,r50Ngibbon:0.01233405123927947163):0.03989385487134706337):0.1
8497727079627165891,((r2Rhowler:0.13200710425619666122,(((R43bonobo:0.00986598412797760026,(hT2R4
3:0.01308541532582442775,R43chimp:0.00326057990521735072):0.00000100000050002909):0.0430859099814
8779938,((R44BOrang:0.02676782843367415260,R44Agibbon:0.01172751801945333752):0.01498388700864369
816,(hT2R44:0.01997080761745447658,((R44bonobo:0.00385248643320432833,R44chimp:0.0060604494015896
4335):0.00997510366223053682,R44gorilla:0.04018216572538545889):0.00362554525684126655):0.0187469
0432386382291):0.02721577848000015371):0.01964359190369862826,((((R46Rmacaq:0.0000010000005000290
9,R43Rmacaq:0.00980966880810356899):0.02630902650359102923,R43Hbaboon:0.01028471325765683425):0.0
7722004930666935552,(R45Slutung:0.06953059294556634962,(R43Slutung:0.03065555794954492874,(R45Rma
caq:0.02981330812894915708,r43Ngibbon:0.03527850588743875609):0.04241273763106142430):0.009195829
03444133258):0.03401551888934724321):0.01320093844024581957,((R45chimp:0.00326814651832308322,(R4
5bonobo:0.00880222227875436178,((R45gorilla:0.00000100000050002909,R45BOrang:0.030987839365454454
55):0.01091120442866598192,hT2R45:0.02431513964551098098):0.00340981146889378535):0.0044306969088
7357148):0.05338748295689591666,((R8Rmacaq:0.02407156308350498486,R44Hbaboon:0.00488037069692062817):0.07579634310042912548,RmacaqR44p_restored:0.11437295400227601161):0.00356882732554886662):0.
01722258566206139388):0.01018350914533412699):0.01519306106041470872):0.01373040207799158460,((hT
2R46:0.00846185079377343406,(R46gorilla:0.00985320441001878190,((R66bonobo:0.06195830466498090244
,R46bonobo:0.00000100000050002909):0.00000100000050002909,R46chimp:0.02682061878015141662):0.0032
8681514184620471):0.00803894829759026035):0.05194178466615301942,((((R47Ngibbon:0.009738485736467
50142,R47Agibbon:0.01689575126808591163):0.02969796200859732560,R47BOrang:0.02475035407857625880)
:0.01119527585618388527,(R47gorilla:0.00348172816507930603,(hT2R47:0.01245903655165497143,(R47chi
mp:0.00000100000050002909,R47bonobo:0.00328678791405294076):0.01664520872693446216):0.00311484538
166936992):0.03207146834595006601):0.06276977955087399696,(R46CEmacaq:0.06155975973040700433,(R46
Hbaboon:0.10768856043988091209,R47Rmacaq:0.09590753332370380302):0.01613145399250309059):0.019700
63841565064533):0.02368733633518447720):0.01381397016588668120):0.10557498906046892950):0.0312253
579326051480 8):0.02096794572630003572):0.12687148642830911593):0.41184408853160031638):0.45178228
509952839120,(V1R_alligator:1.93077790060452070264,T2R_alligator:1.60262076850408119455):0.451782
28509952839120);
23 T2R10-like seqs, 138 T2R46-like seqs (45%+ identity cutoff), 14 hTAS2Rs, 5OG
((((((R20Dyak:0.06300720346225718860,(R13moose:0.05054584751327313719,(R13Dama:0.0073382507945801
9693,R13rdeer:0.00000100000050002909):0.01393748794558778530):0.02509782493009181034):0.487421383
13008631645,(((((r46cattle:0.01407169943756868442,r45Dyak:0.00280766522477034208):0.2635135280669
9646081,(r31Dyak:0.16194234647564323404,(R46Dyak:0.00682771276183098685,r31cattle:0.0000010000005
0002909):0.14678834624966585753):0.08447171654546573039):0.09843141349640123494,(((r66Dbat:0.0641
248861611093817 8,r46Bbat:0.04152149597453755336):0.10436039612326271364,R45Bbat:0.171718598719643
03967):0.14240789598188269283,((r31panda:0.09274872663275163243,PANDA_014286:0.090507493600105043
41):0.08873889907568559277,R136Canis:0.15566876656760814113):0.09801924224250181672):0.0263429222
3075063166):0.04615066320220514995,(((R136mus:0.18695565593434987361,R136rat:0.124328453102323530
09):0.37012873731252776688,(R120mus:0.03333474165372733722,R120rat:0.16221556211614585052):0.4511
355024319326223 5):0.14779113159760984275,(((((R64Rmacaq:0.00763948440647129867,(r48Rmacaq:0.00000
100000050002909,r48CEmacaq:0.00339689091078993746):0.00000100000050002909):0.11653877339848423000
,((r19chimp:0.00000100000050002909,(R48bonobo:0.00664788463160125454,(R48gorilla:0.00336960200438
393004,(R48BOrang:0.06645936701265182933,hT2R48:0.01342019798222628124):0.00653613054230726270):0
.01350686687210234452):0.01013274025470078943):0.04758518537660446496,(R64bonobo:0.04608621265310

312853,r64chimp:0.00463077135603858023):0.03390019098283104243):0.03791718906241425824):0.1408469
9253135107844,((r49CEmacaq:0.00000100000050002909,r49Rmacaq:0.00320011190063296085):0.08016879337
205747025,(R49BOrang:0.03453319673021982422,(((R49gorilla:0.00628285560950837992,R49Hbaboon:0.000
00100000050002909):0.00313353741821020826,(r49chimp:0.00000100000050002909,(r20chimp:0.0031397188
1039382898,hT2R49:0.00948872716874371407):0.00000100000050002909):0.00630884795219852773):0.01633
220392393564327,(R49Agibbon:0.00428292092697392986,r49Ngibbon:0.00000100000050002909):0.034297228
70828133541):0.01648499958771566451):0.04473025513698994371):0.12099544171214704491):0.0389324659
6715446012,((((r2Rhowler:0.13316344814591823953,(((R44Hbaboon:0.00528903658039743194,R8Rmacaq:0.0
2324911960469374511):0.07443011254211941152,((R45bonobo:0.00851456361788900142,((R45BOrang:0.0306
8355372166376230,R45gorilla:0.00000100000050002909):0.01080033029551791257,hT2R45:0.0238024643706
5445739):0.00334437949887351170):0.00430665877062707397,R45chimp:0.00316612167757060340):0.057146
73842690786570):0.01083317254389340283,(((((R44gorilla:0.03892984035029058848,(R31chimp:0.0058830
4501949800211,44bonobo:0.00369854246062357615):0.00966814411615519315):0.00348213934579672760,hT2
R44:0.01928152124852888089):0.01832359722569393279,(R44BOrang:0.02579177016695153798,R44Agibbon:0
.01163922329678868280):0.01441814668005636339):0.02652191464085023459,(R43bonobo:0.00954783252709
157787,(hT2R43:0.01266020455352769719,R43chimp:0.00315328420605896515):0.00000100000050002909):0.
04153254662455459212):0.01544966574501033599,((R43Hbaboon:0.01306390879135818986,(R43Rmacaq:0.009
49651572763727733,R46Rmacaq:0.00000100000050002909):0.02237197117219099848):0.0727889837467220468
5,(R45Slutung:0.07277367145360250089,(R43Slutung:0.02740562936926215637,(R45Rmacaq:0.032460156352
53440834,r43Ngibbon:0.03140081804645401820):0.04096387342754741895):0.00957851300281076408):0.038
84730355337475544):0.01478737564608049483):0.00979811321211819866):0.01601529863847804200):0.0076
0690642501907644,(((R46CEmacaq:0.06022413932744959120,(R47Rmacaq:0.09613148148488058486,R46Hbaboo
n:0.10255439728695844859):0.01558135584752437346):0.01955144963165395336,(((hT2R47:0.022897939403
14528362,(R30chimp:0.00000100000050002909,R47bonobo:0.00318940843660369582):0.0126714026659762411
8):0.00625944858800026038,R47gorilla:0.00309297656908613617):0.03134844724133407923,(R47BOrang:0.
02386010123682225439,(R47Ngibbon:0.00952925896932648245,R47Agibbon:0.01672464428284517468):0.0292
1884355432780023):0.01056679597407300490):0.05987735265449262850):0.02274844412803249047,(hT2R46:
0.00884509519046866068,((R46chimp:0.02596177358720792513,(R46bonobo:0.00000100000050002909,R66bon
obo:0.05976096441736460096):0.00000100000050002909):0.00317849327587422766,R46gorilla:0.009529504
72502990184):0.00712090302113299253):0.04897161070465031601):0.01875742429650942636):0.1033011629
9526889700,((R50Slutung:0.04681589526027954418,((r50CEmacaq:0.00345229372433426135,R50Rmacaq:0.00
000100000050002909):0.00000100000050002909,r50Rmacaq:0.00000100000050002909):0.0191055480187992 53
80):0.03637869437891622787,((R50BOrang:0.04239379585215439367,((r50chimp:0.01030159694720242207,h
T2R50:0.01374418336765580520):0.00685975320288069543,R50gorilla:0.00000100000050002909):0.0028871
7599779372098):0.00686700433105745869,(r50Ngibbon:0.00387338983607755103,R50Agibbon:0.01160120905
383481629):0.00776612831810548701):0.04076580458901490206):0.17664744654684072511):0.030531904937
48162218,(r3Rhowler:0.09314205854658688666,R15BOrang:0.10927433869418805290):0.08792224836426043 5
77):0.02216363488901099826):0.07226808168730899928,R49treeshrew:0.29949263711502760454):0.0349310
1479138027710):0.05062563500880342543):0.28509310309138707895,(((R13marmoset:0.10856544236282603 1
22,((R13Agibbon:0.01804163510973983212,(((R13gorilla:0.01179812616961429736,hT2R13:0.130010549655
46515740):0.00101515754076143762,(R13bonobo:0.00000100000050002909,R13chimp:0.0032590487610454224
9):0.02376267953170717318):0.02303973426297613661,R13BOrang:0.02778486122917918702):0.00374209318
781731177):0.02617866415932340596,(R13Slutung:0.01114376568122422799,(R13Hbaboon:0.00675352732599
747863,(R13CEmacaq:0.00624648752096279743,R13Rmacaq:0.00957384702761047285):0.0076293656002627907
9):0.02180388104668776195):0.03545898753103086942):0.05006677601164991054):0.13994326230952955359
,((R124mus:0.22974319004894200313,(R102mus:0.11576347259381875032,(R102rat:0.06013497589624571676
,r13rat:0.05034859239426670019):0.10933716018855704766):0.12780289329827237976):0.299829218733281
16646,((R121mus:0.08149755819146731195,R13rat:0.12829904553716769966):0.22359653858404035520,(R5G
molerat:0.05878032221707451405,R4Gmolerat:0.07990960593557640268):0.16141208561706832381):0.08740
192806156452110):0.05605934061173285798):0.27962109948391605796,(((R115mus:0.48001148047147507159
,R6Gmolerat:0.45310488262505210777):0.12111698892017434825,((((((R117rat:0.10889241912915573196,R
117mus:0.21882467546485004362):0.05106467245159839602,(R109mus:0.19618699470279063468,R109rat:0.1
3172322880019018965):0.07353977255240548572):0.29729147203166927937,R129rat:0.541682458203856076 4
7):0.09757197233585361562,R12Gmolerat:0.38159268700853871126):0.06019435908130756768,(((R110mus:0
.10693158900921193033,(R110rat:0.14848033567159704682,(R123rat:0.10792651276232403945,R123mus:0.1
2180544109282734344):0.05999852199884080084):0.01030263350433838904):0.52139465756611147462,(R3Gm
olerat:0.38785511973411934683,(R140rat:0.09737965524588440558,R140mus:0.14714759934875532021):0.2
3404291710990438036):0.06780718300087380801):0.01707614589010529246,(R2Gmolerat:0.499505123108074
60015,((r125rat:0.47568845606608944898,R11Gmolerat:0.32293942786043472459):0.04988594677544020556
,(R16rat:0.14075926367570454345,R125mus:0.13564764678675628184):0.20637924320691142244):0.0833758
5315919478213):0.05250877067926495306):0.01581216195484392434):0.03153209133361614680,((R116rat:0
.21269326787065098716,R116mus:0.11951986012251374081):0.30976611093111178219,(R113mus:0.134411265
14327961239,R113rat:0.10640544849508358494):0.43325427053016818402):0.06645840388858706427):0.028
37309824430336858):0.08139472543878166488,((((R14Rmacaq:0.00844464055657732394,R14CEmacaq:0.00375
265248795134728):0.01347390780143334701,R14Hbaboon:0.02116595890185219916):0.03628280196024992466
,R14Slutung:0.05507041119956837538):0.04159552673168864789,((R14gorilla:0.00610160749687738667,((

0100000050002909):0.000001000000050002909):0.000001000000050002909):0.01318650746709739557,R10psDho
le:0.47816506448502132942):0.02353301504178198847,R10panda:0.08196241948267196653):0.079826669800
68513218):0.06103179573052623652,(R10CMarmos:0.17585295968104758257,((R10Rmacaq:0.030448715347479
99042,R10HBaboon:0.03540104249237677192):0.01662026129612075021,((R10NGibbon:0.028036220111569514
63,((hT2R10:0.01994700120247292277,R10gorilla:0.000001000000050002909):0.00652545233793331344,R10c
himp:0.00956880400312283205):0.01427820776905147811):0.01178931724095358749,R10BOrang:0.031265635
10002099838):0.01715438283679925763):0.07032359454561519507):0.04529992672651261126):0.0247455130
9565834606):0.28780542372714551513):1.14875526818439177568,(V1R_alligator:0.96739291947146865969,
((V1r69_mus:1.04962891577166694823,(V1r96_rat:1.09260922590970110946,v1r_RMacaq:1.156731196012271
70072):0.08422319054083844680):0.51761310201829802136,V1r_Zebrafish:0.64573056999141209555):0.189
64822856537660867):1.14875526818439177568);

23 T2R10-like seqs, 138 T2R46-like seqs (45%+ identity cutoff), 14 hTAS2Rs, 10OG

((((((r45Dyak:0.00309802749119156501,r46cattle:0.01432247283144792716):0.26484169859135775482,((R
46Dyak:0.00702974254400849478,r31cattle:0.000001000000050002909):0.15212120597149830781,r31Dyak:0.
16454061728396587871):0.08979340560606631494):0.09432473670232631446,(((r46Bbat:0.042497830146610
40470,r66Dbat:0.06630394514912862913):0.11165970761282741075,R45Bbat:0.16848850433223971912):0.14
961111248271474761,((CanisT2R44p_restored:0.08818276912285885616,R136Canis:0.04001051221526222973
):0.11117202761674901956,(r31panda:0.09085255128698944971,PANDA_014286:0.09664108475877117299):0.
08641799958753738908):0.09251186468376905447):0.03430307806874363652):0.04894248698450501950,((((
(SlutungT2R15p_restored:0.07309730798866244383,((BOrangPs8_restored:0.000001000000050002909,R15BOr
ang:0.000001000000050002909):0.05751152116185466456,hT2R15p:0.01978986739289200630):0.017323615432
12207824):0.03580943028191629907,r3Rhowler:0.09824124444184663407):0.08639186362992619383,((((R46
CEmacaq:0.05822741002121555043,(R46Hbaboon:0.10835815471783073749,R47Rmacaq:0.0985648517636898702
2):0.01746208839184281922):0.01799594297820617667,((R47gorilla:0.00347503960433586225,((R30chimp:
0.000001000000050002909,R47bonobo:0.00331070613068631643):0.01670673897915263620,hT2R47:0.01253133
508827605422):0.00314747570765569603):0.03327499961243449850,(R47BOrang:0.02488724787609022590,(R
47Ngibbon:0.00985998267181236766,R47Agibbon:0.01706780018902075494):0.02994692965749768884):0.010
20375155574767753):0.06846749408310728868):0.02616794463328858678,(r2Rhowler:0.133030897460578501
07,((R43Hbaboon:0.01072256505006280967,(R46Rmacaq:0.000001000000050002909,R43Rmacaq:0.009877371443
85005157):0.02610334721035310543):0.08054371212370296795,((R44Hbaboon:0.00823914019203283651,R8Rm
acaq:0.02170189754714492783):0.07873208158559129310,(((R45chimp:0.00374696744782587136,(((R45gori
lla:0.000001000000050002909,R45BOrang:0.03129490019225008440):0.01105605275306579573,hT2R45:0.0244
9496420371499381):0.00339381416302406190,R45bonobo:0.00863710380035976298):0.00422224094015903433
):0.02871329794059725507,(((R46bonobo:0.000001000000050002909,R46chimp:0.02699670490200350645):0.0
00001000000050002909,(R46gorilla:0.00993459193290183173,hT2R46:0.01666689823698865383):0.003304585
42873068384):0.04755148467440301063,R66bonobo:0.01433537651194164743):0.05549448596090009178):0.0
3567414850938169929,(((r43Ngibbon:0.03315081039165782006,R45Rmacaq:0.03178604908979174026):0.0367
8115431829695470,(R43Slutung:0.02764225291547743948,R45Slutung:0.07773475677670900474):0.01204716
716413193126):0.04645500671584869290,(((hT2R43:0.01317473125061765712,R43chimp:0.0032836564141309
9233):0.000001000000050002909,R43bonobo:0.00993896723150760296):0.04222782895532119912,((R44BOrang
:0.02652709781421908747,R44Agibbon:0.01182492488614988319):0.01484689671270401218,(hT2R44:0.02005
578594878906773,(R44gorilla:0.04038364681208822526,(44bonobo:0.00383630183414208903,R31chimp:0.00
613153111623808249):0.01003544408249853678):0.00365019676105952885):0.01944872142426838299):0.030
54969647927144663):0.00969498539445387206):0.01052138717139600339):0.01887961636553918757):0.0099
9209130236787708):0.01215749378039928177):0.00795206925194522941):0.10750753113813001083,(((r50Ng
ibbon:0.00395991909364857425,R50Agibbon:0.01180630920063904798):0.00815728049698201743,((R50goril
la:0.000001000000050002909,(r50chimp:0.01052033142803762526,hT2R50:0.01404233553358018131):0.00701
620048972778111):0.00304346582697066596,R50BOrang:0.04341070926395182439):0.00670835812189217711)
:0.04100427643935557198,(R50Slutung:0.04785557943623582750,(r50Rmacaq:0.000001000000050002909,(R50
Rmacaq:0.000001000000050002909,r50CEmacaq:0.00352767969616896073):0.000001000000050002909):0.019569
67970500784332):0.03768299309305114808):0.18090665335287678395):0.02455297620111587809):0.0215132
0130803177286,(((hT2R63p_segment:0.22183666584462946592,BOrangT2R68p_restored:0.10458510883641257
416):0.03787530768240376933,((((R64Rmacaq:0.00786775829790504165,r48Rmacaq:0.000001000000050002909
):0.000001000000050002909,r48CEmacaq:0.00350326651337964012):0.02376097416635562093,HbaboonT2R48p_
restored:0.01934387050455986176):0.10163448066711475160,((r64chimp:0.00714073605461289576,((hT2R6
4p:0.00337072178802392272,(BOrangT2R64p_restored:0.01101792356348883957,BOrangPs2_restored:0.0000
0100000050002909):0.06511271247945794871):0.01284066949730831679,R64bonobo:0.00702502557993112727
):0.03854897369292088066):0.03972308387674674396,(r48chimp:0.000001000000050002909,((R48gorilla:0.
00345456070279421016,(hT2R48:0.01383495336954478593,R48BOrang:0.06874313910058375832):0.006720897
37004168391):0.01392291433751242577,R48bonobo:0.00684037462763549108):0.01042496279022966064):0.0
4598306705223231122):0.03716560728646844947):0.02323139357794154827):0.13279491521909247975,((R49
BOrang:0.03521091373219024934,((r49Ngibbon:0.000001000000050002909,R49Agibbon:0.004369231684808200
93):0.03486019920937169503,((r49chimp:0.000001000000050002909,(hT2R49:0.00982615905681440138,r20ch
imp:0.00325122454312267188):0.000001000000050002909):0.00652507157792383413,(R49Hbaboon:0.00000100
000050002909,R49gorilla:0.00650755036943630183):0.00324754993220439069):0.01692565234899937737):0

.01752762564485644062):0.04617865781852557860,(r49Rmacaq:0.00330359544117201894,r49CEmacaq:0.0000
0100000050002909):0.08231409005344592078):0.13053222020021931771):0.04099018022628573427):0.06937
451268709571295,R49treeshrew:0.29679416300405025986):0.07637920513588800087):0.426693440195681561
46,(((R107Mus:0.07718594770165933261,R107Rat:0.09795971281606086212):0.31254439367983127829,(T7Gm
olerat:0.37184701451770368896,(R106Rat:0.11288233427784145901,R106Mus:0.06504043769729979385):0.2
4031418347054273044):0.04494727612121554583):0.12382658013472211400,(((((WolfR10:0.00312376838383
768805,(ManedWolfR10:0.00000100000050002909,(HuntingDogR10:0.00000100000050002909,R10Canis:0.0000
0100000050002909):0.00000100000050002909):0.00000100000050002909):0.00311414843589902322,((Fennec
FoxR10:0.01569085399216592347,(RedFoxR10:0.00935966396196897914,CorsacFoxR10:0.003118533171118492
80):0.00000100000050002909):0.00000100000050002909,TibetanFoxR10:0.00000100000050002909):0.000001
00000050002909):0.02571732465969073581,R10panda:0.09038556746851235391):0.08750598520345638509,(R
10cattle:0.09122566711707552622,R10sheep:0.18024574326643452715):0.19574943819966522662):0.063409
07941723550323,(R10CMarmos:0.18047451114030843566,(((((hT2R10:0.01729576692445664032,R10gorilla:0
.00000100000050002909):0.00664045609502903877,R10chimp:0.00995566242519976309):0.0145074782494594
2954,R10NGibbon:0.02924317951435947624):0.01215132063907453999,R10BOrang:0.03223411214859153023):
0.01751828148817991676,(R10Rmacaq:0.03196211699698794928,R10HBaboon:0.03674607041928125428):0.017
22528900827205098):0.07471500507358262710):0.04664037859065452152):0.05387179654206046553):0.6242
5257596775129976):0.33273544227148210695,(T2R20_frog:1.42616438329631334270,(T2R_alligator:1.3739
5249232061411604,(((v1r_RMacaq:1.10797729253442489572,V1r96_rat:1.02981050739236690283):0.1285368
0874323736294,(V1r62_mus:1.21582073714602989511,(V1r69_mus:0.65232267367769791733,v1r3_chimp:0.41
007574729278239989):0.31487759063579146002):0.22042956394175131085):0.43598536108525920429,(V1r_Z
ebrafish:0.53318297608418629974,(V1R_alligator:1.11178342637220795019,v1r_frog:0.5596731757062409
7733):0.20426605280806639509):0.17119927152449390673):0.51735866405791353095):1.09000623325971468
525):0.33273544227148210695);

40% identity cutoff; OG = mouse Vmn69

(((T2R117_Rattus41%:0.14994061200042693716,T2R117_Mus41%:0.19224970988134801320):0.27019589760918
383270,((((((Bota-T2R10C_Bos62%:0.25608880227588920464,(T2R10_Bos60%:0.10241328143378504445,T2R10
B_Bos59%:0.14080713779429598898):0.12350759986060445839):0.02945574126914101212,((((T2R106_Rattus
52%:0.10361565139182635220,T2R106_Mus54%:0.06357302411686088783):0.25744745243447486605,((T2R107_
Rattus54%:0.09582730799096006447,T2R107_Mus52%:0.07175934741601763811):0.26089521861842157602,((T
2R114_Rattus54%:0.09547432538739947239,T2R114_Mus55%:0.07508321797081143889):0.217503413742528295
40,((T2R105_Rattus54%:0.09785086571265751154,T2R105_Mus53%:0.08135211040826600959):0.192004647096
81683284,(T2R104_Mus53%:0.07755946868683090412,T2R104_Rattus52%:0.07052511258570950958):0.2021347
5245495141075):0.06568930491289885765):0.08143565018621951035):0.05501335210617410570):0.15223357
433077869261,(T2R10_PanTrogl98%:0.01098401391507244917,hT2R10:0.00780656818845017024):0.143596908
16529585566):0.08296807666046400465,T2R10_Canis75%:0.09568112907787292010):0.04529484251041622500
):0.90381598732875922941,((T2R13_Macaca46%:0.05561043928503466710,T2R13_PanTrogl48%:0.09474530487
756441244):0.18031909231798293192,(T2R13_Mus46%:0.08878035190599685433,T2R13_Rattus46%:0.11332538
462759761400):0.30617978053636984992):0.23437319858450889054):0.06091435254111038367,((T2R136_Can
is61%:0.23864662459051197430,T2R46_Bos56%:0.33232579557907004908):0.05596926481779357365,(((T2R13
6_Rattus46%:0.11525734289417390033,T2R136_Mus43%:0.17756901258923835085):0.39030283777110108545,(
T2R120_Rattus48%:0.15649783807922057477,T2R120_Mus50%:0.03678273888752745663):0.41861060397832305
924):0.13241054192523843414,(T2R50_PanTrogl70%:0.21634598993820527979,(T2R20_PanTrogl68%:0.223109
87061640014106,(hT2R46:0.04976693375885155035,T2R46_Macaca84%:0.11509650367716675601):0.131918815
31196625321):0.03322655514835543694):0.10735099272547533877):0.04149092067306756060):0.3142974331
3388602832):0.14535684359021106005,T2R110_Rattus41%:0.55484764947616704855):0.1370942064144393401
9,((T2R125_Mus41%:0.12909883929079180032,T2R125_Rattus41%:0.14535791293957667225):0.2132621234948
3331110,(T2R140_Rattus44%:0.08087560120520777274,T2R140_Mus42%:0.15874960258216089248):0.32146725
213387838949):0.07081504804087426774):0.11203137437360266571):1.63402627541203981920,Vmn1r69_MOUS
E:1.63402627541203981920);

Phylogenetic tree including T2R43s in Shang et al.'s study:

(((pigT2R10:0.104326,((((((R10HBaboon:0.036757,R10Rmacaq:0.032001)N9:0.017400,(R10BOrang:0.032294
,((R10chimp:0.009974,(hT2R10:0.016678,R10gorilla:0.000000)N13:0.006651)N12:0.014549,R10NGibbon:0.
029292)N11:0.012271)N10:0.017329)N8:0.075503,R10CMarmos:0.181894)N7:0.056679,((R107Mus:0.078240,R
107Rat:0.098129)N15:0.314026,(T7Gmolerat:0.373978,(R106Mus:0.063174,R106Rat:0.114496)N17:0.237774
)N16:0.037692)N14:0.167277)N6:0.070137,((R10panda:0.078574,((((HuntingDogR10:0.000000,R10Canis:0.
000000)N23:0.000000,ManedWolfR10:0.000000)N22:0.000000,WolfR10:0.003155)N21:0.003143,(TibetanFoxR
10:0.000000,(FennecFoxR10:0.015832,(CorsacFoxR10:0.003147,RedFoxR10:0.009447)N26:0.000000)N25:0.0
00000)N24:0.000000)N20:0.038290)N19:0.034582,(PantheraPardusR10:0.023395,AcinonyxJubatusR10:0.009
191)N27:0.102279)N18:0.030801)N5:0.070615,(R10cattle:0.104341,R10sheep:0.161162)N28:0.159671)N4:0
.054220)N3:0.593278,((R45Bbat:0.176917,(r46Bbat:0.043555,r66Dbat:0.065305)N31:0.105249)N30:0.1171
07,(((r45Dyak:0.003109,r46cattle:0.014293)N34:0.261910,(r31Dyak:0.166042,(r31cattle:0.000000,R46D
yak:0.007034)N36:0.149465)N35:0.091680)N33:0.087845,((((((r50CEmacaq:0.003530,R50Rmacaq:0.000000)
N42:0.056377,(r50Ngibbon:0.012114,(R50BOrang:0.043401,((hT2R50:0.014055,r50chimp:0.010532)N46:0.0
07023,R50gorilla:0.000000)N45:0.003087)N44:0.006897)N43:0.040431)N41:0.183433,(((hT2R46:0.009184,

(R46gorilla:0.009912,(R46chimp:0.026978,(R66bonobo:0.062291,R46bonobo:0.000000)N52:0.000000)N51:0
.003308)N50:0.007420)N49:0.049782,(((R47gorilla:0.003472,((R47chimp:0.000000,R47bonobo:0.003302)N
57:0.016715,hT2R47:0.012516)N56:0.003138)N55:0.032438,((R47Agibbon:0.017041,R47Ngibbon:0.009846)N
59:0.029962,R47BOrang:0.024886)N58:0.010975)N54:0.061451,(R46CEmacaq:0.063012,(R46Hbaboon:0.10662
0,R47Rmacaq:0.098134)N61:0.016206)N60:0.020299)N53:0.024224)N48:0.019599,((((((hT2R43:0.013160,R4
3chimp:0.003280)N67:0.000000,R43bonobo:0.009916)N66:0.043164,((R44BOrang:0.026707,R44Agibbon:0.01
1809)N69:0.014964,(hT2R44:0.020012,((44bonobo:0.003852,R44chimp:0.006103)N72:0.010037,R44gorilla:
0.040324)N71:0.003652)N70:0.019029)N68:0.027565)N65:0.015289,((R43Hbaboon:0.013600,(R46Rmacaq:0.0
00000,R43Rmacaq:0.009870)N75:0.023180)N74:0.075198,(R45Slutung:0.074637,((R45Rmacaq:0.033439,r43N
gibbon:0.032187)N78:0.042228,R43Slutung:0.028101)N77:0.009749)N76:0.040472)N73:0.015524)N64:0.010
256,((R8Rmacaq:0.023806,R44Hbaboon:0.005763)N80:0.077287,((R45bonobo:0.008843,((R45gorilla:0.0000
00,R45BOrang:0.031213)N84:0.011039,hT2R45:0.024494)N83:0.003411)N82:0.004469,R45chimp:0.003281)N8
1:0.059100)N79:0.010656)N63:0.016538,r2Rhowler:0.136573)N62:0.007605)N47:0.103687)N40:0.032317,(r
3Rhowler:0.096464,R15BOrang:0.109458)N85:0.084988)N39:0.019809,(((R49BOrang:0.033549,(((hT2R49:0.
009805,r49chimp:0.000000)N91:0.006508,(R49Hbaboon:0.000000,R49gorilla:0.006490)N92:0.003239)N90:0
.016842,(r49Ngibbon:0.000000,R49Agibbon:0.004807)N93:0.038552)N89:0.018727)N88:0.047179,(r49Rmaca
q:0.003300,r49CEmacaq:0.000000)N94:0.082367)N87:0.132625,((r48chimp:0.001781,((R48gorilla:0.00345
0,(hT2R48:0.013820,R48BOrang:0.068708)N99:0.006735)N98:0.013910,R48bonobo:0.006841)N97:0.008617)N
96:0.045417,((R64Rmacaq:0.007843,(r48CEmacaq:0.003500,r48Rmacaq:0.000000)N102:0.000000)N101:0.141
304,(R64bonobo:0.038148,r64chimp:0.013634)N103:0.033012)N100:0.018650)N95:0.160001)N86:0.032959)N
38:0.201292,((((FennecFoxTas2r43:0.023688,CorsacfoxTas2r43:0.032693)N107:0.014698,(R136Canis:0.00
2824,WolfTas2r43:0.008900)N108:0.019707)N106:0.138353,(PANDA_014286:0.093508,r31panda:0.096076)N1
09:0.088530)N105:0.061732,(Acinonyx_jubatusT2r43:0.135068,Panthera_pardusT2r43:0.064616)N110:0.09
8654)N104:0.047858)N37:0.013658)N32:0.046450)N29:0.424191)N2:0.332837,(T2R20_frog:1.441513,(T2R_a
lligator:1.409834,(((V1r62_mus:1.233597,(V1r69_mus:0.662300,v1r3_chimp:0.415760)N116:0.322669)N11
5:0.224098,(v1r_RMacaq:1.126916,V1r96_rat:1.059021)N117:0.110331)N114:0.409559,((V1R_alligator:1.
147393,v1r_frog:0.562725)N119:0.205940,V1r_Zebrafish:0.532435)N118:0.216262)N113:0.518780)N112:1.
089530)N111:0.332835)N1;

Resequencing Alignment of Neanderthal T2R10 with hT2R10
REF:36 1
AATGGGAGAAAATTTTTGCAATCTACTCATCTGACAAAGGGTTAATATCTAGAATCTACAATGAACTCCAATAAATTTATAAGAAAAAAAACCCCAC
CAAAAAGTGGGCAAAGGATATGA 120
1KG_NA12878_pilot2 1
....
.... 120
1KG_NA12891_pilot2 1
....
.... 120
1KG_NA12892_pilot2 1
....
.... 120
1KG_NA19240_pilot2 1
....
.... 120

REF:36 121
ACAGACACTTCTCAAAAGAAGACATTTATGCAGCTAAAAGACACATGAAAACATGCTCATCATCACTGGCCATCAGAGAAGTGCAAACCAAAACCAC
AATGAGATACCATCTCACACCAG 240
1KG_NA12878_pilot2 121
....
.... 240
1KG_NA12891_pilot2 121
....
.... 240
1KG_NA12892_pilot2 121
....
.... 240
1KG_NA19240_pilot2 121
....
.... 240

REF:36 241
TTAGAATGGTGATCATTAAAAAGTCAGGAAACAACAGGTGCTGGAGAGGATGTGGAGAAATAGGAACACTCTTACACTGTTGGTGGGACTGTAAACT
AGTTCAACCATTGTGGAAATCAA 360
1KG_NA12878_pilot2 241

```
.................................................................................................
.......................    360
1KG_NA12891_pilot2     241
.................................................................................................
.......................    360
1KG_NA12892_pilot2     241
.................................................................................................
.......................    360
1KG_NA19240_pilot2     241
.................................................................................................
.......................    360

REF:36                 361
TGTGGTGATTCCTCAGGGGTCTAGAACTAGAAATACCATTTGACCTAGTGATCCCATTACTGGGTATATACCCAAAGGATTATAAGTCATGCTGCTA
TAAAGACACATGCACATGTATGT    480
1KG_NA12878_pilot2     361
.................................................................................................
.......................    480
1KG_NA12891_pilot2     361
.................................................................................................
.......................    480
1KG_NA12892_pilot2     361
.................................................................................................
.......................    480
1KG_NA19240_pilot2     361
.................................................................................................
.......................    480

REF:36                 481
TTATTGCGGCACTATTCACAATAGCAAAGACTTGGAACTAACCCAAATGTCCAACAATGATAGACTGGATTAAGAAAATGTGGCACATATACACCAT
GGAATACTATGCAGCCATAAAAA    600
1KG_NA12878_pilot2     481
.................................................................................................
.......................    600
1KG_NA12891_pilot2     481
.................................................................................................
.......................    600
1KG_NA12892_pilot2     481
.................................................................................................
.......................    600
1KG_NA19240_pilot2     481
.................................................................................................
.......................    600

REF:36                 601
ATGATGAGTTCATGTCCTTTGTAGGGACATTGATGAAGCTGGAAACCATCATTCTCAGCAAACTATCGCAAGGACAAAAAACCAAACACCACATGTT
CTCACTCATAGGTGGGAATTGAA    720
1KG_NA12878_pilot2     601
.................................................................................................
.......................    720
1KG_NA12891_pilot2     601
.................................................................................................
.......................    720
1KG_NA12892_pilot2     601
.................................................................................................
.......................    720
1KG_NA19240_pilot2     601
.................................................................................................
.......................    720

REF:36                 721
CAATGAGAACACATGGACACAGGAAGGGGAACATCACATACCGGGGCCTGTTGTGGGGTGGGGGGAGTGGGGAGGGATAGCATTTGGAGATTTACCT
AATGTTAAATGACGAGTTACTGG    840
1KG_NA12878_pilot2     721
.................................................................................................
.......................    840
```

```
1KG_NA12891_pilot2     721
.................................................................................................
.......................    840
1KG_NA12892_pilot2     721
.................................................................................................
.......................    840
1KG_NA19240_pilot2     721
.................................................................................................
.......................    840

REF:36                 841
GTGCAGCACACCAACATGGCACATGTATACATATGTAACGTTGTGCACATGTACCCTAAAACTTAAAGTATAATAATAAAAAAGTCAAAACAATCTT
TCCCTTTATTCTCTTTCCTGATG    960
1KG_NA12878_pilot2     841
.................................................................................................
.......................    960
1KG_NA12891_pilot2     841
.................................................................................................
.......................    960
1KG_NA12892_pilot2     841
.................................................................................................
.......................    960
1KG_NA19240_pilot2     841
.................................................................................................
.......................    960

REF:36                 961
TATACATTTTTAAAAAGGAAAAGTTTAGACTAAATGGAAGAAATTACAATGTAGAATTGGAAAATATTTAAACTTTGCACTA
                          1042
1KG_NA12878_pilot2     961
..................................................................................
                          1042
1KG_NA12891_pilot2     961
..................................................................................
                          1042
1KG_NA12892_pilot2     961
..................................................................................
                          1042
1KG_NA19240_pilot2     961
..................................................................................
                          1042

Resequencing Alignment of Neanderthal T2R46 with hT2R46
REF:36                   1
ATTTGAGATAGAGTCTCGCTCTGTTGCCCAGGCCGGAATGCAGTGTTGCAATCCCGGCTCACTGCAAACTTCGCCTCCTGGGTTCAATCAATTCAAA
TCCTTCAGCCTCCTGAGTAGCTG    120
1KG_NA12878_pilot2       1
.................................................................................................
.......................    120
1KG_NA12891_pilot2       1
......................................................Y..........................................
.......................    120
1KG_NA12892_pilot2       1
.................................................................................................
.......................    120
1KG_NA19240_pilot2       1
.................................................................................................
.......................    120

REF:36                 121
GGTTTACGGGCATTGGCCACCATGCCTGGCTAAATTTTTTTGTATTTTTTTTTAAGTAGAGATGGGGTTTCACCATGTTGCTCAGGCTAGTCTCAAA
CTCCTGGCCTCCAGTGATCTGCC    240
1KG_NA12878_pilot2     121
.................................................................................................
.......................    240
1KG_NA12891_pilot2     121
```

```
.................................................................................................
.......................    240
1KG_NA12892_pilot2     121
.................................M...............................................................
.......................    240
1KG_NA19240_pilot2     121
.................................................................................................
.......................    240

REF:36                 241
TGCGTTGGTCTCCCAAAGTGCTGGTATTACAGGCGTGAACCACCATGCCGAGCAGAAACATCCTGTTTAAGGTAAATGGCTTCCTATATACTTCCTA
TCACTAAAAGAGAAGCACGGCGT    360
1KG_NA12878_pilot2     241
.................................................................................................
.......................    360
1KG_NA12891_pilot2     241
.................................................................................................
.......................    360
1KG_NA12892_pilot2     241
.................................................................................................
.......................    360
1KG_NA19240_pilot2     241
.................................................................................................
.......................    360

REF:36                 361
TTATTGAGCCTCTAGGGATTTTGGAGTCCAAAAATACCACAGTAGAGGATATTGCTCTGATGTGTTAATAAAGTCCTTTTTAAATCCCTGTTCAGTT
AACCTACCTAGCCTCTGCCAAAA    480
1KG_NA12878_pilot2     361
.................................................................................................
.......................    480
1KG_NA12891_pilot2     361
.................................................................................................
.......................    480
1KG_NA12892_pilot2     361
.................................................................................................
.......................    480
1KG_NA19240_pilot2     361
.................................................................................................
.......................    480

REF:36                 481
CCAGATGGGTTACAGAATGAATGCAAAGACTCCTGGTTTCAAGTGGAATCCAGAACATATAATATCTCTACAGCAGATACAGGGTCTGGCCCAACCT
GCTCGGCCCATTGTGCCAGATGA    600
1KG_NA12878_pilot2     481
.................................................................................................
.......................    600
1KG_NA12891_pilot2     481
...........R.....................................................................................
.......................    600
1KG_NA12892_pilot2     481
.................................................................................................
.......................    600
1KG_NA19240_pilot2     481
.................................................................................................
.......................    600

REF:36                 601
TCCAGCAGAATTCAAAGCTGCTAGAGGCATGCAACGTGGGCATCATAGGACTCTGTGCCTGTCTCCATCAAGCCCGTAGGAGAGGAGAAAGCAAACC
CCTAGGGAATTACTGCAGAACTA    720
1KG_NA12878_pilot2     601
.............................................Y...................................................
.......................    720
1KG_NA12891_pilot2     601
.............................................Y...................................................
.......................    720
```

```
1KG_NA12892_pilot2      601
.............................................Y...................................................
.......................    720
1KG_NA19240_pilot2      601
.............................................Y...................................................
.......................    720

REF:36                  721
TTCCCTCTTCAGTGGAGGATTATCCGCTGTCTGAAAAATAAAAATGCAAGTGCAGAACATTAATCCAAGCAAAAAGCCCCTCAAGCACAAGACCCCG
TGCAACTACACAAGGCATCTGCT    840
1KG_NA12878_pilot2      721
...................K.............................................................................
.......................    840
1KG_NA12891_pilot2      721
...................G.............................................................................
.......................    840
1KG_NA12892_pilot2      721
...................K.............................................................................
.......................    840
1KG_NA19240_pilot2      721
...................K.............................................................................
.......................    840

REF:36                  841
TATAAAACCAGCCACAGTTGGAGGGCATGAGCACATCTGAGTGGCACAAGGAGGGGACTATATTGGACACAAACATGTGCTTCCTCAGAT
                           930
1KG_NA12878_pilot2      841
..........................................................................................
                           930
1KG_NA12891_pilot2      841
..........................................................................................
                           930
1KG_NA12892_pilot2      841
..........................................................................................
                           930
1KG_NA19240_pilot2      841
..........................................................................................
                           930
```
