# Supplementary material for: Independent Evolution of Strychnine Recognition by Bitter Taste Receptor Subtypes
Source: Front Mol Biosci. 2018 Mar 2;5:9. doi: 10.3389/fmolb.2018.00009 (PMC5840161; doi:10.3389/fmolb.2018.00009)
Supplement: Supplementary file 3 [file DataSheet3.docx]

Supplementary 3

**Independent Evolution of Strychnine Recognition by Bitter Taste Receptor Subtypes**

Ava Yuan Xue, Antonella Di Pizio, Anat Levit, Tali Yarnitzky, Osnat Penn, Tal Pupko and Masha Y. Niv


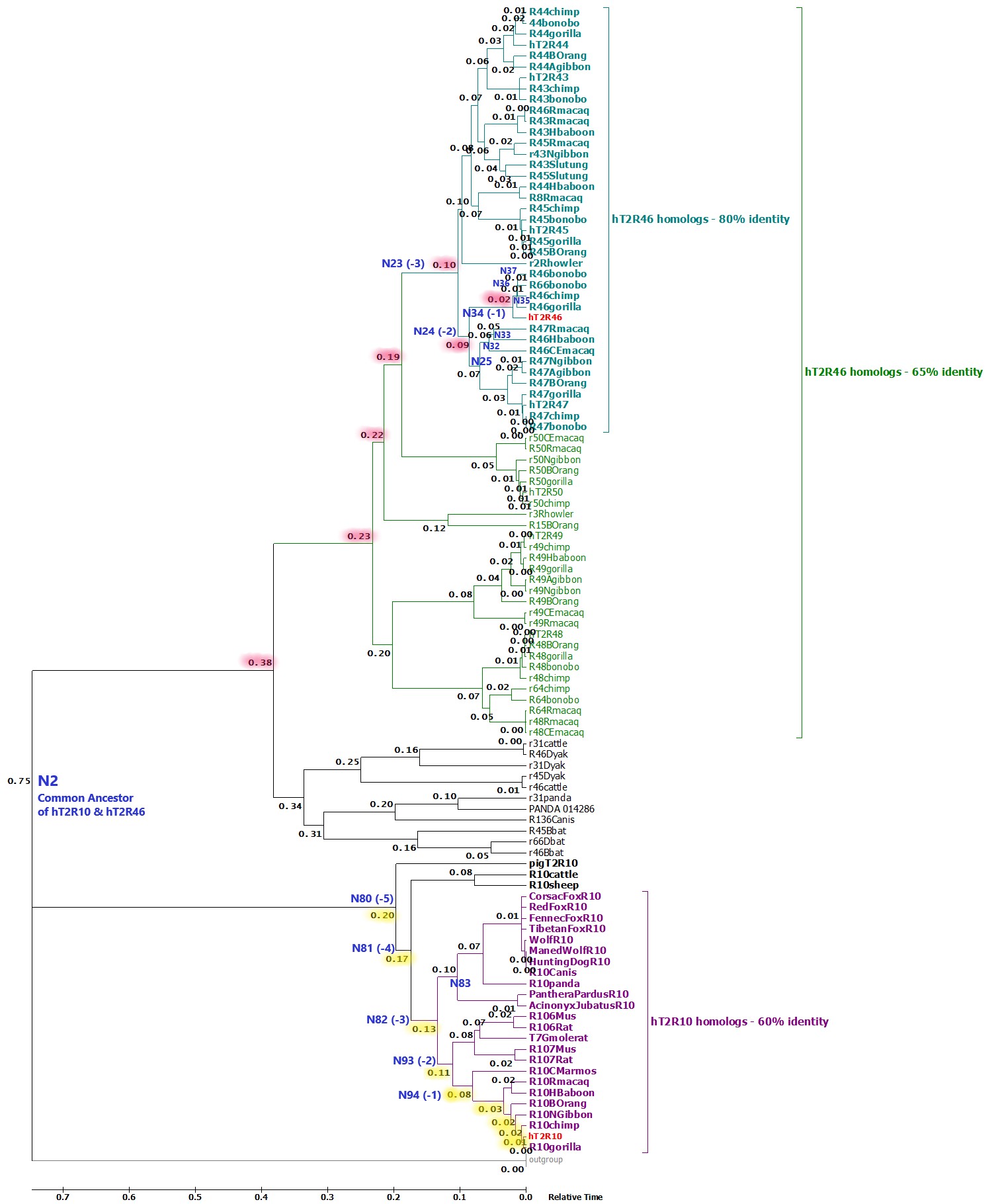


FIG. s1: Phylogeny with reconstructed common ancestors and estimated relative divergence times. Divergence times are displayed near each node and highlighted in yellow for hT2R10 lineage and pink for hT2R46 lineage; N94 (-1) – N80 (-5) refer to the five most recent common ancestor of hT2R10 clade, with N94 (-1) being the most recent and N80 (-5) the earliest (FIG. 3); same principle applies for N34 (-1), N24 (-2), and N23 (-3) in the hT2R46 clade. Generally, strychnine sensitivity is predicted to decrease going back alone the timeline (TAB. 1). N83 is the common ancestor of canidae and felidae; N35-N37 are the common ancestors of gorilla T2R46, chimp T2R46, and bonobo T2R46 and T2R66, respectively; they appear to have evolved after the divergence of hT2R46 (FIG. 4). N2 is the common ancestor of hT2R10 and hT2R46, and is very unlikely to recognize strychnine.

**
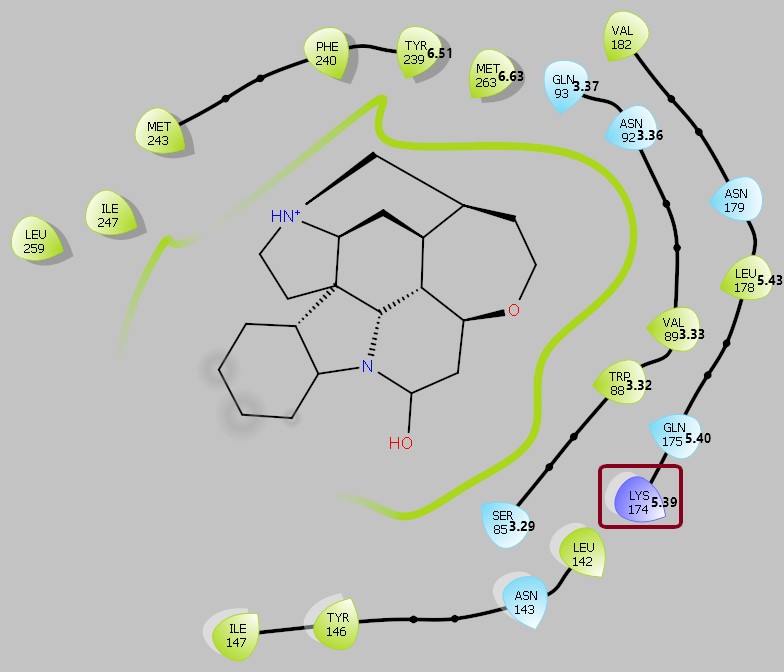
**

FIG. s2: 2D ligand interaction diagram of hTAS2R10 with 5.00 Å cutoff distance between ligand and the closest atom in the residue, using a homology model of T2R10 generated previously (Born et al. 2013); BW numbering is shown next to protein label and residue position; LYS174 mentioned in the text is highlighted in red rectangle. Figure was generated using Maestro 11.

### **Pseudogene restoration**

Nine pseudogenes were retrieved via BLASTn searches by using the nucleic acid sequence of hT2R46 as query sequence with a cutoff identity of 80% in the nucleotide collection database. Additional two (hT2R15p, hT2R64p) were obtained from the human T2R gene family on [HGNC website](http://www.genenames.org/cgi-bin/genefamilies/set/1162). In modifying the open reading frames, a parsimonious approach was used, i.e. minimum adjustments were made to maximally preserve the original sequence.

Homologs were identified by names for properly annotated pseudogenes. For those with incomplete annotation (e.g. “Ps8”), if the intact section before the first stop codon is longer than 250 amino acids, it was aligned with other hT2R46-like sequences on the MAFFT server ([Katoh et al. 2002](https://www.ncbi.nlm.nih.gov/pubmed/12136088)) to find its homologs. Otherwise, sections near but before the first premature stop codon were selected and compared to the functional hT2R46-like sequences. In this process, highly conserved residues such as Trp and conserved motifs in T2Rs, such as FYxxK in TM3 and HSxxL in TM7 (Di Pizio et al. 2016), were used as benchmarks to facilitate the identifying of homologs. These homologs were then used as templates to substitute the stop codons with the respective amino acids. To recover the two human T2R pseudogenes, for instance, the “TAG” in hT2R15p positions 469-471 was replaced with “CAG”, corresponding to the Gln in the functional BOrang T2R15; in hT2R64p, the “TGA” in positions 757-759 was replaced with “TGG” based on the Trp in the corresponding position in the functional T2R64s from chimp and bonobo (Risso et al. 2017). Rhesus macaque (Rmacaq) T2R44p was restored by using R44Hbaboon and R8Rmacaq as templates, based on which the first premature stop codon (“TAA”) was changed to Tyr (TAT or TAC), “T” was inserted between positions 408-409 to change Trp to Leu and also eliminate the rest of the premature codons, and the “G” in position 667 was removed to change Gly in the pseudogene to Val. The resulting sequence matched the homologs without any premature codons. The T2R15 of Bornean orangutan was found most closely resembled Bonobo Ps8 through the MSA. Therefore using R15BOrang as a template, “TT” was inserted between positions 552-553 in Bonobo Ps8 and the stop codon “TAG” at positions 769-771 was changed to “CAG.” “CC” was added between positions 816-817. BOrang Ps2 was found highly similar to the T2R64 from Rheus macaque, bonobo and chimp. Thus, the stop codon in position 39 of translated BOrang Ps2 was changed to Arg by changing “T” in the original DNA sequence position 115 to “C,” and by inserting “T” at positions 894-895 the rest of the sequence was recovered. For BOrang Ps8, the non-DNA residue “S” in position 59 was changed to “G” using Rhowler T2R2, T2R3; and using BOrang T2R15, “R” and “W” in positions 385 and 487 were both replaced with “A.”

Silver lutung T2R15p, HBaboon T2R48p were restored using their corresponding functional versions in other closely related primates, namely *Pongo pygmaeus* (BOrang) T2R15 and macaque T2R48 (r48Rmacaq and r48CEmacaq), respectively. “A” was inserted at Slutung T2R15p positions 537-538. In HbaboonT2R48p, “TGA” at 112-114 was replaced with “AGA,” and “A” was inserted at 892-893, and the remaining 33 nucleotides were discarded after translation. For BOrangT2R64p, “T” was inserted in the beginning of the DNA sequence, and the “T” at position 79 was switched to “C.” Likewise, BORangT2R68p also had “T” inserted in the beginning and the “G” in the original position 143 was deleted, while the “A” in position 254 was changed to “G” by comparing to its homolog, Bonobo T2R66. Lastly, “T” in the original position 451 was removed and “A” in position 720 was changed to “T” in CanisT2R44p, by comparing to its homolog CanisT2R136.

### **Discussion of pseudogenes**

In addition to the intact genes, 11 functionally restored pseudogenes were supplemented to the T2R46 clade for phylogenetic analysis. Pseudogenization has been hypothesized to serve as an engine of evolutionary change (Olson 1999) and is linked to adaptation to changes of environmental challenges ([Go et al. 2005](http://www.genetics.org/content/170/1/313); [Dong et al. 2009](https://bmcevolbiol.biomedcentral.com/articles/10.1186/1471-2148-9-12)). Among the 11 restored pseudogenes, T2R68p from Bornean orangutan (BOrang) share the most key functional residues with hT2R46 and is likely to be a strychnine binder, having three mutants in addition to the non-affecting E70V: L71F^2.66^, N150D^4.65^, and E253R^6.63^. Mutants L71F and N150K modestly reduce the activation by all agonists (Brockhoff et al. 2010). Since the physiochemical properties of Asn are much more similar to Asp than to Lys, N150D could be expected to have less impact on the protein function than does N150K.

In the MSA of selected human T2R pseudogenes together with hT2R10 and hT2R46, more hT2R pseudogenes displayed higher sequence identity with hT2R46 than with hT2R10, in the same way as more functional hT2Rs resemble hT2R46. Particularly, hT2R15p and hT2R64p were 74% and 69% identical to hT2R46, respectively. With respect to the key functional residues, hT2R15p contains 5 differing from hT2R46 while hT2R64p contains 6 (Suppl. 1, Tab “R46-likeKeyResidues”). A recent study reported that unlike hT2R46 the restored hT2R64p was narrowly tuned (Risso et al. 2017). But among the compounds tested (no strychnine included), hT2R64p and hT2R46 both bind amarogentin, andrographolide, colchicine and picrotoxinin. Thus the possibility for hT2R64p to bind strychnine cannot be ruled out. To reach a conclusion, all those pseudogenes need to be functionally restored and synthesized for *in vitro* test using strychnine. Nonetheless, those pseudogenes might have gone through similar selective pressure compared with the intact genes as shown in the similar identity distribution pattern. One possible explanation is that those genes became deactivated very recently after being functional before the hominoid speciation, conceivably as a result of functional redundancy (Risso et al. 2017). It could also be possible that the pseudogenes evolved at a similar speed as the functional T2R genes under low functional constraints (Shi et al. 2003). However, reasons behind the lack of both hT2R10-like pseudogenes and hT2R10-like functional genes remain to be explored and validated. More T2R pseudogenes from other primate species can be examined to test these hypotheses.

### **Outgroup identity with query sequences**

| **hT2R10** | 100% |  |  |  |  |  |  |  |  |  |  |  |  |  |  |  |  |
| --- | --- | --- | --- | --- | --- | --- | --- | --- | --- | --- | --- | --- | --- | --- | --- | --- | --- |
| **hT2R46** | 34% | 100% |  |  |  |  |  |  |  |  |  |  |  |  |  |  |  |
| **T2R11_frog** | 25% | 28% | 100% |  |  |  |  |  |  |  |  |  |  |  |  |  |  |
| **T2R20_frog** | 18% | 25% | 20% | 100% |  |  |  |  |  |  |  |  |  |  |  |  |  |
| **v1r3_chimp** | 14% | 12% | 15% | 12% | 100% |  |  |  |  |  |  |  |  |  |  |  |  |
| **v1r4_BOrang** | 14% | 13% | 14% | 13% | 55% | 100% |  |  |  |  |  |  |  |  |  |  |  |
| **V1r69_mus** | 15% | 16% | 13% | 16% | 40% | 46% | 100% |  |  |  |  |  |  |  |  |  |  |
| **v1r_frog** | 15% | 17% | 13% | 17% | 25% | 25% | 23% | 100% |  |  |  |  |  |  |  |  |  |
| **V1r_Zebrafish** | 17% | 14% | 13% | 15% | 25% | 28% | 23% | 38% | 100% |  |  |  |  |  |  |  |  |
| **V1R_alligator** | 15% | 18% | 13% | 10% | 22% | 17% | 19% | 29% | 28% | 100% |  |  |  |  |  |  |  |
| **V1r62_mus** | 13% | 13% | 12% | 16% | 25% | 30% | 25% | 21% | 20% | 18% | 100% |  |  |  |  |  |  |
| **V1r105_rat** | 12% | 14% | 12% | 13% | 26% | 27% | 25% | 21% | 24% | 21% | 20% | 100% |  |  |  |  |  |
| **V1r96_rat** | 13% | 15% | 12% | 13% | 23% | 24% | 24% | 19% | 22% | 16% | 23% | 50% | 100% |  |  |  |  |
| **v1r_RMacaq** | 12% | 13% | 8% | 10% | 22% | 21% | 24% | 21% | 21% | 17% | 22% | 21% | 23% | 100% |  |  |  |
| **T2R_alligator** | 15% | 15% | 13% | 15% | 16% | 16% | 18% | 20% | 20% | 18% | 17% | 17% | 16% | 14% | 100% |  |  |
| **T2R1stickleback** | 16% | 15% | 10% | 10% | 11% | 10% | 10% | 11% | 11% | 8% | 10% | 14% | 12% | 11% | 10% | 100% |  |
| **T2R3_Zebrafish** | 18% | 15% | 14% | 14% | 8% | 10% | 10% | 10% | 10% | 10% | 12% | 10% | 10% | 9% | 10% | 27% | 100% |
|  | **hT2R10** | **hT2R46** | **T2R11_frog** | **T2R20_frog** | **v1r3_chimp** | **v1r4_BOrang** | **V1r69_mus** | **v1r_frog** | **V1r_Zebrafish** | **V1R_alligator** | **V1r62_mus** | **V1r105_rat** | **V1r96_rat** | **v1r_RMacaq** | **T2R_alligator** | **T2R1stickleback** | **T2R3_Zebrafish** |
